# Skull morphology of the extinct Tasmanian tiger suggests unique biting style

**DOI:** 10.1038/s41467-026-76614-0

**Published:** 2026-08-31

**Authors:** Vera Weisbecker, Andrew J. Pask, Axel H. Newton, Douglass S. Rovinsky

**Affiliations:** 1 College of Science and Engineering, Flinders University, Bedford Park, SA Australia; 2 Australian Research Council Centre of Excellence for Australian Biodiversity and Heritage, Wollongong, NSW Australia; 3 School of BioSciences, The University of Melbourne, Parkville, VIC Australia; 4 Australian Museum, Sydney, NSW Australia

**Keywords:** Palaeoecology, Evolutionary developmental biology, Biomechanics, Palaeontology

## Abstract

The recently extinct thylacine (Tasmanian tiger) was the largest modern marsupial predator. It is considered a classic example of evolutionary convergence due to striking similarities with placental canids (e.g., foxes and wolves), particularly in the skull, despite ~160 million years of evolutionary separation. However, we here present geometric and linear morphometric evidence that the thylacine’s cranial form arises from a mosaic of traits not represented among canids or other living mammalian carnivores. Thylacines had disproportionately large heads, tall and gracile snouts with a flared canine region, and conspicuously large infraorbital foramina. Many of these traits suggest adaptations to fast, high-impact snapping behaviour in prey capture, as proposed for several living and extinct predatorial vertebrates with similar trait combinations. The thylacine’s cranial function may therefore not be inferable from observation of living mammals. However, genomic progress presents new opportunities for future insights into the evolution and development of thylacine cranial adaptation.

## Abstract (web summary)

The recently extinct thylacine was Australia’s largest marsupial predator and famously likened to dog-family carnivorans such as wolves. However, here the authors show that its skull functioned differently in prey capture compared to any living mammalian carnivore.

## Introduction

**Fig. 1:**
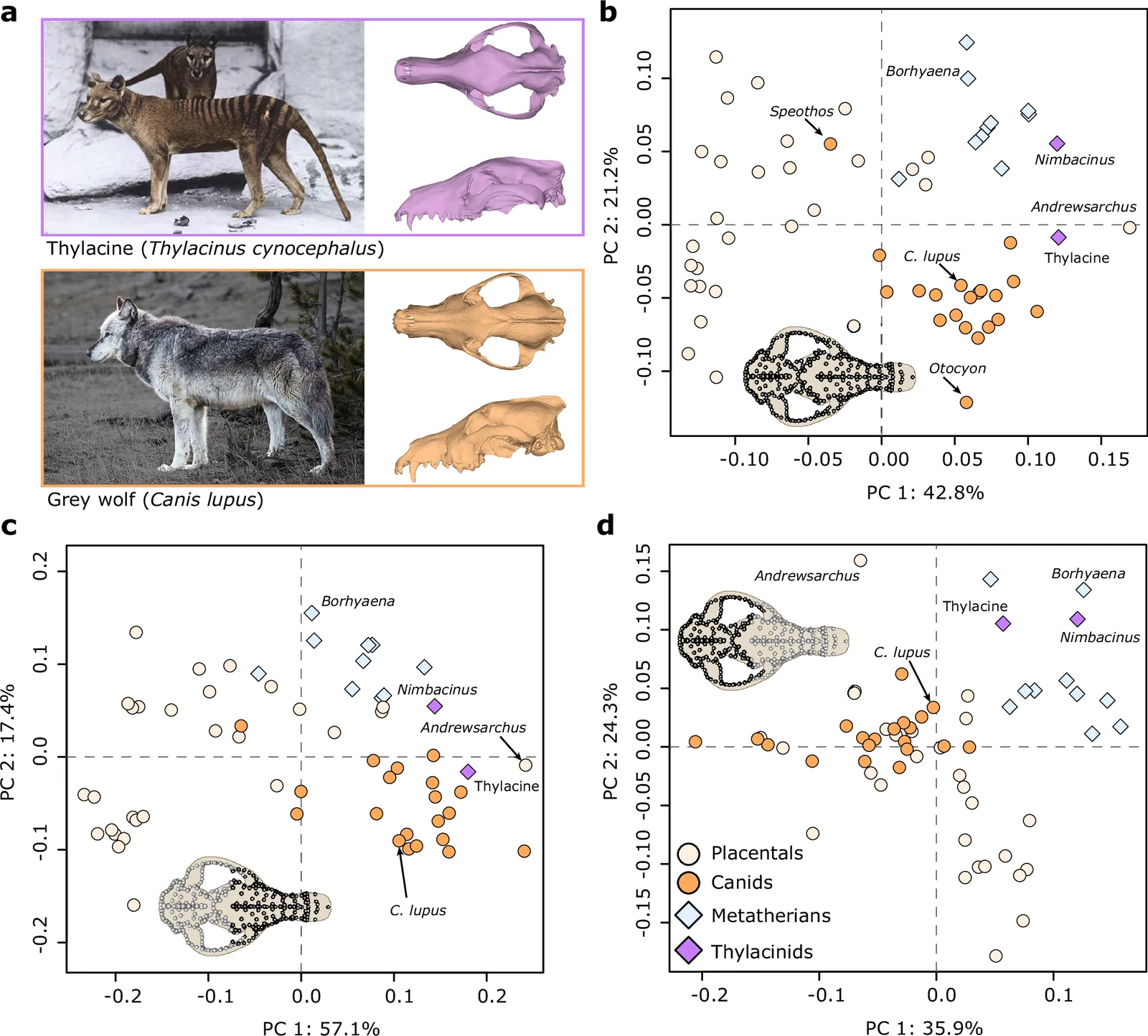
Principal component plots of all partition shapes. Photographs of the thylacine and wolf with dorsal and lateral views of the cranium (**a**); Principal Components (PC) Analysis plots of PC axes 1 and 2 of the thylacine cranium (**b**) and its rostral (**c**) and neurocranial (**d**) partitions in context with carnivorous metatherians, carnivorans, and a 3D replica of the exceedingly long and slender-snouted whippomorphan *Andrewsarchus. Andrewsarchus*, *Andrewsarchus mongoliensis*; *Borhyaena*, *Borhyaena tuberata*, *C. lupus, Canis lupus; Nimbacinus*, *Nimbacinus dicksoni, Otocyon, Otocyon megalotis, Speothos, Speothos venaticus;* Thylacine photo by E.J.K. Baker, Report of the Smithsonian Institution 1904, public domain, colourised by the authors; Grey wolf photo by Neil Herbert, Yellowstone National Park, public domain. Data and code to generate this figure are available in the GitHub repository for this paper^112^.

Few extinctions have captured public imagination like the recent loss of the world’s largest modern hypercarnivorous marsupial, the Australian thylacine (*Thylacinus cynocephalus*, extinct in 1936 and popularly known as the ‘Tasmanian tiger’; Fig. 1a). The thylacine’s similarity in appearance to grey wolves (*Canis lupus;* Fig. 1b) contributed to an assumed reputation as a livestock predator. This association prompted, in part, the rapid extermination of the already rare species by British colonisers within 130 years, despite little evidence of meaningful impact on the livestock economy^1^. The thylacine’s extinction marked the end of a >30-million-year lineage^2^ of relatively large-bodied carnivorous marsupials. Unfortunately, this lack of close living relatives means that our understanding of this iconic species’ ecology, and much of its evolution, is limited to anecdotal wild observations^1^ and comparisons with living mammals that it may have resembled.

Research into the thylacine’s ecology has often been conducted through comparative studies of the cranium (skull without the mandible), a useful source of information on mammalian feeding and sensory ecology^3^. The thylacine’s skull visually resembles that of the grey wolf and other canids, a feature reflected in its species epithet *cynocephalus* (‘dog-headed’) and quantitatively supported by several studies^4–8^. Thylacine cranial growth is also more similar to that of grey wolves than to other marsupials in bones originating from neural crest-derived frontonasal process and paraxial mesoderm tissues^6^. In addition, thylacines and grey wolves share strong positive selection on regulatory sequences for genes involved in skull growth patterning^6,9^.

The similarities between cranial shape, growth, and genomic selection are striking because thylacines and canids are marsupial and placental mammals, respectively. These two clades are separated by 122–166 million years of evolution^10^, differ substantially through craniodental and skeletal apomorphies^2,11–13^, and their cranial morphology is statistically distinguishable^14,15^. Differences between marsupials and placentals have been attributed to evolutionary constraints, such as phylogenetic contingency^16^ or considerable differences in reproductive and developmental biology (particularly the derived^17^ requirement for highly immature marsupial neonates to attach to the mother’s teat^6,18^). Because thylacines and canids are deeply nested within their respective radiations, they are popularly and scientifically perceived as a famous case of ecological convergence^4,6,16,19–22^, the independent evolution of similar phenotypes often attributed to selective pressures favouring similar adaptations^20,23,24^.

To successfully link functional, developmental, and genomic convergences in thylacine and canid cranial evolution, it is important to establish whether their similarities arose under selection for comparable functions. However, despite their overall canid-like appearance, thylacine crania display a mosaic of features that complicates the identification of canids with morpho-functionally comparable crania. For example, the thylacine snout is gracile, like in small-bodied, small-prey-focused canids, while the neurocranium groups with larger-bodied canids routinely taking large prey^7^. This aligns with biomechanical results that, despite potentially being able to generate relatively high bite forces^25^, the thylacine cranium is less suited to high stresses incurred in taking large, struggling prey^16,26–28^ compared to the robust crania of predators like grey wolves. Dentally, thylacine canines most resemble the related but much smaller dasyurid quolls, which use repeated penetrate-and-release bites instead of slashing/pulling bites of large canids or penetrate-and-hold bites of felids^16,29^. In addition, most canids frequently consume non-vertebrate food and have well-developed, complex crushing molars^12^. By contrast, the hypercarnivorous thylacine’s post-canine teeth have less occlusal complexity, emphasising shearing crests more so than canids^11^. Lastly, thylacines are not just canid-like; they also share extensive, independently derived morphological similarities with the extinct hypercarnivorous metatherian sparassodonts (e.g. refs. ^30,31^).

The lack of a clear canid morpho-functional analogue limits scientific understanding of how the thylacine acquired its prey. It also substantially constrains efforts to link shared patterns of cranial adaptation with genomic and developmental similarities of thylacines and grey wolves. We here address this through functional co-interpretation of diverse aspects of the thylacine’s cranial morphology, informed by first-principles biomechanics and known ecomorphological functions of particular anatomical traits in living faunivorous mammals and selected fossils. We assess shape variation in functionally and developmentally defined regions within the cranium, incorporate cranial size as a widely overlooked indicator of bite force^3^, and combine this with comparative information on within-species scaling patterns and previously unexplored traits such as anteriorly flared snouts and large infraorbital foramina. This confirms the thylacine cranium as a mosaic of functional traits without apparent living analogue among mammalian faunivores. The trait combination most resembles several extinct vertebrate predators possessing a relatively large head with long, gracile, and anteriorly widened jaws that would have produced fast, high-impact bites at the canines. The genomic and developmental similarities between thylacines and grey wolves are thus likely unrelated to selection for similar cranial functionality. Our results offer new perspectives on how the origin of thylacine-canid genomic and developmental similarities can be assessed; they also facilitate the integration of molecular and evolutionary evidence for tracing the emergence of the thylacine’s mode of prey capture.

## Results and discussion

### Thylacine-canid similarity varies with cranial region and clade

Our geometric morphometric analyses of a 60-species dataset, covering 14 families of faunivorous mammals (Supplementary Fig. 1; based mostly on a previously published^7^ sample), agree with previous results^4,7^ that the thylacine’s overall cranial shape is distinct from that of its marsupial relatives, and is intermediate between canids and metatherians (marsupials and their extinct relatives^18^). PC1 (43% of shape variation) represents a spectrum of crania with short, robust (e.g., felids) *versus* long, gracile faces (e.g. thylacine and canids; Fig. 1b). PC2 (21% of shape variation) separates species with high-vaulted midfaces and neurocrania (some felids and most canids) from species with low-vaulted neurocrania (some felids and most metatherians). As in previous studies^4,7^, we found near-complete overlap between metatherians and canids on PC1, and the thylacine resembling canids in having low scores on PC2. Additionally, the thylacine and its Miocene relative, *Nimbacinus dicksoni*, have the highest whole-cranium PC1 scores except *Andrewsarchus mongoliensis*, separating them from the entire sample (Fig. 1b).

**Fig. 2:**
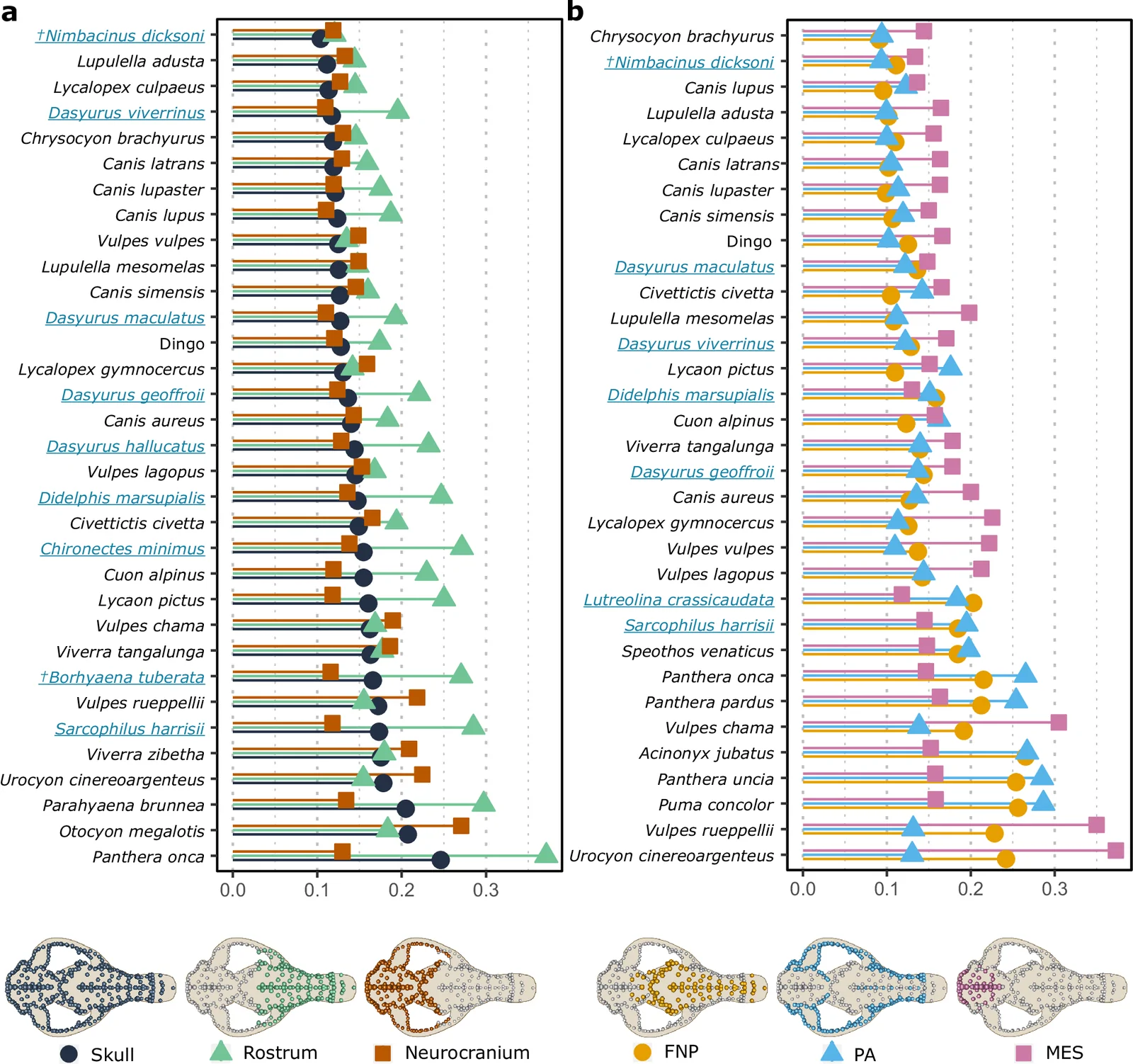
Distance in Procrustes shape between the thylacine and other carnivorous mammals. Distances calculated from the mean 3D Procrustes shapes of the 20 species closest in Procrustes distance to the thylacine in any landmark partition (see ‘Methods’). Full landmark configuration, rostral and neurocranial functional partitions in **a**, ordered by increasing distance in the full configuration. Marsupial species names are underlined. Developmental partitions in **b**, ordered by increasing mean distance of all developmental partitions. Note that the distances are from separately Procrustes-aligned datasets for each landmark configuration, so that distances are comparable between but not within species; note also that differences in distance might not be statistically significant and are for orientation only. For all Procrustes distances, refer to Supp. Table 1. FNP, Fronto-nasal Process; PA, First Pharyngeal Arch; MES, Paraxial Mesoderm. Data and code to generate this figure are available in the GitHub repository for this paper^112^.

The intermediate shape of the thylacine cranium is also reflected in our Procrustes distance plots (Fig. 2a, blue circles; Supplementary Table 1), which are useful in showing whole-shape similarities in species that can appear distinct along different axes of ordinated shape variation^32^. As previously suggested^7^, species most similar to the thylacine in overall shape are mostly mid-sized small-prey specialist canids such as jackals and foxes. In addition, the related but much smaller-bodied (>~5 kg) marsupial quolls fall close to the thylacine in overall Procrustes shape.

Dividing the cranium into partitions shows that the thylacine’s overall intermediate cranial shape arises from complex and at times contrasting sub-patterns within landmark configurations, apparent from the Principal Components Analysis (PCA) plots and Procrustes distances of functional^7^ (rostrum, neurocranium) or developmental^6^ (neural crest-derived first pharyngeal arch [PA] and frontonasal process [FNP], and paraxial mesoderm [MES]) landmark partitions (Figs. 1c, d and 2, and Supplementary Figs. 2–4). Overall, thylacines are closer to most canids in the rostrum and PA region (Figs. 1c and 2, and Supplementary Figs. 2b, 3b and 4b), and closer to most metatherians in the neurocranium and (to a slightly lesser degree) MES region (Figs. 1d and 2, and Supplementary Figs. 2c, 3c and 4c). This further supports the hypothesis that the thylacine cranium should show patterns of similarity with canids specifically within bones of neural crest origin, but less so in neurocranial bones from the head mesoderm^6^. However, the overall similarities of the thylacine with the canids are differently patterned according to partition and clade. Rostrally and in the PA/FNP region, thylacines are most similar to foxes (Vulpini, with the exception of the Arctic fox *Vulpes lagopus*) and the Canini *Chrysocyon* (maned wolf), *Lycalopex* (South American foxes), and *Lupulella* (black-backed/side-striped jackal) (Figs. 1c and 2, and Supplementary Fig. 2a, b, 3b and 4a, b). The opposite is true for the remaining Canini (*Lycaon, Cuon* and *Canis*), whose neurocranial and MES partition shape places them similarly close to the thylacine as its marsupial relatives, like *N. dicksoni* and quolls (*Dasyurus* spp.), whereas their rostral partition is mostly further away from the thylacine compared to the other canids (Figs. 1 and 2, and Supplementary Figs. 2–4). Compared to Vulpini, Canini also resemble the thylacine more in the FNP partition, which covers the top of the nasal and orbital region (Fig. 2b, and Supplementary Fig. 4a).

The PCA and Procrustes distance comparisons show that the thylacine cranium combines anterior and posterior shapes that do not co-occur among the canids or metatherians in our sample. Moreover, because of the opposing patterns of rostral *versus* neurocranial similarity with the thylacine, either the Vulpini or the Canini *Lycaon/Cuon/Canis* must have departed from the ancestral condition to display morphological similarity with the thylacine in one cranial area and divergence in the other. The cranial shape of *Lycaon/Cuon/Canis* is most likely the derived condition among canids, since this group has evolved cranial modifications such as shorter, broader rostra and greater jaw muscle leverage on the cranium as part of their derived style of regularly hunting in packs and preying on animals their size or larger^33^. The similarities between these canids and the thylacine are also aligned with previously noted developmental growth similarities in the MES and potentially FNP regions^6^ in wolves and thylacines. Owing to the discrete developmental origins of these cranial regions, they provide interesting candidates for uncovering the genomic basis behind canid and thylacine cranial adaptation, even if they may not share a similar morpho-functional context.

### Thylacine crania are far larger than expected for body mass and shape

**Fig. 3:**
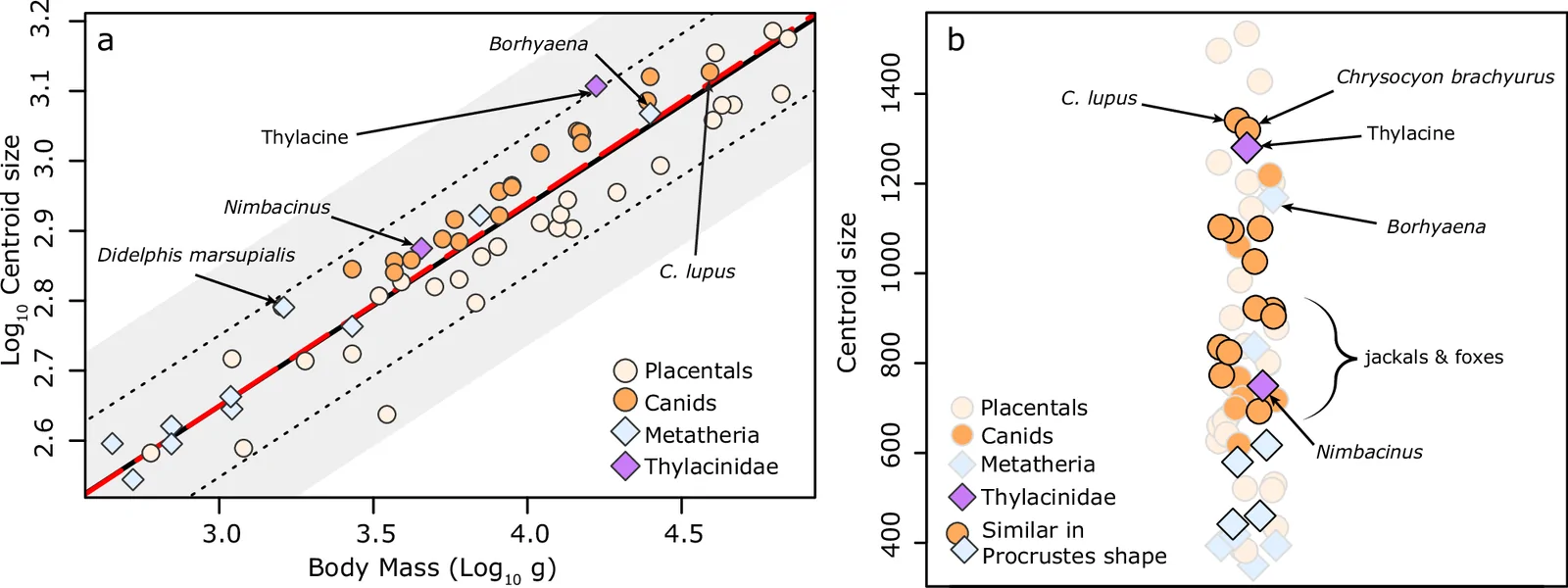
Comparison of Thylacine cranial centroid size relative to the landmarked sample. Phylogenetic Generalised Least Squares (PGLS) regression of log_10_-transformed centroid size against log_10_ body mass (**a**). Solid black regression line and associated 95% confidence interval (black dotted lines) and 95% prediction interval (grey area) calculated from PGLS regression with the thylacine removed. Red dashed PGLS regression line is from the full data set, showing the effect the thylacine has on the interpretation of the data. Untransformed centroid size (**b**) strip chart with slight jitter to show raw centroid sizes of all species, showing the thylacine’s cranium size is far larger than most of the similar species in Procrustes shape (Fig. 2a). *Borhyaena*, *Borhyaena tuberata*; *C. lupus, Canis lupus; Nimbacinus*, *Nimbacinus dicksoni*. Data and code to generate this figure are available in the GitHub repository for this paper^112^.

Thylacine crania are far larger than expected for their body mass, as determined by their centroid size (Fig. 3a), and exceed the absolute cranial sizes of far larger marsupials such as red kangaroos (Supplementary Fig. 5). While the thylacine weighs ~17 kgs^34^, its cranial size (within ±10%) is similar to that of much larger species, between 24.5 and 66.7 kg (Fig. 3a). In contrast, most placental carnivorans that are close to the thylacine in overall cranial shape (Figs. 1b and 2a) are jackals and foxes weighing 6–14 kgs (Fig. 3b). The cranial size of the thylacine is on par with much heavier predators such as the grey wolf, African wild dog (*Lycaon pictus*), puma (*Puma concolor*), and leopard (*Panthera pardus*). Compared to the species closest to the thylacine in overall Procrustes distance, the thylacine skull is far larger than all but the grey and maned wolves (Fig. 3b); this may explain why it has been considered wolf-like in appearance.

There is no indication that the shape of the thylacines’ cranium is predicted for its large size due to allometric patterning (shape variation associated with size changes; Supplementary Note 1). Size explains some shape variation in all partitions (Supplementary Figs. 6–8, and Supplementary Table 2), but it does not dominate the main variation; rather, allometry exists across multiple Principal Components of shape variation (Supplementary Table 3). Phylogenetic Generalised Least Squares (PGLS) analysis of allometry within the major clades (feliforms, caniforms, and metatherians) revealed that size explained different amounts of variation in each clade across partitions (Supplementary Table 4). Linear Procrustes models further indicate significant interaction effects (scaling slope differences) of shape and size between clades for most partitions except the neurocranial and MES partitions, where slopes were parallel (Supplementary Table 5). In addition, mean shape differences between clades explain much more shape variation (25–33%) than either the interaction effects or size alone (7–13% of the variation) (Supplementary Table 5). Pairwise comparisons of slope differences showed consistently differing scaling slope magnitudes and angles between caniforms *versus* marsupials and feliforms (Supplementary Table 6, and Supplementary Fig. 9), an effect also visible in divergent allometric shape deformation plots between predicted shapes for the smallest and largest individuals (Supplementary Fig. 10); similarities between the larger canids and thylacines are therefore not due to their commonality of having large crania on a joint allometric slope. As would be expected for a functionally and phylogenetically diverse dataset^3^, diverse allometric effects therefore exist in the sample, but these do not predict the thylacines’ cranial shape.

### Gracile crania suggest faster bites in larger thylacines

**Fig. 4:**
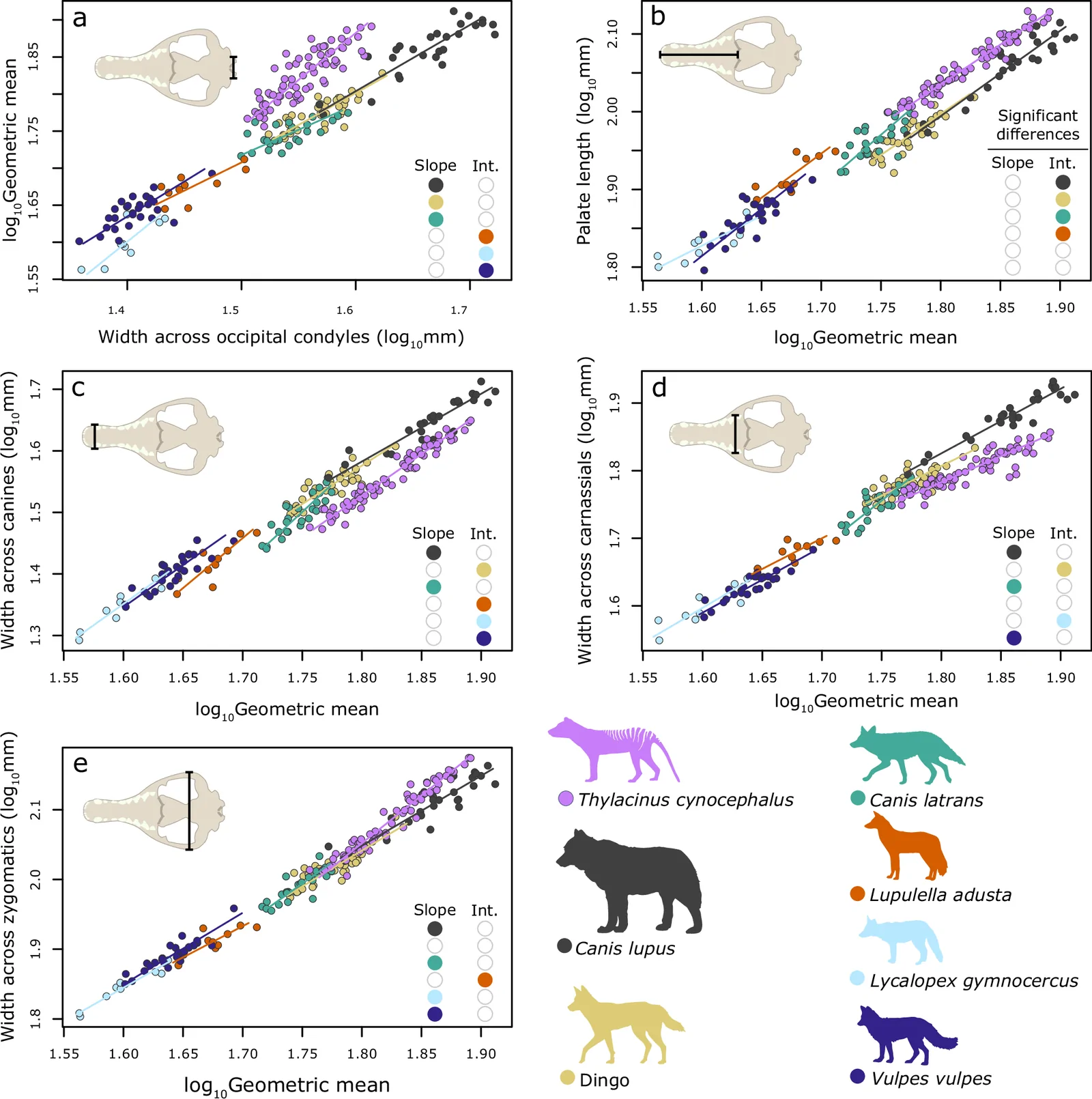
Linear cranial measurement regressions of the thylacine and a selection of other canids. Occipital condyle width (**a**), palate length (**b**), rostral width at the canine (**c**) and carnassial (**d**; M3/m4 in the thylacine, P4/m1 in carnivorans), and cranial width across the zygomatic arches (**e**). All metrics log_10_ transformed and reduced major-axis regressed on the geometric mean of nine cranial linear metrics (see ‘Methods’). Dots indicate significant differences between the thylacine’s scaling slope and intercept (Int.) for each measurement (for detailed outputs, see Supplementary Table 7). Axes in **a** are flipped to better illustrate the much larger cranial size of the thylacine cranium relative to the condyle. Int., Intercept. Data and code to generate this figure are available in the GitHub repository for this paper^112^.

The thylacine’s combination of unusually large crania with gracile rostral proportions is also maintained across cranial sizes. This is apparent from within-species (static) scaling of linear measurements that reflect cranial size relative to the body (occipital condyle width^35^) and relative gracility (palate length and cranial width across the canines, the carnassials, and zygomatic arches; Fig. 4, and Supplementary Table 7). Compared to grey wolves and several canids with similar rostral shapes in Procrustes space (Fig. 2a), the cranial size of thylacine individuals increases disproportionately with the body mass proxy of occipital condyle width (Fig. 4a). Consistent with the geometric morphometric analyses, the thylacine has a significantly longer rostrum length (measured as palate length) compared to all but the smallest species (Fig. 4b). It also is narrower at the canines relative to the geometric mean than nearly all comparative species (Fig. 4c). Facial width across the carnassials is also narrow compared to wolves and the Pampas fox, and becomes significantly narrower as cranial size increases compared to grey wolves, coyotes, and red foxes (Fig. 4d). In contrast to the increasing gracility of the rostrum with size, the zygomatic arches become significantly wider with cranial size compared to four of the six canids in the sample, and are overall larger than a fifth species (Fig. 4e).

The gracile snout in the thylacine, demonstrated in the between-species shape variation and within-species scaling comparisons, is consistent with earlier suggestions that the thylacine’s cranial shape does not appear adapted to high stresses^26–29,36^ commonly associated with strong bite forces, struggling prey, or post-bite behaviour such as shaking or pulling^33,37–40^. Moreover, compared to shorter-faced species, an elongate jaw like the thylacine’s has a longer out-lever and thus allows faster jaw closure^3,41^. This morphology is associated with the capture of small, fast-moving prey^42^, but indicates a lack of bite force and resilience to torsional stress^3,41,42^. However, size is a main point of difference in all our comparisons: large-prey specialist canid crania are large but not gracile, small-prey specialist canid crania are gracile but small, and all canids and most methatherians had a much smaller cranium compared to body size.

### Large thylacine crania may increase bite force resistance

The large size of the thylacine cranium has not been integrated into interpretations of its function, possibly because morphometric analyses generally remove absolute size^43,44^ and biomechanical comparisons usually focus on comparative, rather than absolute, generation of bite force^44^. However, a large cranium counteracts the weaker structure of a long and narrow rostrum by increasing the absolute magnitude of rostral resilience to stress and bite force. This is because larger crania are more resilient to intrinsic and extrinsic loads^3,44^ and carry more muscle^3^ compared to smaller crania that share the same shape. It is also possible that the wide zygomatic arch compared to other canids, particularly in larger thylacines (Fig. 4e), plays a role in buttressing the jaw joint against the leverage of the long jaw^3,45^, but this remains to be tested. Overall, the large size of the thylacine cranium thus indicates a specific mechanism of prey capture and/or immobilisation optimised for fast, high-impact bites whose force was dissipated across a large volume of cranial bone.

### Unique ‘terminal rosette’ of thylacine upper jaws

**Fig. 5:**
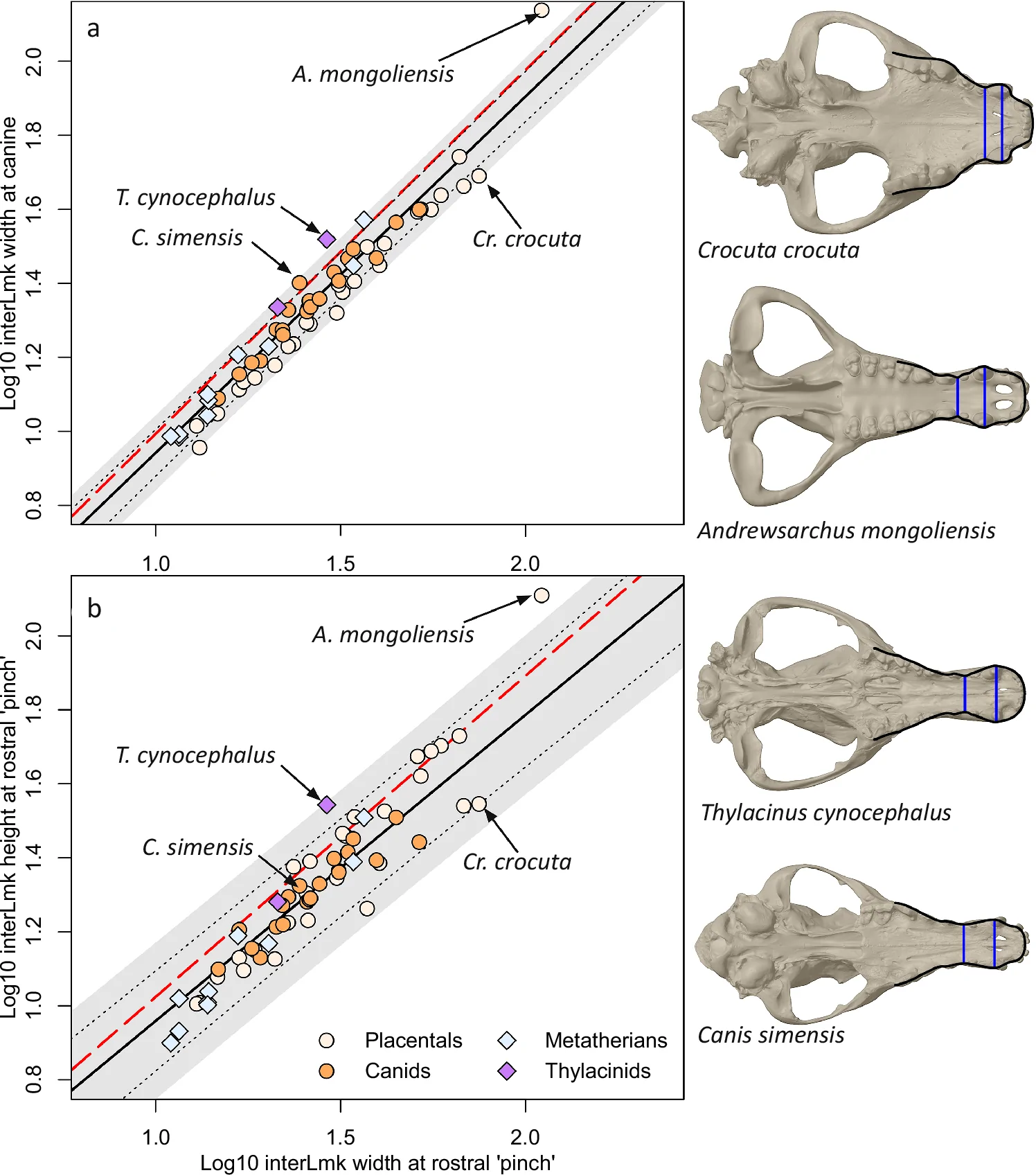
PGLS regressions of rostral ‘pinch’ measurements. PGLS regression of log_10_-transformed interlandmark distance of the least width across the palate against the greatest width across the canines (**a**) and the log_10_-transformed interlandmark distance between the midline of the palate and the midline of the rostrum just anterior to the ‘pinch’ measurement (**b**). Solid black regression line and associated 95% confidence interval (black dotted lines) and 95% prediction interval (grey area) calculated from PGLS regression with the thylacine and *Andrewsarchus mongoliensis* removed. Red dashed PGLS regression line is from the full data set, showing the effect these two taxa have on the analysis. Call-outs illustrate species with high residuals in **a**. *Andrewsarchus*, *Andrewsarchus mongoliensis; C. simensis, Canis simensis; Cr. Crocuta, Crocuta crocuta; T. cynocephalus, Thylacinus cynocephalus*. Data and code to generate this figure are available in the GitHub repository for this paper^112^.

Intriguing additional support that the thylacine could have used fast jaw closure to capture and potentially subdue prey comes from its rostral ‘terminal rosette’: an expanded anterior rostrum at the canines directly followed by a mediolateral ‘pinching’ of the maxilla and palate. This trait is pronounced in the thylacine compared to the remaining sample, except our 3D replica of the gigantic early Eocene stem-whippomorphan *A. mongoliensis* (Fig. 5a). The terminal rosette moves the mass distribution in the rostrum forwards. This imparts greater rotational inertia to the tip. The greater lateral and dorsal extent of the rostrum relative to the post-canine ‘pinch’ (Fig. 5) also increases the amount of hard tissue available to resist the impact around the canines^3,46^. Additionally, while the rostra of the thylacine and *A. mongoliensis* are medio-laterally narrow behind the terminal rosette (Fig. 5a), they are relatively taller than any of our samples (Fig. 5b). This altirostral shape is rare among living mammals^3^, but well-suited to distributing forces acting in a dorsoventral direction while sacrificing resistance against mediolateral or torsional forces^3,40,47^. Capacity to buffer high-impact snapping with the canines could also explain why the thylacine’s canines are sturdier than those of grey wolves and appear suited to handle forces otherwise only experienced in puncturing or crushing bites^29^. The tall, elongate jaw with an expanded terminal rosette is thus also consistent with a relatively fast (for its size), powerful snapping bite used to acquire relatively small, potentially agile prey.

### Large infraorbital foramen suggests high snout sensitivity

**Fig. 6:**
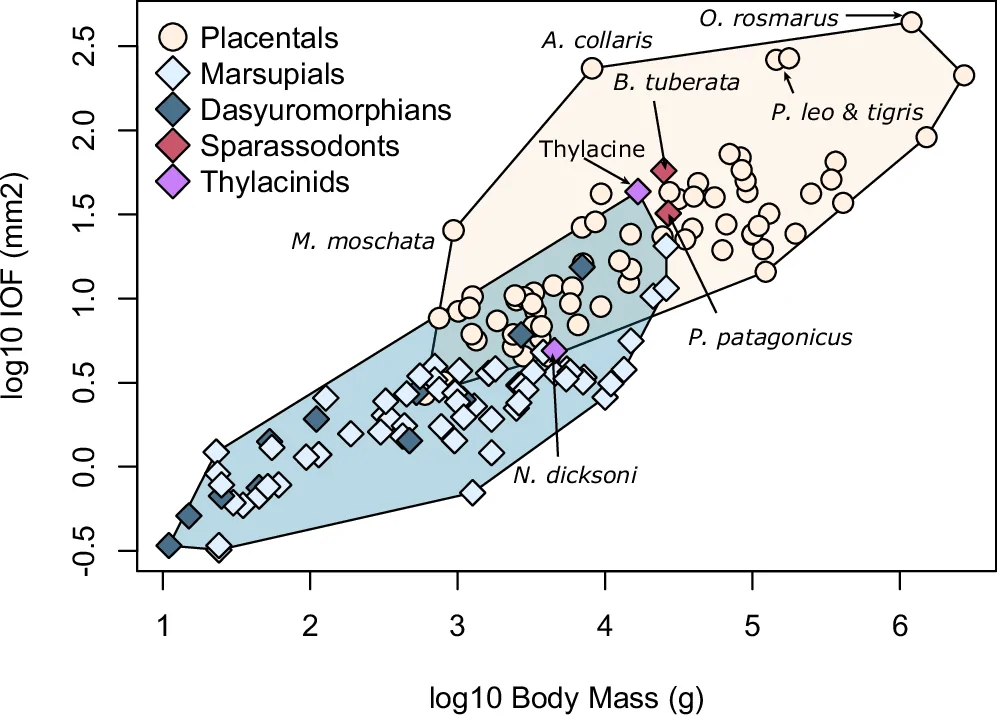
Log _1__0_ Infraorbital foramen size relative to log_10_ body mass in metatherians and placental carnivorans. The convex hulls envelop metatherians (blue) and placentals (beige). *A. collaris*, *Arctonyx collaris* (hog badger); *B. tuberata*, *Borhyaena tuberata*; *M. moschata*, *Melogale moschata* (Chinese ferret-badger); *N. dicksoni*, *Nimbacinus dicksoni*; *O. rosmarus*, *Odobenus rosmarus* (walrus); *P. leo & tigris*, *Panthera leo and tigris* (lion and tiger); *P. patagonicus*, *Prothylacinus patagonicus*. Data and code to generate this figure are available in the GitHub repository for this paper^112^.

Additional, albeit circumstantial, evidence for an unusually specialised thylacine rostrum comes from the very large size of its infraorbital foramen (IOF; Fig. 6), which by far exceeds the absolute and relative size recorded for any living marsupial (Fig. 6, and Supplementary Fig. 11). A large IOF is also present in the borhyaenoid sparassodonts *Prothylacinus* and *Borhyaena* (Fig. 6^48^). The diameter of the mammalian IOF—including that of marsupials—is largely explained by the size of the maxillary branch of the trigeminal nerve (V_2_)^49,50^. No thylacine dissection data exist to assess the soft tissues housed by the IOF, but we assume the same for thylacine. The portion of the mammalian V_2_ that emerges from the IOF confers somatosensory sensitivity to the cheeks, upper lips, external nose, and mystacial vibrissae (whiskers)^51^. Large IOFs are frequently, but not exclusively^52^, found in species which rely on well-developed whiskers to sense their environment or prey, such as (semi-)aquatic mammals^49,50^ and many rodents^51^. However, this is unlikely to apply to the thylacine, whose whiskers have been described as conspicuously short and fine compared to dasyurid relatives^53,54^. In felids with large canines and sabretooth predators, a large IOF has been connected with increased sensitivity of the whiskers and lips to support precision placement of the canines during prey acquisition^55^, although this has not been tested directly. Borhyaenoid sparassodonts (like *Borhyaena* and *Prothylacinus* in Fig. 6) were also proposed to have employed powerful, canine-driven bites^56^, similar to what is observed in dasyurids and was probably also the case for thylacines^16,29^. The combination of a large IOF and large, over-erupting canines could therefore relate to a shared specialised use of thylacine and sparassodont canines during biting via sensory input from the anterior face. Ultimately, however, no clear explanation can be offered for this unusual trait because not enough is known about its involvement in biting behaviour even in living mammals.

### Thylacine crania lack living mammalian functional analogues

The biomechanical properties of the thylacines’ large cranium - with a gracile, tall rostrum and terminal rosette - have no modern mammalian functional analogue we are aware of. Differences between thylacines and canid carnivorans are thus not a result of similar functionalities arising from divergent ancestral conditions. Rather, they relate to substantial differences in how the cranium functions in prey capture. Our findings thus highlight the complexities of investigating a multi-functional structure such as the cranium, emphasizing the need to examine cranial components separately to understand how mammalian cranial adaptations arise^3,57^. For example, the most similar canid to the thylacine in terms of shape and size combined is the maned wolf (*Chrysocyon brachyurus*). This is the 5th-closest species in Procrustes distance (Fig. 2), still roughly 50% larger than the thylacine (~25 kg vs. ~17 kg), but with a slightly larger cranium (Fig. 3b). The maned wolf’s vertebrate prey consists almost entirely of rodents, and it has an unusually high consumption of fruit (~50% of its diet)^58,59^. The use of its cranium in the capture of small prey may be useful in approximations of the thylacine’s mode of action, but its frugivorous diet, smaller cranium relative to body mass, and extremely slender, long-limbed body^60^ make the maned wolf a poor ecological comparison to the hypercarnivorous^11,29^, short limbed^22^ thylacine that took relatively small (i.e. 45% of its own body mass^34,61^), fast-moving prey. Similarly, the only other placental in the dataset with an elongate jaw and a pinched snout is the Ethiopian wolf (*Canis simensis;* Fig. 5a). This species could be useful for approximating aspects of the thylacine’s rostral function because it is roughly the same size (~14.5 kg) and preys almost exclusively on small, quick-moving prey such as rodents and hares. However, this species differs from the thylacine by lacking a dorsoventrally tall snout and having a smaller relative cranial size (Figs. 2 and 3), suggesting substantial differences in absolute bite force and biomechanical properties of the cranium. Lastly, the only carnivorous mammal in our sample with a similarly large cranium relative to body mass is the much smaller marsupial common Opossum (*Didelphis marsupialis;* Fig. 3a, and Supplementary Fig. 5), which therefore could serve as a model for how the cranium was moved relative to the body; however, the snout shape of this species is among the least similar in our Procrustes distance plots (Fig. 2a) and thus unlikely to be functionally equivalent.

### Moving forward with fossils, biomechanics and genomics

Insights on thylacine cranial function could also come from beyond mammals. For example, large crania with long rostra and widened or heightened tips occur in aquatic or aquatic-feeding piscivorous vertebrates such as crocodiles, predatory fishes, *Spinosaurus*, and ornithocheirid pterosaurs. These are not ecologically analogous to the thylacine, which was clearly a terrestrial predator^13,62,63^ with no dental adaptations to piscivory^29^. However, their cranial morphologies—large skulls with elongate, sometimes altirostral jaws and terminal rosettes—are considered an adaptation for high performance in fast-snapping predation^38–40,47^ and, in the case of *Spinosaurus*, fast puncturing bites^46^. Interestingly, diverse extinct terrestrial mammals had a relatively similar morphology; see also ref.^3^. Aside from *A. mongoliensis*, these include members of lineages such as the metatherian sparassodonts (*Acyon*, *Cladosictis*) and placental entelodontids (*Archaeotherium*), hyaenodonts (*Hyaenodon*, *Pterodon*), mesonychids (*Mesonyx*, *Harpagolestes*), and amphicyonids (*Amphicyon*). It is therefore possible that the thylacine cranium showed a complement of biomechanical adaptations to carnivory that is now extinct among mammals.

The lack of alignment between the thylacine’s functional cranial trait combination and any carnivoran ecomorph is intriguing because carnivorans display a large range of morpho-functional trait combinations, showing many-to-one mapping of multiple cranial shapes to similar functions^41,64^. It might therefore be tempting to ascribe the lack of similarity between thylacines and canids to constraints^6,65^ on the evolution of finely-tuned morpho-functional trade-offs in carnivorans. This would align with a long tradition of viewing marsupials as developmentally and evolutionarily constrained compared to placentals^66^, even though support for this is mixed^14,32,67,68^. However, our results challenge this view by suggesting a functionally harmonious set of morpho-functional characters, some of which probably co-occurred before in mammalian evolution. It might therefore be useful to instead contextualise the thylacine’s cranial morphology as facilitated by developmental traits (e.g. extreme marsupial altriciality) and evolutionary contingency (e.g. crania evolving in the context of over-erupting dentition in marsupial carnivores^65^ versus carnassial shear in carnivorans), thus simply changing the likelihood of particular adaptations in the two clades, e.g. ref.^69^.

While the molecular underpinnings of the thylacine’s cranial morphology may not be easily traced in living animals, the completion of the thylacine genome^4,70^ can support investigations into the developmental changes associated with its cranial adaptation. In particular, convergent positive selection between wolves and thylacines was found in regulatory regions of genes involved in patterning the neural crest^4^, a known source of species-specific patterning and craniofacial evolution^71^. Understanding the developmental impacts of these convergences might support a developmental interpretation for the distinctive cranial traits of the thylacine, because many (such as terminal rosette, high transverse profile and large IOF) occur in neural-crest-derived FNP and PA regions^6,72^. Moreover, the enlargement of the equally neural-crest-derived^9^ maxillary branch of the trigeminal nerve might suggest that some of the thylacine’s cranial peculiarities could relate specifically to the convergent positive selection in trigeminal nerve enhancers between thylacine and wolf, because extensive signalling occurs between the peripheral nervous system and developing craniofacial structures in mammals^73^. Further comparative genomic research therefore represents an opportunity to determine how differential gene regulation could shape the development and diversity of the neural-crest derived FNP and PA regions, and potentially even help reconstruct the molecular basis of the thylacine’s phenotype compared to other mammals.

## Methods

### Acknowledgement of Indigenous data sovereignty concerns

We wish to acknowledge that the skulls in museum collections across the globe—and, by extension, the data we collected—were obtained without consent from or consultation with local Indigenous communities, implicating our data collection with the racist science practiced by early naturalists^74^. There is increasing awareness that scientists must consider how museum specimens might be integral to Indigenous kinship systems and worldviews, which can diverge sharply from frameworks that may govern collecting institutions^75^. Existing frameworks from the cultural heritage sector are currently rarely implemented in the Australian biodiversity sector. It is therefore difficult to identify how biodiversity research can be aligned with legal and ethical frameworks^76,77^ on Indigenous data sovereignty, such as Indigenous cultural and intellectual property rights^78^ regarding culturally significant animals and plants, which usually requires entering ethical collaborations with local Indigenous peoples and communities. However, we commit to engaging Indigenous communities—such as Palawa and other Indigenous communities with connections to the thylacine, e.g. as highlighted in artwork^79^—by following the custodial ethics of caring for Country embodied by First Nations communities^80^, and adhering to Indigenous data sovereignty, as best we can in future research on the thylacine and other museum holdings. This will be a critical next step in an ethical future for scientific investigations of this and other culturally significant fauna in Australia and elsewhere^81^.

### Analyses and figure creation

All data manipulation and analyses were performed in R v4.5.2^82^, unless otherwise noted. All figure plots were also done using R functionalities, and in most cases combined with text labels and illustrative figures or drawings via Adobe Illustrator (v. 28.4.1) or InDesign (v.19.3) by Adobe Inc., San Jose, CA, USA

### Geometric morphometrics

Our dataset is based on an existing three-dimensional (3D) landmark dataset^7^, consisting of 222 specimens from 57 mammalian faunivorous mammals, covering 12 families (Supplementary Data 1). The dataset focuses on mid-sized, mostly carnivorous species – those between 5 and 50 kg with diets consisting primarily of vertebrate flesh – for comparison with the ~17 kg hypercarnivorous thylacine (for more detail, see ref.^7^. The protocol included 381 landmarks (46 fixed, and 191 curve and 144 surface semilandmarks; Supplementary Fig. 12), landmarked in Viewbox (v. 4.1.0.12; dHAL software, Greece). To this we added three new specimens: the Argentinian mid-Miocene sparassodont *Borhyaena tuberata* from the Yale Peabody Museum (YPM 15120), the Australian mid-Miocene thylacinid *N. dicksoni* from the Queensland Museum (QMF 36357)^26^, and a 3D replica (see below) of the Mongolian early Eocene stem-whippomorph *A. mongoliensis* housed at the American Museum of Natural History (AMNH 20135), bringing the total to 225 specimens from 14 families across Mammalia (Supplementary Fig. 1). Both *Borhyaena* and *Nimbacinus* required digital reconstruction, as they had undergone plastic and brittle deformation since burial. Brittle deformation was addressed by segmenting and moving individual bony elements back into position within Materialise Mimics software (v. 20). Small holes were then filled in the Artec Studio 14 Professional (Artec Group, Luxembourg). After this initial step, we adjusted for plastic deformation and performed mesh resurfacing in Blender software 3.2.2^83^, using bilateral symmetry to guide reconstruction. We finished the meshes with a final retrodeformation using the R package Morpho v. 2.13^84^, which uses paired landmarks to adjust for plastic deformation, particularly bilateral asymmetry (Morpho landmarks and script are included in our code). All these reconstruction steps were performed by DSR.

We are unaware of any existing 3D scans of the *A. mongoliensis* cranium, so we commissioned palaeoartist Alex James (alexanderjames2000@gmail.com/@Paleosculpts) to digitally reconstruct the cranium. This involved sculpting the cranium in Blender referencing Osborn^85^, cross-referenced with photographs of the holotype to account for potential errors in the description’s Figures. The teeth, which are relatively poorly preserved and not relevant for landmarking, were reconstructed referencing both the initial description^85^ and the description of *Paratriisodon*^86^, which is likely closely related to *A. mongoliensis*, if not a junior synonym, e.g.^87^.

We separated the total configuration of landmark coordinates into two functional partitions (rostral and neurocranial; see Fig. 1b–d) following Rovinsky et al.^7^. In addition, we separated partitions aligned with cranial areas^6^ that reflect embryological origins of the neural crest-derived frontonasal process (FNP) and first pharyngeal arch (PA), and the paraxial mesoderm (MES). These developmental partitions are visualised in Fig. 2b. All data manipulation and analyses were performed in R v4.5.2^82^, unless otherwise noted. We subjected each partition to separate generalised Procrustes superimpositions to remove the effects of translation, rotation, and scaling in the R package geomorph v. 4.0.10^88,89^.

### Cranial shape

We visualised the thylacine’s position relative to the other species in the dataset in two ways. We first employed PCA for the whole-cranium configuration and each of the partitions and plotted the first and second Principal Components (PCs) against each other. In addition, we assessed the similarities of the Procrustes distances (computed using the shapes R package v1.2.8^90^) between the thylacine and other faunivorous mammals across the partitions. Procrustes distances are a better approximation than PCA of how landmark configurations resemble each other overall, as they include the entire shape variation in the sample. This is an advantage over PCA because it does not partition shape into statistical main axes of variation, which may not be biologically meaningful^32^. We calculated the mean Procrustes shapes across the whole skull and each partition for the shape of each species, after which we calculated the distance of each from the thylacine. The results of each Procrustes distance subset were then ordered by increasing distance from the thylacine. For clarity of comparison, we visualised only species that were among the 20 closest species to the thylacine in any partition. We also used the contMap code of the Phytools package (v2.5-2)^91^ to visualise the distribution of distances from the thylacine for each partition, mapped onto the phylogenetic tree. Following a reviewer suggestion, we also trialled the use of Arbuckle’s Wheatsheaf Index^92^ as implemented by Grunstra et al.^93^, but for methodological concerns outlined in Supplementary Note 2 (Supplementary Fig. 13, and Supplementary Table 8), we did not present the results in the main manuscript.

Note that we chose to present our geometric morphometric analyses without accounting for allometry, because we expected allometric effects on cranial shape to differ among the widely disparate clades that our dataset was comprised of, limiting the biological meaning of any emerging allometric patterns^3,57,94^. However, we confirmed this expectation through extended allometric analysis in an extended results section. We first characterised the amount of shape variation associated with log-transformed centroid size across all partitions, for the whole sample and the major radiations of metatherians, caniforms, and feliforms, using the centroid sizes and superimpositions from separate GPAs. These analyses were conducted as PGLS analyses, but we also computed linear models without phylogenetic correction to align with the non-phylogenetic testing required below for between-clade allometry comparisons. We then asked if these overall allometries represented a dominant effect in our dataset by using PGLS analysis of the first ten Principal Components of our sample against log(centroid size). If allometric patterns dominate the variation in the sample, PC1 should be the only allometric PC. By contrast, if several independent allometric patterns exist in the sample, multiple PCs should be correlated with size^95^. We also assessed if the overall allometric pattern is an emergent property of multiple clade-specific allometric patterns, by using non-phylogenetically corrected linear Procrustes models with follow-up pairwise tests to assess if caniforms, feliforms, and metatherians were significantly different in allometric slope magnitude, angle, or mean. These analyses were further visualised through scatter plots of centroid size regression scores and ball-and-vector plots of predicted allometric deformations associated with small *versus* large centroid sizes (the visualisations were supported by the rgl R package (v.1.3.34)^96^). We used PGLS, procD.lm and plot.pgls functionalities in geomorph for allometric analyses of cranial shape, which uses the Randomized Residual Permutation Procedure (RRPP v.2.1.2)^97^ for significance testing, and the caper package’s (v.1.0.4^98^) pgls function for analyses of Principal Components against centroid size.

### Cranial size

To see if the thylacine cranium falls within the predicted range of cranial sizes relative to body mass in our sample, we extracted the centroid sizes from the Procrustes-transformed 3D landmarks of the total landmark configuration. We log_10_-transformed centroid sizes and regressed them against the log_10_-transformed species’ average body masses using PGLS via the gls function in the R package nlme v. 3.1-168^99,100^. As the working phylogeny is not ultrametric (it contains fossil tips), the correlation structure was provided a fixed weight from the computed variance/covariance matrix, and model fits were calculated under both Brownian and Pagel’s correlations, using restricted and maximized log-likelihood methods. Best fits were chosen based on comparisons of Akaike Information Criterion, Bayesian Information Criterion, and logLikelihood. We calculated the 95% prediction intervals of the regression using the gls.ci and gls.pi functions of the evomap R package v.2^101^, represented by the confidence intervals of the regression without the thylacine (prediction interval), and compared the position of the thylacine relative to this prediction interval. We also used the same fitting and plotting methods to compare thylacine cranial centroid size relative to body mass to a larger sample of marsupials, including non-faunivorous species, using a previously published marsupial landmark dataset^102^.

### Linear measurements of cranial robustness

To compare basic cranial proportions of the thylacine and several members of Canini and Vulpini without the multidimensionality of 3D landmark datasets^43^, nine linear metrics (condylobasal length, occipital condyle width, width across the carnassials, palate length, width across the canines, interorbital width, width across the postorbital processes, width at the postorbital constriction, and zygomatic breadth; see Fig. 4) were recorded from the crania of 73 thylacines (representing the largest published sample of measurements of this species) and disparate canid species: grey wolf (*C. lupus*; *n* = 27), dingo (*n* = 37), coyote (*Canis latrans*; *n* = 24), side-striped jackal (*Lupulella adusta*; *n* = 10), Pampas fox (*Lycalopex gymnocercus*; *n* = 9), and red fox (*Vulpes vulpes*; *n* = 27). Measurements were taken with calipers (Mitutoyo Digimatic 6”) from a variety of museum collections (Supplementary Data 2). Note that the position of carnassials in the thylacine was determined by the position of the carnassialised teeth, i.e. the teeth that form the carnassial shear. In thylacines, this is M3/m4, in carnivorans, it is P4/m1^36^. We then individually regressed a subset of five functionally relevant metrics (palate length, width of the rostrum at the canines, width of the palate at the carnassials, and width of the zygomatic arches) against the geometric mean (as a measure of overall size) of the full set. These represent the length and robusticity of the rostrum and the area available for the jaw-closing muscles. We also regressed the occipital condyle width against the combined-set geometric mean. Body mass correlates strongly with occipital condyle width^35^, so this acts as an additional assessment of whether the thylacine’s head size is unusual for its body mass. We then log_10_ transformed the data and performed major-axis regressions with the R package smatr v.3.4-8^103^, testing for correlation between the variables as well as difference in slope and elevation between the species. All pairwise comparison p values were adjusted using the Benjamini–Hochberg procedure^104^.

### Rostral ‘pinch’ analysis

The rostrum of the thylacine is notably pinched, narrow, and flares anteriorly, which has prompted informal comparisons with *A. mongoliensis*, e.g. ref.^105^^.^ To explore this, we used PGLS regression as described above, regressing the log_10_ transformed raw (i.e. not Procrustes-superimposed) interlandmark distances between the midpoints of the alveolar margin curve (narrowest point of the anterior rostrum) against the canine alveoli (widest point of the anterior rostrum) and the midpoint of the palate to midpoint of the rostrum just anterior of the narrowest point of the anterior rostrum (Supplementary Fig. 14). We then asked whether the thylacine and *A. mongoliensis* were unusual among mammals in the proportions of these measurements by assessing whether they fell outside the phylogenetic prediction intervals of the sample without thylacine/*A. mongoliensis*, again using functionalities of the evomap package^101^

### Infraorbital foramen size

We compiled data on the diameter of the infraorbital foramen (IOF) from Muchlinski^52^ and Milne et al.^106^. To this composite dataset, we added the Argentinian mid-Miocene sparassodont *Prothylacinus patagonicus* YPM 15700 along with *Borhyaena* and *Nimbacinus* (Supplementary Data 3). We imported 3D meshes of these additional specimens into Artec Studio 14 and measured their IOF area in mm^2^ via the *Measures > Sections* tool and sourced average body masses from the literature to serve as size proxies (Supplementary Data 3). The IOF area and body mass were log_10_ transformed and plotted against each other. Because several canid carnivorans are known to have very large IOF sizes^52^, the aim here was to visually contextualise the thylacine’s IOF size relative to this sample (rather than asking whether the thylacine was unusually sized relative to the sample); however, to assess if thylacines were unusually large in their IOF dimensions relative to marsupials, we used prediction interval comparisons as for the centroid size and rostral ‘pinch’ analyses.

### Phylogeny

For the majority of our analyses we used the composite phylogeny from Rovinsky et al.^7^, to which we added *A. mongoliensis*, *B. tuberata*, and *N. dicksoni* using Mesquite v.3.70 software^107^ (Supplementary Data 4, and Supplementary Fig. 1). The greater taxonomic breadth in the analysis of the IOF precluded easily forming a rigorous composite phylogeny. We downloaded the complete 10,000-tree dataset from Upham et al.^10^ from https://data.vertlife.org/. From this set, we calculated a Maximum Clade Compatibility (MCC) tree from a random sample of 1000 trees using the R package phangorn v.2.12.1^108^*. N. dicksoni* and the dingo were added to the MCC tree in Mesquite, and then we pruned the resulting composite tree to the total taxa set present in the study. Basic phylogenetic tree principles were then exploited to include the small number of taxa not present in the MCC tree. The non-marsupial metatherians *Pucadelphys andinus* and *Mayulestes ferox* were replaced by the sparassodonts *P. patagonicus* and *B. tuberata*, and their node depth and branch lengths adjusted accordingly. Both *P. andinus* and *M. ferox* are likely either plesiomorphic sparassodonts or occupy positions as early-branching outgroups to Sparassodonta (e.g. ref.^109^). Either position makes them as equidistant to crown-Marsupialia as Sparassodonta is, allowing their substitution for the two sparassodonts. Similarly, we substituted the position of *Felis bieti* for *Felis silvestris* as needed to match datasets, since *F. bieti* and *F. silvestris* both occupy equivalent positions relative to the other *Felis* in our IOF analysis (*Felis chaus*).

For reading in and manipulating trees, including matching quantitative data with taxa on the phylogenetic tree or subdividing datasets and their associated trees, we used operations from the ape v. 5.8-1^110^, caper v. v1.0.4^98^, and geiger v. 2.0.11^76,111^ packages.

### Reporting summary

Further information on research design is available in the Nature Portfolio Reporting Summary linked to this article.

## Supplementary information


Supplementary Information
Description of Additional Supplementary Files
Supplementary Data 1
Supplementary Data 2
Supplementary Data 3
Supplementary Data 4
Reporting Summary
Transparent Peer Review file


## Data Availability

The data generated or analysed in this study, including raw landmark coordinates, cranial measurements, infraorbital foramen measurements, and phylogeny are available on GitHub via Zenodo link^112^ and as Supplementary data alongside this publication (Supplementary data 1–4). Three-dimensional meshes which the landmark dataset is based on are on the MorphoSource repository ID 0000C1004 (https://www.morphosource.org/projects/0000C1004), except for the mesh of *Nimbacinus dicksoni*, which was provided upon our request by Prof. Michael Archer at The University of New South Wales, Australia.

## Code availability

The code to replicate all analyses, and code to recreate the plots for all figures, is available on GitHub via Zenodo link^112^.
