# Supplementary material for: Skull morphology of the extinct Tasmanian tiger suggests unique biting style
Source: Nat Commun. 2026 Aug 31;17:8729. doi: 10.1038/s41467-026-76614-0 (PMC13530167; doi:10.1038/s41467-026-76614-0)
Supplement: Supplementary file 1 [file 41467_2026_76614_MOESM1_ESM.pdf]

Supplementary Information for Weisbecker et al.: Skull morphology of the extinct Tasmanian tiger suggests unique biting style

Table of contents

Supplementary Figure 1 2
Supplementary Figure 2 3
Supplementary Figure 3 4
Supplementary Figure 4 5
Supplementary Figure 5 6
Supplementary Table 1 7
Supplementary Note 1 9
- **Supplementary Figure 6** 9
- **Supplementary Figure 7** 10
- **Supplementary Figure 8** 11
- **Supplementary Table 2** 12
- **Supplementary Table 3** 13
- **Supplementary Table 4** 15
- **Supplementary Table 5** 16
- **Supplementary Table 6** 18
- **Supplementary Figure 9** 20
- **Supplementary Figure 10** 22

Supplementary Table 7 23
Supplementary Figure 11 25
Supplementary Figure 12 26
Supplementary Note 2 27
- **Supplementary Figure 13** 28
- **Supplementary Table 8** 29

Supplementary Figure 14 31

Supplementary Figure 1

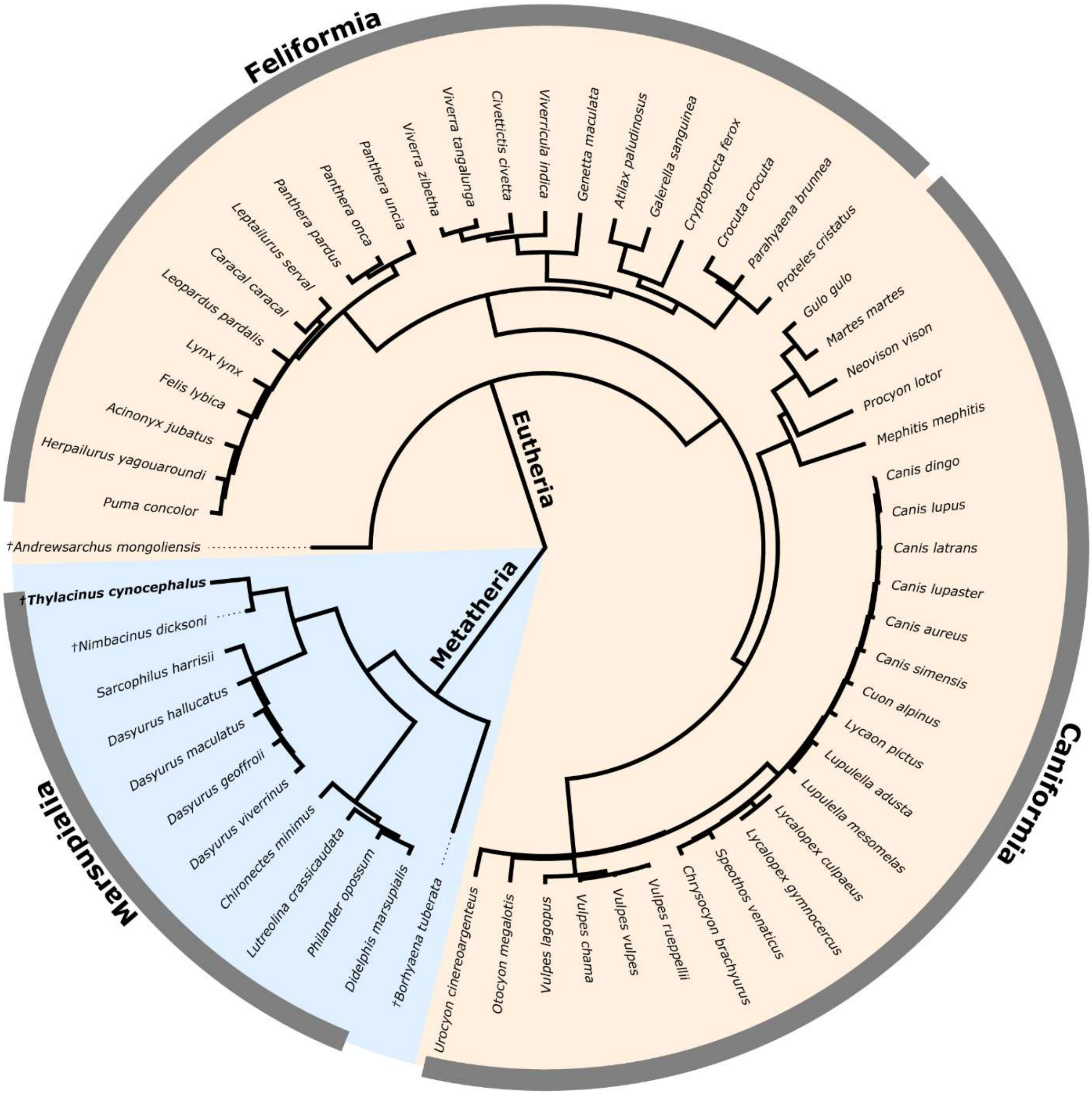

Phylogeny of the species (n=60) covered in our geometric morphometric analysis.

Supplementary Figure 2

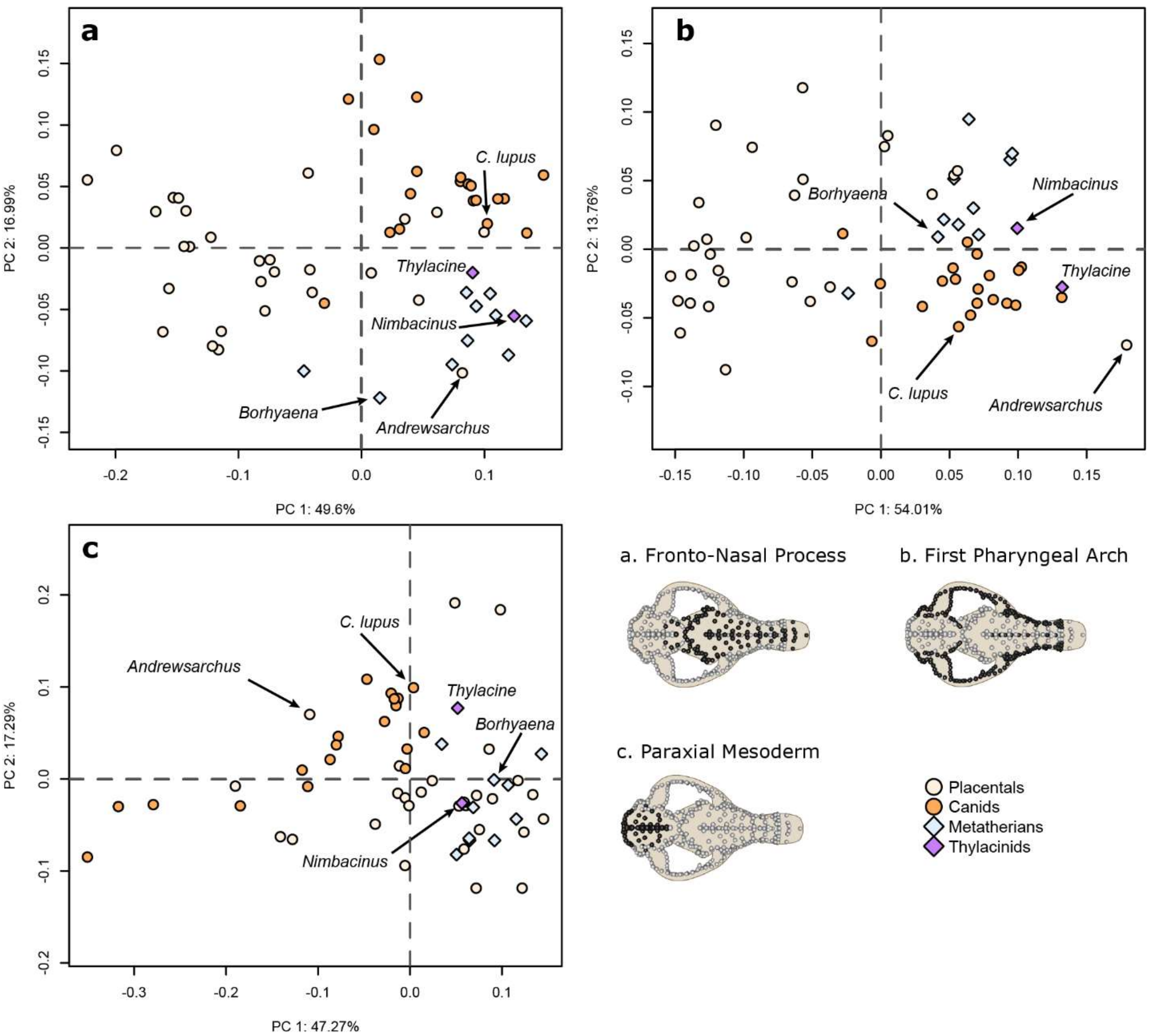

Developmental PCA Principal Components Analysis (PCA) plots of the thylacine cranium in context with carnivorous metatherians and carnivorans, based on developmental partitions (n=60). FNP, Fronto-nasal Process; PA, First Pharyngeal Arch; MES, Paraxial Mesoderm

Supplementary Figure 3

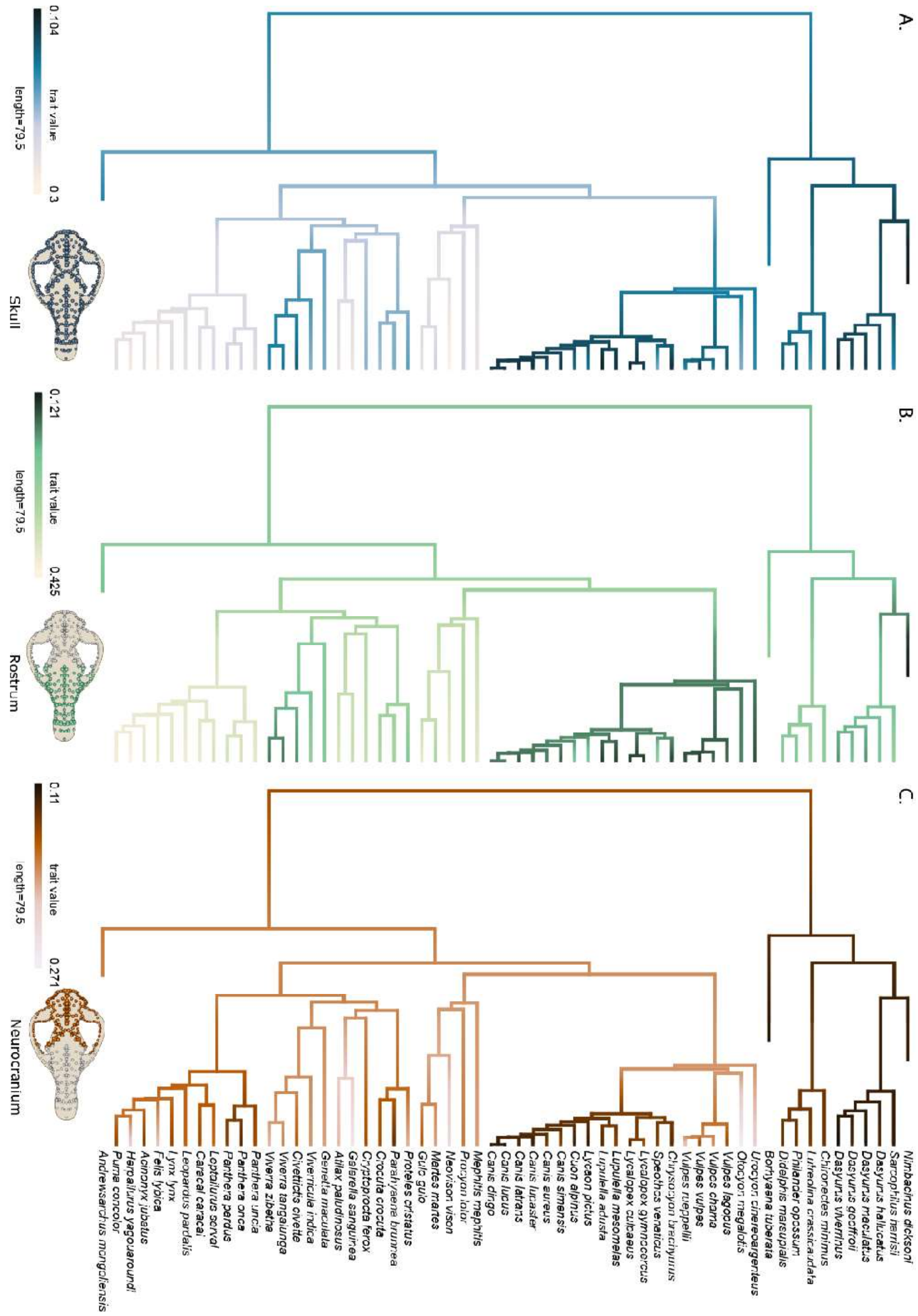

Functional partition Procrustes distances. Procrustes distances of each species from the thylacine for each functional (rostrum *vs* neurocranium) partition, mapped on to the phylogeny used in this study (n=60). These distances are the same as displayed in Fig. 2a of the main manuscript.

Supplementary Figure 4

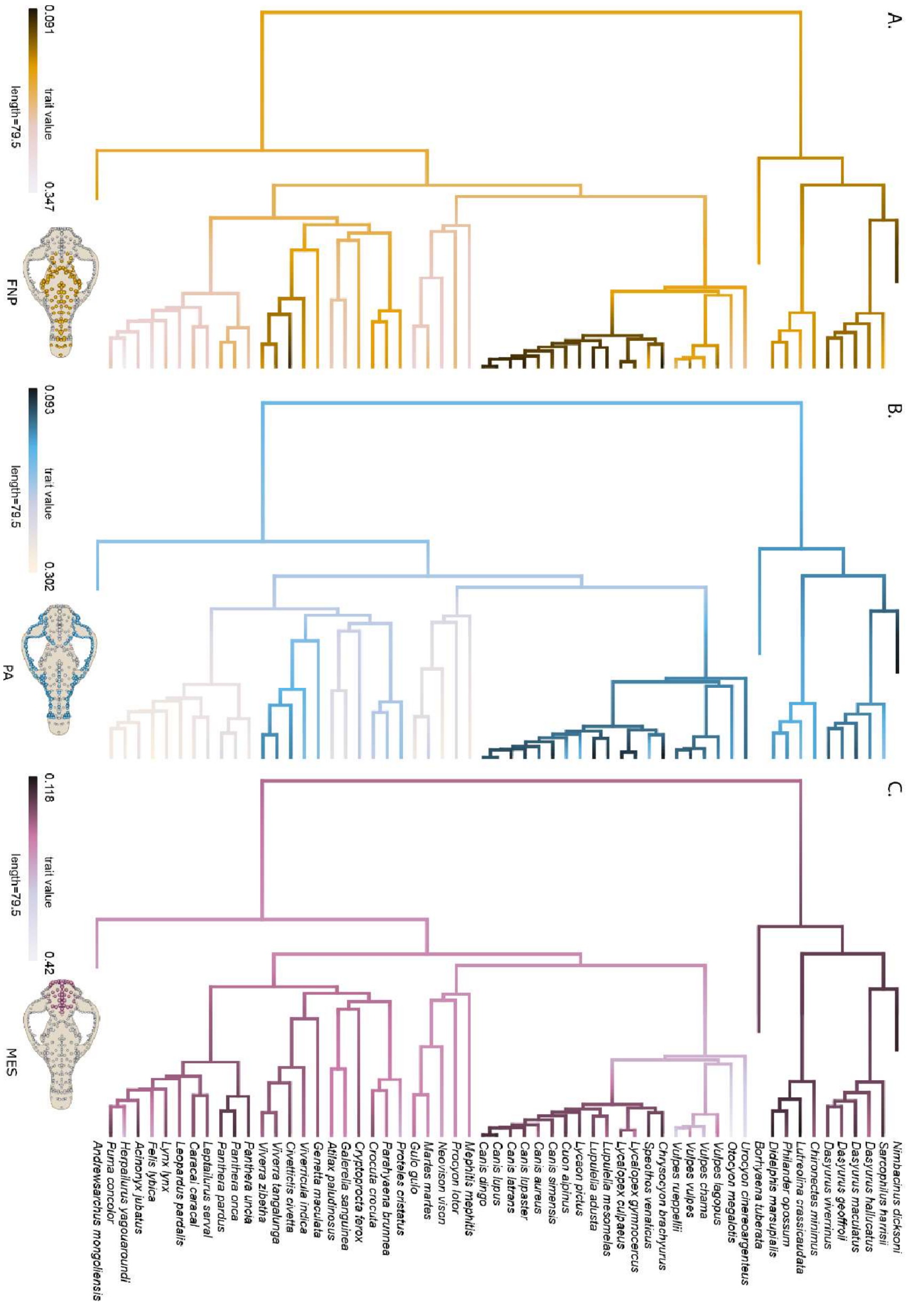

Developmental partition Procrustes distances. Procrustes distances of each species from the thylacine for each developmental (FNP, PA, and MES) partition, mapped on to the phylogeny used in this study (n=60). These distances are the same as displayed in Fig. 2b of the main manuscript.

Supplementary Figure 5

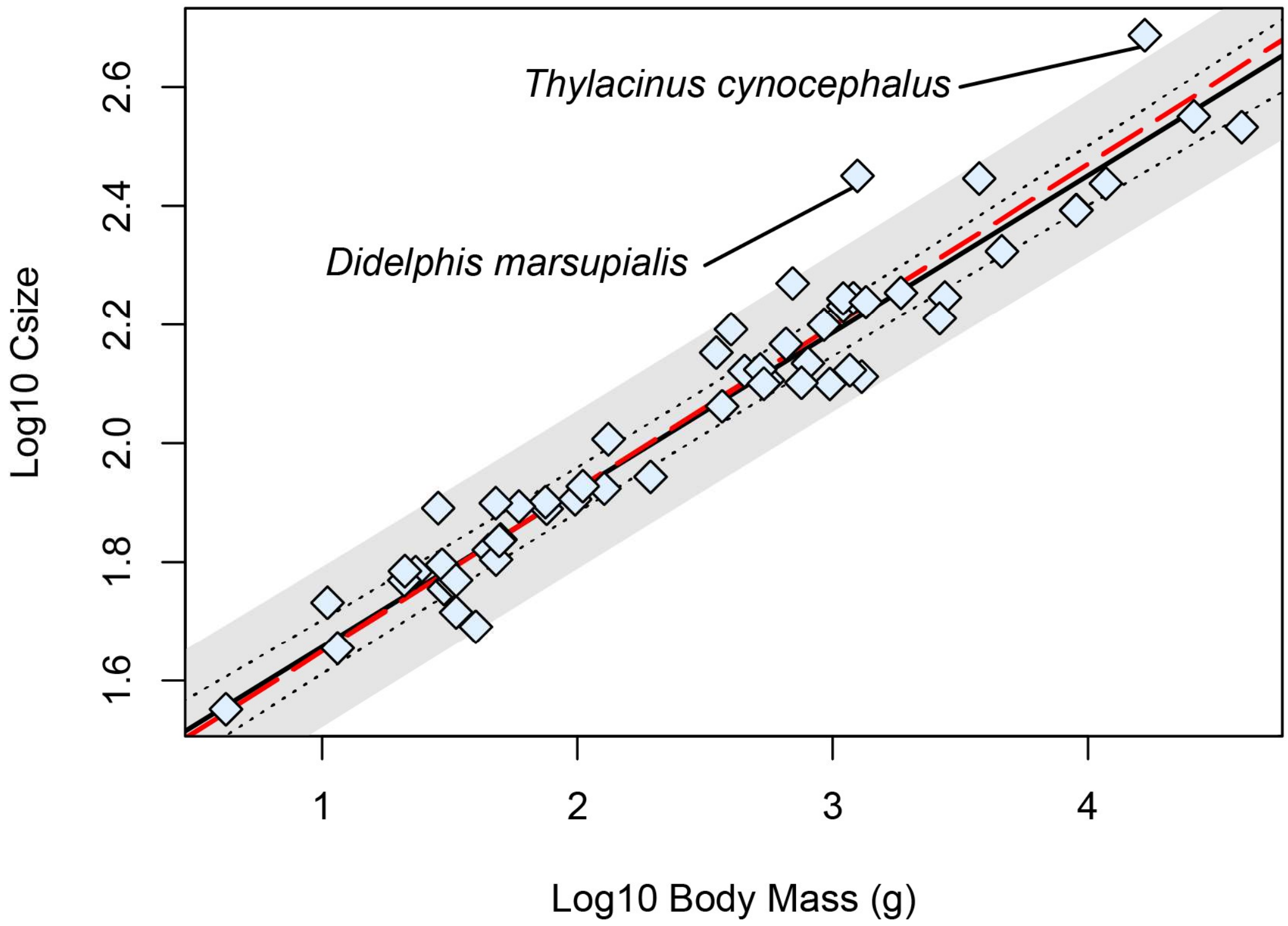

Scaling of marsupial cranial centroid sizes. Phylogenetic Generalised Least Squares (PGLS) regression of log10-transformed centroid size against body mass in a sample of marsupial mammals based on a dataset from Weisbecker et al. (2023; n=63). Solid black regression line, 95% confidence intervals (black dotted lines), and 95% prediction interval (grey area) calculated from PGLS regression with the thylacine removed. Red dashed PGLS regression line is from the full data set, showing the effect the thylacine has on the interpretation of the data.

Supplementary Table 1: Procrustes distances between all species and the thylacine for each partition.

FNP, Fronto-nasal Process; PA, First Pharyngeal Arch; MES, Paraxial Mesoderm. n=59. Note that the distances are from separately Procrustes-aligned datasets for each landmark configuration, so that distances are comparable between but not within species; note also that difference in distance might not be statistically significant and are for orientation only.

Species	Cranium	Rostrum	Neurocranium	FNP	PA	Mes
Acinonyx jubatus	0.2603	0.3916	0.1443	0.2653	0.2671	0.1523
Andrewsarchus mongoliensis	0.1748	0.2253	0.1823	0.1774	0.1824	0.2603
Atilax paludinosus	0.2673	0.3763	0.2087	0.2577	0.2618	0.2184
Borhyaena tuberata	0.1659	0.2705	0.1158	0.1792	0.1573	0.1692
Canis aureus	0.1401	0.1834	0.1432	0.1271	0.1352	0.2001
Canis dingo	0.1279	0.174	0.1203	0.1067	0.1192	0.1499
Canis latrans	0.1191	0.1591	0.1293	0.102	0.1051	0.1633
Canis lupaster	0.1215	0.1751	0.1194	0.0989	0.1135	0.1632
Canis lupus	0.1238	0.1871	0.1105	0.0955	0.1227	0.136
Canis simensis	0.1269	0.1601	0.1458	0.1251	0.1023	0.166
Caracal caracal	0.2616	0.3998	0.155	0.2441	0.2812	0.1801
Chironectes minimus	0.1548	0.2714	0.1381	0.1655	0.159	0.1897
Chrysocyon brachyurus	0.1187	0.1457	0.1305	0.091	0.0939	0.144
Civettictis civetta	0.1492	0.194	0.1652	0.1048	0.142	0.1651
Crocuta crocuta	0.2186	0.3185	0.1568	0.1748	0.2253	0.1776
Cryptoprocta ferox	0.2098	0.3426	0.1546	0.1989	0.2205	0.1782
Cuon alpinus	0.155	0.2297	0.1191	0.123	0.1622	0.1571
Dasyurus geoffroii	0.1363	0.2208	0.1239	0.1437	0.1367	0.178
Dasyurus hallucatus	0.1442	0.232	0.1284	0.1524	0.1464	0.2048
Dasyurus maculatus	0.1273	0.1931	0.1103	0.136	0.1217	0.1481
Dasyurus viverrinus	0.1173	0.1954	0.1095	0.1284	0.1219	0.1708
Didelphis marsupialis	0.1477	0.2469	0.1358	0.1585	0.1511	0.1297
Felis lybica	0.2834	0.4059	0.2147	0.3136	0.3019	0.2605
Galerella sanguinea	0.2754	0.3475	0.256	0.2567	0.2626	0.2341
Genetta maculata	0.2048	0.2527	0.1974	0.1988	0.1878	0.1861
Gulo gulo	0.2683	0.3976	0.1626	0.2834	0.2831	0.2119
Herpailurus yagouaroundi	0.2854	0.4252	0.2225	0.3468	0.2886	0.2831
Leopardus pardalis	0.2663	0.4073	0.1677	0.2851	0.2799	0.197
Leptailurus serval	0.2515	0.3715	0.1678	0.2629	0.2623	0.1804
Lupulella adusta	0.1115	0.1443	0.1327	0.1017	0.0994	0.1643
Lupulella mesomelas	0.1257	0.1478	0.1489	0.108	0.1118	0.198
Lutreolina crassicaudata	0.1873	0.3021	0.1441	0.2029	0.1835	0.1178
Lycalopex culpaeus	0.1134	0.145	0.1271	0.11	0.1001	0.1555

Lycalopex gymnocercus	0.1306	0.1419	0.1591	0.1256	0.113	0.2256
Lycaon pictus	0.1606	0.2502	0.1181	0.1097	0.1759	0.1509
Lynx lynx	0.2563	0.389	0.1521	0.2473	0.2781	0.1744
Martes martes	0.2355	0.3006	0.1969	0.2269	0.2276	0.2406
Mephitis mephitis	0.2745	0.3885	0.1967	0.2842	0.2841	0.2418
Neovison vison	0.2998	0.397	0.2395	0.2774	0.2935	0.2678
Nimbacinus dicksoni	0.1041	0.1205	0.119	0.1109	0.0934	0.1335
Otocyon megalotis	0.2074	0.1836	0.2705	0.2402	0.1545	0.42
Panthera onca	0.2463	0.3714	0.1298	0.2151	0.2653	0.1464
Panthera pardus	0.2361	0.3647	0.144	0.2124	0.254	0.1631
Panthera uncia	0.2596	0.3921	0.154	0.2539	0.2849	0.1577
Parahyaena brunnea	0.2049	0.2973	0.1343	0.1701	0.2115	0.2097
Philander opossum	0.1503	0.2482	0.1385	0.1627	0.1486	0.1705
Procyon lotor	0.2442	0.3202	0.1793	0.2311	0.2564	0.2388
Proteles cristatus	0.1939	0.2473	0.2054	0.1889	0.1987	0.2842
Puma concolor	0.2629	0.41	0.1511	0.2563	0.2865	0.1581
Sarcophilus harrisii	0.1733	0.285	0.1179	0.1847	0.1949	0.1447
Speothos venaticus	0.2039	0.277	0.1444	0.1844	0.1975	0.1476
Urocyon cinereoargenteus	0.1783	0.1546	0.2244	0.242	0.1301	0.3727
Viverra tangalunga	0.1632	0.1771	0.1862	0.1393	0.1396	0.1782
Viverra zibetha	0.1755	0.1795	0.2089	0.1687	0.1446	0.1971
Viverricula indica	0.2104	0.2826	0.1941	0.1701	0.1893	0.2024
Vulpes chama	0.1623	0.1686	0.1895	0.1915	0.1384	0.3048
Vulpes lagopus	0.1453	0.1681	0.153	0.1422	0.1437	0.2126
Vulpes rueppellii	0.1722	0.1553	0.2183	0.2283	0.1313	0.3498
Vulpes vulpes	0.1249	0.1349	0.1487	0.137	0.1095	0.2218

Supplementary Note 1: Summary of assessment of allometry in the landmark coordinates

We chose to run our shape analyses without correcting for allometry, because we expected allometric effects on cranial shape to differ among the widely divergent clades that our dataset was comprised of (e.g. metatherians *vs.* eutherians; felids *vs.* canids), limiting the biological meaning of any emerging allometric patterns. We additionally confirmed that allometry was not a major driver of cranial shape by assessing the amount of cranial shape variation associated with log-transformed cranial centroid size. We did this using Phylogenetic Generalised Least Squares (PGLS), using geomorph's *procD.pgls* function for cranial shape and caper's *pgls* function for Principal Components. Analysis of the full dataset showed significant allometry ($R^2 = 0.33$, p=0.001). However, the inclusion of the extreme-sized *Andrewsarchus mongolensis*, which is a very high-leverage outlier in the pgls analysis, prompted us to do the allometry analyses without this species (Supplementary Fig. 6).

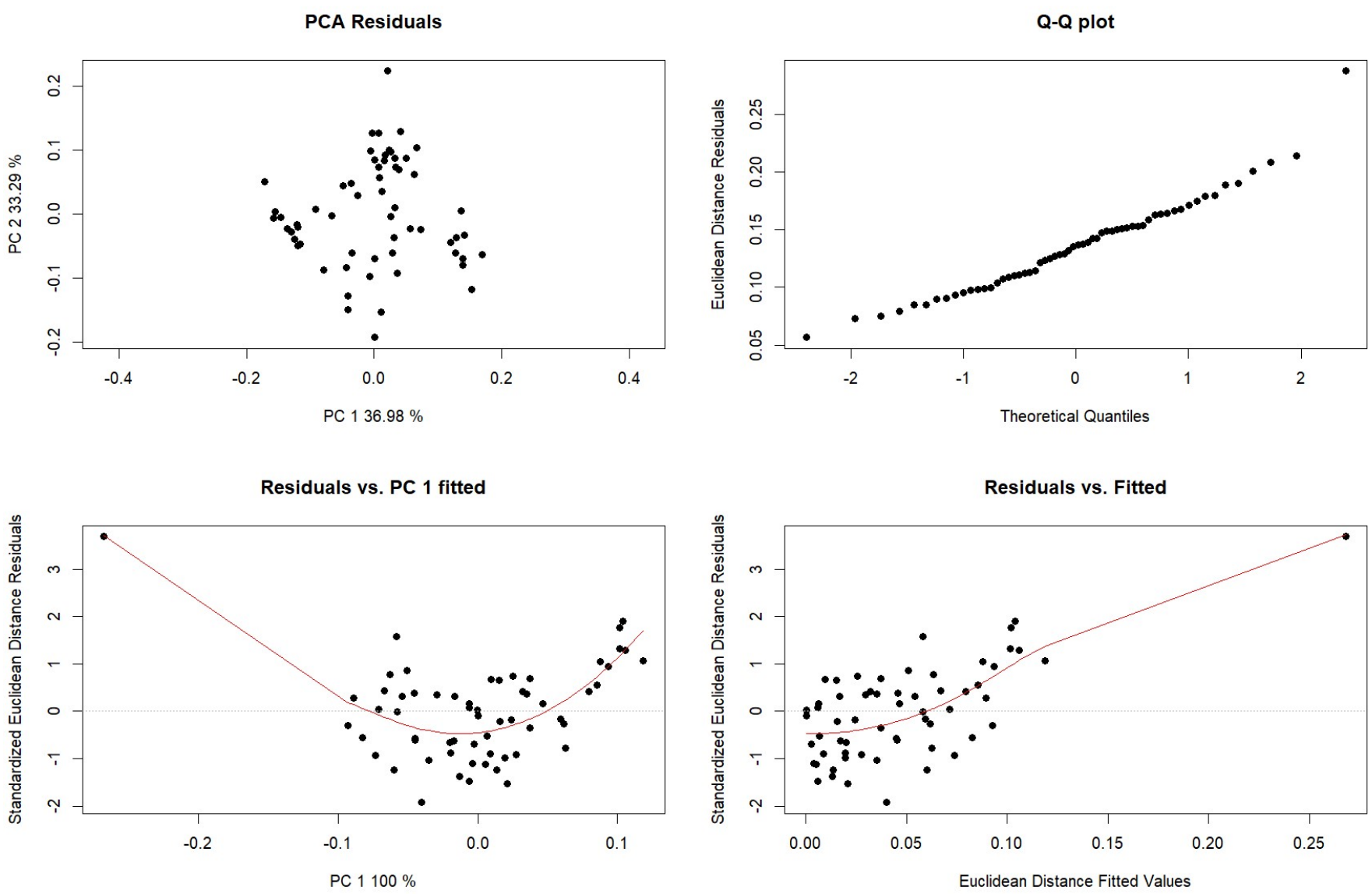

Supplementary Figure 6: Fit of allometry model with *A. mongoliensis.* Model fit output from a Procrustes Generalised Least Squares model of cranial shape *vs* log(cranial centroid size) with *A. mongoliensis* included (n=60).

Excluding *A. mongoliensis* from the analysis produced a significant association (R^2 = 0.33, *p*= 0.000) between cranial shape and log(cranial centroid size), with better fit of the model (Supplementary Fig. 7).

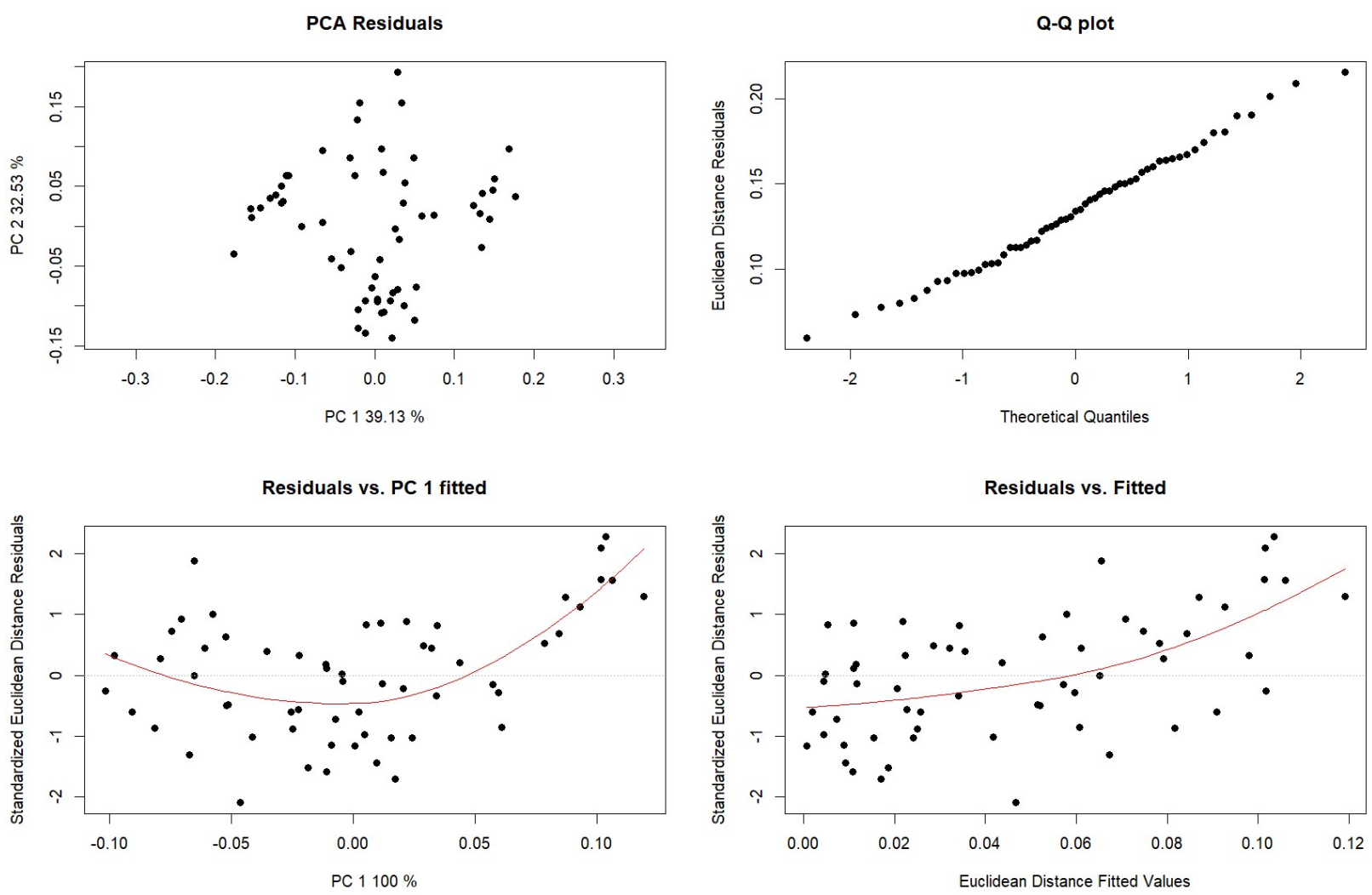

Supplementary Figure 7: **Fit of allometry model without *A. mongoliensis*** Model fit output from a Procrustes Generalised Least Squares model of cranial shape *vs* log(cranial centroid size) with *A. mongoliensis* excluded (n=59).

To ensure that the removal of *Andrewsarchus* from our analyses did not impact on the shape variation represented in PC1 and PC2, we also replicated Figure 1 of the main manuscript with *Andrewsarchus* excluded. This revealed very few differences, with exception of a sign reversal of PC2 of all PCs (Supplementary Fig. 8).

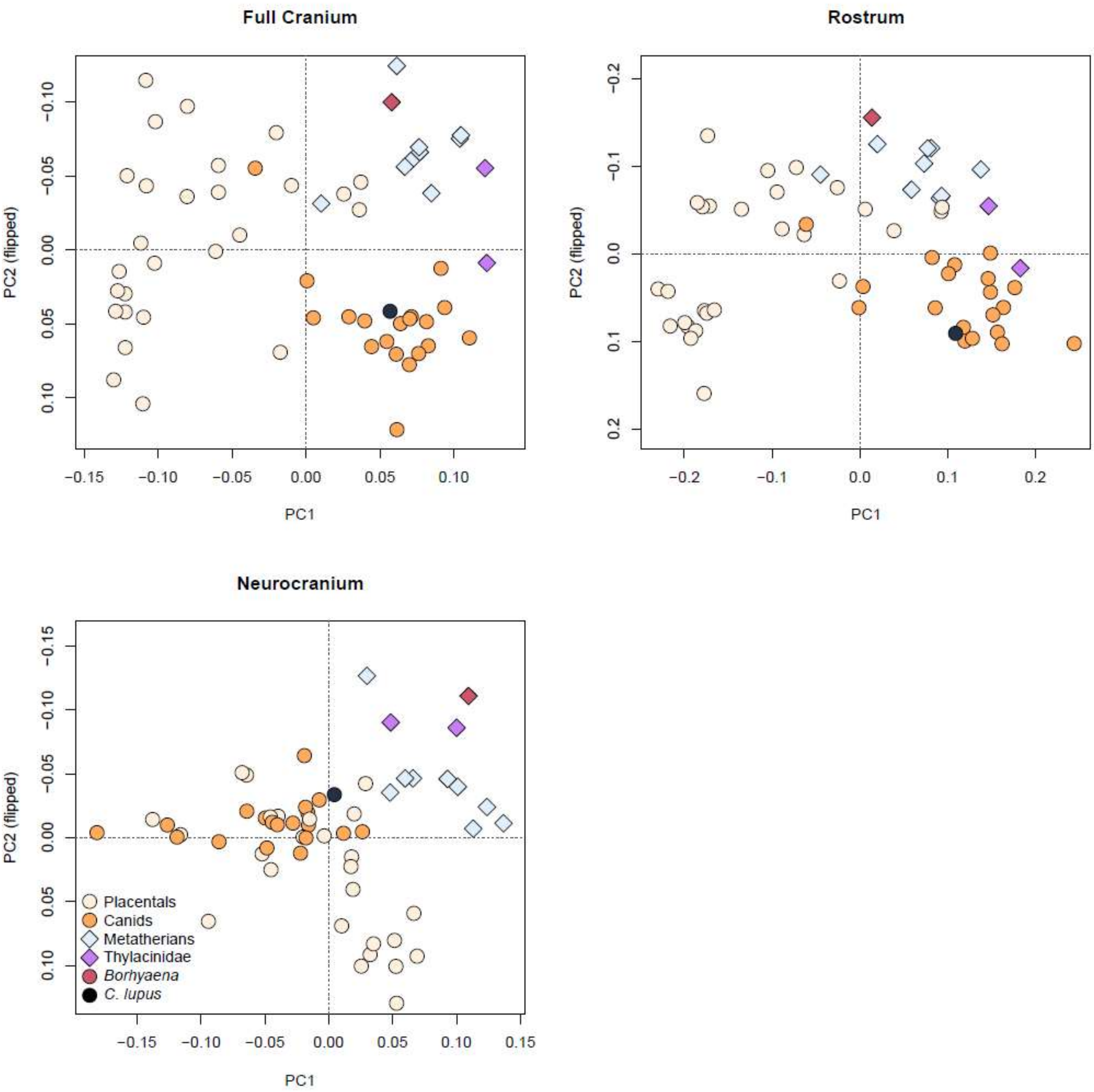

Supplementary Figure 8: **PCA of the whole sample without *A. mongoliensis*.** Replication of Figure 1 of the main manuscript, with *A. mongoliensis* excluded (n=59).

Computing the influence of size while excluding *Andrewsarchus* revealed significant allometry for all partitions, with size explaining most of the neurocranial partition and least of the rostral partition (Supplementary Table 2).

Supplementary Table 2: PGLS of allometry by partition. Output of a PGLS analysis of the landmark configurations of each partition against their log (cranial centroid size), with *Andrewsarchus mongoliensis* removed from the analysis (n=59). MS, Mean Squares; F value, effect size of the model; R^2, r squared value; p, probability of significant association (significance threshold bolded at p = <0.05). Statistics and significances derived through randomized residual permutation procedure (RRPP), a permutation F-test (effectively one-sided).

	MS	R^2	F value	*p*
Cranium	0.026	0.33	28.623	**0.001**
Rostrum	0.033	0.28	22.7	**0.001**
Neurocranium	0.033	0.43	42.776	**0.001**
Frontonasal Process	0.034	0.32	26.811	**0.001**
First Pharyngeal Arch	0.022	0.36	32.685	**0.001**
Paraxial Mesenchyme	0.049	0.36	32.34	**0.001**

Assessing the influence of size on individual Principal Components shows that nearly all PCs in the datsaset are allometric, with notable exception of PCs 1 and 2 of the rostral morphospace (Supplementary Table 3). This suggests that overall allometry in each partition is a composite of multiple allometric sub-patterns, each partially explaining diverse Principal Components.

Supplementary Table 3: PGLS of allometry by Principal Components Output of a PGLS analysis of the first ten Principal Components (PC) for each partition against its log (cranial centroid size) with *Andrewsarchus mongoliensis* removed from the analysis (n=59). Std. Error, Standard error; t value, effect size of the model; R^2, r squared value; p, probability of significant association (significance threshold bolded, at *p* = <0.05, using a two-sided phylogenetic t-test).

	Std.Error	t.value	R^2	p
**Cranium**				
PC1	0.011	1.162	0.02	0.250
PC2	0.013	-3.913	0.21	**0.000**
PC3	0.007	10.391	0.65	**0.000**
PC4	0.006	8.572	0.56	**0.000**
PC5	0.005	-6.139	0.4	**0.000**
PC6	0.005	5.311	0.33	**0.000**
PC7	0.005	-1.301	0.03	0.198
PC8	0.005	-0.55	0.01	0.585
PC9	0.004	6.948	0.46	**0.000**
PC10	0.005	-5.746	0.37	**0.000**
**Rostrum**				
PC1	0.022	-0.754	0.01	0.454
PC2	0.012	1.161	0.02	0.250
PC3	0.006	-9.241	0.6	**0.000**
PC4	0.008	-12.796	0.74	**0.000**
PC5	0.007	-7.455	0.49	**0.000**
PC6	0.005	1.915	0.06	0.061
PC7	0.006	2.446	0.1	**0.018**
PC8	0.005	-1.412	0.03	0.163
PC9	0.004	-2.974	0.13	**0.004**
PC10	0.006	-5.702	0.36	**0.000**
**Neurocranium**				
PC1	0.012	6.967	0.46	**0.000**
PC2	0.007	10.079	0.64	**0.000**
PC3	0.008	2.296	0.08	**0.025**
PC4	0.005	8.511	0.56	**0.000**
PC5	0.006	-0.704	0.01	0.484
PC6	0.005	6.845	0.45	**0.000**
PC7	0.005	-9.671	0.62	**0.000**
PC8	0.005	-5.439	0.34	**0.000**
PC9	0.004	4.883	0.29	**0.000**
PC10	0.004	4.809	0.29	**0.000**
**Frontonasal Process**				
PC1	0.018	5.303	0.33	**0.000**
PC2	0.018	-6.246	0.41	**0.000**

PC3	0.012	5.096	0.31	**0.000**
PC4	0.006	3.066	0.14	**0.003**
PC5	0.011	-4.524	0.26	**0.000**
PC6	0.008	-2.195	0.08	**0.032**
PC7	0.006	-4.846	0.29	**0.000**
PC8	0.008	-5.419	0.34	**0.000**
PC9	0.006	-5.048	0.31	**0.000**
PC10	0.005	-4.079	0.23	**0.000**
**First Pharyngeal Arch**				
PC1	0.013	-1.976	0.06	0.053
PC2	0.006	6.571	0.43	**0.000**
PC3	0.005	-14.008	0.77	**0.000**
PC4	0.004	-0.908	0.01	0.368
PC5	0.004	11.739	0.71	**0.000**
PC6	0.005	-3.02	0.14	0.004
PC7	0.005	3.243	0.16	**0.002**
PC8	0.003	0.516	0	0.608
PC9	0.004	-0.626	0.01	0.534
PC10	0.003	2.268	0.08	0.027
**Paraxial Mesoderm**				
PC1	0.022	5.715	0.36	**0.000**
PC2	0.009	-10.963	0.68	**0.000**
PC3	0.008	3.022	0.14	**0.004**
PC4	0.009	-5.686	0.36	**0.000**
PC5	0.007	-2.635	0.11	**0.011**
PC6	0.012	-1.957	0.06	0.055
PC7	0.009	-6.145	0.4	**0.000**
PC8	0.004	4.761	0.28	**0.000**
PC9	0.004	7.606	0.5	**0.000**
PC10	0.005	-0.46	0	0.647

Importantly, a breakdown of allometries within the main clades in the dataset (Caniformia, Feliformia, and Metatheria) shows that, without phylogenetic correction, allometry within the whole cranium emerges from diverse amounts of variation explained by size, depending on the groups and partitions (Supplementary Table 4). For example, size explains nearly 40% and 47% of the variation in the caniform rostrum and Frontonasal Process (FNP), respectively, but only 21%/14% of variation in the feliform rostrum/FNP (overall allometry: 28%/32%, Supplementary Table 4). By contrast, allometric variation is

comparatively uniform (21-29%) across all partitions in Metatherians. Using phylogenetically corrected analyses results in lack of significance for most configurations tested in metatherians and feliforms, whereas robust allometry was found in caniforms (Supplementary Table 4).

Supplementary Table 4: PGLS and linear models of allometry by partition and clade. Output of a PGLS and linear model analysis of the landmark configurations of each partition against their log (cranial centroid size), by major taxonomic division within the dataset and with *Andrewsarchus mongoliensis* removed from the analysis. SS, Sum of Squares; F, effect size of the model; R^2, r squared value; p, probability of significant association (significance threshold bolded at p = <0.05). Feliformia n=22; Caniformia, n=25; Metatheria, n=12. Statistics and significances derived through randomized residual permutation procedure (RRPP), a one-sided permutation F-test.

	Linear model				PGLS			
	SS	F	RSq	p	SS	F	RSq	p
Cranium								
Caniformia	0.087	9.917	0.3	**0.001**	0.028	15.205	0.4	**0.006**
Feliformia	0.064	6.506	0.25	**0.001**	0.004	1.967	0.09	0.111
Metatheria	0.019	2.832	0.22	**0.006**	0.001	0.647	0.06	0.469
Rostrum								
Caniformia	0.231	14.926	0.39	**0.001**	0.035	12.491	0.35	**0.002**
Feliformia	0.104	5.401	0.21	**0.005**	0.005	2.425	0.11	0.051
Metatheria	0.049	3.321	0.25	**0.011**	0.002	0.976	0.09	0.319
Neurocranium								
Caniformia	0.039	5.33	0.19	**0.002**	0.035	22.012	0.49	**0.002**
Feliformia	0.065	7.941	0.28	**0.001**	0.004	2.076	0.09	0.093
Metatheria	0.021	3.441	0.26	**0.002**	0.001	0.865	0.08	0.346
Frontonasal Process								
Caniformia	0.178	20.341	0.47	**0.001**	0.04	17.124	0.43	**0.002**
Feliformia	0.051	3.259	0.14	**0.021**	0.005	2.382	0.11	0.052
Metatheria	0.034	4.052	0.29	**0.004**	0.002	1.396	0.12	0.18
First Pharyngeal Arch								
Caniformia	0.081	11.44	0.33	**0.001**	0.023	14.167	0.38	**0.009**
Feliformia	0.069	8.033	0.29	**0.001**	0.003	1.897	0.09	0.121
Metatheria	0.018	2.612	0.21	**0.031**	0.001	0.507	0.05	0.583
Paraxial Mesenchyme								
Caniformia	0.124	6.495	0.22	**0.004**	0.043	14.899	0.39	**0.001**
Feliformia	0.06	3.644	0.15	**0.01**	0.01	4.275	0.18	**0.002**
Metatheria	0.037	4.155	0.29	**0.001**	0.003	2.012	0.17	0.071

Modelling size and clade affinity in a non-phylogenetically corrected linear model revealed significant interaction terms between clade and size for all but the posterior (MES/Neurocranial) partitions, meaning that scaling slopes differ between clades (Supplementary Table 5). Notably, however, the clade factor explained around three times as much variation as size alone or the size:clade interaction term in all analyses, which suggests that clade-specific differences are not confounded by any allometry-related patterns and challenging the hypothesis that the variation within the whole sample could relate to allometry.

Supplementary Table 5: **Linear Procrustes regression summary for each partition against its log (centroid size).** *A. mongoliensis* is removed . SS, sum of squares; t value, R^2, R squared value; F, F statistic; Z, Z statistic; p, probability of significant association (significance threshold bolded, at $p = <0.05$). Feliformia n=22; Caniformia, n=25; Metatheria, n=12. Statistics and significances derived through randomized residual permutation procedure (RRPP), a permutation F-test (effectively one-sided).

	SS	R^2	F	Z	p
**Cranium**					
log Csize	0.107	0.11	12.252	3.983	**0.001**
Clade	0.324	0.34	18.549	5.183	**0.001**
Clade:Csize	0.07	0.07	4.016	3.047	**0.002**
**Rostrum**					
log Csize	0.208	0.11	12.425	3.393	**0.001**
Clade	0.586	0.31	17.508	4.187	**0.001**
Clade:Csize	0.22	0.12	6.568	3.37	**0.001**
**Neurocranium**					
log Csize	0.083	0.11	11.216	4.115	**0.001**
Clade	0.236	0.32	15.944	6.345	**0.001**
Clade:Csize	0.025	0.03	1.666	1.32	0.106
**FNP**					
log Csize	0.111	0.09	9.833	3.569	**0.001**
Clade	0.364	0.3	16.128	4.305	**0.001**
Clade:Csize	0.151	0.12	6.701	3.614	**0.001**
**PA**					
log Csize	0.085	0.1	11.185	3.435	**0.001**
Clade	0.284	0.33	18.637	4.205	**0.001**
Clade:Csize	0.081	0.09	5.314	2.958	**0.001**
**MES**					
log Csize	0.136	0.1	8.444	3.335	**0.001**
Clade	0.342	0.25	10.593	4.45	**0.001**
Clade:Csize	0.055	0.04	1.712	1.226	0.126

To also understand which clades were significantly different in allometric slopes or mean, we computed pairwise comparisons between clades using the geomorph pairwise function (Supplementary Table 6). For differences in slopes, we assessed slope magnitude (i.e. how change per unit size), and slope angles (angle between vectors of shape change with size) relative to each other. The mostly significant interaction effects – i.e., slope differences - mean that comparisons of means are potentially confounded by variation in slope. However, because the effect of clade by itself explains 3-6 times as much of the variation as either the size effect or the clade:size interaction, comparisons between clade means are still likely informative and therefore also presented. Differences in slope magnitude and angle reveal similar levels of significance and were mostly between caniforms and the other two clades (Supplementary Table 6), meaning that similarities between canids and thylacines are not attributable to shape changes along a common allometric line between metatherians and canids. Significant differences in intercept between all clades show that all clades have fundamentally different shapes alongside their mostly divergent slopes, further highlighting the lack of commonality in allometric scaling between clades.

Supplementary Table 6: Pairwise comparisons between clades for Procrustes regressions presented in Table 4. Test is for either slope (significant interaction term) or mean (significant clade term) differences. d, distances between vector means or slopes; UCL 95%, 95% confidence intervals; Z, Z statistics; p, probability of significant differences;, p adj, p value adjusted for multiple comparisons using the Benjamini-Hochberg procedure; r, vector correlation; angle, angle between vectors. Feliformia n=22; Caniformia, n=25; Metatheria, n=12. Statistics and significances derived through randomized residual permutation procedure (RRPP), a permutation F-test (one-sided).

	**Slope magnitudes**					**Slope angles**						**Slope means**				
	d	UCL 95%	Z	*p*	*p adj*	r	angle	UCL(95%)	Z	*p*	*p adj*	d	UCL 95%	Z	*p*	*p adj*
Cranium																
Caniformia:Feliformia	0.197	0.129	2.908	**0.001**	**0.003**	0.341	70.1	54.391	2.468	**0.002**	**0.006**	0.111	0.056	3.4	**0.001**	**0.001**
Caniformia:Metatheria	0.178	0.139	2.36	**0.008**	**0.016**	0.366	68.5	59.717	2.107	**0.01**	**0.02**	0.131	0.072	3.272	**0.001**	**0.001**
Feliformia:Metatheria	0.12	0.133	1.372	0.089	0.089	0.595	53.5	57.597	1.482	0.075	0.075	0.186	0.073	4.281	**0.001**	**0.001**
Rostrum																
Caniformia:Feliformia	0.301	0.168	3.062	**0.001**	**0.003**	0.073	85.8	62.279	2.519	**0.003**	**0.009**	0.184	0.081	3.253	**0.001**	**0.002**
Caniformia:Metatheria	0.286	0.195	2.625	**0.003**	**0.006**	0.113	83.5	68.36	2.214	**0.007**	**0.014**	0.11	0.109	1.704	**0.048**	**0.048**
Feliformia:Metatheria	0.176	0.185	1.557	0.068	0.068	0.336	70.4	65.553	1.859	**0.035**	**0.035**	0.23	0.109	3.287	**0.001**	**0.002**
Neurocranium																
Caniformia:Feliformia	0.1	0.122	1.047	0.161	0.161	0.768	39.8	50.341	0.86	0.201	0.201	0.067	0.048	2.773	**0.001**	**0.001**
Caniformia:Metatheria	0.111	0.131	1.188	0.125	0.161	0.641	50.2	54.412	1.422	0.085	0.184	0.153	0.059	4.664	**0.001**	**0.001**
Feliformia:Metatheria	0.104	0.114	1.392	0.084	0.161	0.717	44.2	47.831	1.39	0.092	0.184	0.168	0.064	5.07	**0.001**	**0.001**
FNP																
Caniformia:Feliformia	0.242	0.138	3.207	**0.001**	**0.002**	0.101	84.2	60.454	2.636	**0.001**	**0.003**	0.119	0.068	2.869	**0.001**	**0.002**
Caniformia:Metatheria	0.264	0.153	3.194	**0.001**	**0.002**	-0.126	97.2	66.674	2.82	**0.002**	**0.004**	0.131	0.087	2.703	**0.002**	**0.002**
Feliformia:Metatheria	0.116	0.157	0.897	0.184	0.184	0.551	56.6	67.341	1.173	0.127	0.127	0.195	0.083	3.793	**0.001**	**0.002**
PA																
Caniformia:Feliformia	0.216	0.127	2.762	**0.001**	**0.003**	0.125	82.8	58.064	2.533	**0.002**	**0.006**	0.116	0.055	3.166	**0.001**	**0.002**
Caniformia:Metatheria	0.171	0.136	2.168	**0.014**	**0.028**	0.315	71.6	62.825	2.011	**0.016**	**0.032**	0.102	0.071	2.387	**0.003**	**0.003**
Feliformia:Metatheria	0.118	0.135	1.416	0.094	0.094	0.607	52.6	61.481	1.311	0.113	0.113	0.164	0.069	3.415	**0.001**	**0.002**
MES																
Caniformia:Feliformia	0.148	0.191	0.97	0.175	0.31	0.854	31.3	58.031	-0.057	0.519	0.519	0.103	0.07	2.662	**0.001**	**0.002**
Caniformia:Metatheria	0.223	0.223	1.773	0.051	0.153	0.495	60.3	64.593	1.517	0.078	0.234	0.199	0.094	3.704	**0.001**	**0.002**
Feliformia:Metatheria	0.128	0.196	0.519	0.31	0.31	0.633	50.8	60.176	1.232	0.117	0.234	0.163	0.101	2.927	**0.003**	**0.003**

Plotting the whole samples' allometric regression scores reflecting the shape variation related to allometry (not phylogenetically corrected to show untransformed differences between species) reveals a wide scatter around the common regression line for most partitions, illustrating the low contribution of size variation to our samples' overall variation (Supplementary Fig. 9). Note that the use of the whole samples' (rather than individual) allometry, required for an all-species plot, means that the plots do not reflect the divergent allometries within clades and instead the emergent allometry for the entire sample. Thylacines are generally near the line of best fit, but have consistently lower residuals compared to most canids. In many partitions, the thylacine's shape also appears to follow an overall pattern for Metatherians, even where thylacines are least similar to Metatherians in overall shape (e.g. rostrum, PA; main manuscript Figs. 1&2). However, the line of best fit for the metatherian sample is difficult to approximate because of the divergent regression scores of the largest, and most high-leverage, species: *Borhyaena* (extremely low RegScores) and the thylacine itself (Regression scores near the overall best-fit line). The plots also show little evidence that the thylacine may have "jumped" allometric patterning towards a canid-like condition, since it has low residuals compared to all canids in the overall, rostral, and PA partitions (Supplementary Fig. 9). Similarly, even though wolves are most similar to the thylacine among canids in overall neurocranial shape (main manuscript Fig. 2), this similarity is not predicted based on the overall samples' allometric patterning because thylacine basicrania are both smaller than the wolves' and their overall Regression score is low compared to the wolf and all large canids (Supplementary Fig. 9).

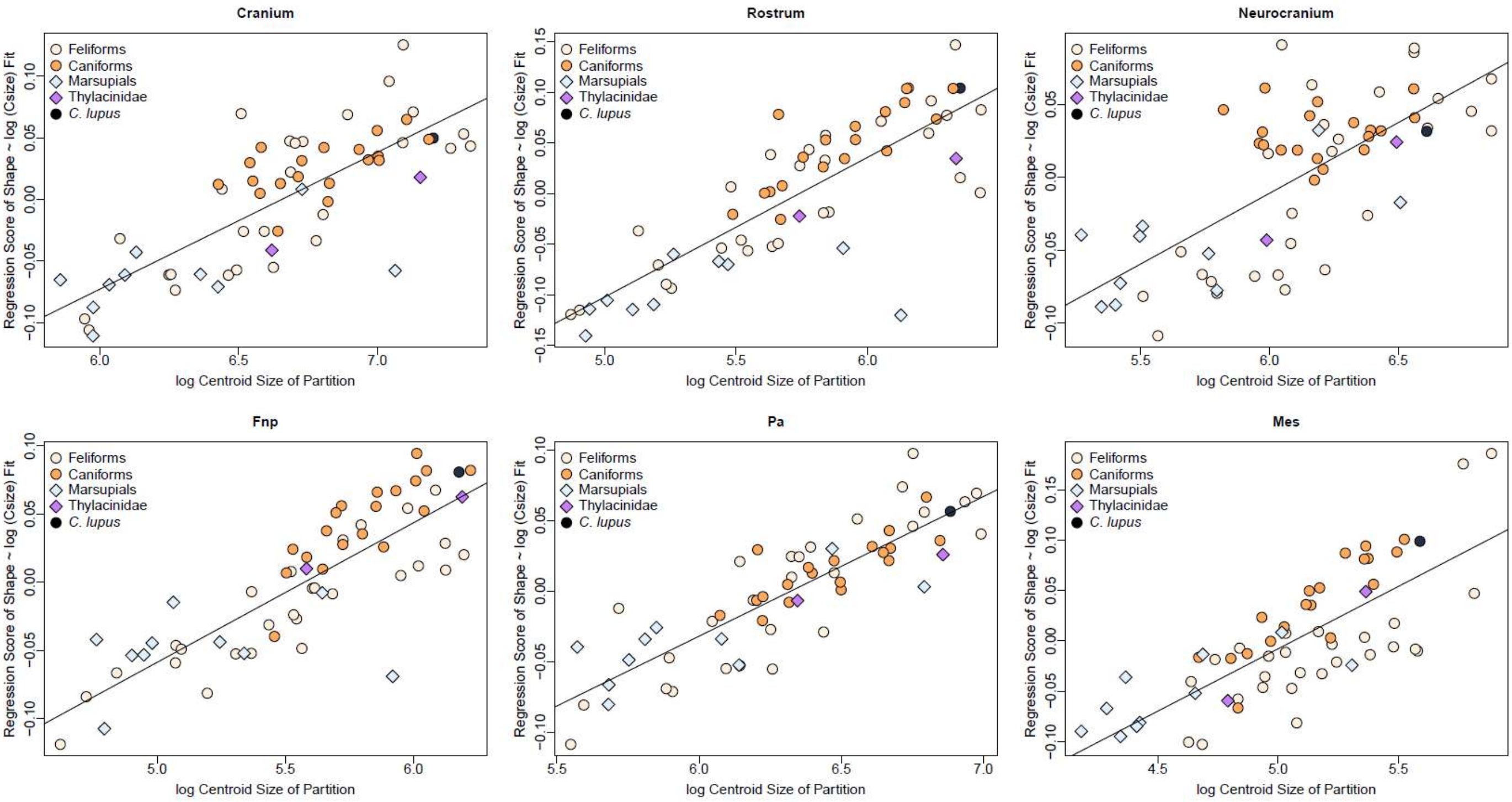

Supplementary Figure 9: Allometry plots by partition. Scatter plot of regression scores (scores that maximise the variation related to allometry) against log centroid size for the whole sample, with the samples' phylogenetically uncorrected common allometric line drawn for orientation. Feliformia n=22; Caniformia, n=25; Metatheria, n=12.

The differences in allometric slopes between the main clades are also visible in shape deformation plots that compare shapes at the smallest vs. largest size for the sample in each partition (Supplementary Fig. 10). In particular, larger canids have longer anterior braincases and narrower anterior zygomatic arches in the rostral and PA partitions, which is not the case in either the feliforms (presumably because of the short-faced large cats in our sample) and metatherians. Unsurprisingly, differences are less extensive in the MES and neurocranial partitions, where scaling slopes between groups are not different; larger individuals of all species tend to have anteriorly displaced occipital areas and narrower cranial vaults (amounting to relatively smaller braincases) and wider zygomatic arches. Metatherian allometry suggests a substantial widening of the postorbital process in the overall, rostral, and FNP regions and a re-arrangement of the snout region whereby the palate landmarks extend anteriorly and the nasal tips retract posteriorly, but this may be an effect of the very disparate shapes of the largest species (short-faced Tasmanian devil and *Borhyaena* vs. long-faced thylacine).

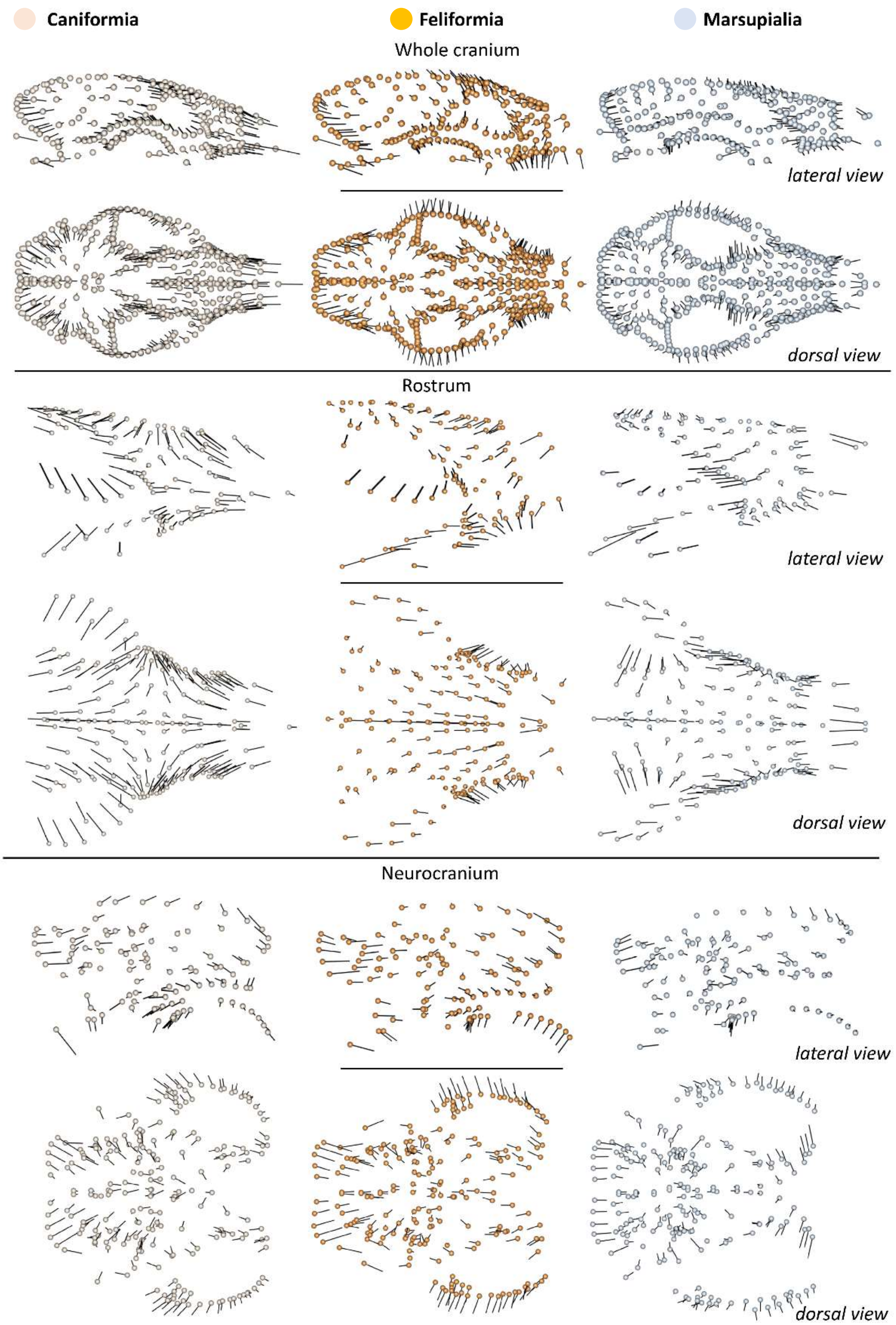

Caniformia
Feliformia
Marsupialia
Whole cranium
lateral view
dorsal view
Rostrum
lateral view
dorsal view
Neurocranium
lateral view
dorsal view

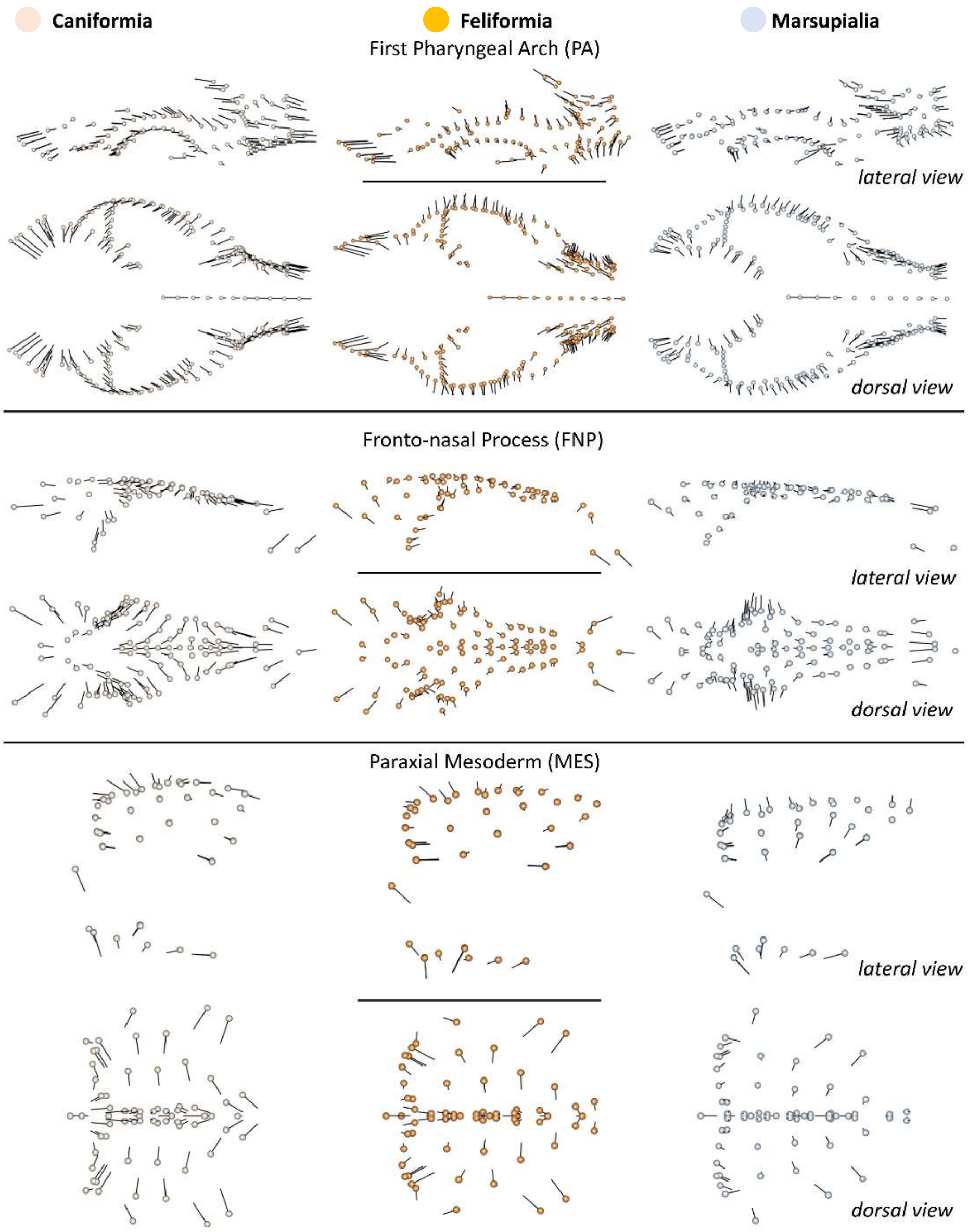

Supplementary Figure 10: Deformation plots of allometric landmark placement differences. Showing the shapes predicted by non-phylogenetically corrected Procrustes linear models for small (balls) *vs.* large (tips of hairs) species within each major clade (left to right: Caniformia, Feliformia, Metatheria) and within each partition. Anterior is right, posterior is left. Feliformia n=22; Caniformia, n=25; Metatheria, n=12.

Supplementary Table 7: Regression coefficients of linear measurement major-axis regressions shown in Fig. 4. These are two-sided tests. Table only lists cases only when significant differences between pairs of species were detected. Elev1/2, intercept of species 1/2; Slope 1/2, slopes of species 1/2; p, p-value (with significance adjusted for multiple comparisons using the Benjamini-Hochberg procedure). Comparisons involving the thylacine are highlighted in blue. *Thylacinus cynocephalus,* n=72; *Canis lupus*, n=27; *Canis dingo,* n=37; *Canis latrans*, n=24; *Lupulella adusta*, n=10, *Lycalopex gymnocercus*, n=9; *Vulpes vulpes*, n=27.

INTERCEPTS

Species 1	Species 2	Elev1	Elev2	TestStat	*p*
**Width across carnassials**					
C. dingo	*C. lupus*	3.08	5.98	10.63	0.001
C. dingo	*L. adusta*	7.50	5.13	4.73	0.030
C. dingo	*T. cynocephalus*	15.30	12.22	56.03	0.000
C. dingo	*V. vulpes*	5.68	2.06	14.21	0.000
C. lupus	*L. adusta*	4.09	0.36	4.42	0.036
C. lupus	*V. vulpes*	5.11	-0.38	12.34	0.000
L. adusta	*V. vulpes*	3.01	1.45	7.94	0.005
L. gymnocercus	*T. cynocephalus*	9.62	13.11	10.19	0.001
**Width across canines**					
C. dingo	*L. gymnocercus*	-9.57	-7.37	4.39	0.036
C. dingo	*T. cynocephalus*	-8.87	-12.85	236.57	0.000
C. dingo	*V. vulpes*	-9.84	-7.73	6.74	0.009
C. latrans	*L. adusta*	-22.99	-19.84	12.85	0.000
C. latrans	*L. gymnocercus*	-17.98	-12.67	19.90	0.000
C. latrans	*V. vulpes*	-17.51	-12.99	29.92	0.000
L. adusta	*L. gymnocercus*	-11.89	-9.57	10.14	0.001
L. adusta	*T. cynocephalus*	-8.87	-13.09	64.70	0.000
L. adusta	*V. vulpes*	-12.05	-10.12	16.59	0.000
L. gymnocercus	*T. cynocephalus*	-7.00	-13.03	118.29	0.000
T. cynocephalus	*V. vulpes*	-13.08	-7.17	158.60	0.000
**Occipital condyle width**					
L. gymnocercus	*V. vulpes*	1.77	-0.10	16.55	0.000

SLOPES

Species 1	Species2	Slope1	Slope2	TestStat	*p*
**Width across carnassials**					
C. dingo	*C. latrans*	0.88	1.39	8.41	0.004
C. latrans	*C. lupus*	1.39	0.96	7.21	0.007
C. latrans	*T. cynocephalus*	1.39	0.77	16.75	0.000
C. latrans	*V. vulpes*	1.39	0.98	5.83	0.016
C. lupus	*T. cynocephalus*	0.96	0.77	6.25	0.012
T. cynocephalus	*V. vulpes*	0.77	0.98	5.48	0.019
**Width across canines**					
C. dingo	*C. latrans*	1.25	1.79	6.29	0.012
C. latrans	*C. lupus*	1.79	1.10	12.06	0.001
C. latrans	*T. cynocephalus*	1.79	1.35	5.70	0.017
C. lupus	*T. cynocephalus*	1.10	1.35	6.43	0.011
**Occipital condyle width**					
C. dingo	*T. cynocephalus*	1.16	0.79	10.46	0.001

INTERCEPTS

**Palate length**					
C. dingo	*C. latrans*	-11.21	-5.68	32.38	0.000
C. dingo	*L. adusta*	-7.58	2.50	28.99	0.000
C. dingo	*L. gymnocercus*	-1.27	3.87	3.89	0.048
C. dingo	*T. cynocephalus*	-11.32	-2.60	202.78	0.000
C. dingo	*V. vulpes*	-11.53	-4.00	14.27	0.000
C. latrans	*C. lupus*	-8.22	-15.77	10.57	0.001
C. latrans	*L. adusta*	-25.36	-17.17	11.00	0.001
C. latrans	*T. cynocephalus*	-7.12	-4.21	9.58	0.002
C. latrans	*V. vulpes*	-22.59	-17.06	5.22	0.022
C. lupus	*L. adusta*	-12.45	-0.40	13.16	0.000
C. lupus	*T. cynocephalus*	-13.07	-3.05	138.33	0.000
C. lupus	*V. vulpes*	-15.63	-5.68	8.64	0.003
L. adusta	*T. cynocephalus*	-1.28	-3.72	3.41	0.065
L. adusta	*V. vulpes*	-11.75	-14.24	3.70	0.054
**Width across zygomatic arches**					
C. latrans	*T. cynocephalus*	-41.54	-44.75	10.54	0.001
C. latrans	*V. vulpes*	-11.33	-7.60	4.64	0.031
L. adusta	*T. cynocephalus*	-37.06	-45.00	20.86	0.000
L. adusta	*V. vulpes*	-3.96	-0.99	8.24	0.004

C. latrans	*T. cynocephalus*	1.47	0.79	11.45	0.001
C. lupus	*T. cynocephalus*	1.12	0.79	8.13	0.004

SLOPES

**Palate length**					
**Width across zygomatic arches**					
C. dingo	*T. cynocephalus*	1.03	1.37	9.36	0.002
C. lupus	*T. cynocephalus*	1.00	1.37	10.72	0.001
L. gymnocercus	*T. cynocephalus*	0.97	1.37	7.71	0.006
T. cynocephalus	*V. vulpes*	1.37	1.03	9.11	0.003

Supplementary Figure 11

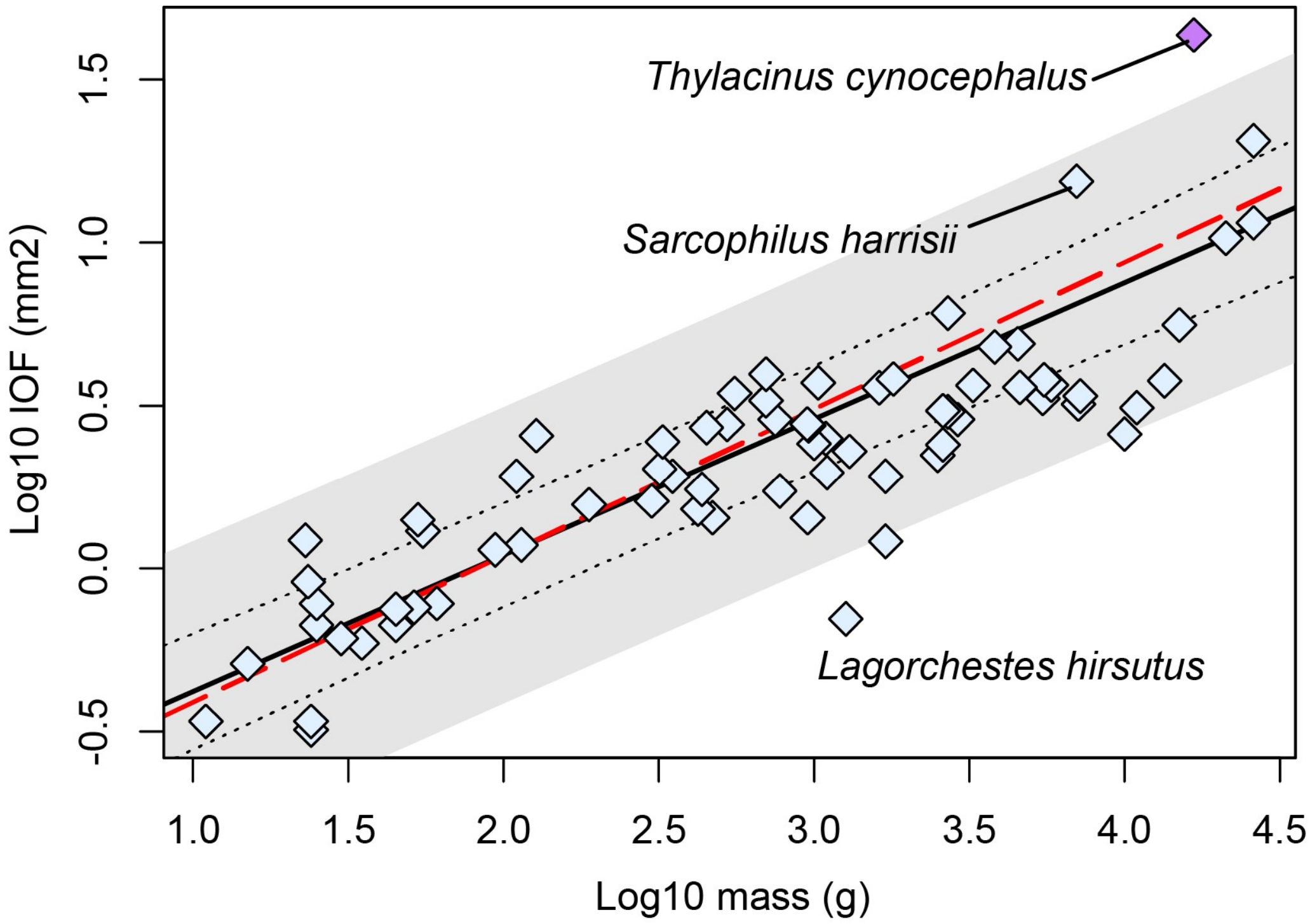

Infraorbital foramen (IOF) size relative to body mass in marsupials only (n=72). Solid black regression line, 95% confidence intervals (black dotted lines), and 95% prediction interval (grey area) calculated from PGLS regression with the thylacine removed in. Red dashed PGLS regression line is from the full data set, showing the effect the thylacine has on the interpretation of the data.

Supplementary Figure 12

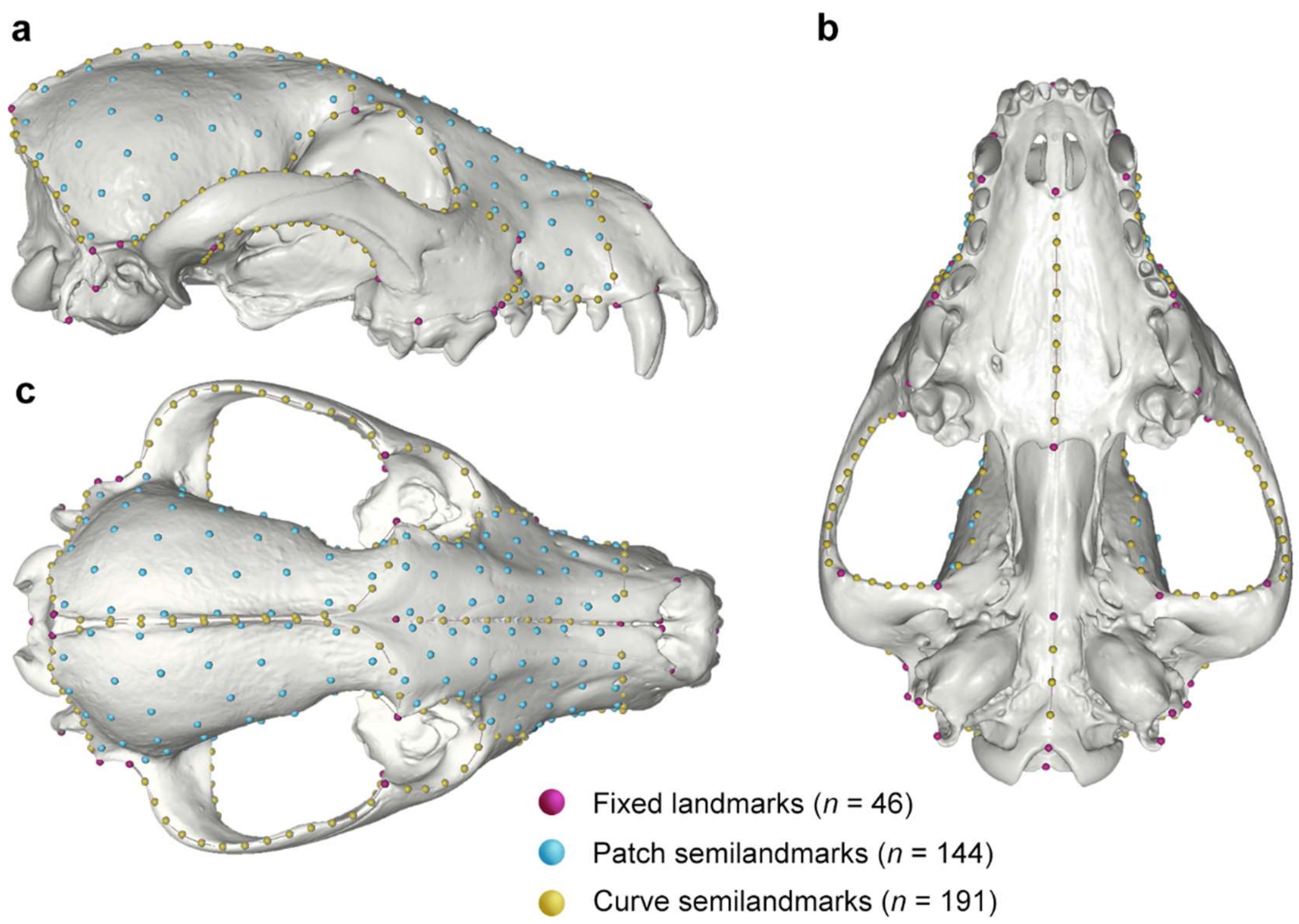

Landmarking protocol used for the geometric morphometric analyses of this study. Colourised spheres reflect fixed, patch, and curve semilandmarks and their numbers.

Supplementary Note 2: **Evaluation of the use of the Wheatsheaf index as an additional means to characterise thylacine-canid convergence.**

Prompted by a reviewer, we evaluated whether further information specifically about shape convergence between thylacines and other carnivorans could expand on our understanding of the thylacine's similarity with other species in the sample. As per reviewer instructions, we consulted the processes explained in Arbuckle (2014)[1] and mostly relied on the implementation of the procedure as published by Grunstra et al. (2024)[2]. However, we did not find application of Wheatsheaf index an appropriate measure, at least not as defined by Arbuckle, for several reasons:

1) The phylogenetic distance matrix approach in Arbuckle 2014[1] (scale to a length of 1, then derive variance-covariance matrix of expected trait variation) creates a matrix where all distances between marsupial vs. placental pairs are zero (i.e. maximum distance, indicating traits are not expected to be affected by co-variation in these species pairs). The flow-on effect when using Arbuckle's phylogenetic correction formula is that all same-clade phenotypic distance pairs are weighted to small values whereas marsupial-placental phenotypic distances remain nearly untransformed (Supplementary Fig. 13), which in our view unduly skews the distances compared to the raw phenotypic distances (plotted against corrected distances in Supplementary Fig. 13; note that the overall pattern of thylacine-placental similarity remains identical and thus confirms our current results). With correction, even the very blunt-faced *Herpailurus yagouaroundi* is closer to the thylacine than any marsupial, even though the thylacine is one of the most gracile-faced species in the sample - so clearly this is not a biologically meaningful operation (Supplementary Fig. 13a)

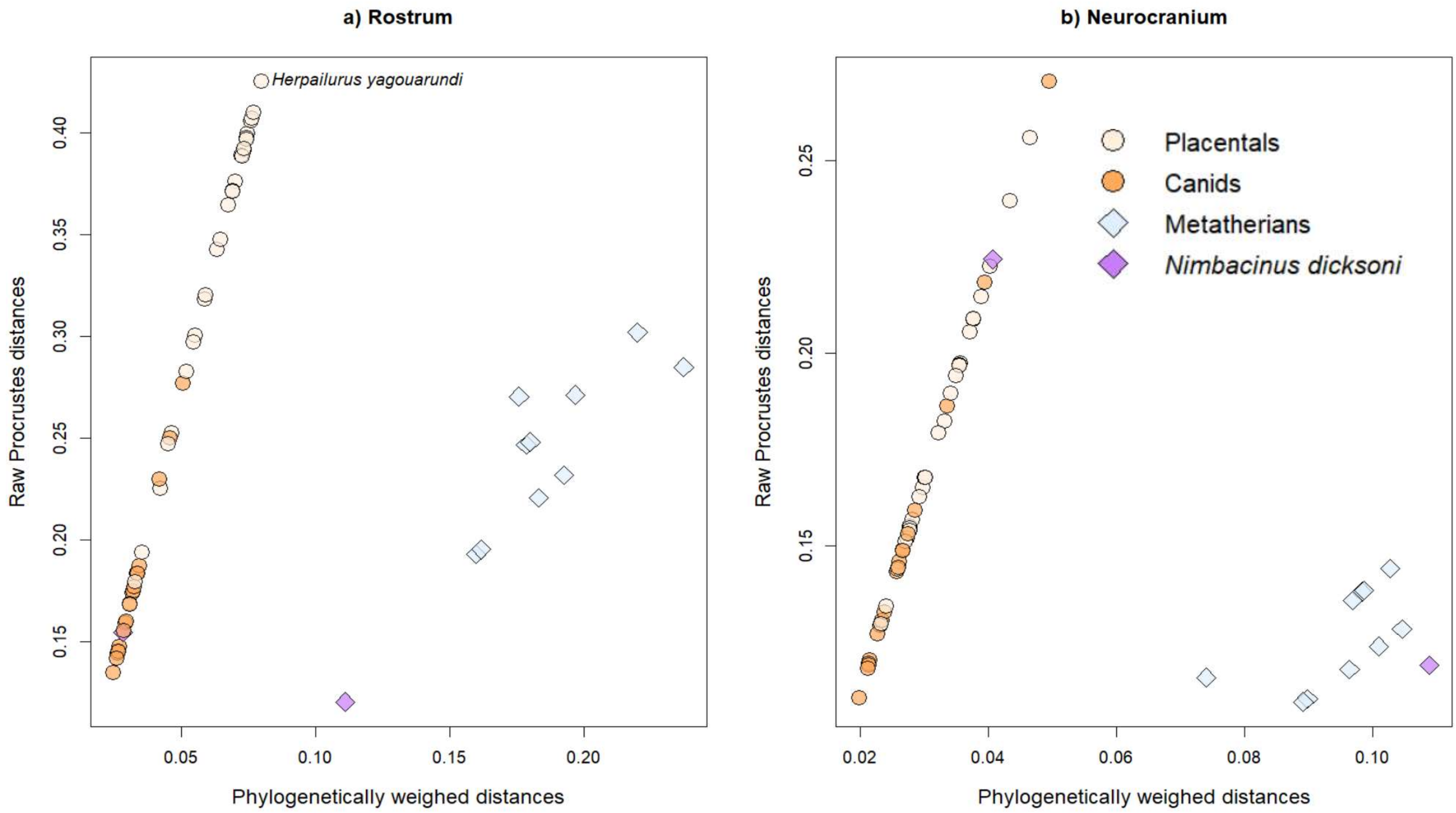

Supplementary Figure 13: Raw vs. weighted Procrustes distance plots. Plot of the raw Procrustes distances of mean shapes for all landmarked species from the thylacine mean shapes against the phylogenetically weighted distances. All species on the right are metatherians.

In Grunstra et al. (2024)[2], the weighing of distant pairs as zero it is much less pronounced because the phylogeny has a single-species outgroup. This means phylogenetic distance matrix has only one line of zeroes, between the platypus and other mammals, leading to a much more even transformation of distances.

2) The apparent over-correction using the phylogenetic distance matrix might be exacerbated by an issue with the trait standardisation recommended by Arbuckle (2014)[1], which cannot be done for 3D data. Arbuckle intended the phenotypic trait distances to be “standardized for variance by dividing by the standard error of the trait across species”. There is no equivalent operation for this for Procrustes coordinates (the superimposition step is a scaling, not a standardising step). It might technically be possible by using Principal Components as individual traits to be standardised, but this would in our view over-process the data. We suspect that use of coordinate data might make the method vulnerable to issues like ours (although they obviously provide clear information under the right conditions, as the Grunstra paper[2] shows).

3) Arbuckle (2014) also intended the Wheatsheaf index to be used only after convergence is established through other statistical means. Based on Rovinsky's (2021)[3] results, this allows us to test for convergence between the thylacine and canids. However, this means that we only have two focal groups - the related canids including the unrelated thylacine versus all other species. The method then asks if the mean of the phylogenetically corrected distances within that group is lower than in the non-focal groups. But averaging twenty canid values with one thylacine value will not provide good information specifically on the canid-thylacine pair.

4) For the same reason of needing to previously demonstrate convergence, creating a "Wheatsheaf matrix" (as suggested by the reviewer) does not apply the Wheatsheaf index as intended by Arbuckle (2014). Nevertheless, we assessed how a Wheatsheaf-like ratio would be calculated for thylacine-wolf and thylacine-canids, relative to marsupial-wolf, wolf-marsupial, and marsupial-canid pairs (the latter with the focal species removed from calculation of the mean distance calculations). The results for a few pertinent comparisons are in Supplementary Table 8; percentages indicate how much greater the similarity between a pair is compared to the comparative group, as also done in Grunstra (2024)[2])

Supplementary Table 8Table with "Wheatsheaf index" and percentages of similarity. Percentages show how much greater the similarity between a focal pair (named first) is compared to the comparative group (named second). WI, Wheatsheaf Index.

	Thylacine-Canidae vs Metatheria-Canidae WI	Thylacine-Canidae vs Metatheria-Canidae %	Thylacine-Wolf vs. Metatheria-Wolf WI	Thylacine-Wolf vs. Metatheria-Wolf %	Wolf-Metatheria vs. Canidae-Metatheria WI	Wolf-Metatheria vs. Canidae-Metatheria %	Wolf-Thylacine vs. Canidae-Thylacine WI	Wolf-Thylacine vs. Canidae-Thylacine %
Skull	1.197	16.5	1.285	22.2	1.102	9.2	1.169	14.5
Rostrum	1.241	19.4	1.24	19.3	0.947	-5.6	0.927	-7.9
Neurocr.	1.175	14.9	1.267	21.1	1.308	23.6	1.407	28.9
FNP	1.306	23.4	1.748	42.8	1.135	11.9	1.489	32.8
PA	1.094	8.6	1.166	14.2	0.991	-1	1.046	4.4
MES	1.17	14.5	1.495	33.1	1.263	20.9	1.6	37.5

This essentially confirms the patterns we observed with just the raw distances: Wolves are much more similar to the thylacine and metatherians as a whole in the back-of-skull neurcranium/Mes relative to the front-of-skull rostrum and FNP; but for the reasons outlined above, we decided to just present the Procrustes distances in the main manuscript.

References for Supplementary Note 2

1 Arbuckle, K., Bennett, C. M. & Speed, M. P. A simple measure of the strength of convergent evolution. *Methods Ecol. Evol.* **5**, 685-693 (2014). https://doi.org/https://doi.org/10.1111/2041-210X.12195

2 Grunstra, N. D. S. *et al.* Convergent evolution in Afrotheria and non-afrotherians demonstrates high evolvability of the mammalian inner ear. *Nature Commun.* **15**, 7869 (2024). https://doi.org/10.1038/s41467-024-52180-1
3 Rovinsky, D. S., Evans, A. R. & Adams, J. W. Functional ecological convergence between the thylacine and small prey-focused canids. *BMC Ecol. Evol.* **21**, 58 (2021). https://doi.org/10.1186/s12862-021-01788-8

Supplementary Figure 14

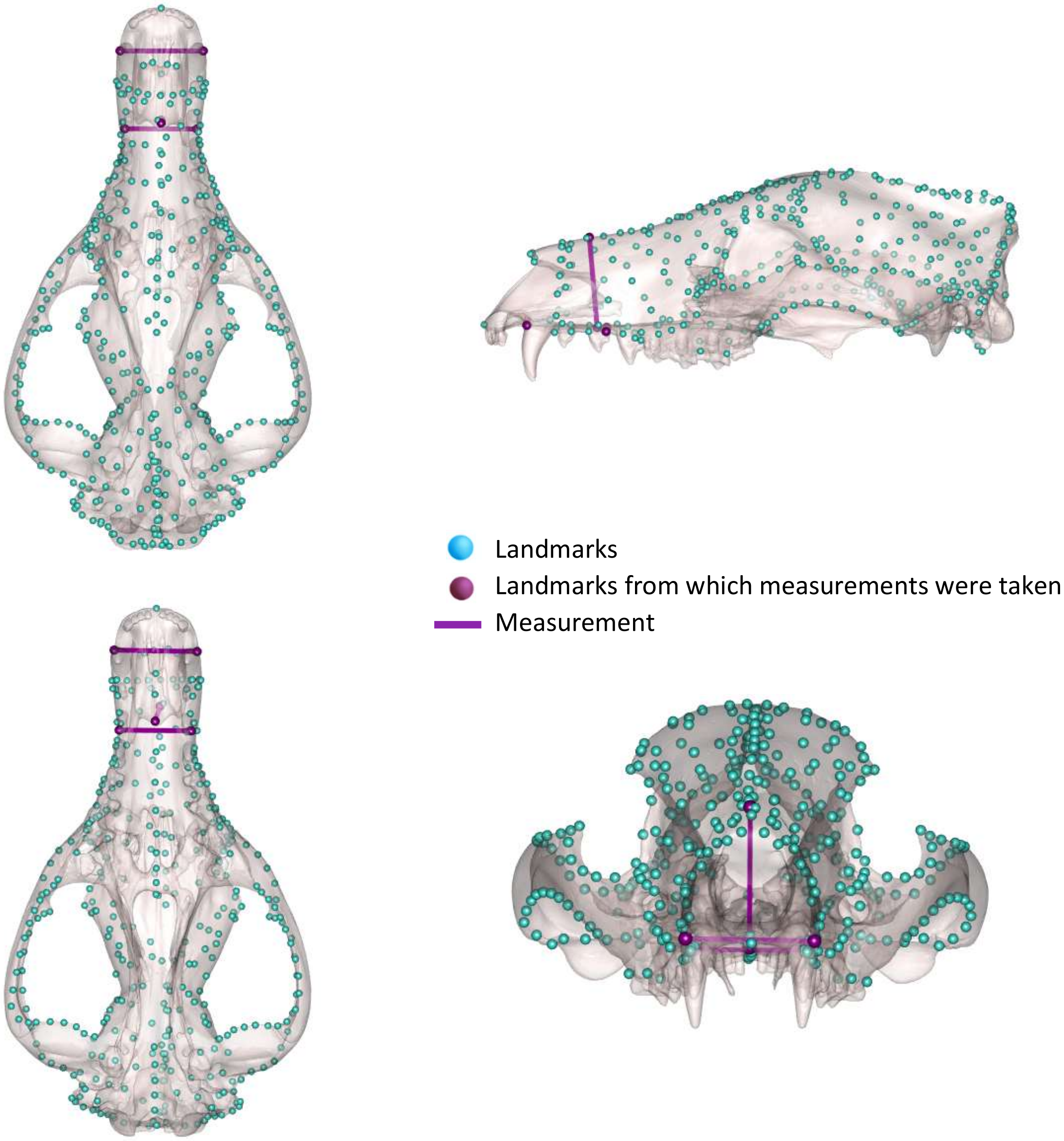

Rostral "pinch" distance measurements. Cranium of *Thylacinus cynocephalus* NMV 5749 displaying all landmarks and the distance measurements from which the plots on Fig. 5 are derived
