# Supplementary material for: Skull morphology of the extinct Tasmanian tiger suggests unique biting style
Source: Nat Commun. 2026 Aug 31;17:8729. doi: 10.1038/s41467-026-76614-0 (PMC13530167; doi:10.1038/s41467-026-76614-0)
Supplement: Supplementary file 2 [file 41467_2026_76614_MOESM2_ESM.pdf]

Description of Additional Supplementary Files

File Name: Supplementary Data 1
Description: 3D landmark coordinates for the geometric morphometric analyses

File Name: Supplementary Data 2
Description: Linear measurements for intraspecific comparisons between canids with the thylacine, with accession numbers

File Name: Supplementary Data 3
Description: Infraorbital foramen measurements

File Name: Supplementary Data 4
Description: Phylogeny used in this study
