# Supplementary material for: Skull morphology of the extinct Tasmanian tiger suggests unique biting style
Source: Nat Commun. 2026 Aug 31;17:8729. doi: 10.1038/s41467-026-76614-0 (PMC13530167; doi:10.1038/s41467-026-76614-0)
Supplement: Supplementary file 3 [file 41467_2026_76614_MOESM7_ESM.pdf]

nature portfolio

Corresponding author(s): Vera Weisbecker
Douglass Rovinsky

Last updated by author(s): 15/11/2024

Reporting Summary

Nature Portfolio wishes to improve the reproducibility of the work that we publish. This form provides structure for consistency and transparency in reporting. For further information on Nature Portfolio policies, see our Editorial Policies and the Editorial Policy Checklist.

Statistics

For all statistical analyses, confirm that the following items are present in the figure legend, table legend, main text, or Methods section.

n/a	Confirmed	
☐	☒	The exact sample size (*n*) for each experimental group/condition, given as a discrete number and unit of measurement
☒	☐	A statement on whether measurements were taken from distinct samples or whether the same sample was measured repeatedly
☐	☒	The statistical test(s) used AND whether they are one- or two-sided<br>*Only common tests should be described solely by name; describe more complex techniques in the Methods section.*
☐	☒	A description of all covariates tested
☐	☒	A description of any assumptions or corrections, such as tests of normality and adjustment for multiple comparisons
☐	☒	A full description of the statistical parameters including central tendency (e.g. means) or other basic estimates (e.g. regression coefficient) AND variation (e.g. standard deviation) or associated estimates of uncertainty (e.g. confidence intervals)
☐	☒	For null hypothesis testing, the test statistic (e.g. *F*, *t*, *r*) with confidence intervals, effect sizes, degrees of freedom and *P* value noted<br>*Give P values as exact values whenever suitable.*
☒	☐	For Bayesian analysis, information on the choice of priors and Markov chain Monte Carlo settings
☒	☐	For hierarchical and complex designs, identification of the appropriate level for tests and full reporting of outcomes
☒	☐	Estimates of effect sizes (e.g. Cohen's *d*, Pearson's *r*), indicating how they were calculated

Our web collection on statistics for biologists contains articles on many of the points above.

Software and code

Policy information about availability of computer code

Data collection

3D landmark data were collected using the Viewbox software (v. 4.1.0.12; dHAL software, Greece). For digital reconstruction of Borhyaena and Nimbacinus skulls, we used Materialise Mimics software (v. 20). Small holes were then filled in the Artec Studio 14 Professional (Artec Group, Luxembourg). We adjusted for plastic deformation and performed mesh resurfacing in Blender software 3.2.282 and the R package Morpho v. 2.1383.

Data analysis

All analyses were conducted in R v4.5.2 using a range of R packages (all cited in the manuscript):
ape v. 5.8-1
caper v.1.0.4
evomap v.2
geiger v. 2.0.11
geomorph v. 4.0.10
Morpho v. 2.13
nlme v.3.1-168
phangorn v.2.12.1
phygools v2.5-2
rgl v.1.3.34
RRPP v.2.1.2
shapes v1.2.8
smatr v.3.4-8

All code to replicate the Data analysis are available on github, with a fixed release via zenodo link https://doi.org/10.5281/zenodo.20268386

For manuscripts utilizing custom algorithms or software that are central to the research but not yet described in published literature, software must be made available to editors and reviewers. We strongly encourage code deposition in a community repository (e.g. GitHub). See the Nature Portfolio guidelines for submitting code & software for further information.

Data

Policy information about availability of data

All manuscripts must include a data availability statement. This statement should provide the following information, where applicable:

- Accession codes, unique identifiers, or web links for publicly available datasets
- A description of any restrictions on data availability
- For clinical datasets or third party data, please ensure that the statement adheres to our policy

All raw landmark coordinates, linear measurements, and phylogeny are are available on github, with a fixed release via zenodo link https://doi.org/10.5281/zenodo.20268386. Three-dimensional meshes which the landmark dataset is based on are on MorphoSource repository ID 0000C1004 (https://www.morphosource.org/projects/0000C1004) except for the mesh of Nimbacinus dicksoni, which we requested from Michael Archer (m.archer@unsw.edu.au).

Research involving human participants, their data, or biological material

Policy information about studies with human participants or human data. See also policy information about sex, gender (identity/presentation), and sexual orientation and race, ethnicity and racism.

Reporting on sex and gender	This is not applicable because this study did not include or report on human data.
Reporting on race, ethnicity, or other socially relevant groupings	This is not applicable because this study did not include or report on human data.
Population characteristics	This is not applicable because this study did not include or report on human data.
Recruitment	This is not applicable because this study did not include or report on human data.
Ethics oversight	This is not applicable because this study did not include or report on human data.

Note that full information on the approval of the study protocol must also be provided in the manuscript.

Field-specific reporting

Please select the one below that is the best fit for your research. If you are not sure, read the appropriate sections before making your selection.

☐ Life sciences ☐ Behavioural & social sciences ☒ Ecological, evolutionary & environmental sciences

For a reference copy of the document with all sections, see nature.com/documents/nr-reporting-summary-flat.pdf

Ecological, evolutionary & environmental sciences study design

All studies must disclose on these points even when the disclosure is negative.

Study description	The study used several analyses<br>1) 3D geometric morphometric landmark data, analysed with multivariate techniques (PCA, Procrustes distances, multivariate regression with centroid size as independent variable)<br>2) Log-transformed inear morphometric data, analysed relative to overall size in phylogenetically adjusted linear regression as described in manuscript, against log geometric mean, log of body mass, or log of other linear measurements .
Research sample	Our dataset is based on an existing three-dimensional (3D) landmark dataset (Rovinsky 2021), consisting of 222 specimens from 57 mammalian faunivorous mammals, covering 12 families. The protocol included 381 landmarks (46 fixed, and 191 curve and 144 surface semilandmarks). We also added three new specimens to this dataset.<br><br>To compare the skull proportions of the thylacine and canids without the multidimensionality of 3D landmark datasets77, nine linear metrics (condylobasal length, occipital condyle width, width across the carnassials, palate length, width across the canines, interorbital width, width across the postorbital processes, width at the postorbital constriction, and zygomatic breadth; see Fig. 4) were recorded from the crania of 73 thylacines (representing the largest published sample of measurements of this species) and disparate species of canid : grey wolf (Canis lupus; n=27), dingo (n=37), coyote (Canis latrans; n=24), side-striped jackal (Lupulella adusta; n=10), Pampas fox (Lycalopex gymnocercus; n=9), and red fox (Vulpes vulpes; n=27).
Sampling strategy	No sample-size calculation is usually performed in GMM studies because the sample sizes for comparative evolutionary studies are limited by the number of taxa and museum specimens available for study. However, the sample size generally recommended for datasets like this is around 40 (e.g. Polly, P. D. 2005. Evol. Dev. 7: 29–41) and accurate results can be achieved with less (Weisbecker et al. 2019, Frontiers in Zoology 16:41), so that our sample of 60 species (from 225 underlying data points) is on the larger side for studies like this.<br>Similarly, the linear morphometrics datasets were limited by available specimens in global museum collections, but we computed/

	visualised appropriateness of fit, and effect sizes for all analyses (see methods/results/supplementary Table 2) to ensure that our results were appropriate.
Data collection	3D data were all collected by Douglass Rovinsky using Viewbox (see above)<br>Linear data for cranial dimensions were also collected by D. Rovinsky, using callipers.<br>Linear data for the Infraorbital foramen analysis was augmented from two other publications (see methods) using the Artec Studio software.
Timing and spatial scale	We are unsure if this applies, but new Geometric Morphometric data and infraorbital foramen data were collected in 2022/2023 and linear measurements were taken during a period of museum travel in 2018, all by D. Rovinsky
Data exclusions	As outlined in the extended results, the allometry analyses had high non-normality with the inclusion of the gigantic species Andrewsarchus mongoliensis. This datapoint was therefore removed from the allometry analyses after ensuring that it did not impact on the main variation of our ordinations.
Reproducibility	There was no experimentation but all raw data and code are provided to replicate all aspects of the study.
Randomization	We had no grouping variables
Blinding	Blinding was not possible because the data collection was on identified specimens. Data analysis was not dependent on sample identity so in that sense, analysis was blind.

Did the study involve field work? ☐ Yes ☒ No

Reporting for specific materials, systems and methods

We require information from authors about some types of materials, experimental systems and methods used in many studies. Here, indicate whether each material, system or method listed is relevant to your study. If you are not sure if a list item applies to your research, read the appropriate section before selecting a response.

Materials & experimental systems

n/a	Involved in the study	
☒	☐	Antibodies
☒	☐	Eukaryotic cell lines
☒	☐	Palaeontology and archaeology
☐	☒	Animals and other organisms
☒	☐	Clinical data
☒	☐	Dual use research of concern
☒	☐	Plants

Methods

n/a	Involved in the study	
☒	☐	ChIP-seq
☒	☐	Flow cytometry
☒	☐	MRI-based neuroimaging

Animals and other research organisms

Policy information about studies involving animals; ARRIVE guidelines recommended for reporting animal research, and Sex and Gender in Research

Laboratory animals	No laboratory animals were used in this study
Wild animals	Our sample was exclusively of bones from museum collections, with all specimens appropriately accessioned and accession numbers documented in our supplementary information.
Reporting on sex	This is not applicable because this study did not include or report on human data.
Field-collected samples	This is not applicable because this study did not include field-collected samples.
Ethics oversight	No ethics approval was required because all animals were from official museum collections and obtained by these under the relevant regulations.

Note that full information on the approval of the study protocol must also be provided in the manuscript.

Plants

Seed stocks	This is not applicable because this study did not include plant data
Novel plant genotypes	This is not applicable because this study did not include plant data
Authentication	This is not applicable because this study did not include plant data
