# Supplementary material for: Skull morphology of the extinct Tasmanian tiger suggests unique biting style
Source: Nat Commun. 2026 Aug 31;17:8729. doi: 10.1038/s41467-026-76614-0 (PMC13530167; doi:10.1038/s41467-026-76614-0)
Supplement: Supplementary file 4 [file 41467_2026_76614_MOESM8_ESM.pdf]

nature portfolio

Peer Review File

Skull morphology of the extinct Tasmanian tiger suggests unique biting style

Corresponding Author: Professor Vera Weisbecker

Parts of this Peer Review File have been redacted as indicated to remove third-party material.

This file contains all reviewer reports in order by version, followed by all author rebuttals in order by version.

Attachments originally included by the reviewers as part of their assessment can be found at the end of this file.

Version 0:

Reviewer comments:

Reviewer #1

(Remarks to the Author)
This manuscript deals with the longstanding topic of convergence between marsupials and placentals. Specifically, the authors aim at describing the extent of convergence between the iconic, and recently extinct, marsupial wolf Thylacinus cynocephalus with other placental carnivores.
The topic is certainly of interest as evolutionary convergence has fascinated biologists, zoologists and palaeontologists for decades. Nonetheless, the authors decomposed the cranial shape into developmental and functional modules, which is something I found enticing.
However, the study presents several conceptual and methodological limits that I am listing below.

1) Despite the presence of an abundant literature on convergence, it was surprising that the authors did not provide a meaningful definition of it. This is far from trivial as convergence can be defined in multiple ways (see Stayton, 2015a). Without a clear definition of what you are trying to demonstrate (or test), it is hard to contextualize the present study. In fact, it is not clear whether the authors aim at showing the thylacine convergence with canids or its uniqueness. Also, it is not clear whether convergence should be achieved via developmental, functional processes. This confusion is widespread throughout the manuscript as both processes are invoked to occur, suggesting that pattern-based (e.g. the independent acquisition of similar characters) and process-based (e.g. when a specific process is demonstrated a priori) definitions have been conflated here (see Stayton, 2015b). This is also evident as the scenario (or the pattern) under which thylacines and canids should produce similar phenotypes is never mentioned. This had notable consequences on the methodological framework chosen to identify and quantify the extent of convergence (see Grossnickle et al., 2024).

2) I am sorry, but the methods are not adequate to test for convergence. First, the extent of convergence must be quantified under a phylogenetic scenario, otherwise it would be impossible to understand if two taxa, independently, evolved toward a similar phenotype. This underlies that the direction of evolution (e.g. ancestor-descendant pathway) plays a central role in determining and quantifying the extent of convergent regimes (see Castiglione et al., 2019). In this scenario, the use of Procrustes distance does not yield the results described by the authors. Basically, you measured the distance between landmarks configurations and a reference. Here, two points can very occupy the extremes of the space, such as +1 and -1, though being equidistant from 0 (the reference). Although, the shapes at +1 and -1 will be very different from each other, and from the reference as well. This means that using Procrustes distance is not only silent about the ancestor-descendant directions (see also other comments below), but also silent about the phylogenetic history of the focal clades, basically impeding any evaluation of convergence. This was somewhat surprising as there are currently multiple efficient strategies to measure the extent of convergence with highly multivariate data (see Castiglione et al. 2019; Grossnickle et al., 2024), or with univariate data (see Ingram and Mahler, 2013; Arbuckle et al., 2014).

In summary, I consider this study too immature to fully evaluate the extent of a complex evolutionary phenomenon such as convergence. Hence, I am sorry, but I cannot recommend this manuscript for publication. However, I hope that my comments and listed papers could serve as a guidance to thoroughly explore such an enticing topic.
Below are listed also other minor comments on the manuscript structure, that deserve attention from the authors.

Stayton, C. T. (2015a). The definition, recognition, and interpretation of convergent evolution, and two new measures for quantifying and assessing the significance of convergence. Evolution, 69(8), 2140-2153.
Stayton, C. T. (2015b). What does convergent evolution mean? The interpretation of convergence and its implications in the search for limits to evolution. Interface focus, 5(6), 20150039.
Grossnickle, D. M., Brightly, W. H., Weaver, L. N., Stanchak, K. E., Roston, R. A., Pevsner, S. K., ... & Law, C. J. (2024). Challenges and advances in measuring phenotypic convergence. Evolution, qpae081.
Castiglione, S., Serio, C., Tamagnini, D., Melchionna, M., Mondanaro, A., Di Febbraro, M., ... & Raia, P. (2019). A new, fast method to search for morphological convergence with shape data. PloS one, 14(12), e0226949.
Ingram, T., & Mahler, D. L. (2013). SURFACE: detecting convergent evolution from comparative data by fitting Ornstein-Uhlenbeck models with stepwise Akaike Information Criterion. Methods in ecology and evolution, 4(5), 416-425.
Arbuckle, K., Bennett, C. M., & Speed, M. P. (2014). A simple measure of the strength of convergent evolution. Methods in Ecology and Evolution, 5(7), 685-693.

Other minor points
Abstract
Line 34 remove repetitions.
Line 38 remove full stops.

Introduction
Lines 40-42 I am not happy with this sentence. Multiple marsupial carnivores display morpho-functional convergence with other placental carnivores. Having differently shaped marsupials (koalas and kangaroos) does not hint to an “unexpected convergence”. I suggest removing this sentence, or to much better frame the issue (or biological bias) of convergence.
Lines 44-47 I do not like at all the word “anomaly” here.
Lines 51-53 This sentence does not work, please remove it.
Lines 59-61 This sentence is very poorly written. I had to read ref 22 to understand what you mean.
May be something like: “Nonetheless, thylacines display notable similarities with multiple skeletal regions (e.g. teeth, skull and forelimbs) of South-American metatherian sparassodonts [Kealy and Beck 2017]. This also challenges the accurate phylogenetic reconstruction of the metatherian, as elegantly shown in [ref 22].”

Results and Discussion
Lines 78-81. How did you test it? Here, you are just looking at two PC axes, this looks too speculative to me.
Lines 82-84. Please rephrase. Put it in a more scientific context, begin saying that attributing a clear ecology to thylacines has been quite a task considering that it is not really that similar to any other species.
Line 99. I am not sure what a “marsupial-like pattern of resemblance” is.
Lines 111-113. Same as above, how did you test it? Again, this is way too speculative without using any phylogenetic comparative method. Also, such putative “departure” could be very well ascribed to within clade allometry.

Figures.
Figure 1 is missing the legend of the scatterplot. Please rework it.

(Remarks on code availability)
I accessed the codes. I perfectly understood the methods used and, as detailed in my review, they are not suited to test the proposed hypotheses.

Reviewer #2

(Remarks to the Author)
In this paper, Rovinsky et al. utilize geometric morphometrics and linear measurements to examine thylacine cranial morphology in the context of marsupials, canids, and three related fossils. They make functional predictions based on comparison with extant taxa. These results are clearly of interest both to those generally interested in functional morphology and those interested in this captivating animal.
This is a great paper and the manuscript is well written with beautiful visual interpretation of results. That being said, we have a few minor recommendations – NONE of which should be considered “required”. If this paper were published as is, it would be an important and worthy contribution. With that said…

The only substantial issue that we struggled with in this paper is understanding the comparative sample and specific data collected. We are confident that we struggled with this because this sample is built on a sample previously described in another paper, but we do not generally consider it good practice to require readers to go to other papers to understand things that are necessary for understanding the current paper. In other words, we think this sample needs to be included in this paper – at least as a supplement – as should be more details about the measures. For instance, if the comparative sample included mostly animals with long pointy faces, then that would mask convergences and emphasize divergence within that morphospace. Does this only include carnivores? Also, we are confused by some of the measures including references to “carnassial”. Obviously, these do not exist in thylacines and other marsupials, so a description of this measure and others based on non-homologous structures seems necessary.

Other than that, there are only minor typos that need correcting:

L19: e.g.,
L32: missing space after –
L34: "an important" is repeated
L37: "Recent studies" seems to need some citations.
L38: Doubled periods
In the first paragraph of the Results and Discussion, we feel it would be of value to contextualize generally what morphology is driving differences PC1 and PC2.
L82: "resembles a both"
Fig. 1 needs an explanation of the color/symbol code. Seems to be same as subsequent figures but needs to be explained at least in this first use if not repeatedly.
Fig. 3: The canids seem to all have positive residuals, right? A significance statement about that would be valuable.
L155: again, we are confused about marsupial carnassials.
Fig. 4: This is a really beautiful and informative figure. No problems, just kudos!
L182: is "<<" intentional?
Fig. 6: what do the hulls signify? They SEEM to be metas vs. placentals, but some of the metatherians are outside of the blue shape. Why?
L300: "subsequent" should be "subsequently."
L352: please cite which literature was used for size proxies
L362: are domestic dogs and grey wolves really lumped together in this sample?! Are highly derived dogs included in here? Dingos? From the structure of this writing (i.e., under the phylogeny subheading) it seems that this sensu lato use is only on the phylogenetic analysis, not the morphological one, right? Maybe that needs to be stated directly?
L385: Eileen Westwig hasn't been at the AMNH for a LONG time, but if she was there when the data were collected then I guess that should still be the acknowledgment? Maybe "then at the…"?

Again, this is a great paper and all of these issues are more for the authors to think about or clean up before final proofs. As it is, it is essentially acceptable.

(Remarks on code availability)

Reviewer #3

(Remarks to the Author)
A BETTER FORMATED VERSION OF THIS REVIEW HAS BEEN UPLOADED AS A PDF

In this manuscript, the authors present a series of arguments suggesting that the thylacine was the last surviving member of a predator group employing fast, high-impact snapping to capture their prey. Surprisingly, they posit that this prey might have been fish instead of small terrestrial vertebrates (as suggested by current hypotheses on thylacine dietary preferences and hunting habits). I'm afraid I don't share the authors' confidence on the implications of their results, neither think I that enough evidence is provided to support that claim.
Overall, I think this is a well-written manuscript presenting novel and interesting results on thylacine (and canid) cranial shape variation, while at the same time linking them to recent studies on cranial development and genomics on those species. My major concern is that the authors focus on their narratives (wolf vs thylacine, extinct predator group) so deeply that they seem to disregard any alternative potential explanations to their results. A few examples would be:
- in some cases the results are presented in a way that could bias the reader (e.g. see comments on L73-76 below).
- most of the Results & Discussion section is focused on the differences and similarities between the thylacine and the wolf (representing large-prey predators), but the same comparison is not made with the small-prey specialists. In fact, any comparison with other large prey specialists, like Cuon or Lycaon is also missing. This could be particularly important when discussing Figure 3A (see comment for L127 below).
- despite being most similar when combining cranial shape and size, Chrysocyon is disregarded as a potential ecological equivalent based on its diet (L132-138) but… isn't the whole purpose of this study to try to understand the thylacine diet? Couldn't it also be an omnivorous species eating both small mammals and fruits? A similar disregard of Canis simensis occurs later in this section (L199-203).
Thus, I would strongly recommend the authors to tone down this narrative by presenting it as a potential explanation, along with several others, of their results.
For more detailed comments, please refer to the line-by-line section below.
Apologies in advance if some of my comments seem too harsh, that was not my intention. I might have issues with how the results are discussed in the manuscript, but I do not wish to undervalue in any way the work or ethics of the authors.
Eloy Gálvez López (anonymity waived)

L2: Author order is different in the manuscript than in the system (emails, reviewer dashboard, etc.). I assume the one in the manuscript is the correct one?
L34: Remove repeated "an important".
L38: Remove repeated period.
L39: This might read as "they're similar in cranial shape", as convergent is often used very loosely in the literature. However, since this has been statistically tested in Rovinsky et al (2021), I would recommend rewriting this as "[…] and have been shown to present convergent cranial shapes with canids" (or something similar). This change will also strengthen other

instances when convergence in skull shape is brought up (e.g. L72).
L268-287: I would add "(see below)" before the colon to avoid the reader wondering about the nature and contents for Rovinsky et al (2021)'s sample for two paragraphs. Alternatively, the authors could rewrite this paragraph to incorporate lines 284-287: "For this study, we used Rovinsky et al's cranial dataset of 381 three-dimensional (3D) landmarks (46 fixed […]) of 225 specimens from 60 mammalian taxa (including 17 thylacine crania; Suppl. Data 1), to which we added three new specimens using the Viewbox 4 (…) protocol established in that study: the Argentinian […]"
Regardless of which approach is selected, please reference Figure 2 on the parentheses describing the landmark configurations. Since there is no proper figure for it in this study (yes, I know it's in Rovinsky et al (2021)), linking to a figure where the configurations are shown would be helpful for those of us who always read the Methods section before the Results.
L288: Why are the zygomatic arches not included in either partition? They are a good indicator of masticatory muscle size, and hence have significant biomechanical implications.
L294-303: This paragraph is quite convoluted. It took me a few reads to understand that it meant "We visualized shape variation in each partition using PCA (Fig. 2). Similarities in cranial shape were assessed using Procrustes distances, and the closest species to the thylacine are shown in Figure 3." I would recommend expanding that summary as needed, but please keep it simple and direct. Also please mind the references to the figures. As mentioned above, it helps readers who don't read the manuscript following the published section order.
L306: I would remove the "felids vs. canids" example (and just leave the other one), as the former are only mentioned here and the latter are only compared to thylacinids.
L307-311: I tried accessing the R script with the links provided but I kept getting errors, hence this comment. Did you use procD.pgls to calculate the size effect on cranial shape or procD.lm (or procD.allometry)? The Extended Results mention Procrustes Generalized Least Squares (which usually refers to procD.lm) for shape instead of Phylogenetic GLS (procD.pgls), so I wonder whether phylogeny was included in the regression or not. If indeed it was, please edit the Extended Results.
L325: Please add "Figure 4" at the end of the parenthesis to guide readers to a visualization of (some of) the measurements taken.
L327-328: Please give species names for all species. Why grey wolf but just "South American canids" or "fox"?
L. adusta – side-striped jackal; L. gymnocercus – Pampas fox (or Azaras' zorro if you want to distinguish South American "foxes" from vulpine foxes); V. vulpes – red fox
L335: Remove repeated "then".
L340 & Figure 5: Two related comments. First, please link the description to Figure 5 to direct readers to a visual aid ("The rostrum of the thylacine (Figure 5) is […]"). Second, either replace the drawings in figure 5 by images of the actual cranium (or mesh, ideally with the relevant landmarks) or add those as separate panels for the figure (ideally for all the species highlighted). While the simplified drawings might be very clear to the authors, I'm still not sure that I understand them.
L343-344: You use "prediction range analysis" here but "prediction interval comparisons" later on L356. For consistency, please change this sentence to "As with cranial size, we calculated the 95% prediction intervals of the regression to ask whether […]".
L361: I think there's a double spacing between Nimbacinus and using.
L361-366: This paragraph is useful when you read the Methods section before Results and Discussion, but probably not in any other situation. I would suggest moving this to the Introduction (maybe between L57-58 when discussing small- vs large-prey specialists).
L73: Here I'd go deeper than "aligning with previous studies". The cranium PCA morphospace is almost identical to that of Rovinsky et al (2021) even after adding the extreme Andrewsarchus, and leaning into that would prevent readers from wondering whether its inclusion is affecting the direction of PC1 in a similar way than it did on the shape vs CS regressions.
L73-76: For me, this is the first red flag regarding the forced narrative I mentioned above. By saying that PC2 "only" explains 21% of shape variation vs PC1 explaining "over half" of said variation, the authors are planting the notion on the readers that PC2 should be disregarded. First, for a study using 381 3D landmarks, we would expect over 1100 PCs to be generated, which means that by chance alone, each one should explain under 0.1% of shape variation. Thus, 21% of shape variation is not a small amount. Second, according to Figure 1B, PC1 explains 42.82% of shape variation, which is not over half.
A more objective description would be: "However, the thylacine lies just outside of the canid region of the PC1-PC2 morphospace, which represents over 60% of cranial shape variation. While the low scores of the thylacine on PC2 fall close to the canid range (excluding the widely divergent Speothos*), the overall distinctness of the thylacine cranium from both metatherians and placentals is emphasized on PC1. Here, metatherians […]"
Furthermore, this more objective description provides stronger support to using PC2 to illustrate the divergence between Thylacinus and Nimbacinus (L79-80), as its importance has not been minimized previously.
* I assumed the canid on the top left region of Fig. 1B is Speothos due to its unusual morphology, but please correct that if I'm wrong.
L79, L82: To avoid confusion, change to "(Fig. 2A, circles)".
L82: Please remove "a" before "both".
L87: Potential double space after the first period.
L95-97 (Figure 1): Please add the same legend as in Fig 3, and provide species names on panel 1B for the canid "outliers" (top left and lowest PC2 score).
Also, why is the polarity of PC1 inverted in the corresponding panels of Suppl. Fig. 1?
L103: Could these distance values be provided with the supplementary material? Since not all canids are represented on Figure 2, is hard to imagine that the canid species with negative PC1 values are closer to the thylacine than the wolf is.
L104-106: These lines could be a great segue to discuss differences and similarities with other large prey-specialists and with small-prey specialists (e.g. the red fox is the closest non-thylacinid in rostrum shape). This would provide a more nuanced interpretation of the results.
L111-113: While the rest of this paragraph does a good job in linking recent genetic studies with the shape variation

described here, I think this sentence pushes the argument too far. This study neither includes any evolutionary modelling, nor presents any morphospace that, once turned into a phylomorphospace, would suggest cranial shape divergence between wolves and other canids. Thus, it needs to be removed.
I understand that the authors might want to make a big statement out of their findings, but this is the kind of sentence that is later referenced out of context and ends up becoming accepted without proper initial support.
L124: The suggestion for L104-106 would also add support to this sentence, as there has been barely no mention of other canids beyond the wolf.
L126: I think Suppl. Fig. 2 should be replaced by Fig. 3B (thus reducing Fig. 3 to the 3A panel) and a similar plot of the living marsupials presented in the current Suppl. Fig. 2. As it stands, the figure draws more attention on how relatively large the cranium of D. marsupialis is than to the thylacine, so it leaves the reader wondering why is that not discussed.
L126: Unless I have missed any regional spelling, it's "outsized" not "out-sized".
L127: Similarly, I would recommend writing "wolf equivalent" instead of "wolf-equivalent". In any case, I would remove "Consistent with its history of being considered a wolf equivalent," altogether, as it doesn't make sense with the rest of the paragraph. On the contrary though, if the two canids between the thylacine and the wolf in Fig. 3A, which also have particularly large crania relative to their size, are Cuon and Lycaon, that could be interpreted as canid large-prey specialists having wolf-sized crania regardless of body mass, which would then support the claim that the authors are trying to refute. (Based on the following paragraph I assume one of them is Chrysocyon, but what is the other?)
Another result of the log CS vs log body mass regression on Fig. 3A is that all canids have relatively large crania for their size, as they always fall on top of the regression line. Could then the convergence with canids explain why the thylacine lies over the marsupial regression line (Suppl. Fig. 2)?
L127-128: Either "the thylacines' cranial size puts them" or "the thylacine's cranial size puts it".
L132-138: As stated above, this is one of the clearest examples of pushing the extinct predator group narrative to the detriment of alternative hypotheses. Compounding on that, the authors list no references supporting the thylacine's hypercarnivory, with the listed references (26.27) corresponding instead to Chrysocyon's diet. Thus, this paragraph needs to be rewritten. Either further evidence of the thylacine's diet which negates the intake of fruits needs to be referenced, or the authors should acknowledge that an alternative hypothesis to their results would be that the thylacine was a predator of small-sized vertebrates which potentially complemented its diet with fruits.
L150-152: If I understand it correctly, the argument here is that the geometric mean, as a proxy of cranial size, increases with condyle width, as a proxy for body mass, with a higher exponent in the thylacine. If that is the case, I would strongly recommend the authors to invert the axes of Fig. 4A. As the visual interpretation of the current panel suggests a lower exponent for the thylacine, which I assume the RMA slope actually is if the geometric mean was used for the x values. While RMA regression incorporates residual error for both variables, it is still best practice to match the regression formula with the desired interpretation of the result. In this case, for the higher scaling of cranial size to body mass, condyle width should be used as independent variable (x).
L153: I don't think saying that this regression mirrors the interspecific differences in cranial centroid size, as they're comparing rates of change to average values, but I do think these results could serve as a hypothesis of why the thylacine have relatively large heads for their size: they increase in size relative to body mass faster than in canids. This leaves the question however, on whether that is a marsupial trend related to their altricial development.
L160: I would suggest removing "related" from this sentence to avoid the potential misinterpretation that the thylacine was a canid (despite how clearly the contrary is otherwise stated in the manuscript).
L163-165: This is another example of the authors pushing their narrative over alternative explanations. For once, the process is not as "extreme" as the authors claim, as for some of the studied variables, while different from larger canids, the thylacine's regression lines seem to extend trends observed in the smaller species. For instance, their scaling of both palate length and width at the canines is not significantly different than any of the studied foxes or jackals. Thus, another interpretation of the same results could be that, by having crania larger than similar-sized canids, they are able to maintain proportions similar to those of smaller canids, all small-prey specialists. This would support previous studies suggesting that the thylacine was a small-prey specialist (Attard et al. 2011; Rovinsky et al. 2021) probably using pounce-pursuit strategies (Jones & Stoddart 1998), so I am unsure on why this isn't included here.
Another topic is that, while the thylacine's gracilization has been shown to be different than that of some of the studied species, differences were also found within that pool of other species, yet this is disregarded. All this, coupled with the proliferation of studies on many-to-one relationships between bone shape and its function, makes the argument presented here a bit thin.
L173-184: This whole paragraph provides further support to the thylacine being a small canid equivalent, but yet the authors dance around that statement to maintain the extinct predator group narrative.
L188: As noted above (L340 & Figure 5), please provide a better visual aid for the reader to understand this "terminal rosette".
L190-192: While this might be true for crocodylians, the thylacine's rosette doesn't seem developed enough to grant this hammer-like properties. Maybe a better figure would help this argument, but as it is, I don't see enough support for this. Figure 5 illustrates a relative pinching at the canines, but that doesn't provide any insight on the mass distribution of the snout. A potential solution to this would be to calculate the voxel proportions of the rosette relative to the rest of the snout in the cranial meshes, but this might be a very rough approximation if the meshes were acquired using a surface scanner instead of CT.
L194-195: Relatively tall compared to what? Can this be observed in the shape changes along PC1 or PC2? Based on Fig. 1A, the thylacine's snout doesn't look taller than the wolf's.
L199-203: Would this make C. simensis also part of this potentially extinct predator group?
L211-219: This paragraph is the final leap on the extinct predatory group narrative: What if the small, fast-moving prey mentioned earlier weren't small mammals but fish? Then the thylacine might be a piscivorous predator because it has a "terminal rosette" like all these other clades.
I don't think that this manuscript provides enough evidence to support this claim.

L220-234: While interesting, this whole section on IOF doesn't feel connected to the rest of the manuscript, particularly when the lack of developed whiskers in the thylacine would be an argument against their piscivorous diet narrative. Why delve into it here then? I believe this might be an interesting short study on its own. Maybe add some other dasyuromorphs and map IOF surface during their evolution? Couple it with evolutionary modelling of dental over-eruption to test their hypothesis on soft tissue maintenance?
L237-243: See most of my comments above, but this needs to shift from present tense to a hypothetical conditional tense.

(Remarks on code availability)
I did not review the code because I was unable to access it, as it is locked to gitfront users.

Reviewer #4

(Remarks to the Author)
I co-reviewed this manuscript with one of the reviewers who provided the listed reports. This is part of the Nature Communications initiative to facilitate training in peer review and to provide appropriate recognition for Early Career Researchers who co-review manuscripts.

(Remarks on code availability)

Version 1:

Reviewer comments:

Reviewer #1

(Remarks to the Author)
This is a fully rewritten version of the manuscript I previously revised. I want to make clear that I sincerely appreciate the efforts taken by the authors to rework and make clearer their ideas and thoughts. Before moving on, I would like to make some clarification about the answers provided by the authors.
First;
ok, this is not a paper on convergence and this a shame. Possibly I was expecting/hoping to read a paper on convergence, but this was not the case. However, I still recommend to replace the word "convergence" with "similarities" throughout the manuscript. However, I deeply appreciated the effort taken by the authors to rework the manuscript. As a side note, the metrics adopted in Rovinsky et al. (2021) are outdated, so, just for fun, I would re-run some of those analyses, take also a look at Grunstra et al. (2024) have a better understanding of what is possible to do with Procrustes distances.

Second;
"With ~160 million years evolutionary divergence and distinct differences between placentals and metatherians, of which the thylacine is the only exception in aspects of its shape, there is no need for testing if these differences arose independently! In addition, this has been done before e.g. in Rovinsky et al. 2021. We are now clearer that this is a reasonable assumption in the introduction (2nd paragraph)."
I probably expressed myself badly here, sorry. However, I was meaning that in dealing with convergence you test whether two species significantly depart from their ancestors' shape toward a common area of the morphospace. That is, without a phylogenetic background it is impossible to trace such ancestor-descendant pathway, independently of how much evolutionary time separates the two focal taxa. This is far from a trivial as more often than not, putatively convergent taxa fail such test. Hope this is clearer.
"We did not, as R1 suggests, use Procrustes distance comparisons as a naïve and phylogenetically uninformed way of testing convergence. And, as outlined above, there is no relevance to include ancestor-descendant relationships because it is clear that any similarities between the thylacine and canids are not based on a common ancestral condition. Instead, our study goes back to the very basic but also deeply fundamental question of asking which animal the thylacine resembles most in different sections of its cranium, based on previous results (Rovinsky et al. 2021) that similarities between canids and thylacines are not consistent with any carnivoran ecomorph."
Sorry, but this is exactly what I said in my comment. You simply measured the distances between some species and the thylacine (here taken as reference), without accounting for phylogeny. There's nothing bad about it, but distances are silent other aspects, namely phylogenetic history. Furthermore, in my comment I haven't by any means claimed that PCA is better than using Procrustes distances, as it really depends on what your scientific design is about.

In this picture (see the attached file) is shown the full Procrustes distance (rho) and the partial Procrustes distances (Drho) between a reference (the red point) and an hypothetical configuration A. Now, what is generally measured, as you did, is the distance Drho. Now, you can imagine another configuration B exactly at the same Drho, but on the -1 side. That is, two configurations can have the same partial Procrustes distance from the reference, despite having different shapes, that's why we need to visually explore the shape changes. If the authors feel I am not adequate or competent in GMM, please have a read at the PalaeoMath Series from Norman MacLeod (https://www.palass.org/publications/newsletter/palaeomath-101) where these topics are treated in a much more mannered way.
Again, my comment was intended to make the author fully aware of the limits in using such distances.

Now, I hope everything is clearer and I can move on reviewing this new version, which is not about convergence. However, this version reads much better than the previous one and is way clearer about the study's aims.

However, I still have some concerns the main one related to the diminutive sample size available. I do not think that 59 species are capable of capturing the variability of placentals carnivores (280 species) and/or marsupials. As a matter of fact, you lack ursids, many felids, many viverrids and mustelids are very poorly represented in the sample. Nonetheless, you lack almost all of the largest carnivores (lions, tigers, brown and white bears), which could have added a lot more considering that an allometric analysis has been performed. Also, it would have been a good idea to add some more fossils, in particular sparassodonts (when available of course).
Another issue is represented by the allometric analysis. Below, I provided more details, but in summary running regression PC by PC is a very discouraged practice in GMM (please see any contribution from David Polly, Dean Adams or Philip Mitteroecker).
Another problem is the use of size as a proxy of bite force. This came out without any testing, and I am not very happy with such a choice. Cranial size is not the only determinant of bite force as muscle orientation and distance (to make an example) make a lot of difference. So please remove it or analyse it differently, like taking bite force data from other published venues where a proper biomechanical analysis was performed.

Minor points

Abstract
I recommend to minimise the use of the word “convergence” here and throughout the manuscript, as you did not measured it. Replace with “similarities” or something like that.

Introduction
Lines 71-73: This would have been interesting to test.
Lines 81-82: I have some issue with using size as a proxy of bite force.

Results and Discussion
Line 93. I have issues with such a diminutive sample size. Carnivora include around 280 living species and many more extinct (see Faurby et al. 2024). I do not think that this sample can be considered representative of the carnivoran variability. Also, as mentioned above, it would have been good to include some more sparassodonts as this could have shifted our view of thylacines to be more similar to canids than to other taxa.
Line 134: How can you say that dingoes and wolves are unique among canids?
Line 136-138: This is a fundamental problem with this manuscript and what I tried to make evident in my previous review. Without any proper phylogenetic comparative analysis, you can’t say anything about any putative departure of any species from their ancestors. This looks very speculative to me.
Lines 157-161: I got what you mean, but please rewrite it. Without having a look at the supp it’s hard to get your message. Anyway, it is highly discouraged to analyse single PCs separately, as you arbitrarily selected 10 PCs and performed allometric analyses PC by PC finding little evidence to say that allometry is meaningless in your data.
The correct way is to analyse the full set of Procrustes coordinates vs size (as you did at first). Here, since you get a significant result, it would have been nice to compare the allometric slopes of shape data. You could have run separate pooled regressions (e.g. separating Canidae, Felidae, etc.) and test whether these resulting slopes were convergent, divergent or parallel. Also, a deeper investigation of allometric slopes would have revealed whether the Thylacine can be considered “hypermorphic” compared to other marsupials.
In the end, please remove the PC by PC analysis as it go against any development in modern morphometrics and rephrase the paragraph.

Figure 4. I guess you miss-labeled the axes in figure 4a.

(Remarks on code availability)

Reviewer #2

(Remarks to the Author)
We believe that the authors did a great job either incorporating the modifications that we requested or addressed our concerns in their response. The only line edit we caught was Line 36: "observatione.g.,".

Other than that, we look forward to seeing this paper in print.

(Remarks on code availability)
NA

Reviewer #3

(Remarks to the Author)
Overall, this revised version of the manuscript has vastly improved upon the original, both in terms of structure and background information, and by addressing the comments of three different reviewers, so I wanted to congratulate the authors on that.
I particularly like the rewritten Introduction (but see comment for L292-298). Both the restructuring of its previous sections

and the added bits of information (particularly regarding the thylacine’s diet) help paint a clearer picture of what we currentlly know about the thylacine’s ecology and relevant morphology and what this manuscript aims to address.
Regarding the changes to the Methods section, I am unable to discern whether they’re easier to understand because of the rewrites, or because I already know what those analyses are from the previous versions. Hopefully other reviewers will add notes on that topic if they still think it’s unclear. No notes from me though.
As for Results & Discussion, my thoughts on the authors’ justification for not describing other species and just focusing on the wolf-thylacine comparison can be found at the end of this document (“Keeping the dialogue open” section), but I wanted to note here why I still slightly disagree with the authors in that regard. Specifically, there is a disagreement between the distance values plotted on Figure 2 and the distance values presented in Suppl. Table 1. If the latter are correct, the pattern described for the grey wolf and dingo (closest to thylacine in posterior cranium but far away in rostral region) applies to all Canis species except C. simensis, and to Cuon and Lycaon as well, not just the grey wolf and dingo. This shifts the results away from the uniqueness of the wolf and dingo relative to other canids and makes it a trend within the Subtribe Canina (now Lupulella and C. simensis are the exceptions to cranial shape/development within Canina). With this in mind, the authors could expand their hypothesis that wolf/dingo and thylacine both diverged from their ancestors to evolve a similar cranial shape, to this divergence occurring early on in the evolution of Canina, right after the Canis/Cuon/Lycaon branch diverged from Lupulella. C. simensis might represent a shift back to the ancestral canid condition, an early stage of divergence towards a new set of cranial features (see comment on L256-263), or their Ethiopian wolf specimens might be incorrectly assigned to that species*. If the authors agree with this interpretation, the overall discussion of their results could be easily shifted from the wolf comparison to a wolf-like canid comparison (i.e. Subtribe Canina).
* In my experience, misclassified specimens are common in museum collections, particularly when they were collected in the early 1900s or when they belong to species in distant continents.
Beyond that, I wanted to congratulate the authors again on a fantastic job improving this section. It reads much better, all my doubts regarding a specific narrative being pushed against other potential explanations have been quenched with clarifications and new analyses and improved figures, and there is no room for misinterpreting the authors as stating that the thylacine was piscivorous (sorry about that btw!). Congratulations!
Eloy Gálvez-López (anonymity waived)

L31: Change “carnivorous” by “hypercarnivorous”. I think this will solidify any potential doubts regarding thylacine diet.
L36: Fix typo: “observatione.g.”
L41: Either “similarities of the thylacine with canids”, or “similarities between the thylacine and canids”
L72: Fix typo: “sparassodontse.g.”
L386: That comma feels weird. I would remove it, but this is just a suggestion, not a request.
L397: You might want to rewrite this line as: “ We log10 transformed centroid sizes and regressed them against log10-transformed species’ average body masses […]”. I believe it’s shorter and easier to read.
L451-456: This paragraph no longer has a purpose, as “grey” has been added to all other relevant mentions of wolf (except L293), clarifying that the authors are referring to this species only. I would strongly recommend the authors to delete it.
L456: If the authors don’t remove those lines, the “prey30” typo needs to be fixed.
L124-L141 & Figure 2 / Suppl. Table 1: See comment above. This paragraph should be rewritten to acknowledge the discrepancy (and potential new evolutionary hypothesis), and the figure modified to present the correct values.
Extended Results: “Excluding A. mongoliensis from the analysis produced a nearly non-significant association (R2 = 0.33, p= 0.000) between cranial shape and log(cranial centroid size)”. I am quite confused with this statement. Why is this nearly non-significant? If this is based on p=0.0000, which is common when outputs are limited to 4 decimal places and the value is lower than 0.00005, the results are actually the opposite (i.e., excluding Andrewsarchus increased the significance of the allometric relationship). With significance set at p=0.05, a nearly non-significant result would be p=0.0499 or higher.
L159-160: This no longer seems to be true, based on my earlier comment regarding the disagreement between distance values in Figure 2 and Suppl. Table 1. I would recommend extending this argument to Canina instead.
L172: I don’t understand what you mean here. It reads as if something is missing after “absolute”.
L219: I assume defining small prey as < 10 kg is a ballpark threshold, as there’s no associated reference. If that’s the case, I would recommend changing this to < 7.5 kg, representing 45% of the thylacine’s latest body weight estimations (ca.16.7 kg). Small and large prey are usually defined relative to predator size and Carbone et al. (1999) proposed that 45% threshold based on the energetics of hunting and prey processing.
Carbone C, Mace GM, Roberts SC, Macdonald DW (1999) Energetic constraints on the diet of terrestrial carnivores. Nature 402, 286–288.
Figure 5: I just wanted to state how gorgeous this figure is now. Zero doubts about what the terminal rosette is. Fantastic job!
L247: Fix “see aso” typo.
L256-263: If the authors adopt the wolf-like canid comparison focusing on Canina instead of just grey wolfs and dingoes, this could be a great opportunity to explore why C. simensis does not follow the trends observed in other Canina. Could the Ethiopian wolf be an early state of divergence towards acquiring a morphology similar to the thylacine/Andrewsarchus? They seem to be developing a terminal rosette, could the development of a taller snout and relatively large head follow?
L274-287: I still don’t think the IOF analysis and discussion connects with the rest of the study, but the authors make compelling arguments for keeping it in their rebuttal.
L292-298: This paragraph is the perfect example of how vastly the authors have improved the manuscript in this revision. The paragraph itself has barely changed but, while in the previous version I thought it exemplified all my issues with the manuscript and I requested that it be changed to a conditional tense, now I believe is the perfect summary for the preceding section. Again, great job.
L326: “thylacine-canid comparison” instead of “thylacine canid-comparison”
L335-336: Delete spaces before reference numbers

--- Keeping the dialogue open ---

Besides my comments on the manuscript proper, I wanted to address some of the topics discussed by the authors in their rebuttal.

Why we do not discuss other species more?

While I agree with the authors in that, when space is limited, not all topics can be discussed, I still think it would have benefitted the manuscript to spend some time addressing the species closest to the thylacine in the morphospace. In fact, I think it would have add support to the uniqueness of the thylacine phenotype as it shows how many different ecologies cluster in that region of the morphospace (this could even be a prime example of one-to-many morphofunctional relationship).

As for most of those species already having been discussed in Rovinsky et al (2021), maybe a more direct sign-posting should be included (e.g. "see Rovinsky et al (2021) for an in-depth discussion of X and Y species' morphology compared with the thylacine's"). While I praise the authors for the broad-picture scope of this study, combining findings from different disciplines, I feel that the fact that this manuscript and Rovinsky et al (2021) are companion pieces and should be read together, is not stressed enough. I read Rovinsky et al (2021) again right before tackling this review and it added great context to this revised manuscript.

Why is our emphasis on the wolf-thylacine comparison?

The authors claim that they keep the focus on the wolf because of (1) high public profile (do they mean popularity?) and (2) existing literature plus popular science. I read the rebuttal before the manuscript and my first thought was that this read like a cop-out at best, or an attempt to be "media-worthy" at worst. Given my previous concerns regarding a certain narrative being pushed, it's fair to say that I started this review with quite an antagonistic mindset. Luckily, although the authors brush over the changes made to the manuscript to reduce the focus on the wolf in their rebuttal, those are significant and have truly changed my opinion on the manuscript. However, at a time when scientific research is often misconstrued by the public, lack of scientific rigor is often used as an excuse to cut funding (e.g. Argentinian government on CONICET), and the press is only interested in fluff science with clickbait-y results, I believe that we should refrain from letting high public profiles and popular science interfere with how we approach research. Please note that this is not a criticism to the authors, but a reflection on how scientific publications seem to be changing agendas from sound research to popular appeal and this is starting to seep into how we design or justify our research.

Back to the original question though, why keep the focus on the wolf, please refer to my comments on the discrepancy between Figure 2 and Suppl. Table 1. I believe in most cases the manuscript should shift to a comparison with wolf-like canids (i.e., the Subtribe Canina) instead of hammering on the uniqueness of grey wolfs and dingoes. In my opinion, this would increase the relevance of the findings presented here, and inspire new research avenues (for instance, looking at the cranial development of other Canina).

(Remarks on code availability)

I have only reviewed bits of the code, particularly regarding some of the functions used. However, this quick glance suggested that the code as presented here will be a very helpful resource for the community.

Reviewer #4

(Remarks to the Author)

I co-reviewed this manuscript with one of the reviewers who provided the listed reports. This is part of the Nature Communications initiative to facilitate training in peer review and to provide appropriate recognition for Early Career Researchers who co-review manuscripts.

(Remarks on code availability)

Reviewer #5

(Remarks to the Author)

Overall Impressions

This study is a relatively standard, comparative, shape analysis of mammal crania, with the aim of interrogating the nature and degree of similarity in shape between the extinct thylacine and members of the Canidae clade of placental mammals. Other carnivoran mammals, including both terrestrial and aquatic taxa, along with additional metatherian taxa were also parts of the sample. I am in favor of the manuscript being published, but only if the authors (1) conduct a new statistical analysis that other reviewers have also suggested, (2) declare a competing interest on behalf of author AJP, and (3) generally clarify their grammar and caveat their narrative with alternate hypotheses. Because these modifications are non-trivial, I would classify them as major revisions, but they may be relatively straightforward given that the authors have all the data necessary to conduct the new statistical analysis.

The study breaks down shape roughly into rostral and caudal "modules" (colloquially speaking), though the authors compare two schemes of such modules, one based on cranial functon(rostrum, braincase) and one based on developmental origin (i.e., tissue type; 2 neural-crest modules and 1 mesoderm module). The results show that the thylacine most resembles large-headed canids like the gray wolf or dingo in terms of the braincase module, whereas it most resembles smaller-headed canids like certain foxes in terms of the rostrum module.

Generally, the methods are appropriate to address the central question: how does the shape of the thylacine cranium compare with other carnivorous mammals? However, I agree with another reviewer that the methods are not adequate to determine whether the sampled metatherians and placentals have different allometric trends in cranial form. This question is one that authors start to address using linear morphometrics of size (visualized in Figure 4), but they have not yet conducted relatively standard analyses that would explicitly assess the presence and nature of allometric differences between lineages. I cite a couple of studies that used such analyses in my line-by-line comments. Authors should employ these

methods for this purpose, as differing allometric trajectories between marsupials and placentals could impact the interpretation of their other results.

I also share a reviewer's concern about an overemphasized narrative of an "extinct predatory behavior". Authors claim that the thylacine was both exceptionally "unique" and yet also part of a grouping of extinct carnivorous mammals that all share some cranial traits, ergo they must share predatory behavior. Neither of these narratives are well supported by the results. There are several canids that resemble the thylacine in both rostrum and braincase (across species within module); among these species are the maned wolf, culpeo, and coyote. It is apparent from the results that there are allometric differences in certain linear dimensions (see Fig. 4) among lineages, but these differences could be put in a different light pending an explicit analysis of shape allometry by lineage. The PC-by-PC analysis is not sufficient.

This narrative with respect to the "terminal rosette" is also not well supported. Authors point out that the thylacine has this rosette at the rostral tip of the rostrum, resembling skull morphology of some reptilian semiaquatic predators as well as some extinct terrestrial mammals. Authors suggest that the thylacine's narrow snout coupled with the rosette means that it likely used a fast, snapping bite to acquire small prey items, and they also suggest that extinct mammalian carnivores with similar morphology shared this behavior. However, authors write as though this behavior, a "fast-snapping" bite, is extinct and has been overlooked because the last paragon of it, the thylacine, has no living analogs. However, authors themselves identify several modern canids that prey on small, quick vertebrates. These canids have crania that closely resemble the thylacine's. The authors carried out no video or biomechanical analysis to support their speculations about bite speed or strength. I don't expect the authors to perform these kinds of studies, as they seem beyond the scope of what the authors set out to do, but if it is the case that biomechanics and kinematics are beyond the scope here, then authors should put greater weight on caveats than they currently do (e.g., alternate hypotheses, discussed below).

Methodological Approach

This study is a standard morphometric analysis using a respectable comparative sample. The authors estimate growth trajectories for several dimensions of the cranium to assess the presence and nature of differences among a handful of canid lineages and the thylacine, and they use a geometric morphometric analysis to interrogate similarity to the thylacine's cranial shape among the wider sample. Using Procrustes distances to assess similarity is reasonable, but it is worth explicitly stating that similar taxa will be, in most cases, distinct along different axes of shape variation (as another reviewer pointed out). Also, the Wheatsheaf index (a measure of strength of convergence) can be calculated with Procrustes distances, and that metric explicitly includes phylogeny (Arbuckle, 2014). Why not employ that index instead of the raw Procrustes distances?

The allometric regressions that use individual PC axes and centroid size are perfectly fine in and of themselves for visualizing differences among groups, but these analyses are not sufficient to statistically tease apart differences in shape allometry among lineages. As suggested by another reviewer, the ideal way to statistically test for such differences is to explicitly model the allometric relationship by lineage, as done in several papers by Polly, Adams, etc. In my line-by-line comments, I cite a couple of example studies that use such an approach. It seems like all the data necessary for such an analysis is present in this study, so the authors ought to take that step.

Formatting and Clarity of Writing

Formatting is generally fine, but the clarity of writing needs some work. This text suffers from many ambiguous ("hanging") demonstratives — "this", "these", "they", "it" and similar — that are unaccompanied by a subject. Authors should modify these sentences for clarity.

Also, the narrative that the authors settle on, that the thylacine's traits mean that there is a lost opportunity to shed light on some kind of totally unique behavior, doesn't make a whole lot of sense and isn't well supported by the data. I discuss this issue both above in my general impressions and below in my line-by-line comments, so I don't want to repeat too much. The bottom line is that if authors wish to speculate that the thylacine was particularly well adapted for a behavior, that seems reasonable, but they should emphasize that the point is speculative absent biomechanical studies that test the hypothesis. They should also discuss alternative hypotheses. In spinosaurid dinosaurs, the terminal rosette has mostly been hypothesized to function as an adaptation for more surface area to better hold on to prey. Likewise, in modern crocodylians, any species can produce the same bite force regardless of skull shape (proportionate with body size; Erickson et al., 2014), so the rosette may have more to do with hanging on to slippery prey than withstanding greater stress at the tip of the rostrum.

Ethical Considerations

The authors ought to declare a competing interest on behalf of author AJP. In this revision, the authors explicitly bring up ethics of researching the thylacine, given its historical and cultural relevance to the Indigenous communities of Tasmania. I would recommend the authors highlight their commitments in an Ethics/Inclusion Statement, which Nature Communications has room for, rather than in the main text (hopefully such a statement would get a separate hyperlink from the main text?). That said, why not start now, while this manuscript is still in revision? Author AJP has a professional relationship with a scientific-and-investment endeavor that explicitly aims to clone the thylacine. That company, Colossal, states that author AJP's lab is leading their efforts to clone the animal. This manuscript currently makes the argument that the thylacine is more "unique" and a greater "loss to diversity" than previously appreciated, a narrative that could reasonably be interpreted by readers to be a bulwark of support for cloning the thylacine. These factors combine to create the appearance of an ethical conflict, and it would be a positive step toward the commitment to be transparent about the professional relationship with Colossal.

Line-by-line comments:

36: missing a space and comma after "observation"

39: "…as it reflects"

"it" might refer to "the thylacine", "the thylacine's ecology", "comparative studies", or the "cranium". I think the "it" is supposed

to refer to “comparative studies”, but the meaning is ambiguous given the disagreement in count.

44-46: “…although marsupials display general trait suites…”
I don’t understand how the “although” clause relates to the rest of the sentence. Do the authors mean that “general trait suites” are shared across all mammals or that these suites are particular to marsupials? Also, the “although” clause more appropriately would contrast the remainder of the sentence, but it seems like the final clause (“their cranial…”) reflects back to the although clause. The first clause is then a sentence fragment and nonsensical: “Similarity between wolves and thylacines is especially notable because.” I recommend rephrasing. “The similarity of wolf and thylacine is noteworthy partially because marsupial and eutherian morphology is so different: marsupials and placentals share some traits that relate to broad-scale dietary regimes (e.g., crushing molars vs. carnassial teeth), but a marsupial cranium is nonetheless usually statistically distinguishable from a eutherian cranium,” or something like it.

49: “The radical departure towards…” (emphasis mine)
Treating “radical departure” as the subject of the sentence means that it is unclear whether the thylacine, marsupials, carnivorans, or some combination of the above is meant to be the subject of this clause. The authors have not established that the cranial anatomy of the thylacine is “radically” departed from anything. The previous paragraph simply notes that the wolf and thylacine are similar and that the similarity is noteworthy. The previous paragraph also states that marsupial and placental crania are nonetheless statistically distinct, so is the thylacine an exception to that previous finding by other authors?

52: “Excitingly…”
I recommend the authors drop this kind of independent marker word throughout (also “intriguingly”, “interestingly”, etc.). If the sentence describes something exciting, then readers do not need to be told that they should be excited. Dropping these unnecessary words will reduce word count.

55: “If these…”
“These” what? Could be “aspects of thylacine cranial growth”, “shared regulatory regions”, “paraxial mesoderm tissues”, etc.

56: “…they would provide…evolutionary, genomic, and developmental processes…”
But this study does not examine genomic or developmental processes. It does examine developmental cranial modules, I suppose, but not “processes”. I recommend rephrasing about expectations for morphology.

59-60: “…the overall cranial similarity…morpho-functional detail.”
This sentence is essentially redundant with the following sentence but is less clear (“translat[ing] well into morpho-functional detail” is awkwardly phrased). I recommend removing this sentence to save words.

61: “significantly converging”
What does “significantly” mean in this context? Statistical significance? If so, why not just say “converging”, cite the study, and save the word?

70: “distantly-related…sparassodonts”
But sparassodonts are metatherians, yes (Forasiepi, 2009)? I don’t think that phylogenetic relationship is ever stated in the manuscript. 70 MY is certainly still a long time, but doesn’t the shorter divergence time increase the chance that similarities are due to shared ancestry? Also, the hyphen between “distantly” and “related” is unnecessary because “distantly” is an adverb.

72-73: “This all suggests…analogue for the thylacine.”
I recommend that authors rephrase the beginning of this sentence and turn it into an introduction for the paragraph. It is a good thesis statement.

75: “clearly convergent”
Do the authors mean “superficially” convergent? Isn’t the point that we don’t know how convergent they are?

75-76: “querying individual patterns of similarity in isolation will not yield a useful perspective.”
A couple of things here: one, what is an “individual pattern of similarity in isolation”? Do the authors mean a one-taxon-to-one-taxon comparison, or do they mean a single anatomical system, or something else? Second, are the authors saying that no valid conclusions can be drawn from this “individual pattern of similarity” (whatever it is), or only that any conclusions risk being limited in utility or specious? Those two interpretations are distinct, and I read the current incarnation of the clause as intending the former (i.e., no valid conclusions), which I would imagine the readership might push back on. The Figueirido and Janis papers cited in this sentence aren’t that unlike the present study. Perhaps their conclusions are specious, but if so, the same risk must be considered for the present study.

76: “They also hinder”
“they” what? Thylacines? Individual patterns of similarity? Convergent analogues?

83: “This shows”
“This” could refer to either the preceding sentence in its entirety or to “large infraorbital foramina”, which is the noun directly preceding “this”.

91-92: “Our…analyses show…placentals and metatherians….”
I recommend rewording this sentence because the thylacine may have a unique combination of certain traits, but the results do show resemblance of the thylacine cranium to those of mid-sized eu- and small metatherians. That finding does not sound the same as “incongru[ity]” with the two clades.

99-100: “The aspect…variation.”
This sentence is a little hard to read, and I think it could be improved by simplifying to something like, “The thylacine resembles canids in having low scores on PC2.”

102-103: “…clearly important…visual impression suggests.”
Important how? I think the authors are trying to say that the separation is statistically “real” but exists on a PC axis that explains less variation than PC1. But readers could interpret the authors as trying to say that there is some kind of biological importance at play that isn’t stated. Also unclear is whether the “visual impression” is of the crania themselves or of datapoints on a scatterplot. Anyway, if there’s overlap between canids, thylacine, and some metatherians on PC1, how can the thylacine’s morphology be “incongruous” with both groups? There is something about the word choice that sounds like the discussion tries to have it both ways, i.e., the thylacine is both totally unique from but also relatively similar to extant mammals.

105-106: “Thus…along PC1.”
I don’t really understand this sentence. If PC1 separates (roughly) dolicho- from bradycephalic crania (lines 96-97), then I don’t understand how the thylacine and Nimbacinus are “unique” along PC1. There are several datapoints near those two on PC1. Couldn’t one pick any two datapoints and say they’re “unique” from the others? It seems reasonable to say that the thylacine’s face is on one extreme of PC1, like some canids, but saying that it is “unique” seems meaningless in this context. If authors are simply saying that canids and the thylacine are “unique” from short-faced species, wasn’t that point already made in line 97? If I’m missing something, please clarify.

107-109: “…showing low distances of both canids and marsupial (sic) from the thylacine…smaller marsupial quolls.”
Did the authors mean high distances? Don’t low distances indicate similarity? In any case, the circles in Fig. 2A don’t exhibit any kind of jumpiness or grouping; they gradually increase in distance from Nimbacinus to Didelphis. Again, if I’m missing something, please clarify, because I do not understand how these figures support “uniqueness” of the thylacine any more than they support uniqueness of, say, the Virginia opossum.

110-111: “Separating…neurocranium.”
Doesn’t this sentence, which identifies similarity with both canids and metatherians, contradict the current introductory sentence of the previous paragraph?

135: “the concept of a one-sided convergence”
What is a “one-sided convergence”? Are the authors saying that other works suggested that the wolf/dog cranium experienced evolutionary stasis and the thylacine’s lineage converged on that same shape? Authors need to cite some background here.

153-165: allometric effects discussion
I agree with the reviewer who raised concerns that authors should, if desiring to test differences in allometry among lineages, explicitly test that question. The individual PC-axes-versus-size regressions should not be relied upon as sole arbiters of allometric differences among clades, whether qualitatively or quantitatively, as such plots of form space may be misleading. Instead, an explicit regression of multivariate shape versus size, followed by significance testing for differences among lineage-specific regressions, would be the statistically appropriate workflow (see Adams & Nistri, 2010; Cardini & Polly, 2013).

207-208: “suggests that there might be”, “between where”, the description of a trade-off
“suggests that” already indicates uncertainty. Can save words by removing “there might be”. “Between where” is a bit awkward; consider “whereby” or “wherein”.
“gracility is maximised relative to a constant absolute bite force” does not sound like a trade-off (it sounds like something has been maximized regardless of another trait). Authors explain the trade-off by (and in) the concluding sentence of the paragraph, but the current intro sentence isn’t very clear. I’d also suggest that authors illustrate the trade-off, either with a figure of crania that are examples of the extreme ends of the trade-off or by briefly stating examples of such taxa here.

210-211: “Gracility and large…functionality.”
This sentence is a bit tautological. A similar point is more clearly made later (“The thylacine cranium is thus…). I recommend removing or rewording this sentence.

225: “extremely pronounced”
Redundant; if the trait is pronounced relative to the entire rest of the sample, there’s no need to say “extremely”.

227: “Biomechanically, this moves”
The preceding sentence talks about how pronounced the trait is in the thylacine, so “this” could refer to either the general trait or to the state in the thylacine.

227-231: discussion of terminal rosette, resilience, and bite force

Has this idea been tested? If it has, a citation to the study should be here. If not, a citation to a reference text would be helpful. In spinosaurid dinosaurs, a terminal rosette was interpreted as an aid to holding food items.

232-234: “absolutely tall compared to canids”, citations for this sentence
I don’t understand what absolute height adds to the discussion here. Isn’t the thylacine cranium absolutely larger in all dimensions than most canid crania? Is absolute height what matters for dispersing dorsoventral forces, or is it relative height?
Also, the citations in this sentence focus on the other side of the amniote tree (specifically archosaurs), where skulls are built quite differently. Is the height rule generalized across amniotes (and/or tetrapods)?

246-273: colloquy about ecomorphological (intertaxon) comparisons with the thylacine
I agree with the reviewer who raised concerns about the “extinct predatory behavior narrative”.
How fast is a fast bite, and how similar does a mammalian terrestrial predator need to be for “useful” ecological or functional analogy? Entelodonts were generally much larger (at least an order of magnitude heavier) than the thylacine, are presumed to have been omnivorous, and in some cases preyed on sheep-sized camelids (Joeckel, 1990; Sundell, 1999; Rivals et al., 2023). Long snouted entelodonts may have had a quick bite, as Sundell suggested in their abstract, but it’s not like large canids don’t have a qualitatively quick bite (see, e.g., video of dog in Brassard et al., 2020). Similar story with mesonychids (size of large wolves or small bears, ~40-60 kg; Solé et al., 2017). Analogies with modern canids are arguably more useful because we can observe and quantify their behavior more easily than for an extinct species. Besides, the current study did not quantitatively assess cranial shape of entelodonts nor biomechanically test skulls to determine robustness to stress.
Bottom line, the point that the community should only approach analogizing the thylacine with modern canids cautiously is a valuable point. Likewise, discussion about aquatic animals with terminal rosettes is interesting, and it seems reasonable for authors to name-drop a few specific extinct taxa (not entire lineages) that look like the thylacine. But the results show that analogy between any two taxa will be imperfect. The suggestion of an “extinct predatory behavior” common to all these lineages is, at best, not based sufficiently in the study’s findings.

280 and 282: “However” repeated in back-to-back sentences, sounds awkward

274-287: discussion about size of infraorbital foramen
A couple of concerns/questions here. Authors begin by a priori calling this trait “important” and “unusual”, words that imply biological relevance, but all readers see is that the thylacine was a large-bodied marsupial with a relatively large infraorbital foramen (IOF). There is no explicit test of allometric slopes by lineages, so we don’t know whether the size is a dasyuromorph thing.
Second, there are other interpretations that ought to be discussed. The infraorbital nerve is not the only structure transmitted by IOF; the infraorbital artery and vein also traverse IOF and might be a root cause of the large size. Even if large IOF relates to the nerve, the citations about vibrissae anatomy are a bit dated (Lyne reference needs title and year details). Has there been modern redescription of thylacine vibrissae? Overeruption of canines is also present in Smilodon (Riviere & Wheeler, 2005) and modern big cats (Smuts et al., 1978). The two placental datapoints in Figure 6 of the present study that are located near coordinates (5.25,2.4) are lion and tiger. If over-eruption covaries with size of infraorbital foramina, then large IOF of lion and tiger suggest that the link is not novel to thylacine and sparassodonts. How tusks and soft-tissue structures of the rostrum fit into this discussion is also worth considering.
Bottom line, this section would benefit from explicit analysis of allometry by lineage and discussion of alternate hypotheses.

295: “maxillary branch of the maxillary nerve”
?? “of the trigeminal nerve”?

293: “emergent property of a trait combination with no…equivalent”
I don’t see how the thylacine can have striking cranial convergence with the wolf but also have a combination of traits with no modern analog. The phrase sounds self-contradictory. I don’t have specific suggestions, but this sentence could use work.

295-298: “far greater loss to global biodiversity”
These sentences are a little unfocused and could use work. Are authors suggesting that the thylacine was a keystone species?
On the question of functional equivalents, authors say we “lost an opportunity” to shed light on the thylacine, but aren’t authors doing just that with this submission? What about biomechanical modeling, CT analysis, soft-tissue inference? Are authors saying that the community can’t learn about or from the thylacine absent a perfect extant analog? The results show that the thylacine and certain canids are convergent. Perfect convergence in continuous traits is a mathematical ideal that no two taxa can ever meet (Stayton, 2015). Authors themselves drew parallels in this submission with modern crocodylians and extinct dinosaurs, members of an entirely different amniote lineage.
I’d like to hear the authors explain what they mean here, because it is demonstrably false that there is no longer an opportunity to understand this phenotype.

299-308: first colloquy about developmental evolution
Given that citation #51 did not even study the body part that the present study interrogates (they studied mandibles, not crania), I’d like to see a little more context in the sentence. It is not that surprising to find that the thylacine is not a perfect analog for species from a group that isn’t especially speciose. It should be harder to match the thylacine’s head with a carnivoran (~300 species of which authors sampled 69), than to match, say, a merlin’s head with a passerine (6000+ species) — there still isn’t a great match even with some strikingly convergent heads (e.g., shrikes).

A full treatment of developmental constraints should include discussion of conflicting results. Some studies suggest developmental constraints on marsupial skeletal form (e.g., Sears, 2007; Kelly et al., 2019). There is no support for the claim that the thylacine is viewed as an "imperfect carnivoran". Developmental constraints do not automatically equal evolutionary disadvantage; morphological integration, one example/manifestation of constraint, can facilitate exploration of phenotype space in some directions while it limits exploration in others (Felice et al., 2018).
Ultimately, I recommend rewording 303-304 to something more like "our findings are consistent with our hypothesis that the thylacine used quick, hard bites to subdue small prey items," adding "vice versa" to the end of the sentence in 305-306 ("For example, it is plausible…"), and generally refocusing this part of the discussion on development rather than the "evolutionary disadvantage/imperfect carnivoran" narrative.

310-312: "The extensive…selection has targeted similar genes…(e.g., the posterior cranial shape) or not." (emphasis mine)
Are authors saying that the genotype is somehow "seen" by selection? How do authors imagine that these similar regulatory regions evolved? Are they trying to point to pleiotropic effects? Or are authors merely stating that thylacines and wolves share more genes than just those linked to head anatomy?
Lines 314-316 sound like the exact opposite of the sentence in 310-312. (i.e., Similar regulatory regions, in affecting early patterning genes in a similar population of cells, result in similar phenotypes in both thylacine and wolf.) What is authors' scenario for how these regulatory regions became involved in this region of the head?

323-329: conclusion paragraph
Unnecessary "at" in the first sentence, hanging demonstrative "this" in the second and third sentences, and beginning of fourth sentence is awkward. This submission did not look at genetic basis of trait evolution, so I would remove that phrase from this paragraph or else rephrase it to make clearer that molecular work is a future direction.

330-341: extended ethical discussion
Are authors planning to publish a popular version of this discussion alongside this submission? This section is a good reminder to the evolutionary morphology community, but it is buried at the end of a long, data-heavy discourse on anatomy. It would be valuable for this kind of commitment to be made more approachable to members of the public, as in a concurrent editorial/letter to the editor. Alternately, Nature Communications has space for an "Inclusion and Ethics" statement that is separate from the main text, so this paragraph could be better highlighted in that space.
That said, the authors should declare a competing interest. Given that author AJP has been leading a lab aiming to clone the thylacine for multiple years — and is deriving media attention from those efforts and through being a scientific advisor to the company (Colossal Biosciences) aiming at de-extinction of this taxon — I am surprised that no competing interests were declared. A major narrative of this manuscript is that the thylacine is especially "unique". This narrative, including that the thylacine is a "greater loss to global biodiversity" than previously realized, could be viewed by readers as an attempt to draw attention to the value of cloning the animal. Transparently declaring a competing interest would address the appearance of an ethical dilemma.

353-357: on re- or retrodeformation
Are the steps taken to re-deform specimens recorded somehow within the software, and could they be included with the submission? These steps constitute hypotheses of anatomy upon which, to at least some extent, the results turn. Likewise, are the paired Morpho landmarks included, or could they be? I may have missed these data when I went through the GitFront folders, but I did not see a folder named "Morpho".

358-363: on the Andrewsarchus artist impression
If the teeth weren't relevant for landmarking, why reconstruct them at all, rather than spending that time and energy on the rest of the cranium? Did the paleoartist start from scratch, or did they modify another animal's digitized cranium? These details are relevant for reproducibility.

386-388: shape analysis and allometry
See earlier comments about testing allometry by lineage.

435: "functionalities of…evomap"
Is there a specific command or section of the code?

443-444: "are known to have extreme IOF sizes"
This claim should be cited, and I recommend rephrasing. "Extreme" is ambiguous and could be interpreted as either very large, very small, or both, so it's better to be explicit than use that term. "Because canid IOF size varies widely," "Because some canids have relatively large IOF," or something like one of these.

Figures and Tables
The figures and tables are generally effective. The authors chose a color palette and general visual approach to the figures that seem broadly accessible. In Figure 4, the colors for the dingo, Canis latrans, and Lupulella adusta are somewhat similar under certain color-deficient proofing schemes. I suggest employing differently shaped data points for each species in that figure.
In Figure 6, the arrowed lead lines are somewhat helpful, but there are other data points on this graph whose taxonomic identity would be valuable for readers to know because those taxa share traits with the thylacine but are not currently discussed (or are only briefly mentioned). The top-right datapoints, particularly the lion, tiger, and at least one pinniped (perhaps the walrus?), would be valuable to label, as would one or two of the smaller-bodied marsupials that have relatively large infraorbital foramina.

Literature Cited

Adams DC, Nistri A. 2010. Ontogenetic convergence and evolution of foot morphology in European cave salamanders (Family: Plethodontidae). BMC Evolutionary Biology 10(1):216. https://doi.org/10.1186/1471-2148-10-216
Arbuckle, K., Bennett, C.M. and Speed, M.P. (2014), A simple measure of the strength of convergent evolution. Methods Ecol Evol, 5: 685-693. https://doi.org/10.1111/2041-210X.12195
Brassard C, Marilaine Merlin, Claude Guintard, Elodie Monchâtre-Leroy, Jacques Barrat, Nathalie Bausmayer, Stéphane Bausmayer, Adrien Bausmayer, Michel Beyer, André Varlet, Céline Houssin, Cécile Callou, Raphaël Cornette, Anthony Herrel; Bite force and its relationship to jaw shape in domestic dogs. J Exp Biol 15 August 2020; 223 (16): jeb224352. doi: https://doi.org/10.1242/jeb.224352
Cardini, A., Polly, P. Larger mammals have longer faces because of size-related constraints on skull form. Nat Commun 4, 2458 (2013). https://doi.org/10.1038/ncomms3458
Erickson G. M., P. M. Gignac, A. K. Lappin, K. A. Vliet, J. D. Brueggen, G. J. W. Webb. 2014. A comparative analysis of ontogenetic bite-force scaling among Crocodylia. Journal of Zoology 292(1):48-55. https://doi.org/10.1111/jzo.12081
Felice RN, M Randau, A Goswami. 2018. A fly in a tube: Macroevolutionary expectations for integrated phenotypes. Evolution 72(12):2580–2594. https://doi.org/10.1111/evo.13608
Forasiepi AM. 2009. Osteology of Arctodictis sinclairi (Mammalia, Metatheria, Sparassodonta) and phylogeny of Cenozoic metatherian carnivores from South America. Monografías del Museo Argentino de Ciencias Naturales 6:1-174.
Goswami A, Marcela Randau, P. David Polly, Vera Weisbecker, C. Verity Bennett, Lionel Hautier, Marcelo R. Sánchez-Villagra, Do Developmental Constraints and High Integration Limit the Evolution of the Marsupial Oral Apparatus?, Integrative and Comparative Biology, Volume 56, Issue 3, September 2016, Pages 404–415, https://doi.org/10.1093/icb/icw039
Joeckel RM. A functional interpretation of the masticatory system and paleoecology of entelodonts. Paleobiology. 1990;16(4):459-482. doi:10.1017/S0094837300010198
Kelly E M, J D Marcot, L Selwood, K E Sears, The Development of Integration in Marsupial and Placental Limbs, Integrative Organismal Biology, Volume 1, Issue 1, 2019, oby013, https://doi.org/10.1093/iob/oby013
Rivals F, Belyaev RI, Basova VB, Prilepskaya NE. Hogs, hippos or bears? Paleodiet of European Oligocene anthracotheres and entelodonts. Palaeogeography, Palaeoclimatology, Palaeoecology. 2023;611:111363. doi:10.1016/j.palaeo.2022.111363
Riviere, H.L. and Wheeler, H.T. (2005), Cementum on Smilodon sabers†. Anat. Rec., 285A: 634-642. https://doi.org/10.1002/ar.a.20199
Sears KE. 2007. Constraints on the Morphological Evolution of Marsupial Shoulder Girdles. Evolution, 58(10), 2004, pp. 2353–2370. https://doi.org/10.1111/j.0014-3820.2004.tb01609.x
Smuts G. L., J. L. Anderson, J. C. Austin. 1978. Age determination of the African lion (Panthera leo). J Zoology 185(1):1-146. doi:10.1111/j.1469-7998.1978.tb03317.x
Solé, F., Godinot, M., Laurent, Y. et al. The European Mesonychid Mammals: Phylogeny, Ecology, Biogeography, and Biochronology. J Mammal Evol 25, 339–379 (2018). https://doi.org/10.1007/s10914-016-9371-8
Stayton CT. The definition, recognition, and interpretation of convergent evolution, and two new measures for quantifying and assessing the significance of convergence, Evolution, Volume 69, Issue 8, 1 August 2015, Pages 2140–2153, https://doi.org/10.1111/evo.12729
Sundell KA. Taphonomy of a Multiple Poebrotherium Kill Site—An Archaeotherium Meat Cache. Journal of Vertebrate Paleontology. 1999;19(sup003):79A. doi:10.1080/02724634.1999.10011202

(Remarks on code availability)
The R code that I reviewed (on GitFront) looks reasonably well annotated and appears to work.

Version 2:

Reviewer comments:

Reviewer #1

(Remarks to the Author)
I have now carefully reviewed this new version of the manuscript. However, the main, conceptual, issues that I noted since the first version are still there. Hence, I am not going into a point by point reply, as at this stage it is no longer necessary.
To make it short, the authors do not have enough results/data to support the very big claims presented in the manuscript. Furthermore, too many doors remain open suggesting that adding more in deep analyses would have been beneficial.
The reworked abstract epitomises these issues. The authors did not provide any biomechanical analysis (it would have been possible even with linear measurements) despite claiming that the thylacine has “substantially different biomechanical properties”. Here, trying to understand why the thylacine was having such a big head and big bite when it is widely assessed that its skull was poorly adapted to support a strong bite, would have provided a solid ground on which build a robust inference about the thylacine uniqueness. Again, providing a more complex adaptive scenario (supported by data) could have been great, rather than using a mere proxy.
Also, in Figure 3a other species display large residuals, with two marsupials showing the largest. Is this an emergent property that can be observed in other taxa? Is it shared with placentals (different canids show large residuals)? Or is it unique to thylacine? Here, having a larger (and new) sample could have been helpful.
In the abstract (and in the title) it is stated that the thylacine was having a fast, high-impact bite. But no data support it. Here, you could have simply measured the jaws mechanical advantage (a couple of linear measurements as noted above) and

compare it (within a phylogenetic context) with other species to understand whether the thylacine really stand out or not. This has been done by multiple authors in multiple venues. Again, the data provided are not sufficient.
The last sentences are not appropriate, you do not have genetic data or performed genomic analyses.
Again, the data and the methodological framework presented here are too thin to provide a meaningful advancement of our knowledge of this extinct species.
I am sorry I cannot be more positive, but I hope that the questions presented above could serve the authors to provide a better scientific design and to stress out their conclusions.

Kind regards.

(Remarks on code availability)

Reviewer #3

(Remarks to the Author)
In this second revision the authors have implemented new analyses (allometric comparisons) and visualizations (phylogenetic visualizations of Procrustes distances) following the comments of other reviewers, and extensively rewritten the manuscript to incorporate those new results and address several concerns raised in the previous iteration.
I was already satisfied with the previous iteration (barring some minor comments) and the current version is even better. I am particularly happy with the new discussion around canids in general (and Canina in particular) instead of focusing on the grey wolf. I'm glad that some of my suggestions were useful for framing this new narrative. I also find Suppl. Figures 3 and 4 much easier to interpret than Figure 2 + Suppl. Table 1 (plus they're gorgeous), so I wanted to thank the authors for providing both types of visualizations.
However, I feel that some of the other additions might be problematic, particularly after reading the comments of R#1 and R#5 to the previous iteration. Except for point (1) in the list below, I don't think any changes are needed on the other topics, but I do believe they're worth mentioning.
1. Allometry
The additions to the Expanded Results add more depth to the study, so they are very welcomed. However, while the authors added them to address reviewer concerns about not incorporating size into the comparisons, the way that they are handled compounds on the issue instead of solving it. I have made several comments on the Supplementary Data file (please check attached file with tracked changes), but my main concerns are repeated here:
- the regression without Andrewsarchus is not "nearly non-significant". On the contrary, it's a better fit than the regression including that species (as shown in Extended Results Table 1). This comment was ignored on the previous iteration and needs fixing.
- the interpretation of the ANOVA tables on Extended Results Table 2 makes no sense. Why would a significant interaction term negate differences in clade means when the clade term is also significant? Each significant model term can and should be interpreted on its own, as it explains a fraction of sample variation.
- the previous point bleeds into the follow-up analyses, as the authors only test for differences in clade means when the interaction term is not significant, and when it is they only test for differences in slopes. Differences in clade means should be tested in all partitions, as the clade term is significant in all of them. Furthermore, the authors should specify whether they are testing for differences in slope magnitude (amount of changes per unit size) or slope orientation (whether shape changes occur in different regions of the shape space and thus represent different changes), as the pairwise() function allows for both tests. Based on the authors providing distance values, I would assume that they're testing for differences in magnitude (I haven't checked the script for this, sorry), but it needs to be clarified. Please note that the differences in slope orientation are likely what R#1 was referring to when suggesting that the authors could "run separate pooled regressions (e.g. separating Canidae, Felidae, etc.) and test whether these resulting slopes were convergent, divergent or parallel."
- "Plotting the regression scores (i.e. the part of the variation related to allometry) of the Procrustes regressions against centroid size illustrates the lack of allometric signal in the similarities between thylacines relative to canids (and vice versa) in the Principal Components Analysis and Procrustes distances of the main mansucript." I don't understand what the authors mean here. If all the models in the Extended Results section have a significant size effect, why mention here a lack of allometric signal? This needs to be corrected or better explained.
2. Colossal Biosciences
Now that R#5 has brought up AJP's connection to Colossal Biosciences, I can see why he raised issues with it, as knowledge of that fact adds negative connotations to some of the paragraphs in this manuscript (which suddenly feel as a potential publicity pamphlet for that company), particularly after the alleged prosecution by Colossal Biosciences of researchers actively vocal against their claims of de-extinction. However, the thing is that such companies and certain elements of the public are not going to stop using fragmentary pieces of written material to back up their claims, regardless of whether they actually do back them within their original context. With that in mind, while I appreciate the conflict-of-interest waiver for that author, in my opinion it will act less as an ethics statement and more like a potentially harmful statement towards the validity of this study. The Editor might consider removing it.
3. Final paragraph
Personally, at first I saw this paragraph as the disclaimer that I hear in every conference presentation from Australian researchers, so I mostly ignored it. I did so on the basis that most times I've heard it, it sounded very void, so I treated it as a forced acknowledgement. But in this new iteration the authors make clear (at least to me), that they do care about the ongoing mistreatment of Indigenous peoples by colonizers, which I admire. I am not sure that a manuscript with a hard word limit is the best platform for this statement, but I will support them in doing so (unlike other reviewers, it seems). Well done.
However, one thing I don't like about the statement is that, by focusing on the harm being done to the peoples, it reads as if

the fauna and flora "collected" were just cultural pieces to exploit (akin to the British Museum's – and others – pillages) instead of living units of biodiversity and pieces of an ecosystem that was damaged (sometimes irrevocably) in the process. To me, it perpetuates the idea that fauna and flora within the borders of a Country belong to its inhabitants instead of being their responsibility. In a manuscript about a species driven to extinction by colonizers, maybe that should also be a big point in the closing paragraph. Just my thoughts though, and please take this in the positive – this paragraph made me reflect on the situation, which I believe is its main goal. Again, good job.
Beyond commenting on these topics and the annotated Supplementary Data file, I have added listed line-by-line typos and such at the end of this document.
Finally, to reiterate, once the points mentioned in (1) are addressed, and the minor comments and typos reviewed, I would consider the manuscript ready to go. I do not request any action on points (2) and (3) besides maybe read the authors' thoughts on them, continuing with keeping the dialogue open. And on that note, nothing more to say here in reply to that section of the rebuttal except that I really enjoyed this dialogue, as I think both the authors and I have reached a "common" understanding of these topics. Quotation marks because our opinions might still not be the same, but I believe we both arrived at a sense of understanding with each other's opinions.
I wish that this was more the norm in manuscripts reviews with multiple revisions and not an unusual outlier. Thanks for letting me be part of this journey, and again congratulations on a job well done.
Eloy Gálvez-López (anonymity waived)

L55: I think "of" is missing after "perception".
L68-69: From this sentence I gather that dasyurids do not hold their prey until it's dead as felids do? If that's the case, maybe "penetrate-and-release bites" would be a better description (relative to the degree of detail given for canids and felids)?
L71: Delete extra space before the reference in "canids 15".
L107: "are" instead of "is"? The subject of this sentence is unclear (the plots? the whole previous phrase?).
L135: Delete "three" or add "genera" to "these three canids", as there are more than three species in the Lycaon/Cuon/Canis lineage.
L144-145: Capitalize "developmental" after "b," to match format after "a,".
L172-175: Capitalize "phylogenetic" after "a," to match format after "b,".
L191-194: Are they OLS regressions or RMA regressions?
L192: I think "width" is missing after "carnassial". Or better, write "width across carnassials" instead.
L270-271: Verb missing after "This"?
L272: B in "Fig. 3B" should not be capitalized to match the rest of the manuscript.
L284: "Common Opossum" should not be capitalized to match other species names in the manuscript.
L291: Double space after "vertebrates'"?
L329: Period missing after "craniofacial evolution71".
L375: Delete extra dot after "Fig".
L387: I don't think "arbitrarily" is an adequate qualifier on how PCA axes of variation are calculated. I would recommend deleting that word.
L402-404: I have to agree with other reviewers that choosing just 10 PCs and regressing them separately against size is not best practice, and the authors should have analyzed simultaneously all the PCs in each partition.
From the script I gather that all the pgls regressions of PC1 to PC10 vs logCS of all partitions are carried out separately in a loop and then packaged into a summary table for inspection, so I assume not checking all the PCs is just a matter of how gigantic that table would be.
As a simpler alternative, I would recommend using a single pgls model of all PCs vs logCS and then using eta_squared() from the effectsize package to obtain η2 values and 95% confidence intervals for each PC in that model. From that function's output it's very easy to check which PCs are significantly related to size (which PC has CI_low > 0.00) and how big the effect is. Please note that I have only used this with the lm() function, so it might not work with model fits from pgls(). It should, but I haven't tested it recently.
L404: First comma should be a period.
L405-406: Fix "be should" typo.
L406-407: This sentence doesn't make much sense.
L427-428: I would use "canid species" instead of "species of canid", as it's shorter and reads easier (at least to me as an ESL person). Fix separation between "canid" and colon.
L477: Fix spacing issues in "Sparassodonta(e.g., 101)."

(Remarks on code availability)
I only checked a few snippets of the code, but they were clear, easy to follow, and the functions used were sound.

Reviewer #5

(Remarks to the Author)
Overall impressions:
I remain in favor of publishing the manuscript. The authors improved the clarity of their narrative. They addressed the concerns of five independent reviewers over multiple rounds of revision, including by the addition of new statistical analyses and a competing interest declaration. I particularly thank the authors for addressing my line-by-line comments, which I recognize were extensive. The responses were thoughtful and earnest, and where the authors departed from me, broadly speaking they had reasonable explanations (e.g., first-principles biomechanics) or there was a misunderstanding on my part. I find this version of the manuscript improved, and though I don't agree in every instance with the authors' interpretation of the results, the interpretations generally stand well on the analyses. I believe my comments below constitute suggestions and minor revisions to clarify points.

Methods:
I appreciate that the authors both implemented the pooled analysis of allometry and experimented with using a Wheatsheaf(-like) Index (WI), both of which took time and energy. I accept that it doesn't seem necessary to include the WI results in the main text, but if the journal allows it, I'd encourage the authors to include the WI work as supporting information.

Results + Discussion:
The thing that I am a little unclear about is the authors' interpretation of the results from the new pooled allometric analyses. The discussion of those results in the extended data seems opposite, or at least inconsistent, with the discussion of the results in the main text. It looks right that clade-specific mean shape is influential. But doesn't statistical significance of the interaction effect between clade and size nonetheless indicate that there are independent allometric slopes by clade (i.e., marsupials vs. caniforms vs. placentals)? And the extended data makes it sound like that result makes the influence of clade-mean shape alone hard to interpret. Can't the thylacine's head morphology be explained, at least in part, by a marsupial-specific allometric trend or trends? If not, why not? I didn't expect a common allometric pattern to the whole sample (or to all partitions) to explain the thylacine's morphology, but I did wonder whether the thylacine is sortuva scaled-up quoll in one or more regions of the head, which the presence of clade-specific allometry might bear out (but if so, would presumably add to the idea of thylacine-as-mosaic).

Part of the problem is that I'm having trouble visualizing what the clade-specific allometry looks like at the extremes. I think I follow authors' logic that because the RegScore is small for thylacine, there is something or some things apomorphic about the thylacine beyond marsupial head-shape allometry that make them look like wolves. But the regression-fit plots in extended data Figure 4 sure look to me like the thylacinidae group's cloud (albeit just the two datapoints) parallels the best-fit lines. In other words, the fit still improves at the same rate as everybody else as size increases. Could the separation in regression fit simply be due to the thylacine group's apomorphically large heads?

Since the interaction terms seem to be influential, at least to some degree, spline deformations could help readers like me visualize exactly what the marsupial allometric extremes are. Did authors visualize splines from the extremes of the pooled regressions? I'd recommend it, if not. They probably would help some readers visualize the strength of the various influences on shape. Given that authors make the case that the thylacine is a mosaic shaped by these influences, visualizing the mosaic could be worthwhile. **I know that authors are limited on space, and they've already done a lot of work, so again I'm not going to demand these visualizations or anything, but I would ask them to talk a bit more about it in the extended results.**

I do want to offer my apologies that, for me, this time through is just my second view of the manuscript, because I know there have been rounds prior to my entering the picture as a reviewer. That said, thanks to the authors for hearing my concerns.

Formatting and flow notes:
The narrative does make more sense, and I do better understand what the authors are trying to say. I am still concerned that some readers are going to take home the "extinct predatory behavior" thing, rather than "the thylacine was a mosaic and this combination of traits doesn't clearly exist in the modern mammalian biota", but the authors basically wrote the latter into this draft, so the only thing I would recommend on this point at this stage is to review the language again and make sure it's how they want the text to sound.

I will say that there are some writing-style tendencies by the authors, e.g., in my first line-by-line comment on this go-round, that make sentences really confusing for me, personally, as a reader. In most of these instances I think there would both be a way to reword the sentence to be clearer and save words. I want to be sensitive to authors' voice in this manuscript, because my intention is not to take away their voice(s), but sometimes the text just sounds like it contradicts itself, or the dependent clause at the beginning of sentence is so long that I get lost on how it relates to the independent clause once I reach it after the comma. My recommendation in any review when I encounter this kind of issue is just to make sentences shorter and simpler. I'll point out a few places, and I recommend the authors go through the manuscript with a fine-toothed comb to find others.

Line-by-line comments:
58: "has provided an ideal potential example"
Confusing to read "has provided" but then also "potential". Either is or it isn't an example of something. If "potential example", then it's not an example yet and therefore hasn't been provided. Recommend dropping "Owing to this" and rewording to be more active, like "Comparisons of thylacine and canid cranial morphology could shed light on the genetic and developmental mechanisms of adaptive evolution in mammals." Saves 4-5 words over what's there but still communicates uncertainty. Not everybody likes the "shed light on" phrasing, and that's fine, but I encourage authors to play around with the wording.

138: I think there's a missing word after "genomic".

152: "significant allometry exists"
Since authors have the results tabled elsewhere for most if not all analyses, I encourage dropping the word "significant" in most cases. The term "significant" carries a lot of baggage (see, e.g., Martínez-Abraín, 2008; Dushoff et al., 2019) and can distract. Either there is clear evidence of an allometric relationship (with a p-value), or there isn't. Additional nuance can then be discussed after. I'm not gonna push the point any farther if the authors feel strongly, but replacing the word "significant"

with either explicit reference to a p-value or to a table with statistical results generally is gonna save characters and potentially words.

155-160: about the new analyses
I don't entirely understand the point here. The first sentence in these lines could use some simplification. Neither the intro clause nor the parentheses are necessary. It could just read something like, "Shape-size allometry differs among feliforms, caniforms, and marsupials for most skull regions in both partitioning schemes, except Neurocranium and MES (table citation)." That saves 9 words over the first half of the sentence. But I don't really follow the point of the second half of the sentence. Doesn't the statistical significance of the interaction term mean that there are clade-specific allometries that do in fact explain the distribution of species in morphospace, at least in part? What does the influence of the interaction term for marsupials look like at the extremes of that term for each partition? Does it resemble the thylacine at all, or is it wholly different? These details aren't in the extended results, but they are relevant if the case is that the thylacine is a mosaic. I also question whether "overwhelmed" is the right term later in these lines. It's currently hard to visualize what influence the interaction term has. Maybe the mean shape is an exaggeration of the clade-specific allometry, or maybe the influence on shape is totally different. I must apologize that I have not been able to test this question myself by running all of the code, but at a bare minimum I do recommend that the authors talk in the extended results about what the influence of the clade-specific allometry looks like. If they don't want to visualize splines, I won't push that specific issue, but discussing the influence, given that it does reach a threshold of statistical significance, would be worthwhile.

168: "Their cranial size"
I think "their" means "thylacine" but the animals immediately preceding "their" are "jackals and foxes" and the grammatical subject of the preceding clause was "most placental carnivores". Please find other instances like this one and clarify the subject of the sentence.

183-185: subject-adjective confusion
Subject of this sentence is "length", so parallel construction of phrase after comma reads like length is "narrow". I expect authors mean that "rostrum" is narrow, but the sentence needs to be rephrased somehow for "rostrum" to be the subject, else it sounds awkward. My recommendation is to just break up the sentence into two: one about length and one about breadth. On the result itself, is the narrowness relative or absolute? I expect relative, since length was relative, but it's not clear. As in previous round of review, please look over use of words like "particularly". Saying that the rostrum is narrower than almost all other species already means that it's "particularly" narrow (and is more precise), so my recommendation is just drop the adverb to save the space.

225: "thickened rostrum"
"Thickened" sounds like the bone is thicker or more robust somehow. Recommend "broader" or "wider" if authors talking about width.

227: "are narrow"
So I think I get it, but in 225 the rostra were "thickened". If authors mean the rosette in 225, recommend rewording to make that clearer.

also 227: "rarely represented"
Redundant, just say "rare".

229: "sacrificing performance"
"Performance" a little vague. Recommend "stability" or "resistance" if that's what authors mean.

232: "unusually powerful"
Unusual relative to what? Recommend removing the parens preceding these words and rephrasing sentence more like "...consistent with a relatively fast, powerful, 'snapping' bite used to acquire smaller, agile prey."

242-257: IOF discussion
This paragraph still feels overly speculative to me, but I do have a new recommendation to address that concern. I absolutely appreciate the authors including the references I recommended in addition to their thoughtful responses to my concerns. I am, though, disappointed that authors did not mention vasculature. Perhaps it seems like a ticky-tacky concern, but there are structures other than a nerve passing through IOF; nerve, artery, and maybe vein (and probably lymph). Muchlinski (2008) studied the soft tissue directly, and that study might be a better citation in this location than the 2010 pub. That being said, the data in Muchlinski (2008) sure look like carnivorans have relatively small infraorbital nerves for the foramen (the study did not explicitly run ANCOVA for any groups other than primates). If thylacine converged on carnivorans morphogenetically **and** vibrissae were only modest, perhaps infraorbital artery was more pronounced in thylacine. I recommend that the authors reframe this paragraph in a more explicitly inferential framework, e.g., the inverted pyramid and extant phylogenetic bracket (Witmer, 1995). **If authors think that this approach will not be helpful, I won't press the issue, but I hope that they will consider it seriously.**

Figure 6: really appreciate the authors making the changes here. They help me, and the number of labeled taxa look about right. It's not too busy.

280: "does not coincide with the dorso-ventral impact resistance"
I don't understand. Are authors saying that C. simensis has low impact resistance? If so, that probably needs a citation. Or

are you just saying that the skull isn't tall? Again, if there are two points, my recommendation is to break them up into two simpler sentences.

305-308: constraint, contingency discussion
I appreciate that the authors discussed Felice et al. (2018).

310-311: "living animals that likely show major differences"
From whom? Unclear

323: "whose"
Imprecise. I know space is limited but "the maxillary branch of which" is only two more words and is more precise. Save words elsewhere.

326-330: from "potentially" to "craniofacial evolution"
This section needs some work, it's a bit tautological. Also, two uses of "unusual" in the same phrase, which sounds awkward. In next sentence, "It is thus possible...", the invocation of cranial neural crest is overly vague. What does "facilitated from evolution within cranial neural crest cells" mean? The population of cells? Traits of the individual cells? Temporal or spatial shifts? It's like...the FNP is made of neural crest, so of course the CNCC evolved. Had to if the FNP looks different. The question is how (and how much), not whether. How do we get from a large infraorbital nerve to SHH to dental stem cells to the CNCC, and how might these things have evolved in the thylacine? I don't think there has to be a ton more text here, but if the hypothesis is that a large infraorbital nerve would lead to the evolution of a terminal rosette — or whatever is meant here — the logic needs to be a bit clearer.

Lit Cited:
Camp, A.L., Konow, N. and Sanford, C.P.J. (2009), Functional morphology and biomechanics of the tongue-bite apparatus in salmonid and osteoglossomorph fishes. Journal of Anatomy, 214: 717-728. https://doi.org/10.1111/j.1469-7580.2009.01056.x

Dushoff J, Kain MP, Bolker BM. I can see clearly now: Reinterpreting statistical significance. Methods Ecol Evol. 2019; 10: 756–759. https://doi.org/10.1111/2041-210X.13159

Evans, A.J., Naylor, E.R., Lujan, N.K., Kawano, S.M. & Hernandez, L. (2024) Deploy the proboscis!: Functional morphology and kinematics of a novel form of extreme jaw protrusion in the hingemouth, Phractolaemus ansorgii (Gonorynchiformes). Journal of Anatomy, 244, 929–942. Available from: https://doi.org/10.1111/joa.14020

Alejandro Martínez-Abraín. 2008. Statistical significance and biological relevance: A call for a more cautious interpretation of results in ecology. Acta Oecologica. Volume 34, Issue 1, July–August 2008, Pages 9-11

Muchlinski, M.N. (2008), The Relationship Between the Infraorbital Foramen, Infraorbital Nerve, and Maxillary Mechanoreception: Implications for Interpreting the Paleoecology of Fossil Mammals Based on Infraorbital Foramen Size. Anat Rec, 291: 1221-1226. https://doi.org/10.1002/ar.20742

Witmer LM. 1995. The Extant Phylogenetic Bracket and the Importance of Reconstructing Soft Tissues in Fossils. in Thomason, J.J. (ed). Functional Morphology in Vertebrate Paleontology. New York. Cambridge University Press. pp: 19–33.

(Remarks on code availability)
The code still seems reasonably well-annotated. I appreciate the humor written into some of the annotations.

Open Access This Peer Review File is licensed under a Creative Commons Attribution 4.0 International License, which permits use, sharing, adaptation, distribution and reproduction in any medium or format, as long as you give appropriate credit to the original author(s) and the source, provide a link to the Creative Commons license, and indicate if changes were made.
In cases where reviewers are anonymous, credit should be given to 'Anonymous Referee' and the source.
The images or other third party material in this Peer Review File are included in the article's Creative Commons license, unless indicated otherwise in a credit line to the material. If material is not included in the article's Creative Commons license and your intended use is not permitted by statutory regulation or exceeds the permitted use, you will need to obtain permission directly from the copyright holder.
To view a copy of this license, visit https://creativecommons.org/licenses/by/4.0/

<u>**Summary of response and changes**</u>

We thank the four reviewers for the considerable time they have taken with our manuscript. Their comments have been extremely useful and we have now extensively re-written the manuscript to be much clearer. However, the key points in our study – and the core analyses – remain identical, with some additions thanks to the reviewer comments.

All figures have been re-worked extensively, and in one case (Fig. 5) substantially expanded, according to the reviewer comments. We added two new analyses:

1) **An added analysis of allometry, now covering all partitions' overall shape and their first ten PCA axis scores (new Table 1, Extended Results). While this has not changed our interpretation of our results, it provides a useful perspective on the diversity of allometric trajectories within our sample.**
2) **A new phylogenetic generalised least squares regression and plot to show that the thylacine's "terminal rosette" is not just uniquely wide, but also uniquely high.**

R1's main concern was that they believed that we were trying to do tests for convergence, or that we should have done them. However, as we outline, these tests have already been done and no further information can be gained from these. An excellent example for the success of our basic trait-by-trait approach is that traditional convergence tests would have never picked up the disjunct between the thylacine's cranial size and its shape – this has been hiding in plain sight. Traditional, single-modality convergence testing simply will pick this up.

R2 and 4's comments were largely about methodological detail, and we have addressed all of these.

R3's comments were excellent and gave us the opportunity to fundamentally clarify our manuscript. The substantial issue here was that R3 believed that we were claiming that the thylacine was piscivorous. However, as we hopefully managed to clarify, this was not at all our intention and we have added several safeguards into the paper to this effect.

R3's other main criticisms were about whether we under-discussed potential ecological correlates of the thylacine's cranial function aside from the wolf. As with convergence tests, we believe that this has already been done exhaustively by previous work and will not yield further insights. Our focus on the wolf also remains, albeit toned-down, because the wolf remains the key reference point in public and scientific discourse about the thylacine; wolves are also best-researched in terms of potential developmental and genetic similarities with the thylacine.

Following discussions with Indigenous Australians in light of increasing awareness that museum specimens were non-consensually taken from Indigenous land, we also added a section to this effect at the end of the discussion. We expect that there will soon be a new code of ethics in Australia (e.g. https://aiatsis.gov.au/research/ethical-research/code-ethics) that will (rightfully) prescribe collaboration with Indigenous Australians on museum specimens, and hope to be on the front foot and reduce offense by adding this section.

Reviewer #1:

This manuscript deals with the longstanding topic of convergence between marsupials and placentals. Specifically, the authors aim at describing the extent of convergence between the iconic, and recently extinct, marsupial wolf Thylacinus cynocephalus with other placental carnivores.

We are afraid that the reviewer misinterpreted the intent of this paper, partially because we did not use the term "convergence" as rigorously as we should have; but also because R1 seems to have thought that we were attempting tests for convergence, which we demonstrably did not set out to do. We mentioned the term in the introduction as part of framing the research background, but not with view to our analyses. Admittedly, we did this without the specification of what convergence we mean (this is now fixed, p. 3, l. 49-51) but this oversight was not an integral part of how the introduction was framed. Our statement of intent was clear and did not suggest that we were testing for convergence:

"*We here revisit the Thylacine's cranial function by surveying its shape compared to living and some extinct carnivorous mammals through multiple lines of geometric and linear morphometric evidence*."

Unfortunately, this fundamental misunderstanding has caused nearly all of the concerns R1 had with our manuscript. However, the question of convergence and formal statistical tests of convergence as suggested by Reviewer 1 have already been done by co-author Rovinsky et al. (2021) on the dataset that we are using for our shape analyses (Figs. 1 and 2), and are of no further interest (see below for details). We have now expanded on the existence of these previous tests in the introduction as well.

Because clarity was clearly an issue, we have fundamentally re-written the manuscript (see tracked changes document), including an explicit articulation of the way that we deal – or rather, do not deal – with hypotheses of convergence, which we show are not helpful in the context of investigating the thylacine's functional ecology (see particularly the last two paragraphs of the introduction).

The premise of our paper was instead that it is *not* meaningful or interesting to test hypotheses of convergence in light of evidence that there are no good functional or ecological conclusions to be had. We had already outlined this in the last two paragraphs of the introduction; however, we see now that this argument was not articulated very clearly and apologise for the confusion this has caused. We have added a paragraph that explains and justifies our approach to the end of the introduction.

The topic is certainly of interest as evolutionary convergence has fascinated biologists, zoologists and palaeontologists for decades. Nonetheless, the authors decomposed the cranial shape into developmental and functional modules, which is something I found enticing.

However, the study presents several conceptual and methodological limits that I am listing below.

1) Despite the presence of an abundant literature on convergence, it was surprising that the authors did not provide a meaningful definition of it. This is far from trivial as convergence can be defined in multiple ways (see Stayton, 2015a).

As R1 suggests, "convergence" can be a loaded term and we were amiss in not thinking of of defining it (but have done so now). However, our manuscript was always unambiguous in its aim to more deeply investigate the pattern of morphological similarities between placentals and metatherians, regardless of how they arose in terms of ancestor-descendant relationships or type of convergence. As already noted, we made no claims or tests about the origins canid-thylacine similarities, merely characterising the much-discussed developmental and functional hypotheses to explain it. We could have hardly written the manuscript without a mention of these, although at no stage did we suggest that we were testing them.

Without a clear definition of what you are trying to demonstrate (or test), it is hard to contextualize the present study. In fact, it is not clear whether the authors aim at showing the thylacine convergence with canids or its uniqueness.

We were not driven by any aim to show a particular conclusion and feel that this is not a good approach for a scientific study. But we also do not feel that the key result of this paper – that the thylacine is highly unique despite all the known significant convergence – was unclear. We foreshadowed this clearly in the abstract ("The thylacine's convergence with canids thus emerges from a unique, now-extinct trait combination similar to many other large extinct predators"), our introduction summary ("This reveals a trait combination unmatched among living mammals…"), and throughout the discussion.

Also, it is not clear whether convergence should be achieved via developmental, functional processes. This confusion is widespread throughout the manuscript as both processes are invoked to occur, suggesting that pattern-based (e.g. the independent acquisition of similar characters) and process-based (e.g. when a specific process is demonstrated a priori) definitions have been conflated here (see Stayton, 2015b). This is also evident as the scenario (or the pattern) under which thylacines and canids should produce similar phenotypes is never mentioned. This had notable consequences on the methodological framework chosen to identify and quantify the extent of convergence (see Grossnickle et al., 2024).

This was an extremely useful comment, because it made us realise that we were not clear on how our study demonstrates that there is no way (and no point trying!) to separate these two because we are only beginning to understand the *patterns* of similarity between the thylacine and other mammals, let alone the *processes* leading to them. The introduction now makes this clear (2nd to last paragraph of introduction), and we pick this up again in the discussion.

As we now outline in greater detail throughout the ms, our results highlight the difficulty in teasing apart the effects of development and function because these are infinite possible combinations and far too little understanding of how these two might interact. For example, as we now discuss on p. 16, middle paragraph, specific phenotypes might be more likely to arise under natural selection on function in either placentals or marsupials because of their well-known developmental differences. In our case, our results are more consistent with a mostly function-driven process of cranial shape evolution in the dasyuromorphian lineage leading to the thylacine. However, that does not rule out a role of development in providing a canalising effect deeper in the tree. But this is such a vague (if common!) hypothesis that testing for development vs. function is not appropriate without the assessments of patterns which we tackle in this ms.

2) I am sorry, but the methods are not adequate to test for convergence.

As above, we did not test for convergence and did not claim to do so.

First, the extent of convergence must be quantified under a phylogenetic scenario, otherwise it would be impossible to understand if two taxa, independently, evolved toward a similar phenotype. This underlies that the direction of evolution (e.g. ancestor-descendant pathway) plays a central role in determining and quantifying the extent of convergent regimes (see Castiglione et al., 2019).

With ~160 million years evolutionary divergence and distinct differences between placentals and metatherians, of which the thylacine is the only exception in aspects of its shape, there is no need for testing if these differences arose independently! In addition, this has been done before e.g. in Rovinsky et al. 2021. We are now clearer that this is a reasonable assumption in the introduction (2nd paragraph).

The more relevant question is about whether the cranial function of the thylacine is truly canid-like, which has too often been merely discussed in a simplistic "is it more like a wolf?" context (R1 in fact calls the thylacine a "marsupial wolf", which is typical of the pervasive nature of this issue). By contrast, our study shows that previous studies that focused on the degree of convergence between wolves and thylacines have overlooked several crucial functional aspects that make the thylacine highly unique among mammals.

In this scenario, the use of Procrustes distance does not yield the results described by the authors.

This is not correct. The results we described match exactly the Procrustes distance comparison.

Basically, you measured the distance between landmarks configurations and a reference. Here, two points can very occupy the extremes of the space, such as +1 and -1, though being equidistant from 0 (the reference). Although, the shapes at +1 and -1 will be very different from each other, and from the reference as well. This means that using Procrustes distance is not only silent about the ancestor-descendant directions (see also other comments below), but also silent about the phylogenetic history of the focal clades, basically impeding any evaluation of convergence. This was somewhat surprising as there are currently multiple efficient strategies to measure the extent of convergence with highly multivariate data (see Castiglione et al. 2019; Grossnickle et al., 2024), or with univariate data (see Ingram and Mahler, 2013; Arbuckle et al., 2014).

We did not, as R1 suggests, use Procrustes distance comparisons as a naïve and phylogenetically uninformed way of testing convergence. And, as outlined above, there is no relevance to include ancestor-descendant relationships because it is clear that any similarities between the thylacine and canids are not based on a common ancestral condition. Instead, our study goes back to the very basic but also deeply fundamental question of asking which animal the thylacine resembles most in different sections of its cranium, based on previous results (Rovinsky et al. 2021) that similarities between canids and thylacines are not consistent with any carnivoran ecomorph.

Furthermore, R1 is not correct in suggesting that a shape space derived from Procrustes space should be more informative than Procrustes distances (but note that we did provide

both metrics and they were in good agreement, compare Figs. 1 and 2). This has two reasons:

1) Procrustes space is multidimensional and therefore has no univariate extremes that could be allocated a sign (+1 to -1), as R1 suggests. What R1 may be thinking of is the use of ordination (e.g. Principal Components (PC) Analysis) to boil down the main variation to the more convenient – and signed – axes. This allows a decision on what is "extreme", but this PC space is just a derivative of Procrustes space and not more realistic just because it is signed! Moreover, PC space only captures a partial subset of overall variation, and contains no information as to whether this variation is all related to a particular underlying process. As we emphasised in the material and methods section, this is why we opted for the whole-shape comparison of Procrustes distances on top of the more conventional ordinations. See Weisbecker et al. 2019 for more in-depth discussions of this issue and the elaboration on the advantages in the use of Procrustes space.

2) R1: "two points can very occupy the extremes of the space, such as +1 and -1, though being equidistant from 0 (the reference). Although, the shapes at +1 and -1 will be very different from each other, and from the reference as well.":

Regardless of how one summarises a multidimensional space, the issue will always be that one can get similar – even near-identical – summary scores based on divergent underlying landmark configurations. This is because there are so many variables contributing to whichever simplifying summary is chosen; if two areas in a configuration contribute equally to a Procrustes distance or a PC, two species that are extreme in just one of these areas will still come out with similar scores.

The fallacy of assuming that similar ordination scores must mean similar shapes is also the reason why our approach of dissecting the cranium into different regions is so powerful: it demonstrated that both in ordinated PC *and* Procrustes space, the shape of the thylacine holds a biologically meaningless position in the middle between canids and metatherians because it has a canid-like snout and a metatherian-like back of the skull. The PCA spaces provide a nice visual for this, but their partitioning of the variation misses crucial details (e.g. that *Nimbacinus* is overall closest to the thylacine in all configurations, despite its considerable distance from the thylacine in the PCA).

We have expanded slightly on PCA vs. Procrustes distance comparisons in the methods section (P.18, "Cranial Shape")

In summary, I consider this study too immature to fully evaluate the extent of a complex evolutionary phenomenon such as convergence. Hence, I am sorry, but I cannot recommend this manuscript for publication. However, I hope that my comments and listed papers could serve as a guidance to thoroughly explore such an enticing topic.

We hope that our responses support our case that there has been a fundamental misunderstanding of what we set out to do and what R1 thought we set out to do.

Below are listed also other minor comments on the manuscript structure, that deserve attention from the authors.

Stayton, C. T. (2015a). The definition, recognition, and interpretation of convergent evolution, and two new measures for quantifying and assessing the significance of convergence. Evolution, 69(8), 2140-2153.

Stayton, C. T. (2015b). What does convergent evolution mean? The interpretation of convergence and its implications in the search for limits to evolution. Interface focus, 5(6), 20150039.

Grossnickle, D. M., Brightly, W. H., Weaver, L. N., Stanchak, K. E., Roston, R. A., Pevsner, S. K., ... & Law, C. J. (2024). Challenges and advances in measuring phenotypic convergence. Evolution, qpae081.

Castiglione, S., Serio, C., Tamagnini, D., Melchionna, M., Mondanaro, A., Di Febbraro, M., ... & Raia, P. (2019). A new, fast method to search for morphological convergence with shape data. PloS one, 14(12), e0226949.

Ingram, T., & Mahler, D. L. (2013). SURFACE: detecting convergent evolution from comparative data by fitting Ornstein-Uhlenbeck models with stepwise Akaike Information Criterion. Methods in ecology and evolution, 4(5), 416-425.

Arbuckle, K., Bennett, C. M., & Speed, M. P. (2014). A simple measure of the strength of convergent evolution. Methods in Ecology and Evolution, 5(7), 685-693.

Other minor points

Abstract

Line 34 remove repetitions.

Done

Line 38 remove full stops.

Done

Introduction

Lines 40-42 I am not happy with this sentence. Multiple marsupial carnivores display morpho-functional convergence with other placental carnivores. Having differently shaped marsupials (koalas and kangaroos) does not hint to an “unexpected convergence”. I suggest removing this sentence, or to much better frame the issue (or biological bias) of convergence.

Apologies, I can see how we gave the wrong impression here. We do find it important to the less specialised audience to have some reference points about how marsupials and placentals relate to each other. We therefore re-wrote the paragraph to be more balanced and add an acknowledgement that both groups include broadly similar ecological trait suites (such as herbivory/carnivory) (P. 3, middle paragraph).

Lines 44-47 I do not like at all the word “anomaly” here.

Agreed and removed

Lines 51-53 This sentence does not work, please remove it.

This (and R3's) set of comments on the introduction have been extremely useful in helping us clarifying the context of the study. We have now completely re-worked it to better highlight the issue.

Lines 59-61 This sentence is very poorly written. I had to read ref 22 to understand what you mean.

May be something like: "Nonetheless, thylacines display notable similarities with multiple skeletal regions (e.g. teeth, skull and forelimbs) of South-American metatherian sparassodonts [Kealy and Beck 2017]. This also challenges the accurate phylogenetic reconstruction of the metatherian, as elegantly shown in [ref 22]."

Thank you for this suggestion. We agree that this whole section could have been clearer and the issue of phylogenetic reconstruction was also not all that relevant to the flow. We have re-written the section with this feedback in mind.

Results and Discussion

Lines 78-81. How did you test it? Here, you are just looking at two PC axes, this looks too speculative to me.

This regards the sentence "…the extinct mid-Miocene thylacinid *Nimbacinus dicksoni* is closer to the metatherian morphospace in both the total and rostral PC2 (Fig 1 B, C), suggesting that the aspect of the thylacine's cranial shape that makes it similar to placentals might not be an ancestral thylacinid trait.

When you look at PC2 in Fig. 1 B, C (pasted below), you can see that the thylacine is clearly closer to canids (orange circles) than *Nimbacinus* is. We could of course technically run a t-test of the PC 2 scores, but this would be statistical overkill given that *Nimbacinus* PC2 scores are clearly well outside the canid scores whereas the thylacines' are either close to or among the canid PC2 scores.

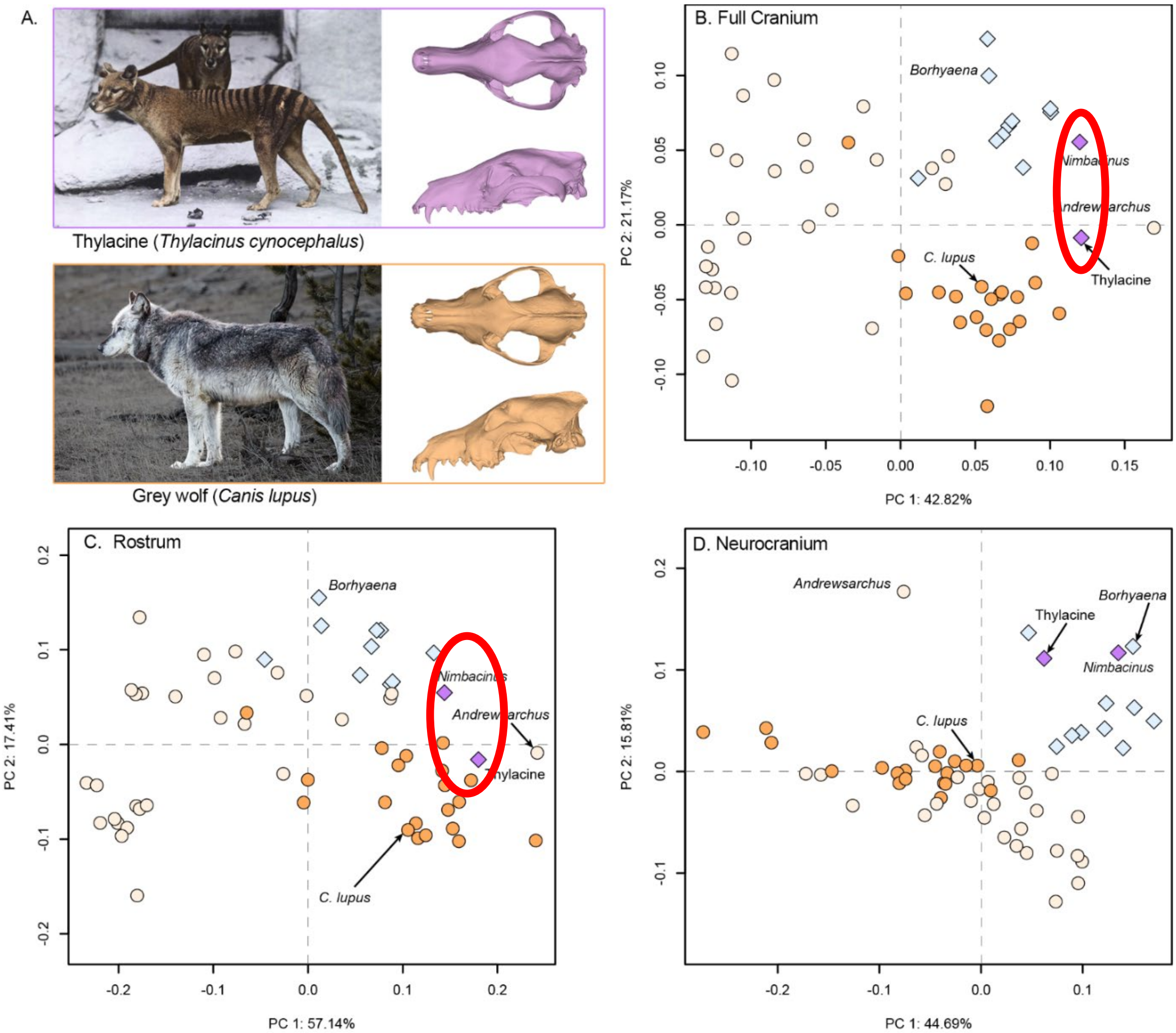

Lines 82-84. Please rephrase. Put it in a more scientific context, begin saying that attributing a clear ecology to thylacines has been quite a task considering that it is not really that similar to any other species.

Done

Line 99. I am not sure what a "marsupial-like pattern of resemblance" is.

This passage was re-phrased

Lines 111-113. Same as above, how did you test it? Again, this is way too speculative without using any phylogenetic comparative method.

This sentence was not a report on a test, but maybe this was not clear enough and we have re-phrased it. We stand by our statement that our result is consistent with an existing hypothesis of a developmental basis for the wolf-thylacine similarity, which was based on developmental results that align with ours. In addition, this sentence was a stepping stone for an important new perspective on this influential hypothesis; which is that any developmental re-arrangements that have been hypothesized for the thylacine would also have to affect wolves for our data to be explained well. To make it clear that we are not

reporting on a test for developmental backgrounds, we have changed the last sentence of this paragraph to read "Thus*, if developmental re-arrangements are the drivers of the similarity between the two*, both wolves *and* thylacines must have diverged from their respective ancestral conditions and evolved partially similar shapes due to re-arrangements of development in the MES and potentially FNP regions."

Also, such putative "departure" could be very well ascribed to within clade allometry.

The departure of the wolf towards the thylacine that we refer to in this sentence is unlikely to be due to allometric effects. This was one of our key points and we continue to argue it because 1) the neurocranium of similarly-sized and larger canids is not as close to the thylacine and 2) the wolf's Procrustes distance relative to the thylacine is similar to that of the much smaller dasyurids in the sample.

However, this comment prompted us to expand the analyses of allometry substantially in our manuscript; we now report allometry of the whole dataset and within the first ten principal components in the extended results, and added a whole new paragraph and outputs on allometry to the ms (P. 9, Table 1). We also have to admit to a mistake in forgetting to re-align our data with the phylogeny after removal of *Andrewsarchus* in the supplementary information, which led to our report that there was no allometry at all in the dataset. This is was not the case; all partitions do show allometry and we have added an evaluation of this effect to the ms and extended result. However, none of these results have changed our overall conclusions.

Figures.

Done

Reviewer #1 (Remarks on code availability):

I accessed the codes. I perfectly understood the methods used and, as detailed in my review, they are not suited to test the proposed hypotheses.

We refer to our previous comments about R1's clear misunderstanding of the purpose of our analyses.

Reviewer #2:

In this paper, Rovinsky et al. utilize geometric morphometrics and linear measurements to examine thylacine cranial morphology in the context of marsupials, canids, and three related fossils. They make functional predictions based on comparison with extant taxa. These results are clearly of interest both to those generally interested in functional morphology and those interested in this captivating animal.

This is a great paper and the manuscript is well written with beautiful visual interpretation of results. That being said, we have a few minor recommendations – NONE of which should be considered "required". If this paper were published as is, it would be an important and worthy contribution. With that said…

The only substantial issue that we struggled with in this paper is understanding the comparative sample and specific data collected. We are confident that we struggled with this because this sample is built on a sample previously described in another paper, but we do not generally consider it good practice to require readers to go to other papers to understand things that are necessary for understanding the current paper. In other words, we think this sample needs to be included in this paper – at least as a supplement – as should be more details about the measures. For instance, if the comparative sample included mostly animals with long pointy faces, then that would mask convergences and emphasize divergence within that morphospace. Does this only include carnivores?

The comparative sample includes 56 species of extant terrestrial faunivorous mammals, covering all major clades including nine carnivoran and two marsupial families: (Canidae, Mephitidae, Mustelidae, Procyonidae, Eupleridae, Felidae, Herpestidae, Hyaenidae, Viverridae, Didelphidae, Dasyuridae). Where possible, preference in sampling was given to more carnivorous members of these groups instead of more insectivorous, omnivorous, or frugivorous members.

Additionally, three extinct carnivorous mammals were included, the mid-Miocene sparassodont Borhyaena tuberata, the mid-Miocene thylacinid Nimbacinus dicksoni, and the stem-whippomorph Andrewsarchus mongoliensis.

Also, we are confused by some of the measures including references to "carnassial". Obviously, these do not exist in thylacines and other marsupials, so a description of this measure and others based on non-homologous structures seems necessary.

It is a common misconception that only carnivorans have carnassials, but this is not correct – our mistake was more in not highlighting this sufficiently. Carnassials are found in many groups of mammals, including Carnivora, Oxyaenidae, Hyaenodonta, Sparassodonta, some (Didelphini) Didelphidae, Dasyuridae, and Thylacinidae.

Carnassials are a set of reduced-complexity sectorial teeth in which at least one (usually the lower) has a carnassial notch at the junction of two cristae. These teeth have reduced their cusp complexity and have hypertrophied cristae, resulting in lesser grinding/crushing function and greater shearing function. With respect to the jaw itself, the lower carnassial is found at or close to 50% of the jaw length from the condyle, and the main resultant force of the jaw adducting muscles occur roughly 60% of the distance from the condyle to the lower carnassial.

Where the confusion comes in is the *specific set of teeth* that form the carnassials. In Carnivora, it is the P4/m1 teeth that always form the carnassial set; this is indeed a synapomorphy of the group. But other lineages form the carnassial set from different teeth; in marsupials it is M3/M4, in oxyaenids it is M1/m2, etc..

For the analyses, measurements were taken at these carnassialised teeth. This is regardless of developmental history – otherwise it would be difficult to standardise measurements (e.g., as marsupials don't have a fourth premolar, and their first lower molar is not functioning as a shearing tooth). This is now clarified in the "Linear measurements" section of the methods, and we added a pertinent reference (Werdelin 1986).

Other than that, there are only minor typos that need correcting:

L19: e.g.,

L32: missing space after –

L34: "an important" is repeated

L37: "Recent studies" seems to need some citations.

This is due to the weird double periods typo mentioned directly below; the two sentences should be a single concept followed by the citations currently present. Thanks!

L38: Doubled periods

In the first paragraph of the Results and Discussion, we feel it would be of value to contextualize generally what morphology is driving differences PC1 and PC2.

We agree, and a passage has been added to this first paragraph

L82: "resembles a both"

Fig. 1 needs an explanation of the color/symbol code. Seems to be same as subsequent figures but needs to be explained at least in this first use if not repeatedly.

This was a mistake in the upload; has been fixed.

Fig. 3: The canids seem to all have positive residuals, right? A significance statement about that would be valuable.

We agree; a product of the long-snouted nature of the skulls. This has been done.

L155: again, we are confused about marsupial carnassials.

See above comment.

Fig. 4: This is a really beautiful and informative figure. No problems, just kudos!

L182: is "<<" intentional?

Yes, it signifies "much less than". However, you are right that it is rather obtuse and perhaps a little non-standard in form, and has been removed.

Fig. 6: what do the hulls signify? They SEEM to be metas vs. placentals, but some of the metatherians are outside of the blue shape. Why?

This was our bad – we had left the extinct large-IOF metatherians out of the hull when we should not have! This is now fixed and the captions explain the hulls.

L300: “subsequent” should be “subsequently.”

This section was deleted.

L352: please cite which literature was used for size proxies

These references are included in the supplemental information, and have been called out to aid their visibility.

L362: are domestic dogs and grey wolves really lumped together in this sample?! Are highly derived dogs included in here? Dingos? From the structure of this writing (i.e., under the phylogeny subheading) it seems that this sensu lato use is only on the phylogenetic analysis, not the morphological one, right? Maybe that needs to be stated directly?

This was indeed was confusing, and really was an ill-fitting leftover from an earlier draft of the manuscript. It (as you point out) doesn’t fit or really make sense in the manuscript in its current form, and has been removed. Where necessary, species’ common names and binomials have been more specifically called out in the manuscript to remove any potential ambiguity.

L385: Eileen Westwig hasn’t been at the AMNH for a LONG time, but if she was there when the data were collected then I guess that should still be the acknowledgment? Maybe “then at the…”?

That is correct (she’s now in Berlin!) and perhaps testimony to the long time that this dataset has been in the making!

Again, this is a great paper and all of these issues are more for the authors to think about or clean up before final proofs. As it is, it is essentially acceptable.

Thank you for those positive and constructive points!

Reviewer #3:

In this manuscript, the authors present a series of arguments suggesting that the thylacine was the last surviving member of a predator group employing fast, high-impact snapping to capture their prey. Surprisingly, they posit that this prey might have been fish instead of small terrestrial vertebrates (as suggested by current hypotheses on thylacine dietary preferences and hunting habits). I'm afraid I don't share the authors' confidence on the implications of their results, neither think I that enough evidence is provided to support that claim.

The manuscript does not state that the diet of the thylacine may have been fish – we never entertained this notion (otherwise it would have been in the title)! But because this was not on our mind at all, we were probably not clear enough in the section where we outlined the many existing similarities between the thylacine and fish-feeding vertebrates. However, in this paragraph, we also referred to terrestrial vertebrates who have similar morphologies and, stated that piscivorous vertebrates... "are clearly not ecologically analogous to the thylacine, but their cranial proportions are thought to be selected for performance in fast-snapping predation...". This section is now strengthened by adding "As aquatic or aquatic-feeding piscivores, these are clearly not ecologically analogous to the thylacine, which is clearly a terrestrial predator[6,29,46] with no dental adaptations to piscivory[3]"

Overall, I think this is a well-written manuscript presenting novel and interesting results on thylacine (and canid) cranial shape variation, while at the same time linking them to recent studies on cranial development and genomics on those species.

Thank you for this overall appraisal and before delving into the details, we also want to thank you for a thorough review that must have been very time-consuming. We enjoyed addressing the comments and hope that they will be satisfactory, once the basic misunderstanding that we are pushing a piscivorous narrative has been dealt with.

My major concern is that the authors focus on their narratives (wolf vs thylacine, extinct predator group) so deeply that they seem to disregard any alternative potential explanations to their results. A few examples would be:

- in some cases the results are presented in a way that could bias the reader (e.g. see comments on L73-76 below).

We have gone through the text and re-wrote these sections. However, since this is a combined results/discussion section, we felt justified in drawing conclusions from the results we reported; see responses below.

- most of the Results & Discussion section is focused on the differences and similarities between the thylacine and the wolf (representing large-prey predators), but the same comparison is not made with the small-prey specialists. In fact, any comparison with other large prey specialists, like Cuon or Lycaon is also missing. This could be particularly important when discussing Figure 3A (see comment for L127 below).

Since this comment is crucial for R3's evaluation of the manuscript, and is repeated throughout when we mention a particular species, here is a summary of our rationale behind not discussion individual species more:

Why do we not discuss other species more?

We agree that we could have added more discussion on how the thylacine compares to other canids, and have now added this particularly with reference to the species pointed out by R3 as far as the word count permitted and as far as these had not already been covered by Rovinsky et al. 20201.

However, we maintain our existing (but now clarified) argument that it is unhelpful to try and match the thylacine to any existing canid because it combines so many divergent traits. It shares aspects of many ecologically totally divergent species within our sample and getting bogged down in detail will not help. Examples that R3 mentions below, which we are discussing as one here, are *Canis simensis*, *Chrysocyon* (aspects of rostral shape but not size and known ecology), and a range of small canids in general (aspects of rostral shape but not size); *Lycaon*, *Cuon* (absolute size but not shape and ecology; see also *Borhyaena*); *D. marsupialis* (relative size, neurocranial but not rostral shape). Each of these similarities exists in the context of fundamental differences with the thylacine, and only serve to highlight that the thylacine has a trait combination that is comparable to none of the species. An exhaustive discussion of these (beyond the existing notes of why some comparisons are not suitable) is not within the scope of this manuscript and also unlikely to lead to new insights, particularly because Rovinsky et al. (2021) has already done so in detail. In fact, our argument is that an undue focus on detailed comparisons with living species is what has prevented the recognition of the thylacine as a totally unique species with no living equivalent.

Specifically to Lycaon and Cuon, these have never been posited as similar to thylacines. It probably never crossed anyone's minds to do so as they have completely different (short and stout) cranial shapes and clearly took much larger prey than the thylacine. Given the abovementioned dissection of the thylacines' similarity to specific canids in Rovinsky 2021, we therefore did not feel that much space needed to devoted to these cases.

Why is our emphasis on the wolf-thylacine comparison?

We agree that it was not necessary to focus so extensively on the wolf, and have now toned this down across the ms. However, a sizeable component of wolf-thylacine mentions remains because

1) because it is the most common comparison with a high public profile, but our paper shows that there is no functional reason to consider the two particularly comparable and

2) because the potential convergence between wolf and thylacine is widely discussed, both in the scientific literature an even more so in the popular science realm.

We also prefaced the paper with the observation that the thylacine does not appear to match any existing canid ecomorph, which is something that was already covered by Rovinsky et al. 2021. Thus, going back into the detail of these discussions would duplicate publications and detract from the point that the paper is making – that the whole notion of the thylacine being convergent to wolves (or canids in general) is not a great basis for investigating its ecology.

- despite being most similar when combining cranial shape and size, Chrysocyon is disregarded as a potential ecological equivalent based on its diet (L132-138) but… isn't the whole purpose of this study to try to understand the thylacine diet? Couldn't it also be an omnivorous species

eating both small mammals and fruits? A similar disregard of Canis simensis occurs later in this section (L199-203).

As discussed above, our question was explicitly not about the diet of the thylacine, rather it was about identifying the cranial function that allowed it to survive on its diet. The diet itself is well-established as hypercarnivorous and there is next no chance of it having eaten fruit. We have added more references to support this and made sure that the hypercarnivory of the thylacine is already outlined in the introduction (2nd sentence), with additional comments on the hypercarnivorous dentition in the 2nd-to-last paragraph of the intro.

Thus, I would strongly recommend the authors to tone down this narrative by presenting it as a potential explanation, along with several others, of their results.

For more detailed comments, please refer to the line-by-line section below.

Apologies in advance if some of my comments seem too harsh, that was not my intention. I might have issues with how the results are discussed in the manuscript, but I do not wish to undervalue in any way the work or ethics of the authors.

Not at all! We appreciate the considerable time it would have taken to write these comments and your decision to waive anonymity. Some of the issues were a misunderstanding, and hopefully you will agree how we addressed them. But otherwise, we hope the changes we have made to the analyses and framing are suitable and make the manuscript clearer.

Eloy Gálvez López (anonymity waived)

L2: Author order is different in the manuscript than in the system (emails, reviewer dashboard, etc.). I assume the one in the manuscript is the correct one?

We were aware of this and had noted in the comments section of our submission, but it does not seem to have been actioned. However, note also that we have switched authorship to VW first, DSR last.

L34: Remove repeated “an important”.

Done

L38: Remove repeated period.

Done

L39: This might read as “they’re similar in cranial shape”, as convergent is often used very loosely in the literature. However, since this has been statistically tested in Rovinsky et al (2021), I would recommend rewriting this as “[…] and have been shown to present convergent cranial shapes with canids” (or something similar). This change will also strengthen other instances when convergence in skull shape is brought up (e.g. L72).

Thank you – our loose use of “convergence” was also an issue for R1, who did not see that this had been already tested. We have defined it better in the intro (2nd paragraph) and now

also highlight the Rovinsky (2021) results more. This whole section is now rewritten according to your recommendation and that of R1.

L268-287: I would add "(see below)" before the colon to avoid the reader wondering about the nature and contents for Rovinsky et al (2021)'s sample for two paragraphs. Alternatively, the authors could rewrite this paragraph to incorporate lines 284-287: "For this study, we used Rovinsky et al's cranial dataset of 381 three-dimensional (3D) landmarks (46 fixed [...]) of 225 specimens from 60 mammalian taxa (including 17 thylacine crania; Suppl. Data 1), to which we added three new specimens using the Viewbox 4 (...) protocol established in that study: the Argentinian [...]"

Yes, that makes a lot of sense – re-written now.

Regardless of which approach is selected, please reference Figure 2 on the parentheses describing the landmark configurations. Since there is no proper figure for it in this study (yes, I know it's in Rovinsky et al (2021)), linking to a figure where the configurations are shown would be helpful for those of us who always read the Methods section before the Results.

Thank you, an excellent suggestion. A new figure with detailed landmarking protocol information is now in supplementary Figure 5.

L288: Why are the zygomatic arches not included in either partition? They are a good indicator of masticatory muscle size, and hence have significant biomechanical implications.

This is a very fair comment and we apologise for not including our rationale for excluding them. The reason was that the zygomatic arch functionally overlaps the front and back partition. However, because we agree that this reasoning is a bit complicated, we have replaced our original analyses with an inclusion of the zygomatic arch partition into the neurocranial portion of the skull. This has not resulted in any substantial changes to our ordinated or Procrustes space results.

L294-303: This paragraph is quite convoluted. It took me a few reads to understand that it meant "We visualized shape variation in each partition using PCA (Fig. 2). Similarities in cranial shape were assessed using Procrustes distances, and the closest species to the thylacine are shown in Figure 3." I would recommend expanding that summary as needed, but please keep it simple and direct. Also please mind the references to the figures. As mentioned above, it helps readers who don't read the manuscript following the published section order.

Sorry for the confusion. This first section is now re-written to be clearer and it also outlines the Procrustes distance approach more clearly (see R1 responses).

L306: I would remove the "felids vs. canids" example (and just leave the other one), as the former are only mentioned here and the latter are only compared to thylacinids.

Done, thank you

L307-311: I tried accessing the R script with the links provided but I kept getting errors, hence this comment. Did you use procD.pgls to calculate the size effect on cranial shape or procD.lm (or procD.allometry)? The Extended Results mention Procrustes Generalized Least Squares (which usually refers to procD.lm) for shape instead of Phylogenetic GLS (procD.pgls), so I wonder whether phylogeny was included in the regression or not. If indeed it was, please edit the Extended Results.

Sorry that the code was not accessible. R1 managed to access it, but we have created a zip file of the repository for the revision submission.

L325: Please add "Figure 4" at the end of the parenthesis to guide readers to a visualization of (some of) the measurements taken.

Done

L327-328: Please give species names for all species. Why grey wolf but just "South American canids" or "fox"?

L. adusta – side-striped jackal; L. gymnocercus – Pampas fox (or Azaras' zorro if you want to distinguish South American "foxes" from vulpine foxes); V. vulpes – red fox

Done.

L335: Remove repeated "then".

Done

L340 & Figure 5: Two related comments. First, please link the description to Figure 5 to direct readers to a visual aid ("The rostrum of the thylacine (Figure 5) is [...]"). Second, either replace the drawings in figure 5 by images of the actual cranium (or mesh, ideally with the relevant landmarks) or add those as separate panels for the figure (ideally for all the species highlighted). While the simplified drawings might be very clear to the authors, I'm still not sure that I understand them.

Thank you for this constructive comment. We have extended the figure now (adding another regression and meshes that match the outlines) and believe it is much better than the previous one!

L343-344: You use "prediction range analysis" here but "prediction interval comparisons" later on L356. For consistency, please change this sentence to "As with cranial size, we calculated the 95% prediction intervals of the regression to ask whether [...]".

Done and this sentence is also now slightly re-written to be clearer.

L361: I think there's a double spacing between Nimbacinus and using.

Yes, that's right – fixed now

L361-366: This paragraph is useful when you read the Methods section before Results and Discussion, but probably not in any other situation. I would suggest moving this to the Introduction (maybe between L57-58 when discussing small- vs large-prey specialists).

We had thought about this and had a lot of discussion where this should go! The problem here is that this is a 122-word (or 92, word, if just focussing on the wolf terminology) paragraph and the word count for the introduction is too small for this. Terminological detail like this also distracts from the main narrative of the introduction, but we still felt that a comment needed to be included for methods readers such as yourself. It is therefore probably best off where it currently is.

L73: Here I'd go deeper than "aligning with previous studies". The cranium PCA morphospace is almost identical to that of Rovinsky et al (2021) even after adding the extreme Andrewsarchus,

and leaning into that would prevent readers from wondering whether its inclusion is affecting the direction of PC1 in a similar way than it did on the shape vs CS regressions.

Excellent points! This whole section was re-written to be clearer about how it fits with previous work and what the novelty of this presentation is.

L73-76: For me, this is the first red flag regarding the forced narrative I mentioned above. By saying that PC2 “only” explains 21% of shape variation vs PC1 explaining “over half” of said variation, the authors are planting the notion on the readers that PC2 should be disregarded. First, for a study using 381 3D landmarks, we would expect over 1100 PCs to be generated, which means that by chance alone, each one should explain under 0.1% of shape variation. Thus, 21% of shape variation is not a small amount. Second, according to Figure 1B, PC1 explains 42.82% of shape variation, which is not over half.

A more objective description would be: “However, the thylacine lies just outside of the canid region of the PC1-PC2 morphospace, which represents over 60% of cranial shape variation. While the low scores of the thylacine on PC2 fall close to the canid range (excluding the widely divergent Speothos*), the overall distinctness of the thylacine cranium from both metatherians and placentals is emphasized on PC1. Here, metatherians […]”

Furthermore, this more objective description provides stronger support to using PC2 to illustrate the divergence between Thylacinus and Nimbacinus (L79-80), as its importance has not been minimized previously.

This was indeed not very clear, and we appreciate the very constructive feedback here. We have re-written the section in the spirit of the suggested change, with the small difference that we prefer to highlight how the departure of the thylacine from metatherians occurs on a PC axis that explains less variation than the PC axis on which metatherians and placentals are the same. The reason we find this important is because the PC plots make the thylacine look much more different to metatherians than it actually is, which we believe has led to previewsly exaggerated perceptions of this convergence.

* I assumed the canid on the top left region of Fig. 1B is Speothos due to its unusual morphology, but please correct that if I’m wrong.

That is correct!

L79, L82: To avoid confusion, change to “(Fig. 2A, circles)”.

Done

L82: Please remove “a” before “both”.

This section was re-written

L87: Potential double space after the first period.

This section was re-written

L95-97 (Figure 1): Please add the same legend as in Fig 3, and provide species names on panel 1B for the canid “outliers” (top left and lowest PC2 score).

Done

Also, why is the polarity of PC1 inverted in the corresponding panels of Suppl. Fig. 1?

I presume you mean Supp. Information Fig. 3 where Fig. 1A is replicated - this is flipped because *Andrewsarchus* is not in the sample. As often happens with small changes in datasets, the morphospace is nearly identical but the polarity flips.

L103: Could these distance values be provided with the supplementary material? Since not all canids are represented on Figure 2, is hard to imagine that the canid species with negative PC1 values are closer to the thylacine than the wolf is.

No problem – these are now in Supplementary Table 2

L104-106: These lines could be a great segue to discuss differences and similarities with other large prey-specialists and with small-prey specialists (e.g. the red fox is the closest non-thylacinid in rostrum shape). This would provide a more nuanced interpretation of the results.

See comment at the top of this response for R3

L111-113: While the rest of this paragraph does a good job in linking recent genetic studies with the shape variation described here, I think this sentence pushes the argument too far. This study neither includes any evolutionary modelling, nor presents any morphospace that, once turned into a phylomorphospace, would suggest cranial shape divergence between wolves and other canids. Thus, it needs to be removed.

I understand that the authors might want to make a big statement out of their findings, but this is the kind of sentence that is later referenced out of context and ends up becoming accepted without proper initial support.

We agree that we should not have been so specific with how we expect developmental divergences to have occurred; this was unnecessary and also not the focus of our study. However, the case that wolves and thylacines are both unique in some aspects within their respective radiations, and should be further investigated in terms of their developmental similarities noticed elsewhere, remains and is well-supported by the PC and the Procrustes distance results. We have now separated the two concepts (departure from respective ancestral shapes *versus* speculative potential for developmental convergence) in this paragraph as well, making it clearer and less exaggerated.

L124: The suggestion for L104-106 would also add support to this sentence, as there has been barely no mention of other canids beyond the wolf.

See comment at the top of this response for R3; this section has also been slightly re-written with reference to canids in general.

L126: I think Suppl. Fig. 2 should be replaced by Fig. 3B (thus reducing Fig. 3 to the 3A panel) and a similar plot of the living marsupials presented in the current Suppl. Fig. 2. As it stands, the figure draws more attention on how relatively large the cranium of D. marsupialis is than to the thylacine, so it leaves the reader wondering why is that not discussed.

We find the plot in 3B powerful in demonstrating the previously totally ignored fact that the thylacine's cranial shape is tremendous compared to what its shape would lead us to expect. With regards to the unusually large cranial size of *D. marsupialis*, as noted above, the nature of the thylacine's shape is that it shares aspects of shape with many species; but this does not mean that each of these should be discussed.

L126: Unless I have missed any regional spelling, it's "outsized" not "out-sized".

This was removed in the re-write

L127: Similarly, I would recommend writing “wolf equivalent” instead of “wolf-equivalent”.

Thanks for pointing this out. We changed the wording – it is grammatically not ideal anyway 😊.

In any case, I would remove “Consistent with its history of being considered a wolf equivalent,” altogether, as it doesn’t make sense with the rest of the paragraph. On the contrary though, if the two canids between the thylacine and the wolf in Fig. 3A, which also have particularly large crania relative to their size, are Cuon and Lycaon, that could be interpreted as canid large-prey specialists having wolf-sized crania regardless of body mass, which would then support the claim that the authors are trying to refute. (Based on the following paragraph I assume one of them is Chrysocyon, but what is the other?)

Regarding the canids closest to the thylacine in centroid size: (in order) *C. lupus* & *Chrysocyon* are larger, and the other canid in that close ‘grouping’ is indeed the smaller Csize *Lycaon*. The next closest canids (again in order of Csize) are C. *simensis*, *C. latrans*, *C. dingo,* and *Cuon*. The only one that may be trending away from the regression line (though still inside the prediction intervals) is *Chrysocyon*, which is decidedly not a large-prey specialist.

Another result of the log CS vs log body mass regression on Fig. 3A is that all canids have relatively large crania for their size, as they always fall on top of the regression line. Could then the convergence with canids explain why the thylacine lies over the marsupial regression line (Suppl. Fig. 2)?

This may have been a misunderstanding of the legend, but canids (orange circles) do not have relatively larger crania than our metatherians (blue diamonds) in this figure.

L127-128: Either “the thylacines’ cranial size puts them” or “the thylacine’s cranial size puts it”.

You are right! I had to look this up 😊 thank you!

L132-138: As stated above, this is one of the clearest examples of pushing the extinct predator group narrative to the detriment of alternative hypotheses. Compounding on that, the authors list no references supporting the thylacine’s hypercarnivory, with the listed references (26.27) corresponding instead to Chrysocyon’s diet. Thus, this paragraph needs to be rewritten. Either further evidence of the thylacine’s diet which negates the intake of fruits needs to be referenced, or the authors should acknowledge that an alternative hypothesis to their results would be that the thylacine was a predator of small-sized vertebrates which potentially complemented its diet with fruits.

As outlined above, we made the mistake of not outlining the thylacines’ hypercarnivorous dentition early enough and this is now fixed in the introduction. It also reminded us to be clearer about the fundamental differences in dentition between thyacines and canids – thylacine dentition is highly reduced in its occlusal complexity (more so than any other dasyuromorphian, including the hypercarnivorous spotted-tail quoll), buccolingually compressed, the anterior molars are strongly reduced, and the lower carnassial is lacking any grinding platform (it has a trenchant heel).

Chrysocyon actually has a dental occlusal complexity higher than that of many canids.

e.g., see character matrix in
Prevosti, F. J. Phylogeny of the large extinct South American canids (Mammalia, Carnivora, Canidae) using a "total evidence" approach. Cladistics 26, 456-481 (2010). https://doi.org:10.1111/j.1096-0031.2009.00298.x

L150-152: If I understand it correctly, the argument here is that the geometric mean, as a proxy of cranial size, increases with condyle width, as a proxy for body mass, with a higher exponent in the thylacine. If that is the case, I would strongly recommend the authors to invert the axes of Fig. 4A. As the visual interpretation of the current panel suggests a lower exponent for the thylacine, which I assume the RMA slope actually is if the geometric mean was used for the x values. While RMA regression incorporates residual error for both variables, it is still best practice to match the regression formula with the desired interpretation of the result. In this case, for the higher scaling of cranial size to body mass, condyle width should be used as independent variable (x).

Thank you so much for pointing this out, this is an excellent suggestion and has been implemented.

L153: I don't think saying that this regression mirrors the interspecific differences in cranial centroid size, as they're comparing rates of change to average values

We are not sure we follow – the intercepts of the regression show that there are differences in cranial centroid size, and the slopes show that these differences become even greater across the whole sample of thylacines. This means that the average values presented in the interspecific comparisons reflect a fundamental scaling difference that makes bigger animal cranial disproportionately large, as outlined in this sentence.

, but I do think these results could serve as a hypothesis of why the thylacine have relatively large heads for their size: they increase in size relative to body mass faster than in canids.

We believe that this is exactly what we suggested ("the cranial size of thylacine individuals increases disproportionately with the body mass proxy of occipital condyle width").

This leaves the question however, on whether that is a marsupial trend related to their altricial development.

This is a good point which we initially did not plan to expand on, but the queries from R1 regarding the distinction between developmental and functional processes gave us another reason to add this point to the manuscript. We have added a perspective on this in the P. 16, middle paragraph and are really pleased that this concept ended up in the manuscript!

L160: I would suggest removing "related" from this sentence to avoid the potential misinterpretation that the thylacine was a canid (despite how clearly the contrary is otherwise stated in the manuscript).

Thank you, that was definitely not the appropriate word is now changed.

L163-165: This is another example of the authors pushing their narrative over alternative explanations. For once, the process is not as "extreme" as the authors claim, as for some of the studied variables, while different from larger canids, the thylacine's regression lines seem to extend trends observed in the smaller species. For instance, their scaling of both palate length and width at the canines is not significantly different than any of the studied foxes or jackals.

Thus, another interpretation of the same results could be that, by having crania larger than similar-sized canids, they are able to maintain proportions similar to those of smaller canids, all small-prey specialists. This would support previous studies suggesting that the thylacine was a small-prey specialist (Attard et al. 2011; Rovinsky et al. 2021) probably using pounce-pursuit strategies (Jones & Stoddart 1998), so I am unsure on why this isn't included here.

We believe that we had made exactly this point by writing (here with fixed wording) :" Thus, if the size of prey taken was similar to ~~that of the thylacines' smaller relatives~~ that of the small canids with similar shapes to the thylacine (i.e., <<10 kg on average), facial gracility would allow for the optimisation of jaw-closing speed and mechanical efficiency while still retaining an appropriate bite force from the absolute increase in muscle from simply having an overall larger head".

This is another sentence where we believe it is clear that we did not suggest that the thylacine was a piscivore.

Another topic is that, while the thylacine's gracilization has been shown to be different than that of some of the studied species, differences were also found within that pool of other species, yet this is disregarded. All this, coupled with the proliferation of studies on many-to-one relationships between bone shape and its function, makes the argument presented here a bit thin.

This comment probably goes back to our overall intention of focussing on the Thylacine. It is true that there is a many-to-one relationship between bone shape and a general function (e.g. many ways of being a small-prey specialist), but this manuscript is about the thylacine and the fact that its particular combination of traits give its cranium a unique function, despite showing a patchwork of similarities across the comparative sample.

L173-184: This whole paragraph provides further support to the thylacine being a small canid equivalent, but yet the authors dance around that statement to maintain the extinct predator group narrative.

This paragraph is why we were surprised that the impression arose that we were arguing for the thylacine to have a different diet to canids. Dietarily, we never thought of suggesting that it preyed on other prey than small canids. The point of the manuscript, and all the different comparisons, was to show that the thylacine's probably small-prey specialisation came with a completely different set of functional traits.

L188: As noted above (L340 & Figure 5), please provide a better visual aid for the reader to understand this "terminal rosette".

Done, thank you – the new figure looks great and makes the point much better!

L190-192: While this might be true for crocodylians, the thylacine's rosette doesn't seem developed enough to grant this hammer-like properties. Maybe a better figure would help this argument, but as it is, I don't see enough support for this. Figure 5 illustrates a relative pinching at the canines, but that doesn't provide any insight on the mass distribution of the snout. A potential solution to this would be to calculate the voxel proportions of the rosette relative to the rest of the snout in the cranial meshes, but this might be a very rough approximation if the meshes were acquired using a surface scanner instead of CT.

We have taken out the "hammer" analogy because it was not necessary, but maintain that a widened snout tip will increase the force of impact by simple lever mechanics. This is because the tip of the snout, if moved relative to the occipital condyle, is the fastest-accelerating part of the skull just because it is furthest away from the centre of rotation. Thus, even if the tip is not unusually heavy, if it is wider it will automatically have relatively more impact compared to a tip that is not widened.

L194-195: Relatively tall compared to what? Can this be observed in the shape changes along PC1 or PC2? Based on Fig. 1A, the thylacine's snout doesn't look taller than the wolf's.

These were brilliant comments, thank you! The high-snoutedness of the Thylacine and *Andrewsarchus* is not part of its differentiation from other species on PC1 in Fig. 1a or B, because traits that are unique to individual (or a small subset of) species tend to not show up in the first few PCs. This is because PCs display spectra of patterns that relate to the main variation variation across all species, and thus neglect unique cases. What we should have done earlier, and have done now, is add another set of distance measurements that also quantify the relative height of the rostrum. This shows that the thylacine (and Andrewsarchus) are also much higher-snouted relative to the narrow post-canine region than any other species.

L199-203: Would this make C. simensis also part of this potentially extinct predator group?

We are unsure how to interpret this question but it probably comes down to the misunderstanding discussed above that a species with *one* trait that resembles the thylacine needs to be discussed in terms of how the thylacine might be like it. Functionally, this is not a good approach to take. However, we also re-worded this paragraph because *C. simensis* clearly does not show the same high-snoutedness that the thylacine displays, giving its snout a very different biomechanical strain resistance profile.

L211-219: This paragraph is the final leap on the extinct predatory group narrative: What if the small, fast-moving prey mentioned earlier weren't small mammals but fish? Then the thylacine might be a piscivorous predator because it has a "terminal rosette" like all these other clades.

I don't think that this manuscript provides enough evidence to support this claim.

This paragraph does emphatically not say what R3 thought it says! We also cannot find a way that our paragraph could be misinterpreted since 1) we noted that the piscivorous vertebrates "…are clearly not ecologically analogous to the thylacine…" and 2) we emphasized the existence of other extinct terrestrial mammmals with a similar morphology.

Note also that we later refer to the thylacine as "…the last large marsupial mesopredator from the ecosystems of lutruwita (Tasmania)." It hopefully emphasizes our total lack of intention to frame this animal as piscivorous.

As outline above, this misunderstanding probably arose because we took it for granted that the thylacine hunted small, terrestrial vertebrate prey. We have corrected this now.

L220-234: While interesting, this whole section on IOF doesn't feel connected to the rest of the manuscript, particularly when the lack of developed whiskers in the thylacine would be an argument against their piscivorous diet narrative. Why delve into it here then? I believe this might be an interesting short study on its own. Maybe add some other dasyuromorphs and map

IOF surface during their evolution? Couple it with evolutionary modelling of dental over-eruption to test their hypothesis on soft tissue maintenance?

This is a good point and we had been thinking about this as a potential separate short study. However, part of the problem with interpreting thylacine function is that different lines of evidence are often not sufficiently combined because they are difficult to fit into a narrative. In this case, the IOF does produce an important view on another unique thylacine trait that is not picked up by shape analysis.

In addition, we find it relevant that the genetic parallels between wolf and thylacine development, outlined above, affect several genes that relate to the trigeminal nerve which presumably went through this enlarged IOF.

L237-243: See most of my comments above, but this needs to shift from present tense to a hypothetical conditional tense.

This section (starting with "Our study has revealed multiple lines of evidence that the thylacine's striking cranial convergence with the wolf is an emergent property of a trait combination with no living functional equivalent") summarises what we believe is an accurate representation of our finds. We do not believe that, after our analyses, there can be any doubt about it. Nor do we believe that there is reason to not articulate how much worse this makes the loss of the thylacine in the scheme of mammalian biodiversity loss. We prefer to keep it as it is.

Reviewer #3 (Remarks on code availability):

I did not review the code because I was unable to access it, as it is locked to gitfront users.

We have now included a zip file of the code with our submission; hopefully this helps.

Summary

We thank the reviewers for a rigorous and constructive review process, which has made a tremendous difference to the manuscript. The response document is now very long, so we here summarise the main changes.

1) **Introduction and discussion improvement:** we have further reduced our emphasis on just the wolf-thylacine comparison and are now clearer and more explicit about our aim to articulate how thylacines are unlike the whole sample of mammalian carnivores by re-writing the introduction. This also included a re-working of the discussion to provide more clarity as requested by R3 and R5.
2) **Improved comparative context:** We have re-written and extended the results/discussion to include more information on individual other species. Even though R3 was happy with our existing re-write, their comments still showed concern about our lack of broader discussion. We thus added more extensive, maximally concise comparative discussion while remaining within the word count and narrative flow.
3) **Substantial additional analysis**: We added several additional analyses and presentations as requested by the reviewers. This included a clade-based allometric analysis (presented in a much-extended supplementary results section and additional coding files), phylogeny-based presentation of Procrustes distances in Supp. Figures 3 and 4 to support Fig. 2, and several figure improvements of colour scheme and labelling. We also engaged in an extensive assessment of the Wheatsheaf index proposed by R5, which we ultimately did not deem suitable for our data (see end of manuscript for detailed explanation).

Reviewer #1 (Remarks to the Author):

This is a fully rewritten version of the manuscript I previously revised. I want to make clear that I sincerely appreciate the efforts taken by the authors to rework and make clearer their ideas and thoughts. Before moving on, I would like to make some clarification about the answers provided by the authors.

First;

ok, this is not a paper on convergence and this a shame. Possibly I was expecting/hoping to read a paper on convergence, but this was not the case. However, I still recommend to replace the word "convergence" with "similarities" throughout the manuscript. However, I deeply appreciated the effort taken by the authors to rework the manuscript. As a side note, the metrics adopted in Rovinsky et al. (2021) are outdated, so, just for fun, I would re-run some of those analyses, take also a look at Grunstra et al. (2024) have a better understanding of what is possible to do with Procrustes distances.

Thank you for this kind appraisal. We are glad he re-write helped. We saw the Grunstra paper with interest and it is very relevant to many aspects of our work and pertinent to a number of comment responses about the techniques and sample sizes. As you will see, we tried to use the Wheatsheaf index implementation as per Grunstra (and also recommended by R5) but were found it inappropriate to apply for our particular case (see explanation and results presentation at the very end of this ms). In addition, we prefer to retain the nature of this paper as not being about convergence per se, noting that this has been formally done twice just within the last decade (Feigin et al., 2018; Rovinsky et al., 2021). Additionally, while we do note that issues regarding the very commonly-used *c*-measure have been raised, we do not expect that the multiple methods used in Rovinsky et al. (2021) - not just *c*-measures - are so outdated as to produce substantially misleading results.

Second;

"With ~160 million years evolutionary divergence and distinct differences between placentals and metatherians, of which the thylacine is the only exception in aspects of its shape, there is no need for testing if these differences arose independently! In addition, this has been done before e.g. in Rovinsky et al. 2021. We are now clearer that this is a reasonable assumption in the introduction (2nd paragraph)."

I probably expressed myself badly here, sorry. However, I was meaning that in dealing with convergence you test whether two species significantly depart from their ancestors' shape toward a common area of the morphospace. That is, without a phylogenetic background it is impossible to trace such ancestor-descendant pathway, independently of how much evolutionary time separates the two focal taxa. This is far from a trivial as more often than not, putatively convergent taxa fail such test. Hope this is clearer.

Again, this is not a study on convergence per se, and convergence between the thylacine and canids in general has been well-noted (e.g., Keast, 1982; Moeller, 1968; Werdelin, 1986; Wroe et al., 2007 for a very abbreviated selection) and empirically tested - including analyses incorporating their respective phylogenetic backgrounds (e.g., Feigin et al. 2018; Rovinsky et al., 2021).

"We did not, as R1 suggests, use Procrustes distance comparisons as a naïve and phylogenetically uninformed way of testing convergence. And, as outlined above, there is no relevance to include ancestor-descendant relationships because it is clear that any similarities between the thylacine and canids are not based on a common ancestral condition. Instead, our study goes back to the very basic but also deeply fundamental question of asking which animal the thylacine resembles most in different sections of its cranium, based on previous results (Rovinsky et al. 2021) that similarities between canids and thylacines are not consistent with any carnivoran ecomorph."

Sorry, but this is exactly what I said in my comment. You simply measured the distances between some species and the thylacine (here taken as reference), without accounting for phylogeny. There's nothing bad about it, but distances are silent other aspects, namely phylogenetic history. Furthermore, in my comment I haven't by any means claimed that PCA is better than using Procrustes distances, as it really depends on what your scientific design is about.

This comment clarified things a lot, thank you! It appears that we are in agreement – our question was explicitly not with view to ancestor-descendant relationships, and solely focused on similarities. For our question on similarity in shapes and the functional implications of these similarities, how they arose is not relevant. The last paragraph of our introduction hopefully makes this even clearer as well.

In this picture (see the attached file) is shown the full Procrustes distance (rho) and the partial Procrustes distances (Drho) between a reference (the red point) and an hypothetical configuration A. Now, what is generally measured, as you did, is the distance Drho. Now, you can imagine another configuration B exactly at the same Drho, but on the -1 side. That is, two configurations can have the same partial Procrustes distance from the reference, despite having different shapes, that's why we need to visually explore the shape changes. If the authors feel I am not adequate or competent in GMM, please have a read at the PalaeoMath Series from Norman MacLeod (https://www.palass.org/publications/newsletter/palaeomath-101) where these topics are treated in a much more mannered way.

Again, my comment was intended to make the author fully aware of the limits in using such distances.

This fortunately means that again, we are 100% in agreement and it looks like the concept of "signed" differences (which are not easily applicable to multidimensional variation) caused some misunderstanding.

The image provided shows exactly our understanding of Procrustes distances (or ordinated summaries thereof), and the effect in the illustration is the reason why we employed them. We extend R1s' illustration with another species (blue) and some text to break this down a little further for our particular case, as it applies to previously suggested similarities between wolves and thylacines. Separated into their individual partitions, we showed that this similarity arises from a combination of two patterns that provide the same "sum result" (red dot) but from divergent combinations of sub-patterns (blue/green dots; where blue could be wolves and their relatives, and green could be foxes and their relatives). This is just as R1 explained, but perhaps the additional illustration will settle the issue entirely.

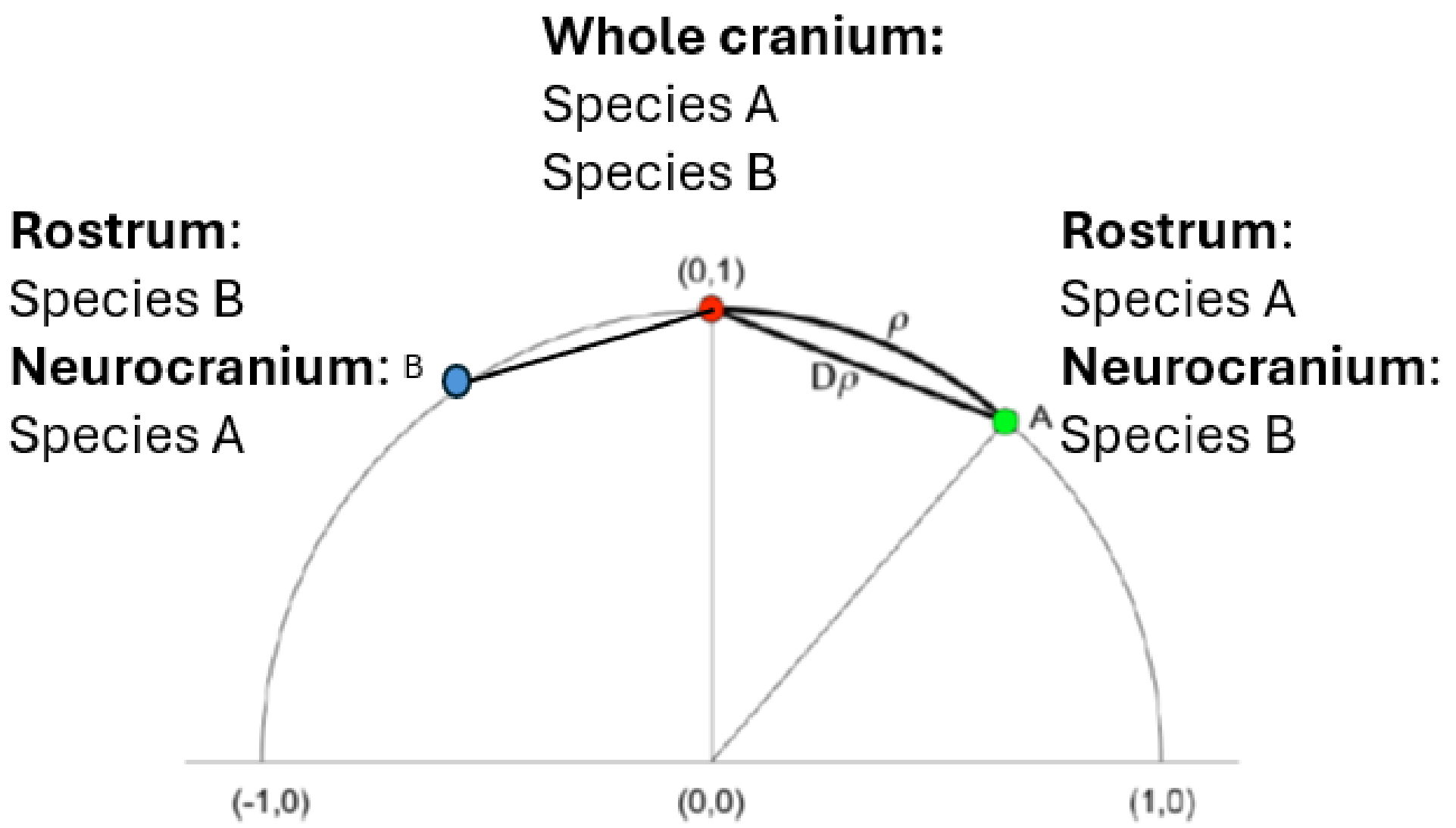

Now, I hope everything is clearer and I can move on reviewing this new version, which is not about convergence. However, this version reads much better than the previous one and is way clearer about the study's aims.

However, I still have some concerns the main one related to the diminutive sample size available. I do not think that 59 species are capable of capturing the variability of placentals carnivores (280 species) and/or marsupials. As a matter of fact, you lack ursids, many felids, many viverrids and mustelids are very poorly represented in the sample. Nonetheless, you lack almost all of the largest carnivores (lions, tigers, brown and white bears), which could have added a lot more considering that an allometric analysis has been performed. Also, it would have been a good idea to add some more fossils, in particular sparassodonts (when available of course).

This is a misunderstanding; we did not set out to explore the variability of placental carnivorans, nor would it be methodologically sound to include them here. The sample was intentionally chosen to address ecological form-function hypotheses of the thylacine, and as such, it focused on terrestrial mid-sized carnivorous species (those between 5-50 kg with diets consisting primarily of vertebrate flesh). Adding species with sizes or diets/ecologies greatly outside of this would be incongruous with regards to the study at best, harmful at worst (e.g., in respect to the variation reflected in Principal Component Analysis and resultant downstream analyses). The main exceptions to this focus are:
A. the number of smaller-bodied felids, viverrids, and musteloids which are included - mainly to balance and contextualise the marsupial dasyurids and didelphids, nearly all of which are <5 kg with few highly carnivorous forms.

B. *Andrewsarchus mongoliensis*, which was primarily added due to the noted superficial cranial similarity to the thylacine (Scanlon, 2009).

We have added a clarification at the beginning of the methods (P. 19). We also contest the suggestion that sample sizes were insufficient (the Grunstra paper R1 cites above has an even smaller sample of 48 species, and clearly has dependable results; see also R5 comments).

In addition, allometric patterns were not initially a focus (they were added to the analyses based on reviewer feedback to demonstrate that allometry does not play a role in explaining the thylacine's place in morphospace). But also, allometry is not a process in itself and rather the result of diverse and oftentimes independent effects. Across our large diversity of species, it is more likely to be an emergent property of processes such as masticatory adaptation and relative decreases in brain size (for a review, see Mitchell et al. 2024 as cited in the manuscript). Adding species that are clearly not dietarily comparable with the thylacine, such as the including the well-known allometry of large-prey specialists like bears and large cats, will thus not serve to clarify any point.

We also wholeheartedly agree that adding more fossils would be optimal (it is for nearly all analyses of our kind). However, as R1 suspects, while there are some sparassodonts with reasonably complete cranial material known (e.g., *Callistoe vincei, Lycopsis longirostris, Acyon myctoderos, Cladosictis patagonica, Sipalocyon gracilis*) we were unable to reasonably source any sparassodont aside from *Borhyaena tuberata*. As far as we are aware, of the reasonably complete crania known, only *Borhyaena*, *Sipalocyon*, and *Thylacosmilus* have been scanned. At the time of writing, the *Sipalocyon* scan was not available, and *Thylacosmilus* is so radically apomorphic that it would add only spectacle to the study. Unfortunately, no one involved in this study was able to travel to the various South American countries or liaise/collaborate with researchers therein in order to collect new scans.

Another issue is represented by the allometric analysis. Below, I provided more details, but in summary running regression PC by PC is a very discouraged practice in GMM (please see any contribution from David Polly, Dean Adams or Philip Mitteroecker)

We have now addressed this issue through substantial additional analysis, and refer to our response below under "minor points". However, we are not convinced that these additional results are helpful because any analysis really does depend on the question asked. The Polly/Adams/Mitterroecker papers all correctly criticise the use of Principal Components as representatives of biological variation. This is because existing patterns can be "smeared" across multiple PCs if they do not relate to the main variation (e.g. it is not appropriate to ask about the contribution of allometry, sex, or precipitation to the overall variation in a sample by regressing these variables against individual PCs in a sample). We agree with this point (Weisbecker et al. 2019 also makes it).

However, in our case, we first appropriately assessed for overall allometry using PGLS (and now also added clade-by-clade analyses). We then added the axis-by-axis analysis explicitly to test if 1) allometric patterns dominates the variation in the sample (in which case, PC1 should be the only allometric PC) or 2) if several independent allometric patterns exist in the sample (in which case, multiple PCs – each calculated to be maximally independent of each other – should correlated with size). The latter result turned out to be correct, answering in the negative the previous review's query about whether the thylacine's cranial shape might be due to a single, strong, overarching allometric effect on the whole sample.

Another problem is the use of size as a proxy of bite force. This came out without any testing, and I am not very happy with such a choice. Cranial size is not the only determinant of bite force as muscle orientation and distance (to make an example) make a lot of difference. So please remove it or analyse it differently, like taking bite force data from other published venues where a proper biomechanical analysis was performed.

This is a new query on original text for this review round. We feel that our comments regarding cranial size and bite force are not only justified in the literature we cited originally, but also laws of physics. As we had extensively discussed and referenced in the manuscript, bite force allometry is a well-understood and widely observed phenomenon where larger skulls, all things being equal, can resist harder bites. This means that a larger-skulled species can have a biomechanically less efficient skull if it bites into the same foods as a smaller species. We propose that this effect explains the combination of (weakening) gracility and (reinforcing) large size of thylacine crania. We also now cite a study one of the us just published, with biomechanical analyses that exactly correspond to the scenario we propose for the thylacine - the small but very robust-skulled desert rat-kangaroo can only resist bites about as hard as the much larger, very gracile skull of the potoroo (Mitchell et al. 2025).

Moreover, while having 'real' bite force data would be wonderful, analyses of bite force are very rare and not available for the vast majority of the included species. Furthermore, as there are multiple different methods of acquiring bite force data (e.g., force plate transducer, multi-body dynamics, finite element analysis, physiological cross-sectional area, etc.) the resultant data are not directly comparable between studies. Lastly, we are not analysing bite force per se, only commenting that, all things being equal, a larger head will tend to produce absolutely greater force than a smaller one, even if relative force production is equal or lesser.

Minor points

Abstract

I recommend to minimise the use of the word "convergence" here and throughout the manuscript, as you did not measured it. Replace with "similarities" or something like that.

We have gone through the manuscript and changed the term where possible and appropriate. In the abstract, the word is part of a sentence referring to previous work about thylacine convergence, so we are leaving the term in.

Introduction

Lines 71-73: This would have been interesting to test.

We agree that similarities of thylacines with sparassodonts have been under-explored since the tests in the 1980s cladistics publications. However, we only had one sparassodont that was obviously dissimilar to the thylacine, and it was not in the scope of our manuscript to explore this further.

Lines 81-82: I have some issue with using size as a proxy of bite force.

See comments above: Physics dictate that a larger head will have a stronger bite than a smaller head unless the smaller head is substantially sturdier or otherwise adapted to higher bite forces. This allows gracility in larger heads of animals that eat the same diet as the animals with smaller heads (see MItchell et al. 2024 for the physics and Mitchell et al. 2025 for the experimental corroboration).

Results and Discussion

Line 93. I have issues with such a diminutive sample size. Carnivora include around 280 living species and many more extinct (see Faurby et al. 2024). I do not think that this sample can be considered representative of the carnivoran variability. Also, as mentioned above, it would have been good to include some more sparassodonts as this could have shifted our view of thylacines to be more similar to canids than to other taxa.

See comment above. We did not set out to catalogue carnivoran variability and we added as many relevant fossils as we could.

Line 134: How can you say that dingoes and wolves are unique among canids?

We have completely re-written the paragraph to place the emphasis even further away from the wolf-dingo comparison, as suggested by R4. The whole text is now different and discusses the canini (including dingo and wolves) more broadly (p. 8).

Line 136-138: This is a fundamental problem with this manuscript and what I tried to make evident in my previous review. Without any proper phylogenetic comparative analysis, you can't say anything about any putative departure of any species from their ancestors. This looks very speculative to me.

This sentence is now gone as part of the re-write of this section, but its content ("As we can assume that canids and thylacines do not share an ancestral cranial shape due to the thylacine's unique shape among marsupials…") is not contentious or speculative. As noted above, and as had already been noted in the introduction, this conclusion has been made several times before and there is no reason to test it again).

Lines 157-161: I got what you mean, but please rewrite it. Without having a look at the supp it's hard to get your message. Anyway, it is highly discouraged to analyse single PCs separately, as you arbitrarily selected 10 PCs and performed allometric analyses PC by PC finding little evidence to say that allometry is meaningless in your data.

The correct way is to analyse the full set of Procrustes coordinates vs size (as you did at first). Here, since you get a significant result, it would have been nice to compare the allometric slopes of shape data. You could have run separate pooled regressions (e.g. separating Canidae, Felidae, etc.) and test whether these resulting slopes were convergent, divergent or parallel. Also, a deeper investigation of allometric slopes would have revealed whether the Thylacine can be considered "hypermorphic" compared to other marsupials.

In the end, please remove the PC by PC analysis as it go against any development in modern morphometrics and rephrase the paragraph.

We added the clade-by-clade allometric analysis as requested, and removed the PC1 and 2 analyses of the previous Table 1 (see above for details). We have kept the analyses of the first ten PCs in the extended results because these do answer an important specific question about whether or not allometry dominates the variation - this is different to the early-2000s fallacies of using PC1 to ask if (and how much) allometry exists in a dataset, which we agree should not be done (see above).

Figure 4. I guess you miss-labeled the axes in figure 4a.

This was done on purpose upon request by R4. We agree that this does a better job at highlighting that the thylacine cranium is consistently much larger than the postcranium (the occipital condyle width is a routinely used indicator of postcranial dimensions relative to cranial dimensions). We have now added an explanation to the caption.

Reviewer #2 (Remarks to the Author):

We believe that the authors did a great job either incorporating the modifications that we requested or addressed our concerns in their response. The only line edit we caught was Line 36: "observatione.g.,".

Done and thank you very much for this positive appraisal!

Other than that, we look forward to seeing this paper in print.

Reviewer #2 (Remarks on code availability):

NA

Reviewer #3 (Remarks to the Author):

Overall, this revised version of the manuscript has vastly improved upon the original, both in terms of structure and background information, and by addressing the comments of three different reviewers, so I wanted to congratulate the authors on that.

I particularly like the rewritten Introduction (but see comment for L292-298). Both the restructuring of its previous sections and the added bits of information (particularly regarding the thylacine's diet) help paint a clearer picture of what we currentlly know about the thylacine's ecology and relevant morphology and what this manuscript aims to address.

Regarding the changes to the Methods section, I am unable to discern whether they're easier to understand because of the rewrites, or because I already know what those analyses are from the previous versions. Hopefully other reviewers will add notes on that topic if they still think it's unclear. No notes from me though.

Thank you very much, we are glad that the re-writes helped.

As for Results & Discussion, my thoughts on the authors' justification for not describing other species and just focusing on the wolf-thylacine comparison can be found at the end of this document ("Keeping the dialogue open" section), but I wanted to note here why I still slightly disagree with the authors in that regard.

Specifically, there is a disagreement between the distance values plotted on Figure 2 and the distance values presented in Suppl. Table 1.

If the latter are correct, the pattern described for the grey wolf and dingo (closest to thylacine in posterior cranium but far away in rostral region) applies to all Canis species except C. simensis, and to Cuon and Lycaon as well, not just the grey wolf and dingo. This shifts the results away from the uniqueness of the wolf and dingo relative to other canids and makes it a trend within the Subtribe Canina (now Lupulella and C. simensis are the exceptions to cranial shape/development within Canina). With this in mind, the authors could expand their hypothesis that wolf/dingo and thylacine both diverged from their ancestors to evolve a similar cranial shape, to this divergence occurring early on in the evolution of Canina, right after the Canis/Cuon/Lycaon branch diverged from Lupulella. C. simensis might represent a shift back to the ancestral canid condition, an early stage of divergence towards a new set of cranial features (see comment on L256-263), or their Ethiopian wolf specimens might be incorrectly assigned to that species*. If the authors agree with this interpretation, the overall discussion of their results could be easily shifted from the wolf comparison to a wolf-like canid comparison (i.e. Subtribe Canina).

Great catch and many apologies! This mis-match was due to our change of landmarking protocol to include the zygomatic arch landmarks at R3's request. The values in Supp. Table 1 were correct but we mistakenly did not upload the updated figure (and obviously didn't catch that the figure didn't change). The discrepancy is seen in the mock-up below (orange squares to the left are the new values). This shows that adding the zygomatic landmarks moves all species closer to the thylacine by roughly similar amounts.

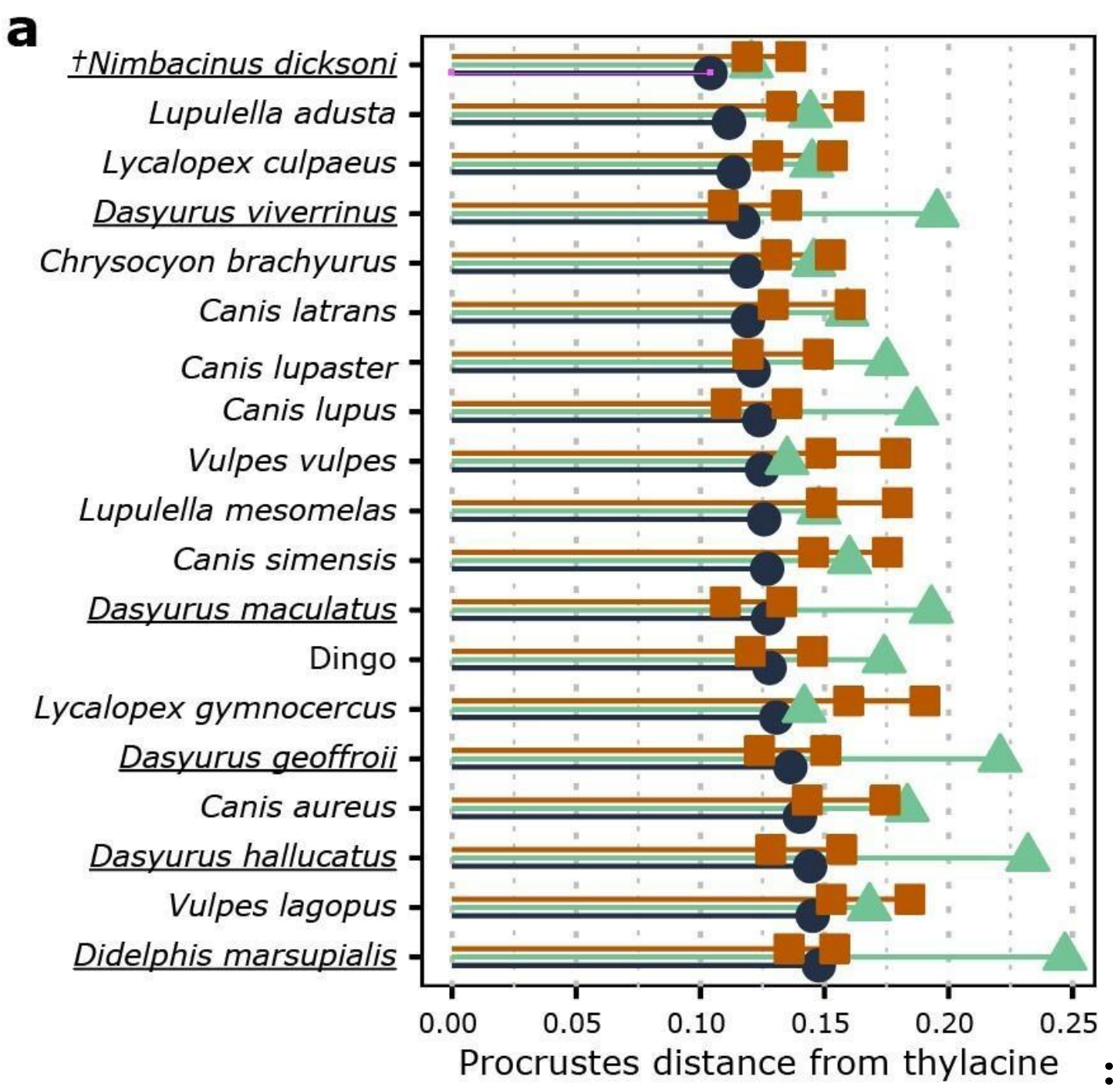

This does have the effect highlighting R3's point that dingoes and wolves follow the general trend where canines (minus most of the vulpins) are closer to the thylacine in the neurocranial set than the rostral set. The two exceptions to this are *Lycalopex gymnocercus* and *Lupulella mesomelas*. The latter has values only slightly different from each other (Procrustes distance Rostral:Neurocranial 0.1478:0.1489) that it would be better to say they are equidistant. The Pampas fox (*Ly. gymnocercus*) is interesting in the vulpin-like pattern, and it would be curious to see if the rest of cerdocyonina – which by-and-large are much more 'fox-like' than e.g., the culpeo, bush dog, or maned wolf – follow a similar pattern.

That said, we do note that we are comparing apples-to-oranges here, and in reality should only view these 'patterns' in the most general way possible. They are derived from separate generalised Procrustes analyses and as such should really only be interrogated between species (not between sets within each species).The between-set 'patterns' may reflect real biological trends, but cannot be held up as empirical realities as this cannot be tested. We are now making this clearer in the caption.

All aside, the comment R3 put forward helped us understand what kind of additional discussion R3 was after. We have accommodated this, hopefully within the space allowed in the manuscript, by adding a more informative list of Procrustes distances (now ordering each partition's distance values relative to the thylacine) (expanded Fig. 2) and producing phylogenies that support R3's suggestion for the shape variation within canids (new Supp. Figs. 3 &4). We are aware that R1 may take issue with the lack of testability of these hypotheses, but place them here as a speculative starting point for further research with new fossils required to establish the polarity of the evolutionary changes.

* In my experience, misclassified specimens are common in museum collections, particularly when they were collected in the early 1900s or when they belong to species in distant continents.

Beyond that, I wanted to congratulate the authors again on a fantastic job improving this section. It reads much better, all my doubts regarding a specific narrative being pushed against other potential explanations have been quenched with clarifications and new analyses and improved figures, and there is no room for misinterpreting the authors as stating that the thylacine was piscivorous (sorry about that btw!). Congratulations!

Thank you very much for this positive feedback and final improvement suggestion!

Eloy Gálvez-López (anonymity waived)

L31: Change "carnivorous" by "hypercarnivorous". I think this will solidify any potential doubts regarding thylacine diet.

Done

L36: Fix typo: "observatione.g."

L41: Either "similarities of the thylacine with canids", or "similarities between the thylacine and canids"

Done

L72: Fix typo: "sparassodontse.g."

L386: That comma feels weird. I would remove it, but this is just a suggestion, not a request.

Done

L397: You might want to rewrite this line as: " We log10 transformed centroid sizes and regressed them against log10-transformed species' average body masses [...]". I believe it's shorter and easier to read.

Done

L451-456: This paragraph no longer has a purpose, as "grey" has been added to all other relevant mentions of wolf (except L293), clarifying that the authors are referring to this species only. I would strongly recommend the authors to delete it.

That's a good suggestion. Done.

L456: If the authors don't remove those lines, the "prey30" typo needs to be fixed.

Done

L124-L141 & Figure 2 / Suppl. Table 1: See comment above. This paragraph should be rewritten to acknowledge the discrepancy (and potential new evolutionary hypothesis), and the figure modified to present the correct values.
See above and we have now modified the figure.

Extended Results: "Excluding A. mongoliensis from the analysis produced a nearly non-significant association ($R2 = 0.33$, $p= 0.000$) between cranial shape and log(cranial centroid size)". I am quite confused with this statement. Why is this nearly non-significant? If this is based on $p=0.0000$, which is common when outputs are limited to 4 decimal places and the value is lower than 0.00005, the results are actually the opposite (i.e., excluding Andrewsarchus increased the significance of the allometric relationship). With significance set at $p=0.05$, a nearly non-significant result would be $p=0.0499$ or higher.

L159-160: This no longer seems to be true, based on my earlier comment regarding the disagreement between distance values in Figure 2 and Suppl. Table 1. I would recommend extending this argument to Canina instead.

L172: I don't understand what you mean here. It reads as if something is missing after "absolute".

This was indeed a mangled sentence, and has been restructured.

L219: I assume defining small prey as < 10 kg is a ballpark threshold, as there's no associated reference. If that's the case, I would recommend changing this to < 7.5 kg, representing 45% of the thylacine's latest

body weight estimations (ca.16.7 kg). Small and large prey are usually defined relative to predator size and Carbone et al. (1999) proposed that 45% threshold based on the energetics of hunting and prey processing.

Carbone C, Mace GM, Roberts SC, Macdonald DW (1999) Energetic constraints on the diet of terrestrial carnivores. Nature 402, 286–288.

No problem, we have added this and agree that it is a better reference.

Figure 5: I just wanted to state how gorgeous this figure is now. Zero doubts about what the terminal rosette is. Fantastic job!

Thank you, that is great to hear!

L247: Fix “see aso” typo.

Done

L256-263: If the authors adopt the wolf-like canid comparison focusing on Canina instead of just grey wolfs and dingoes, this could be a great opportunity to explore why C. simensis does not follow the trends observed in other Canina. Could the Ethiopian wolf be an early state of divergence towards acquiring a morphology similar to the thylacine/Andrewsarchus? They seem to be developing a terminal rosette, could the development of a taller snout and relatively large head follow?

This is an interesting thought and worthy of further study/incorporation into a larger dataset. We suspect that a larger head/body would not necessarily follow, as that seems to potentially be (slightly?) decoupled from the prey acquisition morphology. I.e., if the Ethiopian wolf’s prey is minute and there is no non-predatory reason to attain larger size/larger head (e.g., sexual selection/combat), then we would not expect to see it.

L274-287: I still don’t think the IOF analysis and discussion connects with the rest of the study, but the authors make compelling arguments for keeping it in their rebuttal.

Thank you for your patience with this. We totally understand that it takes a bit of a re-orientation to move to the IOF part, and have tried to improve on the segue. We are excited to be able to present evidence from diverse sources including the IOF.

L292-298: This paragraph is the perfect example of how vastly the authors have improved the manuscript in this revision. The paragraph itself has barely changed but, while in the previous version I thought it exemplified all my issues with the manuscript and I requested that it be changed to a conditional tense, now I believe is the perfect summary for the preceding section. Again, great job.

Thank you so much, really appreciated and glad we could make a meaningful change.

L326: “thylacine-canid comparison” instead of “thylacine canid-comparison”

Done

L335-336: Delete spaces before reference numbers

Done

--- Keeping the dialogue open ---

Besides my comments on the manuscript proper, I wanted to address some of the topics discussed by the authors in their rebuttal.

Why we do not discuss other species more?

While I agree with the authors in that, when space is limited, not all topics can be discussed, I still think it would have benefitted the manuscript to spend some time addressing the species closest to the thylacine in the morphospace. In fact, I think it would have add support to the uniqueness of the thylacine phenotype as it shows how many different ecologies cluster in that region of the morphospace (this could even be a prime example of one-to-many morphofunctional relationship).

Agreed – and worth delving into much more so than this relatively short manuscript focused on the thylacine can do. A one-to-many morphofunctional relationship seems to make a lot of sense, too. It is also an issue for such deeper interpretations that the Principal Components Analysis approach is not ideal to truly show morphofunctional relationships because we do not know what kind of shape is summarised along these axes. It would be worth a separate study to summarise shapes that are similar (in the sense of agreeing across different partitions of the skull) and assess how many different shape types all do the same thing. We did not have space for these considerations, but the dataset is freely available online and we hope it will be useful for such additional work.

As for most of those species already having been discussed in Rovinsky et al (2021), maybe a more direct sign-posting should be included (e.g. "see Rovinsky et al (2021) for an in-depth discussion of X and Y species' morphology compared with the thylacine's"). While I praise the authors for the broad-picture scope of this study, combining findings from different disciplines, I feel that the fact that this manuscript and Rovinsky et al (2021) are companion pieces and should be read together, is not stressed enough. I read Rovinsky et al (2021) again right before tackling this review and it added great context to this revised manuscript.

That is a really valuable suggestion and we have gone through and made sure this is highlighted appropriately.

Why is our emphasis on the wolf-thylacine comparison?

The authors claim that they keep the focus on the wolf because of (1) high public profile (do they mean popularity?) and (2) existing literature plus popular science. I read the rebuttal before the manuscript and my first thought was that this read like a cop-out at best, or an attempt to be "media-worthy" at worst. Given my previous concerns regarding a certain narrative being pushed, it's fair to say that I started this review with quite an antagonistic mindset. Luckily, although the authors brush over the changes made to the manuscript to reduce the focus on the wolf in their rebuttal, those are significant and have truly changed my opinion on the manuscript. However, at a time when scientific research is often misconstrued by the public, lack of scientific rigor is often used as an excuse to cut funding (e.g. Argentinian government on CONICET), and the press is only interested in fluff science with clickbait-y results, I believe that we should refrain from letting high public profiles and popular science interfere with how we approach research. Please note that this is not a criticism to the authors, but a reflection on how scientific publications seem to be changing agendas from sound research to popular appeal and this is starting to seep into how we design or justify our research.

We are glad we could convince R3 but we also absolutely agree that rigour can easily fall prey to public perception. The problem we face is that consensus on what is "interesting" scientifically and publicly is hard to evade and also, crucially, hard to shake. As hopefully helpful background, VW conceived of this study as a way of breaking the lack of scientific rigour surrounding perceptions of Australian marsupials, who have a massive PR problem as second-rate mammals (journal rejections because of supposedly "local interest" are really common for Australian mammal papers). VW made a commentary on this on a YouTube-recorded talk at 32:30 minutes. Focussing a publication on a clearly mis-estimated species that is also of high profile seems like an efficient way of raising the profile of this scientifically relevant question while also highlighting a truly exciting evolutionary phenomenon in itself.

Back to the original question though, why keep the focus on the wolf, please refer to my comments on the discrepancy between Figure 2 and Suppl. Table 1. I believe in most cases the manuscript should shift to a comparison with wolf-like canids (i.e., the Subtribe Canina) instead of hammering on the uniqueness of grey wolfs and dingoes. In my opinion, this would increase the relevance of the findings presented here, and inspire new research avenues (for instance, looking at the cranial development of other Canina).

We had initially focused on the wolf-thylacine dichotomy because it is historically very common and still widely discussed, but we understand that this perspective should perhaps not get more air time than it deserves (not much!). We have therefore taken the focus off this comparison even further, particularly in the Procrustes part of the manuscript, and now discuss this part with respect to patterns within Vulpini/Canini and only passing mention of grey wolves.

Reviewer #3 (Remarks on code availability):

I have only reviewed bits of the code, particularly regarding some of the functions used. However, this quick glance suggested that the code as presented here will be a very helpful resource for the community.

This is great feedback, thank you. We are proud of publishing our code in working order ·.

Reviewer #5 (Remarks to the Author):

Overall Impressions

This study is a relatively standard, comparative, shape analysis of mammal crania, with the aim of interrogating the nature and degree of similarity in shape between the extinct thylacine and members of the Canidae clade of placental mammals. Other carnivoran mammals, including both terrestrial and aquatic taxa, along with additional metatherian taxa were also parts of the sample. I am in favor of the manuscript being published, but only if the authors (1) conduct a new statistical analysis that other reviewers have also suggested, (2) declare a competing interest on behalf of author AJP, and (3) generally clarify their grammar and caveat their narrative with alternate hypotheses. Because these modifications are non-trivial, I would classify them as major revisions, but they may be relatively straightforward given that the authors have all the data necessary to conduct the new statistical analysis.

Thank you very much for your balanced appraisal. I believe that we have been able to address the concerns raised, as per the below responses.

The study breaks down shape roughly into rostral and caudal “modules” (colloquially speaking), though the authors compare two schemes of such modules, one based on cranial functon(rostrum, braincase) and one based on developmental origin (i.e., tissue type; 2 neural-crest modules and 1 mesoderm module). The results show that the thylacine most resembles large-headed canids like the gray wolf or dingo in terms of the braincase module, whereas it most resembles smaller-headed canids like certain foxes in terms of the rostrum module.

Generally, the methods are appropriate to address the central question: how does the shape of the thylacine cranium compare with other carnivorous mammals? However, I agree with another reviewer that the methods are not adequate to determine whether the sampled metatherians and placentals have different allometric trends in cranial form. This question is one that authors start to address using linear morphometrics of size (visualized in Figure 4), but they have not yet conducted relatively standard analyses that would explicitly assess the presence and nature of allometric differences between lineages. I cite a couple of studies that used such analyses in my line-by-line comments. Authors should employ these methods for this purpose, as differing allometric trajectories between marsupials and placentals could impact the interpretation of their other results.

We have implemented these straightforward analyses, as per below – but please see our comments to R1 and also below as to why we are still unsure that these analyses are particularly helpful. They do not add much information to our question of whether thylacine crania are canid-like.

I also share a reviewer's concern about an overemphasized narrative of an "extinct predatory behavior". Authors claim that the thylacine was both exceptionally "unique" and yet also part of a grouping of extinct carnivorous mammals that all share some cranial traits, ergo they must share predatory behavior.

Neither of these narratives are well supported by the results. There are several canids that resemble the thylacine in both rostrum and braincase (across species within module); among these species are the maned wolf, culpeo, and coyote. It is apparent from the results that there are allometric differences in certain linear dimensions (see Fig. 4) among lineages, but these differences could be put in a different light pending an explicit analysis of shape allometry by lineage. The PC-by-PC analysis is not sufficient.

There is a misunderstanding here, probably related to how we have used the word "unique" which we have not replaced with more informative phrasing throughout. We note that the thylacine is shows a trait combination that does not exist among extant marsupials and carnivorans (in this sense, it is unique). We also did not mean to convey that it "groups" with extinct carnivorous mammals, only that it shares several specific, functionally clearly relevant traits with them. In other words, as R5 points out, thylacines share individual traits with a large number of disparate species in our datasets, but the combination of these traits is unique among living carnivorous mammals and includes traits known from a number of living and extinct carnivorous species, including mammals. We have tried to make this clearer by re-writing parts of the discussion.

This narrative with respect to the "terminal rosette" is also not well supported. Authors point out that the thylacine has this rosette at the rostral tip of the rostrum, resembling skull morphology of some reptilian semiaquatic predators as well as some extinct terrestrial mammals. Authors suggest that the thylacine's narrow snout coupled with the rosette means that it likely used a fast, snapping bite to acquire small prey items, and they also suggest that extinct mammalian carnivores with similar morphology shared this behavior. However, authors write as though this behavior, a "fast-snapping" bite, is extinct and has been overlooked because the last paragon of it, the thylacine, has no living analogs.

Apologies if this was the impression, this is not what we intended to say. We meant to convey that the way the thylacine's cranium shows adaptation consistent with fast-snapping, high-impact bites (mediated by a combination of large skull, long jaw, and terminal rosette) might be exinct among mammals . What we wrote (and consider correct) is: "*The combination of gracility, large size, and terminal rosette in thylacine crania has no modern mammalian functional analogue we are aware of…*"

However, authors themselves identify several modern canids that prey on small, quick vertebrates. These canids have crania that closely resemble the thylacine's.

Again, the point here was that individual similarities are unlikely to sum up to a full picture of functionality. We dedicated a paragraph to discussing potential cases of similarities and weighing the evidence for functional similarity on p. 16.

The authors carried out no video or biomechanical analysis to support their speculations about bite speed or strength. I don't expect the authors to perform these kinds of studies, as they seem beyond the scope of what the authors set out to do, but if it is the case that biomechanics and kinematics are beyond the scope here, then authors should put greater weight on caveats than they currently do (e.g., alternate hypotheses, discussed below).

Our core argument is that, all else being equal, larger skulls bite harder and longer jaws have faster-moving tips. This is not speculative, it is a mechanical necessity, as outlined in our comments to R5; but since it has cropped up a few times as a concern, we now expand on the lever mechanics and

related literature more extensively, particularly in the sections after reporting/discussing cranial size (p. 13-14).

Methodological Approach

This study is a standard morphometric analysis using a respectable comparative sample. The authors estimate growth trajectories for several dimensions of the cranium to assess the presence and nature of differences among a handful of canid lineages and the thylacine, and they use a geometric morphometric analysis to interrogate similarity to the thylacine's cranial shape among the wider sample. Using Procrustes distances to assess similarity is reasonable,

Thank you for this appraisal and we hope that this alleviates concerns about sample sizes by R1.

but it is worth explicitly stating that similar taxa will be, in most cases, distinct along different axes of shape variation (as another reviewer pointed out).

That is a good suggestion! We have added a comment to this effect on P. 6 (first paragraph).

Also, the Wheatsheaf index (a measure of strength of convergence) can be calculated with Procrustes distances, and that metric explicitly includes phylogeny (Arbuckle, 2014). Why not employ that index instead of the raw Procrustes distances?

This was such an interesting question and VW spent a few days implementing the code published by Grunstra et al. 2024 with their paper on afrotherians. However, we did not find the Wheatsheaf index an appropriate measure, at least not as defined by Arbuckle 2014. A detailed explanation of our code implementation and specific issues are at end of this response for your interest (and to share a fascinating analystical journey!), and also to show that we made every effort to engage with this suggestion.

We did, however, recognise that incorporation of phylogenetic information was a common theme in R1 and 5's suggestions, and was also required to facilitate our discussion of shape variation more broadly (rather than just thylacine vs. wolf). We therefore added an alternative presentation to the supplementaries that more clearly shows the phylogenetic distribution of Procrustes distances from the thylacine (Supplementary Figs. 3 and 4). This hopefully helps with the phylogenetic contextualisation of our results, which R1 was also requesting.

The allometric regressions that use individual PC axes and centroid size are perfectly fine in and of themselves for visualizing differences among groups, but these analyses are not sufficient to statistically tease apart differences in shape allometry among lineages.

As suggested by another reviewer, the ideal way to statistically test for such differences is to explicitly model the allometric relationship by lineage, as done in several papers by Polly, Adams, etc. In my line-by-line comments, I cite a couple of example studies that use such an approach. It seems like all the data necessary for such an analysis is present in this study, so the authors ought to take that step.

Thank you for this appraisal, and that is a fair comment (see below). Adding the allometry by lineage was no issue at all and we hope that the addition of our by-lineage allometric results (Extended Results; bottom of p. 9 onwards) are acceptable.

Formatting and Clarity of Writing

Formatting is generally fine, but the clarity of writing needs some work. This text suffers from many ambiguous ("hanging") demonstratives — "this", "these", "they", "it" and similar — that are unaccompanied by a subject. Authors should modify these sentences for clarity.

Thank you. We have gone through the manuscript and weeded out those sentences.

Also, the narrative that the authors settle on, that the thylacine's traits mean that there is a lost opportunity to shed light on some kind of totally unique behavior, doesn't make a whole lot of sense and isn't well supported by the data. I discuss this issue both above in my general impressions and below in my line-by-line comments, so I don't want to repeat too much. The bottom line is that if authors wish to speculate that the thylacine was particularly well adapted for a behavior, that seems reasonable, but they should emphasize that the point is speculative absent biomechanical studies that test the hypothesis.

Thank you for this appraisal, we have gone through the relevant section and improved our referencing for laws-of-physics conclusions and expanded on the mechanical rules that govern how acceleration and weight distribution work together to produce impact - see below for details and bottom of p. 13 for re-worked text.

They should also discuss alternative hypotheses. In spinosaurid dinosaurs, the terminal rosette has mostly been hypothesized to function as an adaptation for more surface area to better hold on to prey. Likewise, in modern crocodylians, any species can produce the same bite force regardless of skull shape (proportionate with body size; Erickson et al., 2014), so the rosette may have more to do with hanging on to slippery prey than withstanding greater stress at the tip of the rostrum.

There is surprisingly little testing about the biomechanical function of the terminal rosette that we are aware of. From what we have seen, authors have simply implied that the shape in some way aids in prey capture, presumably due to the expanded surface area. However, even at that superficial level the reason that it is in fact a 'rosette' is not commonly hypothesised on; i.e., that the rostrum proximal to the tip is narrower instead of the whole rostrum simply being as wide as the rosette. The closest we are aware of is in a recent paper by D'Amore et al. (2025; which is now added to our discussion): they hypothesise that a reason for the shape is the conflicting demands of prey capture and overcoming drag.

The bite forces Erickson et al. (2014) measured were for the distal end of the molariform dentition (essentially the crocodylian version of the carnassial). Skull shape across the molariform area is more or less equivalent across crocodylian species, so it is not unexpected to find similar bite forces in that area. However, it is the anterior rostra that really differ and the bite force in this area would also be expected to differ.

Ethical Considerations

The authors ought to declare a competing interest on behalf of author AJP.

Done, see below.

In this revision, the authors explicitly bring up ethics of researching the thylacine, given its historical and cultural relevance to the Indigenous communities of Tasmania. I would recommend the authors highlight their commitments in an Ethics/Inclusion Statement, which Nature Communications has room for, rather than in the main text (hopefully such a statement would get a separate hyperlink from the main text?). That said, why not start now, while this manuscript is still in revision?

Thank you for this guidance. This was a new paragraph in the last revision that VW felt strongly needed to be included after discussions with Indigenous biodiversity researchers. We would like it to be where it is most visible. We need a clear signal to the scientific community here in Australia, where there are next to no mechanisms for involving Indigenous custodians with research.

We suspect that the paragraph does not fit an Ethics and Inclusion statement, however. The Nature portfolio guidance notes that this is a disclosure document, and it does not appear lto allow for our extensive referencing of the local Indigenous context (one of the document's queries).

The first question for the disclosure statement ("Has the research included local researchers throughout the research process – study design, study implementation, data ownership, intellectual

property and authorship of publications?") is also clearly "No". But nearly all papers that use Australian animals and plants would have to answer this question in the negative (for example, the Grunstra et al. 2024 paper uses a platypus, which is also highly culturally significant). We are concerned that flagging our failure to engage with Indigenous communities in the statement will single us out by being pro-active in our admission that the field has to change. Note also that no policies, mechanisms, or guidance exist that we could follow in this case. VW recently published a paper, now cited here, discussing this in more detail - Weisbecker et al. 2025 in BioScience; and has since reached out to the Tasmanian museum, who have reacted with confusion and some contrition that no mechanisms exist for culturally appropriate research on their specimens.

Author AJP has a professional relationship with a scientific-and-investment endeavor that explicitly aims to clone the thylacine. That company, Colossal, states that author AJP's lab is leading their efforts to clone the animal. This manuscript currently makes the argument that the thylacine is more "unique" and a greater "loss to diversity" than previously appreciated, a narrative that could reasonably be interpreted by readers to be a bulwark of support for cloning the thylacine. These factors combine to create the appearance of an ethical conflict, and it would be a positive step toward the commitment to be transparent about the professional relationship with Colossal.

We had not thought about this – thank you. We have now added the following:

"Andrew Pask is funded in part by Colossal Biosciences, a company engaged in de-extinction and genetic restoration projects, including work on the thylacine. However, Colossal Biosciences had no direct influence on this project design, data collection, analysis, interpretation, or conclusions presented in this paper."

Line-by-line comments:

36: missing a space and comma after "observation"

Done. Sorry, EndNote was playing tricks with us and we had a few of these issues.

39: "...as it reflects"

"it" might refer to "the thylacine", "the thylacine's ecology", "comparative studies", or the "cranium". I think the "it" is supposed to refer to "comparative studies", but the meaning is ambiguous given the disagreement in count.

No, it refers to the cranium. This has been made clearer.

44-46: "...although marsupials display general trait suites..."

I don't understand how the "although" clause relates to the rest of the sentence. Do the authors mean that "general trait suites" are shared across all mammals or that these suites are particular to marsupials? Also, the "although" clause more appropriately would contrast the remainder of the sentence, but it seems like the final clause ("their cranial...") reflects back to the although clause. The first clause is then a sentence fragment and nonsensical: "Similarity between wolves and thylacines is especially notable because." I recommend rephrasing. "The similarity of wolf and thylacine is noteworthy partially because marsupial and eutherian morphology is so different: marsupials and placentals share some traits that relate to broad-scale dietary regimes (e.g., crushing molars vs. carnassial teeth), but a marsupial cranium is nonetheless usually statistically distinguishable from a eutherian cranium," or something like it.

This has been reworded. Thank you for the example.

49: "The radical departure towards..." (emphasis mine)

Treating "radical departure" as the subject of the sentence means that it is unclear whether the thylacine, marsupials, carnivorans, or some combination of the above is meant to be the subject of this clause. The

authors have not established that the cranial anatomy of the thylacine is "radically" departed from anything. The previous paragraph simply notes that the wolf and thylacine are similar and that the similarity is noteworthy. The previous paragraph also states that marsupial and placental crania are nonetheless statistically distinct, so is the thylacine an exception to that previous finding by other authors?

This paragraph lost a crucial piece of information in the last revision process, about the details of similarity between thylacines and canids. This has now been addressed ("*The thylacine's skull visually resembles that of the grey wolf, a feature … quantitatively supported by several morphological studies*", and we have toned down the wording too (p. 3, 3rd paragraph).

52: "Excitingly…"

I recommend the authors drop this kind of independent marker word throughout (also "intriguingly", "interestingly", etc.). If the sentence describes something exciting, then readers do not need to be told that they should be excited. Dropping these unnecessary words will reduce word count.

Done.

55: "If these…"

"These" what? Could be "aspects of thylacine cranial growth", "shared regulatory regions", "paraxial mesoderm tissues", etc.

This has been addressed by adding "…*this morphological similarity*…" (p. 3, l. 43)

56: "…they would provide…evolutionary, genomic, and developmental processes…"

But this study does not examine genomic or developmental processes. It does examine developmental cranial modules, I suppose, but not "processes". I recommend rephrasing about expectations for morphology.

We agree, this was not a good end to the paragraph. To improve on the narrative, we have now taken this sentence to the next paragraph to highlight that the excellent potential to assess these processes is currently constrained by our lack of knowledge on what function the shape actually represents. This also deals with the awkward start to the next paragraph in the next comment (p. 4, 2nd paragraph).

"Future evaluation of the genomic and developmental convergences between thylacines and canids requires a robust estimate of the functional significance of their similarities. However, recent studies revealed that thylacine crania display a mosaic of features that complicate the identification of canids with morpho-functionally similar crania"

59-60: "…the overall cranial similarity…morpho-functional detail."

This sentence is essentially redundant with the following sentence but is less clear ("translat[ing] well into morpho-functional detail" is awkwardly phrased). I recommend removing this sentence to save words.

See comment just above, this was an unnecessary and awkward duplication; the sentence has been replaced by the text above.

61: "significantly converging"

What does "significantly" mean in this context? Statistical significance? If so, why not just say "converging", cite the study, and save the word?

Done.

70: "distantly-related…sparassodonts"

But sparassodonts are metatherians, yes (Forasiepi, 2009)? I don't think that phylogenetic relationship is ever stated in the manuscript.

70 MY is certainly still a long time, but doesn't the shorter divergence time increase the chance that similarities are due to shared ancestry? Also, the hyphen between "distantly" and "related" is unnecessary because "distantly" is an adverb.

Yes, they are metatherians, we lost this information in the last revision and did not notice – sorry! The idea that sparassodonts and thylacinids share a common ancestral morphology has been tested and refuted in the papers we cite in this section. We clarify this now (p. 4, 2nd paragraph).

72-73: "This all suggests…analogue for the thylacine."

I recommend that authors rephrase the beginning of this sentence and turn it into an introduction for the paragraph. It is a good thesis statement.

Thank you – we have now re-written the whole paragraph including the introduction for the paragraph, and hopefully this improves the narrative.

75: "clearly convergent"

Do the authors mean "superficially" convergent? Isn't the point that we don't know how convergent they are?

This wording did not need to be there, and is now changed as part of our attempts to not focus on convergence and the thorny issues around this concept. But "superficially convergent" is not really a thing; either two organisms are convergent in having evolved a trait independently, or not. The question of our manuscript is less about the yes/no of this convergence, or how much convergence (this has been addressed multiple times), but rather what this means in morpho-functional terms. It is therefore appropriate to call overall cranial shape in canids and thylacines "convergent", as previous works have left no doubt that these shapes share a degree of similarity and arose independently. It is equally appropriate to also state that this overall similarity is not biologically meaningful because 1) it is due to a statistical averaging-out of disparate levels of similarity in the front compared to the back of the skull (as per PCA and Procrustes distance results) and 2) due to this and substantial anatomical differences (size, rosette, IOF) it also does not indicate functional equivalence (or convergently evolved function). But all this does not make the morphological similarity, or convergence, between canids and thylacines superficial – it just makes it just meaningless in a functional sense.

, 75-76: "querying individual patterns of similarity in isolation will not yield a useful perspective."

A couple of things here: one, what is an "individual pattern of similarity in isolation"? Do the authors mean a one-taxon-to-one-taxon comparison, or do they mean a single anatomical system, or something else? Second, are the authors saying that no valid conclusions can be drawn from this "individual pattern of similarity" (whatever it is), or only that any conclusions risk being limited in utility or specious? Those two interpretations are distinct, and I read the current incarnation of the clause as intending the former (i.e., no valid conclusions), which I would imagine the readership might push back on. The Figueirido and Janis papers cited in this sentence aren't that unlike the present study. Perhaps their conclusions are specious, but if so, the same risk must be considered for the present study.

Thank you for this insightful critique, this wording was not very specific. We have replaced it with a much more targeted sentence (p. 4, last paragraph):

"The differences between thylacine and canid crania make it difficult to infer thylacine predatory behaviour and functional ecology using canids as analogues. These differences may also confound the potential to link selection processes on cranial function with genomic and developmental similarities to canids such as grey wolves."

76: “They also hinder”

“they” what? Thylacines? Individual patterns of similarity? Convergent analogues?

This text is not removed as part of the paragraph re-write.

83: “This shows”

“This” could refer to either the preceding sentence in its entirety or to “large infraorbital foramina”, which is the noun directly preceding “this”.

Good point, this now reads (p. 5, l. 82) “*Our results confirm that the thylacine cranium is a mosaic of functional traits without apparent living mammalian analogue.. “*

91-92: “Our…analyses show…placentals and metatherians….”

I recommend rewording this sentence because the thylacine may have a unique combination of certain traits, but the results do show resemblance of the thylacine cranium to those of mid-sized eu- and small metatherians. That finding does not sound the same as “incongru[ity]” with the two clades.

This has been reworded.

99-100: “The aspect…variation.”

This sentence is a little hard to read, and I think it could be improved by simplifying to something like, “The thylacine resembles canids in having low scores on PC2.”

Thank you for this suggestion, this is now included and reads much better.

102-103: “…clearly important…visual impression suggests.”

Important how? I think the authors are trying to say that the separation is statistically “real” but exists on a PC axis that explains less variation than PC1. But readers could interpret the authors as trying to say that there is some kind of biological importance at play that isn’t stated. Also unclear is whether the “visual impression” is of the crania themselves or of datapoints on a scatterplot. Anyway, if there’s overlap between canids, thylacine, and some metatherians on PC1, how can the thylacine’s morphology be “incongruous” with both groups? There is something about the word choice that sounds like the discussion tries to have it both ways, i.e., the thylacine is both totally unique from but also relatively similar to extant mammals.

We have re-worked this paragraph extensively to stick more closely to the results. In particular, we changed this sentence to be more clear and neutral by simply pointing out (p. 6, 1st paragraph) that “*Our geometric morphometric analyses of thylacine cranial shape and a previously published comparative sample of 59 species, covering 14 families of faunivorous mammals (Fig. 1b–d; Supp. Fig. 1), agree with previous results that the thylacine’s overall cranial shape has shifted from the metatherian shape to be intermediate between canids and metatherians”*

105-106: “Thus…along PC1.”

I don’t really understand this sentence. If PC1 separates (roughly) dolicho- from bradycephalic crania (lines 96-97), then I don’t understand how the thylacine and Nimbacinus are “unique” along PC1. There are several datapoints near those two on PC1. Couldn’t one pick any two datapoints and say they’re “unique” from the others? It seems reasonable to say that the thylacine’s face is on one extreme of PC1, like some canids, but saying that it is “unique” seems meaningless in this context. If authors are simply saying that canids and the thylacine are “unique” from short-faced species, wasn’t that point already made in line 97? If I’m missing something, please clarify.

That makes a lot of sense, the word “unique” was not right and it was also factually not quite correct. We have phrased it to be more straightforward: “*Additionally, the thylacine and its Miocene relative,*

Nimbacinus dicksoni, have the highest PC1 scores except Andrewsarchus, separating them from the entire sample."

107-109: "…showing low distances of both canids and marsupial (sic) from the thylacine…smaller marsupial quolls."

Did the authors mean high distances? Don't low distances indicate similarity? In any case, the circles in Fig. 2A don't exhibit any kind of jumpiness or grouping; they gradually increase in distance from Nimbacinus to Didelphis. Again, if I'm missing something, please clarify, because I do not understand how these figures support "uniqueness" of the thylacine any more than they support uniqueness of, say, the Virginia opossum.

Small distances do mean high similarity. But our writing must have needed clarification because the list is not meant to be "jumpy", it is a list of species that are closest to the thylacine in Procrustes distance, ranked by cranial distances (in Fig. 2a) and mean distance of all partitions (2b). We have re-written the paragraph and captions to be clearer about this.

The figure uses the thylacine as the point of comparison (now clarified more extensively), which is not informative on the other species like *Didelphis,* other than its position relative to the thylacine. In that sense, however, *Didelphis* is definitely not unique, since it shows the same patterns of high neurocranial, low rostral similarity relative to the thylacine as the other marsupials in the figure.

The reason why the figures show the thylacine's" uniqueness" - just focusing on Fig 2a now - is that its overall, intermediate shape is a composite of a canid-like rostrum (turquoise triangles) and a marsupials-like neurocranium (orange).

However, we get a sense that the word "unique" is a stumbling point here, so we have gone through the manuscript and changed it with more neutral wording.

110-111: "Separating…neurocranium."

Doesn't this sentence, which identifies similarity with both canids and metatherians, contradict the current introductory sentence of the previous paragraph?

It does not. The thylacine's *overall* cranial shape shape is similar to both canids and metatherians. The thylacine's rostral/PA shape is similar to canids; the thylacine's neurocranial/MES region is similar to metatherians. We now clarify this further by adding "*, "Dividing the cranium into partitions shows that the thylacine's overall intermediate cranial shape arises from complex and at times contrasting sub-patterns within landmark configurations*"

135: "the concept of a one-sided convergence"

What is a "one-sided convergence"? Are the authors saying that other works suggested that the wolf/dog cranium experienced evolutionary stasis and the thylacine's lineage converged on that same shape? Authors need to cite some background here.

That was awkward wording and we definitely did not meant to suggest this, sorry. We find it important to combat the perception that the thylacine is an imperfect copy of the wolf, which is implicit in much of the historical discourse about marsupial-placental comparisons. But the whole paragraph was re-written to instead focus on the fact that the similarity between thylacines and canids arises from different cranial shape combinations in different canid tribes (see response to R3).

153-165: allometric effects discussion

I agree with the reviewer who raised concerns that authors should, if desiring to test differences in allometry among lineages, explicitly test that question. The individual PC-axes-versus-size regressions should not be relied upon as sole arbiters of allometric differences among clades, whether qualitatively or quantitatively,

as such plots of form space may be misleading. Instead, an explicit regression of multivariate shape versus size, followed by significance testing for differences among lineage-specific regressions, would be the statistically appropriate workflow (see Adams & Nistri, 2010; Cardini & Polly, 2013).

This has now been done and the results are included in the Extended Results section - see our comments to R5.

207-208: "suggests that there might be", "between where", the description of a trade-off

"suggests that" already indicates uncertainty. Can save words by removing "there might be". "Between where" is a bit awkward; consider "whereby" or "wherein".

Fixed, thank you.

"gracility is maximised relative to a constant absolute bite force" does not sound like a trade-off (it sounds like something has been maximized regardless of another trait). Authors explain the trade-off by (and in) the concluding sentence of the paragraph, but the current intro sentence isn't very clear. I'd also suggest that authors illustrate the trade-off, either with a figure of crania that are examples of the extreme ends of the trade-off or by briefly stating examples of such taxa here.

Prompted by R1 and R5, we have expanded on the relationship between cranial size, gracility, and bite force more extensively and this is now fully re-written with references to publications where size/shape trade-offs are explained and demonstrated using Finite Element Analyses (p. 13). We have removed the unnecessary reference to a trade-off to instead focus on the size *vs.* gracility relationships in the thylacine relative to specific canid groupings (p. 13, 2nd paragraph).

210-211: "Gracility and large…functionality."

This sentence is a bit tautological. A similar point is more clearly made later ("The thylacine cranium is thus…). I recommend removing or rewording this sentence.

This has been fixed; thank you.

225: "extremely pronounced"

Redundant; if the trait is pronounced relative to the entire rest of the sample, there's no need to say "extremely".

Done.

227: "Biomechanically, this moves"

The preceding sentence talks about how pronounced the trait is in the thylacine, so "this" could refer to either the general trait or to the state in the thylacine.

Agreed; fixed.

227-231: discussion of terminal rosette, resilience, and bite force

Has this idea been tested? If it has, a citation to the study should be here. If not, a citation to a reference text would be helpful. In spinosaurid dinosaurs, a terminal rosette was interpreted as an aid to holding food items.

As with the physics of jaw size, this sentence describes the mechanical properties of a structure that is elongated with a laterally widened, tooth bearing tip. The properties we describe will necessarily be attained by such a structure: rotational inertia at the tip is higher when there is more mass at the tip; tips also move faster the further they are away from the fulcrum. Greater mass at the tip and greater

speed of the tip necessarily lead to higher force output at the tip. The widening of the tooth-bearing part of the tip furthermore means just by the laws of physics that any local bite impact force is dissipated across a wider area at the point of bite at the canines, where it is widest. The spinosaurid rosette and dentition has also been described as adapted for fast, puncture-inflicting bites with little capacity for actual hard biting,as well as holding food (d'Amore 2025; now cited in the paper) purely based on these very rigorous first-principles physical effects. We have now made this more explicit (paragraph on p. 13-14).

232-234: "absolutely tall compared to canids", citations for this sentence

I don't understand what absolute height adds to the discussion here. Isn't the thylacine cranium absolutely larger in all dimensions than most canid crania? Is absolute height what matters for dispersing dorsoventral forces, or is it relative height?

No, this comment was in there for comparative illustration (some canids have crania as large as the thylacines', but none of them have a rostrum nearly as tall in absolute terms). But we have removed this sentence as it does not provide all that much information.

Also, the citations in this sentence focus on the other side of the amniote tree (specifically archosaurs), where skulls are built quite differently. Is the height rule generalized across amniotes (and/or tetrapods)?

This must be referring to the next sentence ("*This shape is well-suited…*") using references related to spinosaurids and crocodylians. One of the points of our paper is that the thylacine shares this altirostral shape with non-mammals, but there is no research on this in mammals because the shape is rarely found among mammals (see also Mitchell et al. 2024 as cited in the text). However, as with cranial size and the inertia of a wider tip, an altirostral morphology will have the same physical properties regardless of where it appears. Any structure that is tall and narrow will be best at distributing forces in the direction of the shape's apex and worst at resisting twisting forces.

246-273: colloquy about ecomorphological (intertaxon) comparisons with the thylacine

I agree with the reviewer who raised concerns about the "extinct predatory behavior narrative".

We have substantially toned this down, and made several clarifications to ensure it is clear that we are talking about a morpho-functional trait combination hat is extinct among mammals (see comments in the relevant sections).

How fast is a fast bite,

As explained in the paragraph above, the lever mechanics of a very long face in the thylacine mean that it must have had a considerably faster bite relative to the much shorter-faced canids of a similar size. We made this clear by adding in *"…fast (for its size)…"* (p. 14, l. 232)

and how similar does a mammalian terrestrial predator need to be for "useful" ecological or functional analogy?

These are different questions. *Ecological* analogy does not require much morphological similarity at all because morphologically dissimilar species can have similar diets or prey types. We also do not challenge the notion that thylacines had ecological analogues among carnivorans as hypercarnivorous consumers of small-to-medium prey. However, *functional* analogy requires sufficient similarity for crania to behave in a similar biomechanical way. Our paper shows that the thylacine does not have a living mammalian functional analogue in this respect.

Entelodonts were generally much larger (at least an order of magnitude heavier) than the thylacine, are presumed to have been omnivorous, and in some cases preyed on sheep-sized camelids (Joeckel, 1990; Sundell, 1999; Rivals et al., 2023). Long snouted entelodonts may have had a quick bite, as Sundell

suggested in their abstract, but it's not like large canids don't have a qualitatively quick bite (see, e.g., video of dog in Brassard et al., 2020). Similar story with mesonychids (size of large wolves or small bears, ~40-60 kg; Solé et al., 2017). Analogies with modern canids are arguably more useful because we can observe and quantify their behavior more easily than for an extinct species. Besides, the current study did not quantitatively assess cranial shape of entelodonts nor biomechanically test skulls to determine robustness to stress.

This is correct, and the collection of species in this paragraph was not to suggest that they all had the same ecology or cranial morpho-function as the thylacine. Indeed, one of take-home messages of our paper is that we only have canids to observe, but that this does not mean that all behaviours that are relevant to interpreting extinct predatory (or omnivorous) mammals existed in canids.

Bottom line, the point that the community should only approach analogizing the thylacine with modern canids cautiously is a valuable point. Likewise, discussion about aquatic animals with terminal rosettes is interesting, and it seems reasonable for authors to name-drop a few specific extinct taxa (not entire lineages) that look like the thylacine. But the results show that analogy between any two taxa will be imperfect. The suggestion of an "extinct predatory behavior" common to all these lineages is, at best, not based sufficiently in the study's findings.

Yes, that sentence was both unclear and constituted some overreach. We have rephrased it to *"It is therefore possible that the thylacine cranium was the last representative of morpho- functional mammalian cranial shape that has evolved and gone extinct multiple times in mammalian history" p. 17, l.298)*

280 and 282: "However" repeated in back-to-back sentences, sounds awkward

Fixed.

274-287: discussion about size of infraorbital foramen

A couple of concerns/questions here. Authors begin by a priori calling this trait "important" and "unusual", words that imply biological relevance, but all readers see is that the thylacine was a large-bodied marsupial with a relatively large infraorbital foramen (IOF). There is no explicit test of allometric slopes by lineages, so we don't know whether the size is a dasyuromorph thing.

We have toned down the beginning of this sentence. We did include (and referred to in the text) an allometric regression with phylogenetic prediction intervals in Supp. Fig. 4, showing that the thylacine is well outside the IOF size predicted for a marsupial of its size. Given the dataset and also the lack of clear association of the IOF with any behaviour (including whiskers; this association is not particularly strong), breaking down the statistical patterns further seems inappropriate. However, to improve clarity, we have now added a new colouration to highlight the position of the dasyurids and to also show that the relatively largest marsupial IOFs are in the thylacine and sparassodonts. We also now highlight lions/tigers and walruses in the graph, as requested.

Second, there are other interpretations that ought to be discussed. The infraorbital nerve is not the only structure transmitted by IOF; the infraorbital artery and vein also traverse IOF and might be a root cause of the large size. Even if large IOF relates to the nerve, the citations about vibrissae anatomy are a bit dated (Lyne reference needs title and year details). Has there been modern redescription of thylacine vibrissae? Overeruption of canines is also present in Smilodon (Riviere & Wheeler, 2005) and modern big cats (Smuts et al., 1978). The two placental datapoints in Figure 6 of the present study that are located near coordinates (5.25,2.4) are lion and tiger. If over-eruption covaries with size of infraorbital foramina, then large IOF of lion and tiger suggest that the link is not novel to thylacine and sparassodonts. How tusks and soft-tissue structures of the rostrum fit into this discussion is also worth considering.

Bottom line, this section would benefit from explicit analysis of allometry by lineage and discussion of alternate hypotheses.

We have now re-written this paragraph (on p. 15) to more explicitly discuss other potential interpretations of the IOF, taking on R5's excellent point on lions and tigers as well as tusks (now highlighted in the figure). But, as above, we strongly feel that an allometric analysis would put statistics over data. As we noted in the methods section, "Because several canid carnivorans are known to have very large IOF sizes, the aim here was to visually contextualise the thylacine's IOF size relative to this sample (rather than asking whether the thylacine was unusually sized relative to the sample)"

There has been no modern description of thylacine vibrissae, but we see no reason why the two references cited, in agreement with each other and explicitly comparing marsupial vibrissal patterns, are not credible as they are supported by imagery and from reputable mammalogists (Lyne and Pocock).

295: "maxillary branch of the maxillary nerve"

?? "of the trigeminal nerve"?

Correct! Fixed now.

293: "emergent property of a trait combination with no…equivalent"

I don't see how the thylacine can have striking cranial convergence with the wolf but also have a combination of traits with no modern analog. The phrase sounds self-contradictory. I don't have specific suggestions, but this sentence could use work.

The term "convergence" probably confused the picture. We replaced it with "similarity" and clarify that we refer to the overall cranial shape.

295-298: "far greater loss to global biodiversity"

These sentences are a little unfocused and could use work. Are authors suggesting that the thylacine was a keystone species?

On the question of functional equivalents, authors say we "lost an opportunity" to shed light on the thylacine, but aren't authors doing just that with this submission? What about biomechanical modeling, CT analysis, soft-tissue inference? Are authors saying that the community can't learn about or from the thylacine absent a perfect extant analog? The results show that the thylacine and certain canids are convergent. Perfect convergence in continuous traits is a mathematical ideal that no two taxa can ever meet (Stayton, 2015). Authors themselves drew parallels in this submission with modern crocodylians and extinct dinosaurs, members of an entirely different amniote lineage.

I'd like to hear the authors explain what they mean here, because it is demonstrably false that there is no longer an opportunity to understand this phenotype.

We wanted to articulate that, because thylacines are definitely not canid-like as previously thought, in 1936 the world lost the last living mammal where the function of a particularly unusual mammalian cranial phenotype could be observed in action (as opposed to merely reconstructing it using clearly inadequate comparative living sample). This makes a bigger difference to global biodiversity than losing, say, one of the 30 species of *Sminthopsis* or a fox species. We now use the more scientific phrasing of "adaptive distinctness" and re-wrote this section to be clearer that we mean live-animal observation (p.18, 2nd paragraph).

We are also not basing our argument on impossible ideals of perfect convergence. Instead, our evidence shows that the shape similarities between thylacines and canids are not based on

morphological *or* biomechanical convergence at all; that no faunivorous living mammal shares the thylacines' combination of shape and size traits; and that we therefore have no means of going beyond our paper's first-principle inferences about how this phenotype worked.

299-308: first colloquy about developmental evolution

Given that citation #51 did not even study the body part that the present study interrogates (they studied mandibles, not crania), I'd like to see a little more context in the sentence. It is not that surprising to find that the thylacine is not a perfect analog for species from a group that isn't especially speciose. It should be harder to match the thylacine's head with a carnivoran (~300 species of which authors sampled 69), than to match, say, a merlin's head with a passerine (6000+ species) — there still isn't a great match even with some strikingly convergent heads (e.g., shrikes).

This refers to the sentence "The apparent incongruity of the thylacine's cranial traits with an identifiable carnivoran ecomorph is intriguing because carnivorans display a large range of finely traded-off trait combinations". We are not sure additional context is needed because it is hopefully clear that our study is not about a convergence strawman set up to fail on the mathematical impossibility of perfect convergence. We re-wrote the sentence to be clearer about the mis-alignment between traits that confer specific functions to the thylacine cranium:

"The lack of alignment between the thylacine's functional cranial trait combination and any carnivoran ecomorph is intriguing because carnivorans are well-known to cover a large range of morpho-functional trait combinations, and show many-to-one mapping of different shapes to similar functions"

A full treatment of developmental constraints should include discussion of conflicting results. Some studies suggest developmental constraints on marsupial skeletal form (e.g., Sears, 2007; Kelly et al., 2019). There is no support for the claim that the thylacine is viewed as an "imperfect carnivoran".

Developmental constraints do not automatically equal evolutionary disadvantage; morphological integration, one example/manifestation of constraint, can facilitate exploration of phenotype space in some directions while it limits exploration in others (Felice et al., 2018).

We had already made R5's point that development might facilitate evolution in a particular morphospace (now re-written, but the original wording was"…it is plausible that marsupial development facilitates selection for cranial adaptations that are less likely to evolve in placentals"). We now also include the Felice et al. 2018 paper in the last sentence of this paragraph, discussing how development and contingency might facilitate selection of particular functional phenotypes.

We have also re-written this paragraph so as to not sound adversarial, but retain the overall point that it is important to consider the long history that has attributed a perceived lack of marsupial adaptability to developmental constraints. A full treatment of this issue is beyond the scope of this manuscript because it is a large and live field, but we added several key references on the marsupial constraint that direct the reader to the debate (paragraph p. 17 and 18).

Ultimately, I recommend rewording 303-304 to something more like "our findings are consistent with our hypothesis that the thylacine used quick, hard bites to subdue small prey items," adding "vice versa" to the end of the sentence in 305-306 ("For example, it is plausible…"), and generally refocusing this part of the discussion on development rather than the "evolutionary disadvantage/imperfect carnivoran" narrative.

"Vice versa" would have been a perfect addition, but this sentence is now totally rewritten (p. 17, lines 306-307).

310-312: "The extensive…selection has targeted similar genes…(e.g., the posterior cranial shape) or not." (emphasis mine)

Are authors saying that the genotype is somehow “seen” by selection? How do authors imagine that these similar regulatory regions evolved? Are they trying to point to pleiotropic effects? Or are authors merely stating that thylacines and wolves share more genes than just those linked to head anatomy?

This sentence used the term “targeted” in an inappropriately teleological way and probably caused confusion by glossing over some existing research results. We have now unpacked this to be clear about the selection evidence (paragraph 2, p. 18).

Lines 314-316 sound like the exact opposite of the sentence in 310-312. (i.e., Similar regulatory regions, in affecting early patterning genes in a similar population of cells, result in similar phenotypes in both thylacine and wolf.) What is authors’ scenario for how these regulatory regions became involved in this region of the head?

This text was not meant to be contradictory, but rather it duplicates information from the previous sentence. We have now amalgamated the two as part of the abovementioned re-write.

323-329: conclusion paragraph

Unnecessary “at” in the first sentence, hanging demonstrative “this” in the second and third sentences, and beginning of fourth sentence is awkward. This submission did not look at genetic basis of trait evolution, so I would remove that phrase from this paragraph or else rephrase it to make clearer that molecular work is a future direction.

Thank you for pointing out this lack of clarity. We also detected some redundancy within this paragraph, which is now shortened, explains “this” more clearly, and makes it explicit that we refer to future research (p. 18)

330-341: extended ethical discussion

Are authors planning to publish a popular version of this discussion alongside this submission? This section is a good reminder to the evolutionary morphology community, but it is buried at the end of a long, data-heavy discourse on anatomy. It would be valuable for this kind of commitment to be made more approachable to members of the public, as in a concurrent editorial/letter to the editor. Alternately, Nature Communications has space for an “Inclusion and Ethics” statement that is separate from the main text, so this paragraph could be better highlighted in that space.

As explained in greater detail above, an “Inclusion and Ethics” piece may not be appropriate because this section is less about disclosure, and more about acknowledging the comlete lack of guidance in the area. There is currently absolutely no oversight or mechanisms to address the issue we raise here.

An editorial/letter to the editor would be an amazing solution and might also allow us to bring in some of the Indigenous contacts we have been working with since! We will highlight this in our cover letter to see if this is an option.

That said, the authors should declare a competing interest. Given that author AJP has been leading a lab aiming to clone the thylacine for multiple years — and is deriving media attention from those efforts and through being a scientific advisor to the company (Colossal Biosciences) aiming at de-extinction of this taxon — I am surprised that no competing interests were declared. A major narrative of this manuscript is that the thylacine is especially “unique”. This narrative, including that the thylacine is a “greater loss to global biodiversity” than previously realized, could be viewed by readers as an attempt to draw attention to the value of cloning the animal. Transparently declaring a competing interest would address the appearance of an ethical dilemma.

This is now done (although note that most of AJPs work so far has stated the opposite - that the thylacine might be near-identical to wolves).

353-357: on re- or retrodeformation

Are the steps taken to re-deform specimens recorded somehow within the software, and could they be included with the submission? These steps constitute hypotheses of anatomy upon which, to at least some extent, the results turn. Likewise, are the paired Morpho landmarks included, or could they be? I may have missed these data when I went through the GitFront folders, but I did not see a folder named "Morpho".

We have now included the Morpho landmarks and script in the github repository to improve on repeatability. Unfortunately, the individual steps are not recorded in the software. We agree that this is not ideal, but as R1 points out the inclusion of fossils is important in studies like this so we deemed this step acceptable. Retrodeforming the specimens to shapes that are closer to the in vivo appearance (even though slightly hypothetical) is by far a better choice than landmarking deformed fossils which we know do not represent the in vivo appearance (a situation that unfortunately occurs in many studies).

358-363: on the Andrewsarchus artist impression

If the teeth weren't relevant for landmarking, why reconstruct them at all, rather than spending that time and energy on the rest of the cranium? Did the paleoartist start from scratch, or did they modify another animal's digitized cranium? These details are relevant for reproducibility.

The mesh of the *Andrewsarchus* cranium was sculpted from scratch in Blender using the figures and precise proportions from Osborn (1924) as a template. As the object was created with the intention of having it openly available for use (research and otherwise) and it being the creation of a skilled craftsperson, we considered it a waste not to reconstruct the cranium as best as possible, regardless if areas were not relevant to landmarking.

386-388: shape analysis and allometry

See earlier comments about testing allometry by lineage.

Now done and the methods are included in this section.

435: "functionalities of…evomap"

Is there a specific command or section of the code?

We now specified the functions in the text.

443-444: "are known to have extreme IOF sizes"

This claim should be cited, and I recommend rephrasing. "Extreme" is ambiguous and could be interpreted as either very large, very small, or both, so it's better to be explicit than use that term. "Because canid IOF size varies widely," "Because some canids have relatively large IOF," or something like one of these.

Fixed, thank you - and also added a reference.

Figures and Tables

The figures and tables are generally effective. The authors chose a color palette and general visual approach to the figures that seem broadly accessible. In Figure 4, the colors for the dingo, Canis latrans, and Lupulella adusta are somewhat similar under certain color-deficient proofing schemes. I suggest employing differently shaped data points for each species in that figure.

We are glad the pictures work! We have been slightly split between making figures not too busy while maximising clarity. A new colour scheme has now been implemented for Fig. 4 which specifically addresses as many colour-blindness types as possible.

In Figure 6, the arrowed lead lines are somewhat helpful, but there are other data points on this graph whose taxonomic identity would be valuable for readers to know because those taxa share traits with the thylacine but are not currently discussed (or are only briefly mentioned). The top-right datapoints, particularly the lion, tiger, and at least one pinniped (perhaps the walrus?), would be valuable to label, as would one or two of the smaller-bodied marsupials that have relatively large infraorbital foramina.

We are glad this was suggested, as we were split on this as well- the figure now has substantially more relevant species included, including lions/tigers, walrus, and the outlier mustelids. Consistent with the general unease about focusing too much on the wolf among the reviewers, we also removed the wolf arrow to keep the image clear.

Literature Cited

Thank you for the comprehensive list! We have integrated some, but not all of these references because we were already well over the maximum count for this paper. But we are discussing all of them as they are raised in the comments.

Adams DC, Nistri A. 2010. Ontogenetic convergence and evolution of foot morphology in European cave salamanders (Family: Plethodontidae). BMC Evolutionary Biology 10(1):216. https://doi.org/10.1186/1471-2148-10-216

Arbuckle, K., Bennett, C.M. and Speed, M.P. (2014), A simple measure of the strength of convergent evolution. Methods Ecol Evol, 5: 685-693. https://doi.org/10.1111/2041-210X.12195

Brassard C, Marilaine Merlin, Claude Guintard, Elodie Monchâtre-Leroy, Jacques Barrat, Nathalie Bausmayer, Stéphane Bausmayer, Adrien Bausmayer, Michel Beyer, André Varlet, Céline Houssin, Cécile Callou, Raphaël Cornette, Anthony Herrel; Bite force and its relationship to jaw shape in domestic dogs. J Exp Biol 15 August 2020; 223 (16): jeb224352. doi: https://doi.org/10.1242/jeb.224352

Cardini, A., Polly, P. Larger mammals have longer faces because of size-related constraints on skull form. Nat Commun 4, 2458 (2013). https://doi.org/10.1038/ncomms3458

Erickson G. M., P. M. Gignac, A. K. Lappin, K. A. Vliet, J. D. Brueggen, G. J. W. Webb. 2014. A comparative analysis of ontogenetic bite-force scaling among Crocodylia. Journal of Zoology 292(1):48-55. https://doi.org/10.1111/jzo.12081

Felice RN, M Randau, A Goswami. 2018. A fly in a tube: Macroevolutionary expectations for integrated phenotypes. Evolution 72(12):2580–2594. https://doi.org/10.1111/evo.13608

Forasiepi AM. 2009. Osteology of Arctodictis sinclairi (Mammalia, Metatheria, Sparassodonta) and phylogeny of Cenozoic metatherian carnivores from South America. Monografías del Museo Argentino de Ciencias Naturales 6:1-174.

Goswami A, Marcela Randau, P. David Polly, Vera Weisbecker, C. Verity Bennett, Lionel Hautier, Marcelo R. Sánchez-Villagra, Do Developmental Constraints and High Integration Limit the Evolution of the Marsupial Oral Apparatus?, Integrative and Comparative Biology, Volume 56, Issue 3, September 2016, Pages 404–415, https://doi.org/10.1093/icb/icw039

Joeckel RM. A functional interpretation of the masticatory system and paleoecology of entelodonts. Paleobiology. 1990;16(4):459-482. doi:10.1017/S0094837300010198

Kelly E M, J D Marcot, L Selwood, K E Sears, The Development of Integration in Marsupial and Placental Limbs, Integrative Organismal Biology, Volume 1, Issue 1, 2019, oby013, https://doi.org/10.1093/iob/oby013

Rivals F, Belyaev RI, Basova VB, Prilepskaya NE. Hogs, hippos or bears? Paleodiet of European Oligocene anthracotheres and entelodonts. Palaeogeography, Palaeoclimatology, Palaeoecology. 2023;611:111363. doi:10.1016/j.palaeo.2022.111363

Riviere, H.L. and Wheeler, H.T. (2005), Cementum on Smilodon sabers†. Anat. Rec., 285A: 634-642. https://doi.org/10.1002/ar.a.20199

Sears KE. 2007. Constraints on the Morphological Evolution of Marsupial Shoulder Girdles. Evolution, 58(10), 2004, pp. 2353–2370. https://doi.org/10.1111/j.0014-3820.2004.tb01609.x

Smuts G. L., J. L. Anderson, J. C. Austin. 1978. Age determination of the African lion (Panthera leo). J Zoology 185(1):1-146. doi:10.1111/j.1469-7998.1978.tb03317.x

Solé, F., Godinot, M., Laurent, Y. et al. The European Mesonychid Mammals: Phylogeny, Ecology, Biogeography, and Biochronology. J Mammal Evol 25, 339–379 (2018). https://doi.org/10.1007/s10914-016-9371-8

Stayton CT. The definition, recognition, and interpretation of convergent evolution, and two new measures for quantifying and assessing the significance of convergence, Evolution, Volume 69, Issue 8, 1 August 2015, Pages 2140–2153, https://doi.org/10.1111/evo.12729

Sundell KA. Taphonomy of a Multiple Poebrotherium Kill Site—An Archaeotherium Meat Cache. Journal of Vertebrate Paleontology. 1999;19(sup003):79A. doi:10.1080/02724634.1999.10011202

Reviewer #5 (Remarks on code availability):

The R code that I reviewed (on GitFront) looks reasonably well annotated and appears to work.

Glad it was accessible and we have re-run all code before publishing it. So it should continue to work.

Extended explanation of our decision not to use the Wheatsheaf index:

1) **The phylogenetic distance matrix approach in Arbuckle 2014 (scale to a length of 1, then derive variance-covariance matrix of expected trait variation) creates a matrix where all distances between marsupial vs. placental pairs are zero (i.e. maximum distance, indicating traits are not expected to be affected by co-variation in these species pairs). The flow-on effect when using Arbuckle's phylogenetic correction formula is that all same-clade phenotypic distance pairs are weighted to small values whereas marsupial-placental phenotypic distances remain nearly untransformed, which in our view unduly skews the distances compared to the raw phenotypic distances (plotted against corrected distances below; note that the overall pattern of thylacine-placental similarity remains identical and thus confirms our current results). With correction, even the totally blunt-faced *Hepailurus yagouaroundi* is closer to the thylacine, which is one of the most gracile-faced species in the sample - so clearly this is not a biologically meaningful operation:**

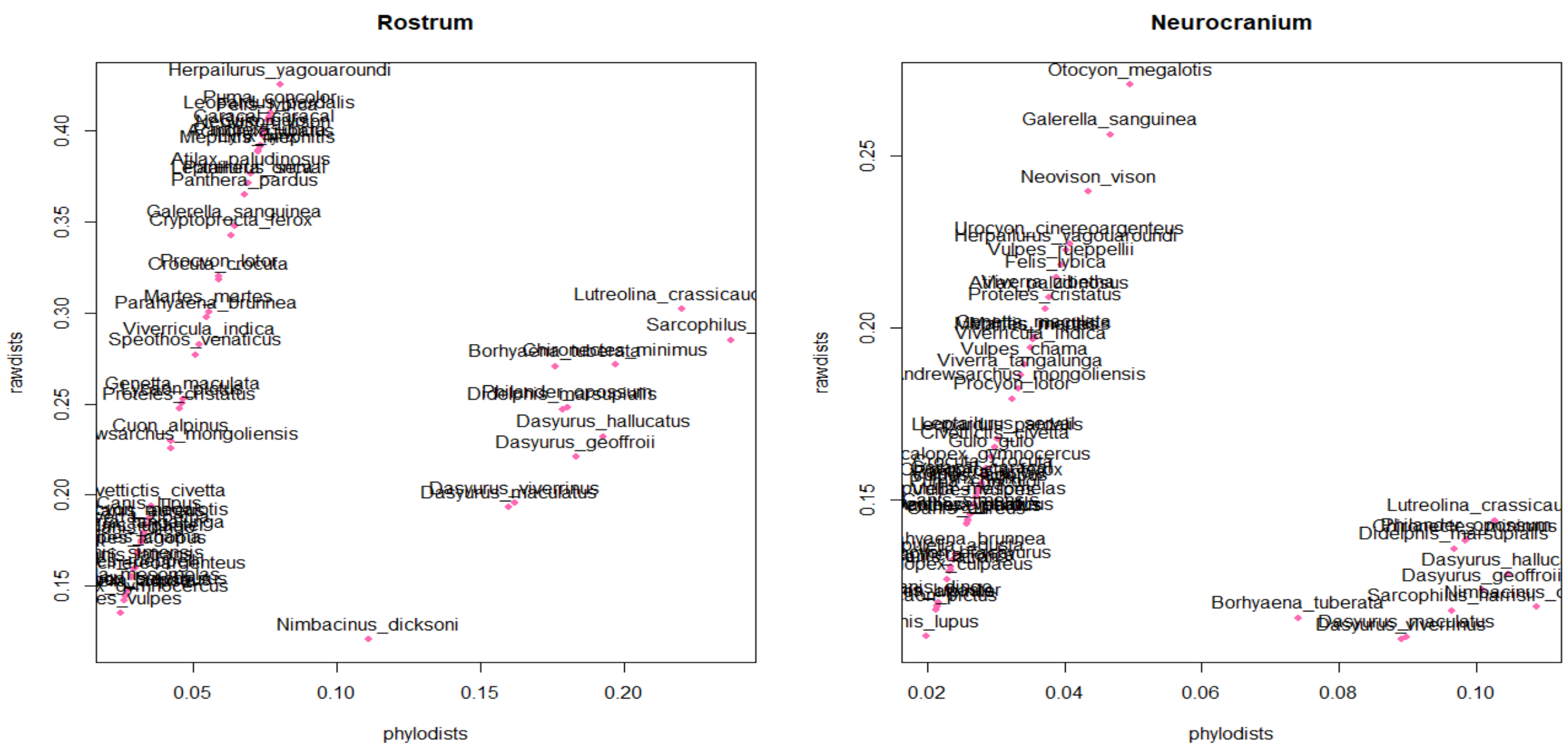

We see a similar effect in the corrected distances of the Grunstra et al. paper, but it is much less pronounced because the phylogeny has a single-species outgroup (so there is only one line of zeroes, between the platypus and other mammals), and a much more even transformation of distances. Playing with phylogenetic sub-sets, we also observed that all phylogenies with a split at the base (like ours, or including canids vs. feliforms) produce this effect of zeroes between all pairs, whereas a more ladderised phylogeny (e.g. canids, feliforms, with *Andrewsarchus* included) does not.

2) The apparent phylogenetic over-correction might be exacerbated by an issue with the trait standardisation which cannot be done for 3D data. Arbuckle (2014) intended the phenotypic trait distances to be "standardized for variance by dividing by the standard error of the trait across species". There is no equivalent operation for this for Procrustes coordinates (the superimposition step is a scaling, not a standardising step). It might technically be do-able by using principal components as individual traits to be standardised, but this would in our view over-process the data. We suspect that use of coordinate data might make the method vulnerable to issues like ours (although they obviously provide clear information under the right conditions, as the Grunstra paper shows; and we also got OKish results, just not publishable as rigorous analyses, see below)
3) Arbuckle also intended the Wheatsheaf index to be used only after convergence is established through other statistical means. Based on Rovinsky's (2021) results, this allows us to test for convergence between the thylacine and canids. However, this means that we only have two focal groups - the related canids including the unrelated thylacine *versus* all other species. The method then asks if the mean of the phylogenetically corrected distances within that group is lower than in the non-focal groups. But averaging twenty canid values with one thylacine value will not provide good information specifically on the canid-thylacine pair.
4) We had the impression that R5 suggested replacing the Procrustes distances with Wheatsheaf indices for each pair in our sample. Because of the requirement to previously demonstrate convergence, this does not appear to be appropriate, at least as intended by Arbuckle. Nevertheless, VW assessed what would happen if, instead of sticking to Arbuckle's specific guidance, a Wheatsheaf-like ratio was calculated for thylacine-wolf and thylacine-canids, relative to marsupial-wolf, wolf-marsupial, and marsupial-canid pairs (the latter with the focal species removed from calculation of the mean distance calculations). The results for a few pertinent comparisons are as below (percentages indicate how much greater the similarity between a pair is compared to the comparative group, as also done in Grunstra 2024):

	Thylacine-Canidae WI	Thylacine-Canidae %	Thylacine-Wolf WI	Thylacine-Wolf %	Wolf-Marsupials WI	Wolf-Marsupials %	Wolf-Thylacine WI	Wolf-Thylacine %
Skull	1.197	0.165	1.285	0.222	1.102	0.092	1.169	0.145
Rostrum	1.241	0.194	1.24	0.193	0.947	-0.056	0.927	-0.079
Neurocranium	1.175	0.149	1.267	0.211	1.308	0.236	1.407	0.289
FNP	1.306	0.234	1.748	0.428	1.135	0.119	1.489	0.328
Pa	1.094	0.086	1.166	0.142	0.991	-0.01	1.046	0.044
Mes	1.17	0.145	1.495	0.331	1.263	0.209	1.6	0.375

This essentially confirms the patterns we observed with just the raw distances: Wolves are much more similar to the thylacine and marsupials as a whole in the back-of-skull neurcranium/Mes relative to the front-of-skull rostrum and FNP. We find this interesting as a thought experiment, but do not suggest to enter it into the paper because of the methodological issues above. Happy to share the code or discuss further though - it's been an intriguing exercise!

REVIEWER COMMENTS

Reviewer #1 (Remarks to the Author):

I have now carefully reviewed this new version of the manuscript. However, the main, conceptual, issues that I noted since the first version are still there. Hence, I am not going into a point by point reply, as at this stage it is no longer necessary.

To make it short, the authors do not have enough results/data to support the very big claims presented in the manuscript. Furthermore, too many doors remain open suggesting that adding more in deep analyses would have been beneficial.

We have responded in-depth to all the suggestions (several response pages in two revisions, and adding several new analyses and figures) to address Reviewer 1's concerns. This included extensive allometric analyses in the first revision, which we expanded on in the second and third revision after they were picked up by R3 and R5 as well; we also provided substantially re-worked Procrustes analyses with two additional supplementary figures.

We also responded with two in-depth re-writes of the manuscripts to 1) ensure the focus was not, as R1 initially assumed, on testing convergence and 2) adding substantial caveats and clarifications. These were sufficient for the other reviewers, who were initially also quite critical of this manuscript.

The manuscript has had extensive literature and more detailed explanation added in the last two revision cycles to accommodate R1s concerns, but we cannot see how we can improve any further. We are repeating summaries of our responses from the last two revisions below, but if this is not deemed satisfactory, we do not believe that a resolution can be achieved by another review round.

The reworked abstract epitomises these issues. The authors did not provide any biomechanical analysis (it would have been possible even with linear measurements) despite claiming that the thylacine has "substantially different biomechanical properties". Here, trying to understand why the thylacine was having such a big head and big bite when it is widely assessed that its skull was poorly adapted to support a strong bite, would have provided a solid ground on which build a robust inference about the thylacine uniqueness. Again, providing a more complex adaptive scenario (supported by data) could have been great, rather than using a mere proxy.

As noted in the previous two revisions (particularly the last one), the reasoning we use to come to our conclusion that thylacine skulls were biomechanically unique is grounded in simple lever-mechanics and there is simply no reason to assume that these do not apply. The main points we noted and extensively clarified in the two revisions (to the satisfaction of the other reviewers) are:

1) All else being equal, bigger skulls will be able to withstand higher levels of strain compared to smaller ones of the same shape. For the thylacine, that means that its more fox-shaped snout would have withstood greater bite forces than the snouts of foxes because of its larger skull.

2) Longer jaws exhibit higher jaw closing velocities at the tip than shorter jaws because the speed will increase with increasing distance from the pivot point (jaw joint)

3) Tall, narrow snouts shapes are better at dissipating such forces along a dorso-ventral axis and worse at resisting torsion than snouts approaching subequal height and width.

In response to R1, we had extensively clarified this and particularly pointed to two publications of our own, where 1) is proven via *in silico* experimentation (Mitchell et al. 2025) and 2) and 3) are explained in detail (Mitchell et al. 2024).

Mitchell, D. R., S. Wroe, M. Martin, and V. Weisbecker. 2025. Testing hypotheses of skull function with comparative finite element analysis: three methods reveal contrasting results. *J. Exp. Biology*.

Mitchell, D. R., E. Sherratt, and V. Weisbecker. 2024. Facing the facts: adaptive trade-offs along body size ranges determine mammalian craniofacial scaling. *Biol. Rev.* 99:496-524.

Also, in Figure 3a other species display large residuals, with two marsupials showing the largest. Is this an emergent property that can be observed in other taxa? Is it shared with placentals (different canids show large residuals)? Or is it unique to thylacine? Here, having a larger (and new) sample could have been helpful.

We are not sure how investigation of these queries would support our question of whether similarities between thylacines and canids are indicative of similar hunting adaptations of the skull. But here we comment on each of these sub queries:

Is this (large residuals) an emergent property that can be observed in other taxa?

We are unsure what kind of emergent property a relatively large skull would indicate in this context. But marsupials with large residuals (really just one, which we highlighted in the figure – the much smaller opossum *Didelphis marsupialis*) do not have the overall similarities with canids that thylacines have (Fig.1), have a very differently shaped snout compared to thylacines (Fig.2), and show overall less cranial shape similarity with thylacines than a large number of canids and dasyurids (Fig.2). The unusually large size of the opossum suggests that the opossum could bite harder than would be expected for an animal of its body mass and skull shape, and this indicates an under-appreciated disparity of relative cranial sizes in other marsupials that makes it an interesting subject for a separate study. We had already highlighted this (p.18, l.300) but there is no reason to elaborate in the present paper about the thylacine.

Is it shared with placentals (different canids show large residuals)? Or is it unique to thylacine?

No canids share the spectacularly large residual of the thylacine or the opossum; and the thylacine is outside its phylogenetic prediction interval. It is known that canids generally have larger skulls relative to body size than other carnivorans – particularly felids and mustelids – due to their longer rostra (e.g., see Van Valkenburgh, 1990). The thylacine still clearly exceeds even that range, falling outside the confidence interval produced.

Van Valkenburgh, B. 1990 Skeletal and dental predictors of body mass in carnivores. In: Body size in mammalian paleobiology: estimation and biological implications. Cambridge University Press. Pp 181-205

Here, having a larger (and new) sample could have been helpful.

As explained in detail in the previous revision (and manuscript), our dataset was specifically chosen to cover the range of carnivoran cranial variation that might include ecological equivalents of the thylacine. As we noted in our last response, this because the manuscript's question was whether thylacine skulls were really as comparable to canids as has been claimed. As also already noted, an extended sample is either not possible (e.g., we included all medium marsupial carnivores – quolls - save two rare New Guinean species with no skulls available), duplicative (more foxes, jackals or cats are unlikely to change the patterns we see), or would introduce irrelevant noise (e.g. including huge-skull large-prey specialists like the largest of cats or bears).

In the abstract (and in the title) it is stated that the thylacine was having a fast, high-impact bite. But no data support it. Here, you could have simply measured the jaws mechanical advantage (a couple of linear measurements as noted above) and compare it (within a phylogenetic context) with other species to understand whether the thylacine really stand out or not. This has been done by multiple authors in multiple venues. Again, the data provided are not sufficient.

The last sentences are not appropriate, you do not have genetic data or performed genomic analyses.

We will have to remain in disagreement about whether we have data to support this statement. But it is not correct to expect that the mechanical advantage of the thylacine should "stand out" – quite the opposite: we show that the rostral region shape of the thylacine is most similar to that of species whose long snouts are known (as per literature cited) to give them a low mechanical advantage but greater jaw-closing speeds. Thylacines are therefore expected to show the same pattern (and, as we note, our linear scaling data confirm this – see Fig. 4b where the length of the thylacine's palate is relatively longer than all but the two smallest species). Moreover, one of the main points of the paper is that we identify specific traits of the thylacine that counteract this low mechanical advantage:

1) its large overall cranial size, which results in higher bite forces by physical necessity and

2) its laterally wide and dorsoventrally tall snout which dissipates forces dorsoventrally, again a well-accepted phenomenon by first-principles physics.

Neither of these two traits can be accommodated by simply assessing for lever mechanics. The physical properties of these traits are established beyond doubt both by first-principle mechanics and the expansive literature we cite in support. And in combination, they are crucial to characterising the differences between thylacine and canid cranial biomechanics.

Again, the data and the methodological framework presented here are too thin to provide a meaningful advancement of our knowledge of this extinct species.

I am sorry I cannot be more positive, but I hope that the questions presented above could serve the authors to provide a better scientific design and to stress out their conclusions.

Reviewer #3 (Remarks to the Author):

In this second revision the authors have implemented new analyses (allometric comparisons) and visualizations (phylogenetic visualizations of Procrustes distances) following the comments of other reviewers, and extensively rewritten the manuscript to incorporate those new results and address several concerns raised in the previous iteration. I was already satisfied with the previous iteration (barring some minor comments) and the current version is even better. I am particularly happy with the new discussion around canids in general (and Canina in particular) instead of focusing on the grey wolf. I'm glad that some of my suggestions were useful for framing this new narrative. I also find Suppl. Figures 3 and 4 much easier to interpret than Figure 2 + Suppl. Table 1 (plus they're gorgeous), so I wanted to thank the authors for providing both types of visualizations.

Thank you very much! It has been a pleasure to develop this with R3s help.

However, I feel that some of the other additions might be problematic, particularly after reading the comments of R#1 and R#5 to the previous iteration. Except for point (1) in the list below, I don't think any changes are needed on the other topics, but I do believe they're worth mentioning.

1. Allometry

Note that, in addition to the queries from R2 below, we also realised that the PC-by-PC analysis and the new allometry comparisons between clades are probably usefully backed up by individual analyses of allometry for each clade we tested. This is now a new table in the Extended Results (Table 3); the analyses show that allometry is mostly non-significant for all partitions within feliforms and caniforms in a pgls context, but even assuming that phylogenetic correction "over-corrects", procD.lm linear modelling suggest divergent levels of association between size and shape in the different partitions and clades.

The additions to the Expanded Results add more depth to the study, so they are very welcomed. However, while the authors added them to address reviewer concerns about not incorporating size into the comparisons, the way that they are handled compounds on the issue instead of solving it. I have made several comments on the Supplementary Data file (please check attached file with tracked changes), but my main concerns are repeated here:

- the regression without Andrewsarchus is not "nearly non-significant". On the contrary, it's a better fit than the regression including that species (as shown in Extended Results Table 1). This comment was ignored on the previous iteration and needs fixing.

Oh no, sorry! This was a complete mistake, obviously the reason for excluding *Andrewsarchus* was the poor fit of the regression residuals with it included.

- the interpretation of the ANOVA tables on Extended Results Table 2 makes no sense. Why would a significant interaction term negate differences in clade means when the clade term

is also significant? Each significant model term can and should be interpreted on its own, as it explains a fraction of sample variation.

And

- the previous point bleeds into the follow-up analyses, as the authors only test for differences in clade means when the interaction term is not significant, and when it is they only test for differences in slopes. Differences in clade means should be tested in all partitions, as the clade term is significant in all of them.

We have now added means difference testing for all comparisons by creating a full landscape table with slope magnitude, angle, and mean differences all in one (Extended Results Table 5). VW would personally not do this but her concerns are not so serious as to make us insist. It is technically not appropriate to include mean comparisons in the presence of significant interaction terms because the mean (i.e., projected intercept) for a clade 1 relative to a clade 2 differs depending on size. In an extreme scenario, the mean for clade 1 can be below clade 2 at small and above clade 2 at large sizes and the slopes cross over in the middle. A visual from a web page :

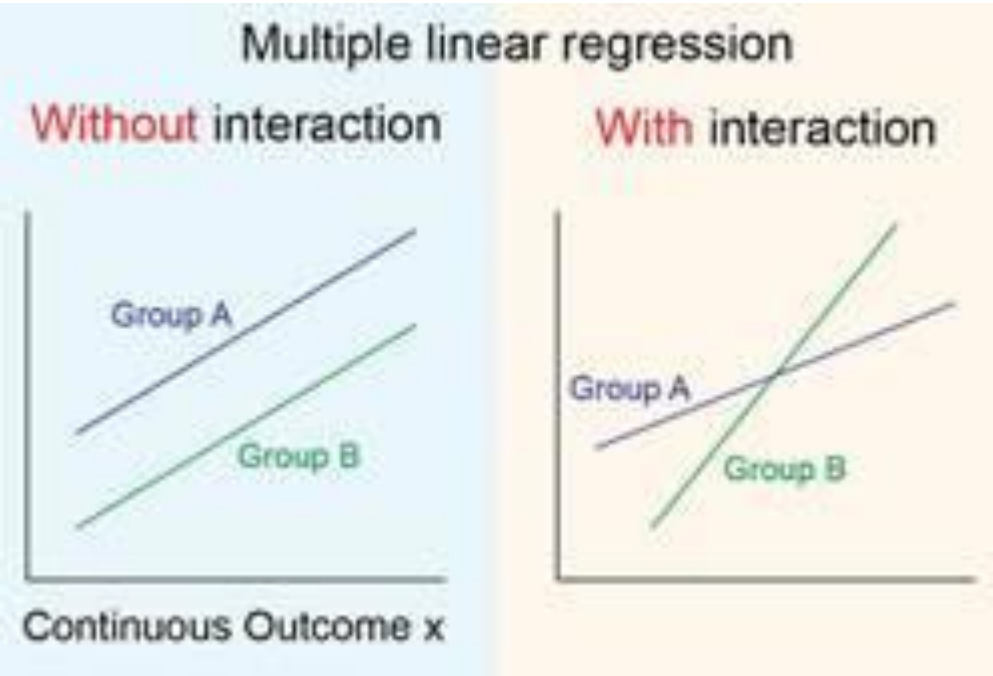

Thus, if we were to ask if there are different clade means in a model of shape against size and clade plus interaction term, we need to have parallel slopes, i.e. no significant interaction term. However, an argument can be made that small interaction terms are not too relevant if overall means differences are clearly much larger (e.g. if the two groups are clearly separate along y and/or slopes converge at values that are well outside of the samples' range).

Looking at the effect sizes/Rsquareds, this point can actually be made for our analyses because differences between clades clearly have far more variation explained by the size:shape interaction (size itself is also never a strong predictor - as we expected all along). So we can assume that the effects of scaling and interaction are not a major confounding influence on the result and run the pairwise mean tests of shape relative to clade. We have now added all the means comparisons and write an explanatory text for this in Extended Results Table 5.

Furthermore, the authors should specify whether they are testing for differences in slope magnitude (amount of changes per unit size) or slope orientation (whether shape changes occur in different regions of the shape space and thus represent different changes), as the pairwise() function allows for both tests. Based on the authors providing distance values, I would assume that they're testing for differences in magnitude (I haven't checked the script for this, sorry), but it needs to be clarified. Please note that the differences in slope orientation are likely what R#1 was referring to when suggesting that the authors could run

separate pooled regressions (e.g. separating Canidae, Felidae, etc.) and test whether these resulting slopes were convergent, divergent or parallel."

That is a nice addition, thank you. We have added the differences in slope angle (which we assume is what R3 means by "orientation") to the Extended Results Table 5. Further, also including R5's suggestions of deformation plots (see below) further summarises the biological reality of the slope differences.

- "Plotting the regression scores (i.e. the part of the variation related to allometry) of the Procrustes regressions against centroid size illustrates the lack of allometric signal in the similarities between thylacines relative to canids (and vice versa) in the Principal Components Analysis and Procrustes distances of the main mansucript." I don't understand what the authors mean here. If all the models in the Extended Results section have a significant size effect, why mention here a lack of allometric signal? This needs to be corrected or better explained.

The sentence referred specifically to allometric signal *in the similarities between thylacines relative to canids*…. , not overall allometric signal.

But to improve clarity and detail, and because it fits with the issue of means comparisons anyway, these lines are now re-written to expand on the *presence* versus *effect size* of a significant allometric signal. Specifically, differences in shape between clades explain 3-6 times more variation than size or size:clade interactions, meaning that the clade term all by itself effectively dominates the variation compared to the relatively minor allometric effects. An analogous situation would be if Group A and Group B above also had different slopes, but A had much higher y values so that the slopes never cross over within the x boundaries.

2. Colossal Biosciences
Now that R#5 has brought up AJP's connection to Colossal Biosciences, I can see why he raised issues with it, as knowledge of that fact adds negative connotations to some of the paragraphs in this manuscript (which suddenly feel as a potential publicity pamphlet for that company), particularly after the alleged prosecution by Colossal Biosciences of researchers actively vocal against their claims of de-extinction. However, the thing is that such companies and certain elements of the public are not going to stop using fragmentary pieces of written material to back up their claims, regardless of whether they actually do back them within their original context. With that in mind, while I appreciate the conflict-of-interest waiver for that author, in my opinion it will act less as an ethics statement and more like a potentially harmful statement towards the validity of this study. The Editor might consider removing it.

We are neutral on this point. This collaboration well pre-dates AJPs involvement with Colossal and we are just happy to do the right thing, whatever that is deemed to be.

3. Final paragraph
Personally, at first I saw this paragraph as the disclaimer that I hear in every conference presentation from Australian researchers, so I mostly ignored it. I did so on the basis that most times I've heard it, it sounded very void, so I treated it as a forced acknowledgement. But in this new iteration the authors make clear (at least to me), that they do care about the ongoing mistreatment of Indigenous peoples by colonizers, which I admire. I am not sure that a manuscript with a hard word limit is the best platform for this statement, but I will

support them in doing so (unlike other reviewers, it seems). Well done. However, one thing I don't like about the statement is that, by focusing on the harm being done to the peoples, it reads as if the fauna and flora "collected"were just cultural pieces to exploit (akin to the British Museum's – and others – pillages) instead of living units of biodiversity and pieces of an ecosystem that was damaged (sometimes irrevocably) in the process. To me, it perpetuates the idea that fauna and flora within the borders of a Country belong to its inhabitants instead of being their responsibility. In a manuscript about a species driven to extinction by colonizers, maybe that should also be a big point in the closing paragraph. Just my thoughts though, and please take this in the positive – this paragraph made me reflect on the situation, which I believe is its main goal. Again, good job.

Thank you for this positive feedback! And 100% agreed on the notion of custodianship, rather than ownership. This is an important issue because "Traditional Owners" of Country generally consider themselves as custodians, so there is some pushback to the ownership concept. But for this reason, this paragraph refers to data sovereignty and Indigenous Cultural and Intellectual Property Rights (ICIP), with no explicit mention of ownership (just removal without permission).

Beyond commenting on these topics and the annotated Supplementary Data file, I have added listed line-by-line typos and such at the end of this document.

Finally, to reiterate, once the points mentioned in (1) are addressed, and the minor comments and typos reviewed, I would consider the manuscript ready to go. I do not request any action on points (2) and (3) besides maybe read the authors' thoughts on them, continuing with keeping the dialogue open. And on that note, nothing more to say here in reply to that section of the rebuttal except that I really enjoyed this dialogue, as I think both the authors and I have reached a "common" understanding of these topics. Quotation marks because our opinions might still not be the same, but I believe we both arrived at a sense of understanding with each other's opinions.

I wish that this was more the norm in manuscripts reviews with multiple revisions and not an unusual outlier. Thanks for letting me be part of this journey, and again congratulations on a job well done.

Thank you very much for your thoughtful engagement with this paper. The comments truly (not just as a matter of saying the right thing) improved the manuscript substantially and it is a far more mature paper now thanks to this dialogue.

Thanks also for going through our code!

Eloy Gálvez-López (anonymity waived)

L55: I think "of" is missing after "perception". **Fixed**

L68-69: From this sentence I gather that dasyurids do not hold their prey until it's dead as felids do? If that's the case, maybe "penetrate-and-release bites" would be a better description (relative to the degree of detail given for canids and felids)? **Great point - we have changed this to "repeated penetrate-and-release-bites".**

L71: Delete extra space before the reference in "canids 15".**Fixed**

L107: "are" instead of "is"? The subject of this sentence is unclear (the plots? the whole previous phrase?).**Correct, thank you! Fixed**

L135: Delete “three” or add “genera” to “these three canids”, as there are more than three species in the Lycaon/Cuon/Canis lineage.**Fixed, deleted “three”**
L144-145: Capitalize “developmental” after “b,” to match format after “a,”.**Done**
L172-175: Capitalize “phylogenetic” after “a,” to match format after “b,”.**DONE**
L191-194: Are they OLS regressions or RMA regressions? **RMA! Great catch!**
L192: I think “width” is missing after “carnassial”. Or better, write “width across carnassials” instead. **The sentence has been clarified to state “...and cranial width across the canines, the carnassials, and zygomatic arches”.**
L270-271: Verb missing after “This”? **Added “is”**
L272: B in “Fig. 3B” should not be capitalized to match the rest of the manuscript.**Fixed**
L284: “Common Opossum” should not be capitalized to match other species names in the manuscript.**Correct! Fixed**
L291: Double space after “vertebrates’”? **Fixed**
L329: Period missing after “craniofacial evolution71”.**Fixed**
L375: Delete extra dot after “Fig”.**Fixed**
L387: I don’t think “arbitrarily” is an adequate qualifier on how PCA axes of variation are calculated. I would recommend deleting that word. **done**
L402-404: I have to agree with other reviewers that choosing just 10 PCs and regressing them separately against size is not best practice, and the authors should have analyzed simultaneously all the PCs in each partition.
From the script I gather that all the pgls regressions of PC1 to PC10 vs logCS of all partitions are carried out separately in a loop and then packaged into a summary table for inspection, so I assume not checking all the PCs is just a matter of how gigantic that table would be.
As a simpler alternative, I would recommend using a single pgls model of all PCs vs logCS and then using eta_squared() from the effectsize package to obtain η2 values and 95% confidence intervals for each PC in that model. From that function’s output it’s very easy to check which PCs are significantly related to size (which PC has CI_low > 0.00) and how big the effect is. Please note that I have only used this with the lm() function, so it might not work with model fits from pgls(). It should, but I haven’t tested it recently.

We are sure that many lower-order PCs also would also be associated with size, but this would stretch the (already very critically received) analysis too far. This analysis was the original one and asked if allometry is present in more than just the first PC, i.e. whether several axes of variation are explained by size. The use of ten PCs may be arbitrary, but it is not a rule-breaking mistake and does answer our question 100%. Further PCs are not needed to make the point and the message arising from the result is now extensively expanded on in the new allometric analyses. I hope we have shown that we are not averse to exhaustively accommodating different views of how allometry should be analysed, but would be grateful if this point could remain as it is.

L404: First comma should be a period. **Fixed**
L405-406: Fix “be should” typo. **Fixed**
L406-407: This sentence doesn’t make much sense. **Sorry, that is correct! Now fully explained.**
L427-428: I would use “canid species” instead of “species of canid”, as it’s shorter and reads easier (at least to me as an ESL person). Fix separation between “canid” and colon. **Both fixed**
L477: Fix spacing issues in “Sparassodonta(e.g., 101).”**Fixed**

Reviewer #3 (Remarks on code availability):

I only checked a few snippets of the code, but they were clear, easy to follow, and the functions used were sound.

That is great to hear!

Reviewer #5 (Remarks to the Author):

Overall impressions:

I remain in favor of publishing the manuscript. The authors improved the clarity of their narrative. They addressed the concerns of five independent reviewers over multiple rounds of revision, including by the addition of new statistical analyses and a competing interest declaration. I particularly thank the authors for addressing my line-by-line comments, which I recognize were extensive. The responses were thoughtful and earnest, and where the authors departed from me, broadly speaking they had reasonable explanations (e.g., first-principles biomechanics) or there was a misunderstanding on my part. I find this version of the manuscript improved, and though I don't agree in every instance with the authors' interpretation of the results, the interpretations generally stand well on the analyses. I believe my comments below constitute suggestions and minor revisions to clarify points.

Thank you very much, we really appreciate the time it would have taken to go through the previous revisions and then review our responses to your suggestions!

Methods:
I appreciate that the authors both implemented the pooled analysis of allometry and experimented with using a Wheatsheaf(-like) Index (WI), both of which took time and energy. I accept that it doesn't seem necessary to include the WI results in the main text, but if the journal allows it, I'd encourage the authors to include the WI work as supporting information.

Ohh this is difficult to decide and so tempting. It would just require a small side mention, but we worry about the appropriateness of it - it would be presenting an abortive trial in using a metric not designed to be used in the way that we would have used it. We really appreciate the suggestion because it would provide recognition to the effort of coding it, and we would put it in if R5 feels strongly, but we are not sure it should go into the paper because it is difficult to justify its use in the methods.

For now, we retain the code as "apocryphal" in the github repository along with the code for the published results. That way, it can be seen by those wanting to replicate our work or interested in the matter.

Results + Discussion:
The thing that I am a little unclear about is the authors' interpretation of the results from the new pooled allometric analyses. The discussion of those results in the extended data seems opposite, or at least inconsistent, with the discussion of the results in the main text. It looks right that clade-specific mean shape is influential. But doesn't statistical significance of the interaction effect between clade and size nonetheless indicate that there are independent allometric slopes by clade (i.e., marsupials vs. caniforms vs. placentals)?

Yes, that is absolutely correct (and also why mean shape differences per size cannot be statistically pinned down, see discussion with R). We have now re-written the text to be clearer about this.

And the extended data makes it sound like that result makes the influence of clade-mean shape alone hard to interpret. Can't the thylacine's head morphology be explained, at least in part, by a marsupial-specific allometric trend or trends? If not, why not? I didn't expect a common allometric pattern to the whole sample (or to all partitions) to explain the thylacine's morphology, but I did wonder whether the thylacine is sortuva scaled-up quoll in one or more regions of the head, which the presence of clade-specific allometry might bear out (but if so, would presumably add to the idea of thylacine-as-mosaic).

VW tried to analyse this suggestion ("is the thylacine a scaled-up quoll?) by removing the very disparately-shaped large-skulled dasyurids – devils, thylacines, and *Borhyaena* - but in this sample the already weak allometric effect becomes non-significant. This is probably partly because the remaining sample size is quite small, but even so it shows that allometry is just not a strong effect.

Part of the problem is that I'm having trouble visualizing what the clade-specific allometry looks like at the extremes. I think I follow authors' logic that because the RegScore is small for thylacine, there is something or some things apomorphic about the thylacine beyond marsupial head-shape allometry that make them look like wolves. But the regression-fit plots in extended data Figure 4 sure look to me like the thylacinidae group's cloud (albeit just the two datapoints) parallels the best-fit lines. In other words, the fit still improves at the same rate as everybody else as size increases. Could the separation in regression fit simply be due to the thylacine group's apomorphically large heads

Those questions are helpful guides to an articulation of the allometric effects, thank you! With regards to whether the thylacine is a scaled-up quoll, we agree that the thylacine follows the overall samples' best-fit line, but whether this reflects a marsupial-specific pattern is not clear because it also plausibly aligns with the other two clades in most plots.

But R5 is also totally right that the plots do not reflect the allometry within each clade (we added an additional caveat on this in the Extended Results), which would require an 18-panel pgls plot, with no comparability between groups, which just replicates Extended Results Table 4. To avoid this and add clearer information on the actual divergence in variation described by the allometric extremes, we instead opted to follow R5's excellent advice above and below to show within-clade shape variation in deformation plots.

Since the interaction terms seem to be influential, at least to some degree, spline deformations could help readers like me visualize exactly what the marsupial allometric extremes are. Did authors visualize splines from the extremes of the pooled regressions? I'd recommend it, if not. They probably would help some readers visualize the strength of the various influences on shape. Given that authors make the case that the thylacine is a mosaic shaped by these influences, visualizing the mosaic could be worthwhile. I know that authors are limited on space, and they've already done a lot of work, so again I'm not going to demand these visualizations or anything, but I would ask them to talk a bit more about it in the extended results.

That is a great idea and also shows things more clearly than the plots, so we now have added a large figure characterising the smallest-to-largest allometric variation

for each clade, and this is such a great way of illustrating it. Thank you! But just to be clear, we are not arguing that the thylacine is a mosaic shaped by allometric influences. We are arguing that its huge size conveys a particular biomechanical function given its shape. Thus, while the results are great at showing the manifestation of divergent allometric slopes, they have little to say about the overall mosaic that is the thylacine - because allometry, as the section now hopefully clearly shows, does not come into it.

A final word on the allometry issue, to wrap together the different points made across the revisions: We hope that our now-extensive allometry section demonstrates our willingness to respond to all reviewer concerns, but we are still keen to retain most of this in the extended results because we do not see it impacting on the core interpretation of the manuscript.

Allometry is a pattern, not a process, and there is no clear hypothesis why we should expect a process that leads to allometric patterning tp drive the variation in the sample. The reason to investigate allometry deeply is still not clear to us. Might it be related to the idea of an marsupial allometric "constraint" that forces marsupials with larger skulls into a particular shape, so that thylacines kind of couldn't help but look like they do?

If this is part of the reasoning, there is next to no evidence for a strong allometric constraint in marsupial crania. For example, Weisbecker et al. 2023 assessed allometry of a much larger sample of marsupials including herbivores etc., and found that allometry accounted for a maximum of 12% of the variation. Similarly, in Weisbecker et al. 2021 very little allometry (~7% of variation) is found in the cranial endocast of marsupials, which arguably corresponds with parts of the overall, neurocranial, FNP, and MES partitions.

By contrast, as VWs lab showed in a recent review (Mitchell et al. 2024), allometric patterns (such as long faces in larger species) are explained comprehensively by biomechanical adaptation, and disappear when species have divergent feeding biomechanics/dietary hardnesses. For our metatherian sample, this makes strong allometric effects particularly unexpected (and is probably the reason a significant association disappears with PGLS) because the Tasmanian devil, *Borhyaena*, and the thylacine have highly specialised skulls. Allometric patterns in our metatherian sample are therefore at best a confused mix of those feeding adaptations, with added high leverage of a few divergently shaped shaped skulls such as the robust-skulled *Borhyaena* and the bone-cracking Tasmanian devil, both clearly biomechanically dissimilar to the thylacine.

Against this backdrop, we just have not seen the need to delve into allometry but hopefully have left no questions unanswered regarding the lack of influence of allometry on our sample 😊

Weisbecker, V., R. M. Beck, T. Guillerme, A. R. Harrington, L. Lange-Hodgson, M. S. Lee, K. Mardon, and M. J. Phillips. 2023. Multiple modes of inference reveal less phylogenetic signal in marsupial basicranial shape compared with the rest of the cranium. Phil. Trans. Roy. Soc. B 378:20220085.

Weisbecker, V., T. Rowe, S. Wroe, T. E. Macrini, K. L. Garland, K. J. Travouillon, K. Black, M. Archer, S. J. Hand, and J. C. Berlin. 2021. Global elongation and high shape

flexibility as an evolutionary hypothesis of accommodating mammalian brains into skulls. Evolution 75:625-640.

Mitchell, D. R., E. Sherratt, and V. Weisbecker. 2024. Facing the facts: adaptive trade-offs along body size ranges determine mammalian craniofacial scaling. Biol. Rev. 99:496-524.

I do want to offer my apologies that, for me, this time through is just my second view of the manuscript, because I know there have been rounds prior to my entering the picture as a reviewer. That said, thanks to the authors for hearing my concerns.

Thank you for spending so much time on this, it has been a journey but a very productive one for us!

Formatting and flow notes:
The narrative does make more sense, and I do better understand what the authors are trying to say. I am still concerned that some readers are going to take home the "extinct predatory behavior" thing, rather than "the thylacine was a mosaic and this combination of traits doesn't clearly exist in the modern mammalian biota", but the authors basically wrote the latter into this draft, so the only thing I would recommend on this point at this stage is to review the language again and make sure it's how they want the text to sound.

That is such a fair comment, thank you. We have made substantial changes to the Abstract to better align it with the manuscript and hopefully avoid this issue. We also went through the end of the manuscript to avoid this impression.

I will say that there are some writing-style tendencies by the authors, e.g., in my first line-by-line comment on this go-round, that make sentences really confusing for me, personally, as a reader. In most of these instances I think there would both be a way to reword the sentence to be clearer and save words. I want to be sensitive to authors' voice in this manuscript, because my intention is not to take away their voice(s), but sometimes the text just sounds like it contradicts itself, or the dependent clause at the beginning of sentence is so long that I get lost on how it relates to the independent clause once I reach it after the comma. My recommendation in any review when I encounter this kind of issue is just to make sentences shorter and simpler. I'll point out a few places, and I recommend the authors go through the manuscript with a fine-toothed comb to find others.

We have fixed the below points as recommended, and you will see in a smattering of tracked-changes that this helped us find several other instances where sentences could be simplified. Thank you for helping us with this!

Line-by-line comments:
58: "has provided an ideal potential example"
Confusing to read "has provided" but then also "potential". Either is or it isn't an example of something. If "potential example", then it's not an example yet and therefore hasn't been provided. Recommend dropping "Owing to this" and rewording to be more active, like "Comparisons of thylacine and canid cranial morphology could shed light on the genetic and developmental mechanisms of adaptive evolution in mammals." Saves 4-5 words over what's there but still communicates uncertainty. Not everybody likes the "shed light on" phrasing, and that's fine, but I encourage authors to play around with the wording.

This paragraph was subjected to some radical shortening because as R5 pointed out, the sentences were verbose and repeated parts of the following paragraph. The sentence is now gone.

138: I think there's a missing word after "genomic". **Correct - fixed to read "genomic basis"**

152: "significant allometry exists"
Since authors have the results tabled elsewhere for most if not all analyses, I encourage dropping the word "significant" in most cases. The term "significant" carries a lot of baggage (see, e.g., Martínez-Abraín, 2008; Dushoff et al., 2019) and can distract. Either there is clear evidence of an allometric relationship (with a p-value), or there isn't. Additional nuance can then be discussed after. I'm not gonna push the point any farther if the authors feel strongly, but replacing the word "significant" with either explicit reference to a p-value or to a table with statistical results generally is gonna save characters and potentially words.

VW has loved the concept of "clarity" proposed by Dushoff et al. and used it before, but her reviewer comments have been consistently negative (it is hard to change views on statistics!). So our experience is that replacing "clear" with "significant" is confusing. However, we place a heavy emphasis on referring to the amount of variation explained, rather than just significant vs. non-significant, which provides useful nuance (because a significant effect can explain very little or most of the variation). We hope this is acceptable as an indication as to how clear an effect is.

155-160: about the new analyses
I don't entirely understand the point here. The first sentence in these lines could use some simplification. Neither the intro clause nor the parentheses are necessary. It could just read something like, "Shape-size allometry differs among feliforms, caniforms, and marsupials for most skull regions in both partitioning schemes, except Neurocranium and MES (table citation)." That saves 9 words over the first half of the sentence. But I don't really follow the point of the second half of the sentence.

The allometry section has been re-written after the substantial additions of two tables (and a table extension) Visualising splines was an excellent idea and is now done, see above.

Doesn't the statistical significance of the interaction term mean that there are clade-specific allometries that do in fact explain the distribution of species in morphospace, at least in part?

Yes, but the scaling slope differences do not explain much of the variation and are definitely not a driving force in the distribution of species in Figs. 1 and 2. We now make this clearer in the re-written allometry paragraph.

What does the influence of the interaction term for marsupials look like at the extremes of that term for each partition? Does it resemble the thylacine at all, or is it wholly different? These details aren't in the extended results, but they are relevant if the case is that the thylacine is a mosaic.

See comment above (is the thylacine a scaled-up quoll?) - the extremes do not resemble the thylacine much even when the whole sample (including the thylacine, whose large size gives it strong leverage over the regression) is considered.

I also question whether "overwhelmed" is the right term later in these lines. It's currently hard to visualize what influence the interaction term has. Maybe the mean shape is an exaggeration of the clade-specific allometry, or maybe the influence on shape is totally different. I must apologize that I have not been able to test this question myself by running all of the code, but at a bare minimum I do recommend that the authors talk in the extended results about what the influence of the clade-specific allometry looks like. If they don't want to visualize splines, I won't push that specific issue, but discussing the influence, given that it does reach a threshold of statistical significance, would be worthwhile.

We have added the splines, it was a useful addition! We also changed the phrase "overwhelmed", which was indeed a poor choice of word.

168: "Their cranial size"
I think "their" means "thylacine" but the animals immediately preceding "their" are "jackals and foxes" and the grammatical subject of the preceding clause was "most placental carnivores". Please find other instances like this one and clarify the subject of the sentence.

We have addressed this hopefully throughout, you will see the odd tracked change where we improved cohesion between sentences.

183-185: subject-adjective confusion
Subject of this sentence is "length", so parallel construction of phrase after comma reads like length is "narrow". I expect authors mean that "rostrum" is narrow, but the sentence needs to be rephrased somehow for "rostrum" to be the subject, else it sounds awkward. My recommendation is to just break up the sentence into two: one about length and one about breadth. On the result itself, is the narrowness relative or absolute? I expect relative, since length was relative, but it's not clear. As in previous round of review, please look over use of words like "particularly". Saying that the rostrum is narrower than almost all other species already means that it's "particularly" narrow (and is more precise), so my recommendation is just drop the adverb to save the space.

This sentence has been re-written according to the recommendation: "Consistent with the geometric morphometric analyses, the thylacine has a significantly longer rostrum length (measured as palate length) compared to all but the smallest species (Fig. 4b). It also is narrower at the canines relative to the geometric mean than nearly all comparative species (Fig. 4c). "

225: "thickened rostrum"
"Thickened" sounds like the bone is thicker or more robust somehow. Recommend "broader" or "wider" if authors talking about width.

We went for a more precise phrasing: "…greater lateral and dorsal extent of the rostrum relative to the post-canine "pinch" "

227: "are narrow"
So I think I get it, but in 225 the rostra were "thickened". If authors mean the rosette in 225, recommend rewording to make that clearer.

We improved this to be clearer: "the rostra of the thylacine and A. mongoliensis are medio-laterally narrow behind the terminal rosette (Fig. 5a),…"

also 227: "rarely represented"
Redundant, just say "rare".
Fixed!

229: "sacrificing performance"
"Performance" a little vague. Recommend "stability" or "resistance" if that's what authors mean.

Thank you, we went with "resistance"

232: "unusually powerful"
Unusual relative to what? Recommend removing the parens preceding these words and rephrasing sentence more like "...consistent with a relatively fast, powerful, 'snapping' bite used to acquire smaller, agile prey."

Done, thank you!

242-257: IOF discussion
This paragraph still feels overly speculative to me, but I do have a new recommendation to address that concern. I absolutely appreciate the authors including the references I recommended in addition to their thoughtful responses to my concerns. I am, though, disappointed that authors did not mention vasculature. Perhaps it seems like a ticky-tacky concern, but there are structures other than a nerve passing through IOF; nerve, artery, and maybe vein (and probably lymph). Muchlinski (2008) studied the soft tissue directly, and that study might be a better citation in this location than the 2010 pub. That being said, the data in Muchlinski (2008) sure look like carnivorans have relatively small infraorbital nerves for the foramen (the study did not explicitly run ANCOVA for any groups other than primates). If thylacine converged on carnivorans morphogenetically **and** vibrissae were only modest, perhaps infraorbital artery was more pronounced in thylacine. I recommend that the authors reframe this paragraph in a more explicitly inferential framework, e.g., the inverted pyramid and extant phylogenetic bracket (Witmer, 1995). If authors think that this approach will not be helpful, I won't press the issue, but I hope that they will consider it seriously.

The speculative feel probably comes from needing to document a conspicuous trait that is unusual enough to warrant a note, despite not being easily explainable.

We now cite Muchlinski 2008 and have extended our justification for equating the IOF with IO nerve (ION) diameter, but are reluctant to expand on the scenario of a dominant artery. IOFs are generally assumed to be mostly filled by neuronal tissue (we now add a new reference, Sánchez-Villagra and Asher, in support). In addition, Muchlinski (2008) indeed found that the contribution of the infraorbital artery is negligible; the variation in ION vs. IOF correlation seems to come more from the connective tissue surrounding the neuronal bundles, by that paper's Fig. 1.

It is also not correct to assume that a large IOF is predominantly associated with extensive or highly sensitive vibrissae, and that the lack of these means that the IOF might have been filled with a blood vessel (hopefully this reflects R5's suggestion correctly?). There are diverse areas of the maxillary region innervated by the ION and

the whisker-vibrissae relationship is definitely less clear than the relationship between IOF size and ION size (Muchlinski 2010).

We now substantially changed the text in this section to clearly highlight this lack of strong association and difficulty in ascribing any function for the IOF size in extinct animals.

Importantly, we reduced the mention of over-erupting canines substantially because it became too speculative and was not initially intended to be our focus. In their first review, R5 suggested that we include a comment on canine overeruption in felids (Smuts et al. 1978; Riviere & Wheeler 2005). We initially included these references in an expanded section on canine over-eruption. However, when we re-visited this section and tried to simplify, we realised that felid over-eruption noted in these papers is not comparable to what we see in dasyuromorphians. It is more the suggested use of whiskers/lips in canine placement, and less canine over-eruption, that needs highlighting when discussing felids.

Smuts et al. detail the dental eruption and wear stages etc. in lions; showing "complete and fully erupted dentition" by ~4 years of age, with no evidence of further eruption. Rather, there is resorption of alveolar bone and ablation of enamel, as can be seen and discussed re: Plate 5a therein. This is not overeruption, but dramatic use wear of the dentition.

Riviere & Wheeler discuss the visually obvious 'extended root' of *Smilodon* canines, which gives them an appearance of 'tooth slippage' (common in dry skulls) or overeruption. They detail arguments that this is not an extended or exposed root, but an apical extension of cementum which morphologically and histologically supports a hypothesis of a cover of gingival tissue.

As such, neither of the articles support dramatic dental overeruption in felids (nor indeed other placental carnivores; see discussion in Engleman & Croft, 2014; Jones 2023).

Jones (2023) only samples two feliform representatives (*Hyaena hyaena* and *Herpestes ichneumon*), but neither show anything close to the degree of canine overeruption as the marsupials sampled - particularly the larger-bodied Tasmanian devil (*Sarcophilus harrisii*) or the thylacine (See fig. below).

[Figure Redacted]

Engelman RK, and Croft DA. 2014. A new species of small-bodied sparassodont (Mammalia, Metatheria) from the middle Miocene locality of Quebrada Honda, Bolivia. Journal of Vertebrate Paleontology 34:672-688. 10.1080/02724634.2013.827118

Jones ME. 2023. Over-eruption in marsupial carnivore teeth: compensation for a constraint. Proceedings of the Royal Society B: Biological Sciences 290:20230644. 10.1098/rspb.2023.0644

Figure 6: really appreciate the authors making the changes here. They help me, and the number of labeled taxa look about right. It's not too busy.

Thank you!

280: "does not coincide with the dorso-ventral impact resistance"
I don't understand. Are authors saying that C. simensis has low impact resistance? If so, that probably needs a citation. Or are you just saying that the skull isn't tall? Again, if there are two points, my recommendation is to break them up into two simpler sentences.

Sorry, that really was a clanger. It now reads "However, this species differs from the thylacine in lacking a dorsoventrally tall snout and in a smaller relative cranial size (Fig. 2, 3), suggesting substantial differences in absolute bite force and biomechanical properties of the cranium"

305-308: constraint, contingency discussion
I appreciate that the authors discussed Felice et al. (2018).

It was a good addition, thank you.

310-311: "living animals that likely show major differences"
From whom? Unclear

Toned down to "While the molecular underpinnings of the thylacine's cranial morphology may not be easily traced,"

323: "whose"
Imprecise. I know space is limited but "the maxillary branch of which" is only two more words and is more precise. Save words elsewhere.

In keeping with R5s overall concerns that the text could be further streamlined, we re-vamped this section to be much shorter and more concise.

326-330: from "potentially" to "craniofacial evolution"
This section needs some work, it's a bit tautological. Also, two uses of "unusual" in the same phrase, which sounds awkward. In next sentence, "It is thus possible...", the invocation of cranial neural crest is overly vague. What does "facilitated from evolution within cranial neural crest cells" mean? The population of cells? Traits of the individual cells? Temporal or spatial shifts? It's like...the FNP is made of neural crest, so of course the CNCC evolved. Had to if the FNP looks different. The question is how (and how much), not whether. How do we get from a large infraorbital nerve to SHH to dental stem cells to the CNCC, and how might these things have evolved in the thylacine? I don't think there has to be a ton more text here, but if the hypothesis is that a large infraorbital nerve would lead to the evolution of a terminal rosette — or whatever is meant here — the logic needs to be a bit clearer.

As above, we unpacked this whole paragraph to now be clear what we mean by mentioning the potential developmental implications of an enlarged maxillary branch of the trigeminal nerve. We hope this is less awkward, and just note that we are not confident to have a strongly articulated hypothesis (such as large IOF leads to terminal rosette, sorry if it came across as an actual suggestion!). So we just highlight some relevant future areas where our results can lead the way to fruitful future investigation. The SHH sentence is now deleted because it is an unnecessary example relating to the prior statement of signalling between nerves and developing craniofacial morphology.

Lit Cited:

Camp, A.L., Konow, N. and Sanford, C.P.J. (2009), Functional morphology and biomechanics of the tongue-bite apparatus in salmonid and osteoglossomorph fishes. Journal of Anatomy, 214: 717-728. https://doi.org/10.1111/j.1469-7580.2009.01056.x

Dushoff J, Kain MP, Bolker BM. I can see clearly now: Reinterpreting statistical significance. Methods Ecol Evol. 2019; 10: 756–759. https://doi.org/10.1111/2041-210X.13159

Evans, A.J., Naylor, E.R., Lujan, N.K., Kawano, S.M. & Hernandez, L. (2024) Deploy the

proboscis!: Functional morphology and kinematics of a novel form of extreme jaw protrusion in the hingemouth, Phractolaemus ansorgii (Gonorynchiformes). Journal of Anatomy, 244, 929–942. Available from: https://doi.org/10.1111/joa.14020

Alejandro Martínez-Abraín. 2008. Statistical significance and biological relevance: A call for a more cautious interpretation of results in ecology. Acta Oecologica. Volume 34, Issue 1, July–August 2008, Pages 9-11

Muchlinski, M.N. (2008), The Relationship Between the Infraorbital Foramen, Infraorbital Nerve, and Maxillary Mechanoreception: Implications for Interpreting the Paleoecology of Fossil Mammals Based on Infraorbital Foramen Size. Anat Rec, 291: 1221-1226. https://doi.org/10.1002/ar.20742

Witmer LM. 1995. The Extant Phylogenetic Bracket and the Importance of Reconstructing Soft Tissues in Fossils. in Thomason, J.J. (ed). Functional Morphology in Vertebrate Paleontology. New York. Cambridge University Press. pp: 19–33.

Reviewer #5 (Remarks on code availability):

The code still seems reasonably well-annotated. I appreciate the humor written into some of the annotations

Thank you! Glad it seems clear.

In this manuscript, the authors present a series of arguments suggesting that the thylacine was the last surviving member of a predator group employing fast, high-impact snapping to capture their prey. Surprisingly, they posit that this prey might have been fish instead of small terrestrial vertebrates (as suggested by current hypotheses on thylacine dietary preferences and hunting habits). I'm afraid I don't share the authors' confidence on the implications of their results, neither think I that enough evidence is provided to support that claim.

Overall, I think this is a well-written manuscript presenting novel and interesting results on thylacine (and canid) cranial shape variation, while at the same time linking them to recent studies on cranial development and genomics on those species. My major concern is that the authors focus on their narratives (wolf vs thylacine, extinct predator group) so deeply that they seem to disregard any alternative potential explanations to their results. A few examples would be:

- in some cases the results are presented in a way that could bias the reader (e.g. see comments on L73-76 below).

- most of the Results & Discussion section is focused on the differences and similarities between the thylacine and the wolf (representing large-prey predators), but the same comparison is not made with the small-prey specialists. In fact, any comparison with other large prey specialists, like *Cuon* or *Lycaon* is also missing. This could be particularly important when discussing Figure 3A (see comment for L127 below).

- despite being most similar when combining cranial shape and size, *Chrysocyon* is disregarded as a potential ecological equivalent based on its diet (L132-138) but... isn't the whole purpose of this study to try to understand the thylacine diet? Couldn't it also be an omnivorous species eating both small mammals and fruits? A similar disregard of *Canis simensis* occurs later in this section (L199-203).

Thus, I would strongly recommend the authors to tone down this narrative by presenting it as a potential explanation, along with several others, of their results.

For more detailed comments, please refer to the line-by-line section below.

Apologies in advance if some of my comments seem too harsh, that was not my intention. I might have issues with how the results are discussed in the manuscript, but I do not wish to undervalue in any way the work or ethics of the authors.

Eloy Gálvez López (anonymity waived)

L2: Author order is different in the manuscript than in the system (emails, reviewer dashboard, etc.). I assume the one in the manuscript is the correct one?

L34: Remove repeated "an important".

L38: Remove repeated period.

L39: This might read as "they're similar in cranial shape", as convergent is often used very loosely in the literature. However, since this has been statistically tested in Rovinsky et al (2021), I would recommend rewriting this as "[...] and have been shown to present convergent cranial shapes with canids" (or something similar). This change will also strengthen other instances when convergence in skull shape is brought up (e.g. L72).

L268-287: I would add "(see below)" before the colon to avoid the reader wondering about the nature and contents for Rovinsky et al (2021)'s sample for two paragraphs. Alternatively, the authors could rewrite this paragraph to incorporate lines 284-287: "For this study, we used Rovinsky et al's cranial dataset of 381

three-dimensional (3D) landmarks (46 fixed [...]) of 225 specimens from 60 mammalian taxa (including 17 thylacine crania; Suppl. Data 1), to which we added three new specimens using the Viewbox 4 (...) protocol established in that study: the Argentinian [...]"

Regardless of which approach is selected, please reference Figure 2 on the parentheses describing the landmark configurations. Since there is no proper figure for it in this study (yes, I know it's in Rovinsky et al (2021)), linking to a figure where the configurations are shown would be helpful for those of us who always read the Methods section before the Results.

L288: Why are the zygomatic arches not included in either partition? They are a good indicator of masticatory muscle size, and hence have significant biomechanical implications.

L294-303: This paragraph is quite convoluted. It took me a few reads to understand that it meant "We visualized shape variation in each partition using PCA (Fig. 2). Similarities in cranial shape were assessed using Procrustes distances, and the closest species to the thylacine are shown in Figure 3." I would recommend expanding that summary as needed, but please keep it simple and direct. Also please mind the references to the figures. As mentioned above, it helps readers who don't read the manuscript following the published section order.

L306: I would remove the "felids vs. canids" example (and just leave the other one), as the former are only mentioned here and the latter are only compared to thylacinids.

L307-311: I tried accessing the R script with the links provided but I kept getting errors, hence this comment. Did you use *procD.pgls* to calculate the size effect on cranial shape or *procD.lm* (or *procD.allometry*)? The Extended Results mention Procrustes Generalized Least Squares (which usually refers to *procD.lm*) for shape instead of Phylogenetic GLS (*procD.pgls*), so I wonder whether phylogeny was included in the regression or not. If indeed it was, please edit the Extended Results.

L325: Please add "Figure 4" at the end of the parenthesis to guide readers to a visualization of (some of) the measurements taken.

L327-328: Please give species names for all species. Why grey wolf but just "South American canids" or "fox"?

L. adusta – side-striped jackal; *L. gymnocercus* – Pampas fox (or Azaras' zorro if you want to distinguish South American "foxes" from vulpine foxes); *V. vulpes* – red fox

L335: Remove repeated "then".

L340 & Figure 5: Two related comments. First, please link the description to Figure 5 to direct readers to a visual aid ("The rostrum of the thylacine (Figure 5) is [...]"). Second, either replace the drawings in figure 5 by images of the actual cranium (or mesh, ideally with the relevant landmarks) or add those as separate panels for the figure (ideally for all the species highlighted). While the simplified drawings might be very clear to the authors, I'm still not sure that I understand them.

L343-344: You use "prediction range analysis" here but "prediction interval comparisons" later on L356. For consistency, please change this sentence to "As with cranial size, we calculated the 95% prediction intervals of the regression to ask whether [...]".

L361: I think there's a double spacing between *Nimbacinus* and using.

L361-366: This paragraph is useful when you read the Methods section before Results and Discussion, but probably not in any other situation. I would suggest moving this to the Introduction (maybe between L57-58 when discussing small- vs large-prey specialists).

L73: Here I'd go deeper than "aligning with previous studies". The cranium PCA morphospace is almost identical to that of Rovinsky et al (2021) even after adding the extreme *Andrewsarchus*, and leaning into that would prevent readers from wondering whether its inclusion is affecting the direction of PC1 in a similar way than it did on the shape vs CS regressions.

L73-76: For me, this is the first red flag regarding the forced narrative I mentioned above. By saying that PC2 "only" explains 21% of shape variation vs PC1 explaining "over half" of said variation, the authors are planting the notion on the readers that PC2 should be disregarded. First, for a study using 381 3D landmarks, we would expect over 1100 PCs to be generated, which means that by chance alone, each one should explain under 0.1% of shape variation. Thus, 21% of shape variation is not a small amount. Second, according to Figure 1B, PC1 explains 42.82% of shape variation, which is not over half.

A more objective description would be: "However, the thylacine lies just outside of the canid region of the PC1-PC2 morphospace, which represents over 60% of cranial shape variation. While the low scores of the thylacine on PC2 fall close to the canid range (excluding the widely divergent *Speothos**), the overall distinctness of the thylacine cranium from both metatherians *and* placentals is emphasized on PC1. Here, metatherians [...]"

Furthermore, this more objective description provides stronger support to using PC2 to illustrate the divergence between *Thylacinus* and *Nimbacinus* (L79-80), as its importance has not been minimized previously.

* I assumed the canid on the top left region of Fig. 1B is *Speothos* due to its unusual morphology, but please correct that if I'm wrong.

L79, L82: To avoid confusion, change to "(Fig. 2A, circles)".

L82: Please remove "a" before "both".

L87: Potential double space after the first period.

L95-97 (Figure 1): Please add the same legend as in Fig 3, and provide species names on panel 1B for the canid "outliers" (top left and lowest PC2 score).

Also, why is the polarity of PC1 inverted in the corresponding panels of Suppl. Fig. 1?

L103: Could these distance values be provided with the supplementary material? Since not all canids are represented on Figure 2, is hard to imagine that the canid species with negative PC1 values are closer to the thylacine than the wolf is.

L104-106: These lines could be a great segue to discuss differences and similarities with other large prey-specialists and with small-prey specialists (e.g. the red fox is the closest non-thylacinid in rostrum shape). This would provide a more nuanced interpretation of the results.

L111-113: While the rest of this paragraph does a good job in linking recent genetic studies with the shape variation described here, I think this sentence pushes the argument too far. This study neither includes any evolutionary modelling, nor presents any morphospace that, once turned into a phylomorphospace, would suggest cranial shape divergence between wolves and other canids. Thus, it needs to be removed.

I understand that the authors might want to make a big statement out of their findings, but this is the kind of sentence that is later referenced out of context and ends up becoming accepted without proper initial support.

L124: The suggestion for L104-106 would also add support to this sentence, as there has been barely no mention of other canids beyond the wolf.

L126: I think Suppl. Fig. 2 should be replaced by Fig. 3B (thus reducing Fig. 3 to the 3A panel) and a similar plot of the living marsupials presented in the current Suppl. Fig. 2. As it stands, the figure draws more attention on how relatively large the cranium of *D. marsupialis* is than to the thylacine, so it leaves the reader wondering why is that not discussed.

L126: Unless I have missed any regional spelling, it's "outsized" not "out-sized".

L127: Similarly, I would recommend writing "wolf equivalent" instead of "wolf-equivalent". In any case, I would remove "Consistent with its history of being considered a wolf equivalent," altogether, as it doesn't make sense with the rest of the paragraph. On the contrary though, if the two canids between the thylacine and the wolf in Fig. 3A, which also have particularly large crania relative to their size, are *Cuon* and *Lycaon*, that could be interpreted as canid large-prey specialists having wolf-sized crania regardless of body mass, which would then support the claim that the authors are trying to refute. (Based on the following paragraph I assume one of them is *Chrysocyon*, but what is the other?)

Another result of the log CS vs log body mass regression on Fig. 3A is that all canids have relatively large crania for their size, as they always fall on top of the regression line. Could then the convergence with canids explain why the thylacine lies over the marsupial regression line (Suppl. Fig. 2)?

L127-128: Either "the thylacines' cranial size puts them" or "the thylacine's cranial size puts it".

L132-138: As stated above, this is one of the clearest examples of pushing the extinct predator group narrative to the detriment of alternative hypotheses. Compounding on that, the authors list no references supporting the thylacine's hypercarnivory, with the listed references (26.27) corresponding instead to *Chrysocyon*'s diet. Thus, this paragraph needs to be rewritten. Either further evidence of the thylacine's diet which negates the intake of fruits needs to be referenced, or the authors should acknowledge that an alternative hypothesis to their results would be that the thylacine was a predator of small-sized vertebrates which potentially complemented its diet with fruits.

L150-152: If I understand it correctly, the argument here is that the geometric mean, as a proxy of cranial size, increases with condyle width, as a proxy for body mass, with a higher exponent in the thylacine. If that is the case, I would strongly recommend the authors to invert the axes of Fig. 4A. As the visual interpretation of the current panel suggests a lower exponent for the thylacine, which I assume the RMA slope actually is if the geometric mean was used for the x values. While RMA regression incorporates residual error for both variables, it is still best practice to match the regression formula with the desired interpretation of the result. In this case, for the higher scaling of cranial size to body mass, condyle width should be used as independent variable (x).

L153: I don't think saying that this regression mirrors the interspecific differences in cranial centroid size, as they're comparing rates of change to average values, but I do think these results could serve as a hypothesis of why the thylacine have relatively large heads for their size: they increase in size relative to body mass faster than in canids. This leaves the question however, on whether that is a marsupial trend related to their altricial development.

L160: I would suggest removing "related" from this sentence to avoid the potential misinterpretation that the thylacine was a canid (despite how clearly the contrary is otherwise stated in the manuscript).

L163-165: This is another example of the authors pushing their narrative over alternative explanations. For once, the process is not as "extreme" as the authors claim, as for some of the studied variables, while different from larger canids, the thylacine's regression lines seem to extend trends observed in the smaller species. For instance, their scaling of both palate length and width at the canines is not significantly different than any of the studied foxes or jackals. Thus, another interpretation of the same results could be

that, by having crania larger than similar-sized canids, they are able to maintain proportions similar to those of smaller canids, all small-prey specialists. This would support previous studies suggesting that the thylacine was a small-prey specialist (Attard et al. 2011; Rovinsky et al. 2021) probably using pounce-pursuit strategies (Jones & Stoddart 1998), so I am unsure on why this isn't included here.

Another topic is that, while the thylacine's gracilization has been shown to be different than that of some of the studied species, differences were also found within that pool of other species, yet this is disregarded. All this, coupled with the proliferation of studies on many-to-one relationships between bone shape and its function, makes the argument presented here a bit thin.

L173-184: This whole paragraph provides further support to the thylacine being a small canid equivalent, but yet the authors dance around that statement to maintain the extinct predator group narrative.

L188: As noted above (L340 & Figure 5), please provide a better visual aid for the reader to understand this "terminal rosette".

L190-192: While this might be true for crocodylians, the thylacine's rosette doesn't seem developed enough to grant this hammer-like properties. Maybe a better figure would help this argument, but as it is, I don't see enough support for this. Figure 5 illustrates a relative pinching at the canines, but that doesn't provide any insight on the mass distribution of the snout. A potential solution to this would be to calculate the voxel proportions of the rosette relative to the rest of the snout in the cranial meshes, but this might be a very rough approximation if the meshes were acquired using a surface scanner instead of CT.

L194-195: Relatively tall compared to what? Can this be observed in the shape changes along PC1 or PC2? Based on Fig. 1A, the thylacine's snout doesn't look taller than the wolf's.

L199-203: Would this make *C. simensis* also part of this potentially extinct predator group?

L211-219: This paragraph is the final leap on the extinct predatory group narrative: What if the small, fast-moving prey mentioned earlier weren't small mammals but fish? Then the thylacine might be a piscivorous predator because it has a "terminal rosette" like all these other clades.

I don't think that this manuscript provides enough evidence to support this claim.

L220-234: While interesting, this whole section on IOF doesn't feel connected to the rest of the manuscript, particularly when the lack of developed whiskers in the thylacine would be an argument against their piscivorous diet narrative. Why delve into it here then? I believe this might be an interesting short study on its own. Maybe add some other dasyuromorphs and map IOF surface during their evolution? Couple it with evolutionary modelling of dental over-eruption to test their hypothesis on soft tissue maintenance?

L237-243: See most of my comments above, but this needs to shift from present tense to a hypothetical conditional tense.

This is a fully rewritten version of the manuscript I previously revised. I want to make clear that I sincerely appreciate the efforts taken by the authors to rework and make clearer their ideas and thoughts. Before moving on, I would like to make some clarification about the answers provided by the authors.

First;

ok, this is not a paper on convergence and this a shame. Possibly I was expecting/hoping to read a paper on convergence, but this was not the case. However, I still recommend to replace the word "convergence" with "similarities" throughout the manuscript. However, I deeply appreciated the effort taken by the authors to rework the manuscript. As a side note, the metrics adopted in Rovinsky et al. (2021) are outdated, so, just for fun, I would re-run some of those analyses, take also a look at Grunstra et al. (2024) have a better understanding of what is possible to do with Procrustes distances.

Second;

"With ~160 million years evolutionary divergence and distinct differences between placentals and metatherians, of which the thylacine is the only exception in aspects of its shape, there is no need for testing if these differences arose independently! In addition, this has been done before e.g. in Rovinsky et al. 2021. We are now clearer that this is a reasonable assumption in the introduction (2nd paragraph)."

I probably expressed myself badly here, sorry. However, I was meaning that in dealing with convergence you test whether two species significantly depart from their ancestors' shape toward a common area of the morphospace. That is, without a phylogenetic background it is impossible to trace such ancestor-descendant pathway, independently of how much evolutionary time separates the two focal taxa. This is far from a trivial as more often than not, putatively convergent taxa fail such test. Hope this is clearer.

"We did not, as R1 suggests, use Procrustes distance comparisons as a naïve and phylogenetically uninformed way of testing convergence. And, as outlined above, there is no relevance to include ancestor-descendant relationships because it is clear that any similarities between the thylacine and canids are not based on a common ancestral condition. Instead, our study goes back to the very basic but also deeply fundamental question of asking which animal the thylacine resembles most in different sections of its cranium, based on previous results (Rovinsky et al. 2021) that similarities between canids and thylacines are not consistent with any carnivoran ecomorph."

Sorry, but this is exactly what I said in my comment. You simply measured the distances between some species and the thylacine (here taken as reference), without accounting for phylogeny. There's nothing bad about it, but distances are silent other aspects, namely phylogenetic history. Furthermore, in my comment I haven't by any means claimed that PCA is better than using Procrustes distances, as it really depends on what your scientific design is about.

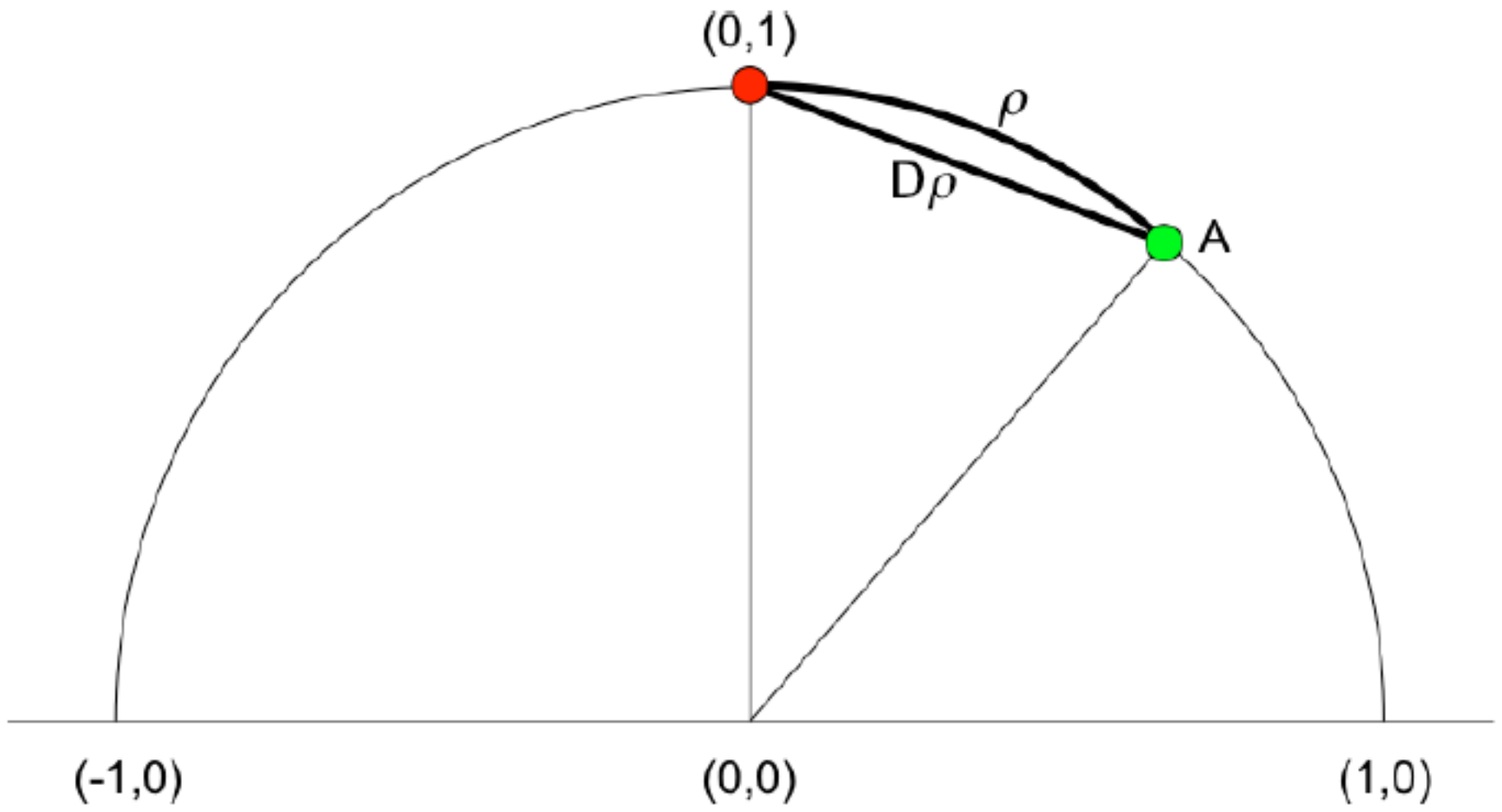

In this picture is shown the full Procrustes distance (rho) and the partial Procrustes distances (Drho) between a reference (the red point) and an hypothetical configuration A. Now, what is generally measured, as you did, is the distance Drho. Now, you can imagine another configuration B exactly at the same Drho, but on the -1 side. That is, two configurations can have the same partial Procrustes distance from the reference, despite having different shapes, that's why we need to visually explore the shape changes. If the authors feel I am not adequate or competent in GMM, please have a read at the PalaeoMath Series from Norman MacLeod (https://www.palass.org/publications/newsletter/palaeomath-101) where these topics are treated in a much more mannered way.

Again, my comment was intended to make the author fully aware of the limits in using such distances.

Now, I hope everything is clearer and I can move on reviewing this new version, which is not about convergence. However, this version reads much better than the previous one and is way clearer about the study's aims.

However, I still have some concerns the main one related to the diminutive sample size available. I do not think that 59 species are capable of capturing the variability of placentals carnivores (280

species) and/or marsupials. As a matter of fact, you lack ursids, many felids, many viverrids and mustelids are very poorly represented in the sample. Nonetheless, you lack almost all of the largest carnivores (lions, tigers, brown and white bears), which could have added a lot more considering that an allometric analysis has been performed. Also, it would have been a good idea to add some more fossils, in particular sparassodonts (when available of course).

Another issue is represented by the allometric analysis. Below, I provided more details, but in summary running regression PC by PC is a very discouraged practice in GMM (please see any contribution from David Polly, Dean Adams or Philip Mitteroecker).

Another problem is the use of size as a proxy of bite force. This came out without any testing, and I am not very happy with such a choice. Cranial size is not the only determinant of bite force as muscle orientation and distance (to make an example) make a lot of difference. So please remove it or analyse it differently, like taking bite force data from other published venues where a proper biomechanical analysis was performed.

Minor points

Abstract

I recommend to minimise the use of the word "convergence" here and throughout the manuscript, as you did not measured it. Replace with "similarities" or something like that.

Introduction

Lines 71-73: This would have been interesting to test.

Lines 81-82: I have some issue with using size as a proxy of bite force.

Results and Discussion

Line 93. I have issues with such a diminutive sample size. Carnivora include around 280 living species and many more extinct (see Faurby et al. 2024). I do not think that this sample can be considered representative of the carnivoran variability. Also, as mentioned above, it would have

been good to include some more sparassodonts as this could have shifted our view of thylacines to be more similar to canids than to other taxa.

Line 134: How can you say that dingoes and wolves are unique among canids?

Line 136-138: This is a fundamental problem with this manuscript and what I tried to make evident in my previous review. Without any proper phylogenetic comparative analysis, you can’t say anything about any putative departure of any species from their ancestors. This looks very speculative to me.

Lines 157-161: I got what you mean, but please rewrite it. Without having a look at the supp it’s hard to get your message. Anyway, it is highly discouraged to analyse single PCs separately, as you arbitrarily selected 10 PCs and performed allometric analyses PC by PC finding little evidence to say that allometry is meaningless in your data.

The correct way is to analyse the full set of Procrustes coordinates vs size (as you did at first). Here, since you get a significant result, it would have been nice to compare the allometric slopes of shape data. You could have run separate pooled regressions (e.g. separating Canidae, Felidae, etc.) and test whether these resulting slopes were convergent, divergent or parallel. Also, a deeper investigation of allometric slopes would have revealed whether the Thylacine can be considered “hypermorphic” compared to other marsupials.

In the end, please remove the PC by PC analysis as it go against any development in modern morphometrics and rephrase the paragraph.

Figure 4. I guess you miss-labeled the axes in figure 4a.

Overall, this revised version of the manuscript has vastly improved upon the original, both in terms of structure and background information, and by addressing the comments of three different reviewers, so I wanted to congratulate the authors on that.

I particularly like the rewritten Introduction (but see comment for L292-298). Both the restructuring of its previous sections and the added bits of information (particularly regarding the thylacine's diet) help paint a clearer picture of what we currentlly know about the thylacine's ecology and relevant morphology and what this manuscript aims to address.

Regarding the changes to the Methods section, I am unable to discern whether they're easier to understand because of the rewrites, or because I already know what those analyses are from the previous versions. Hopefully other reviewers will add notes on that topic if they still think it's unclear. No notes from me though.

As for Results & Discussion, my thoughts on the authors' justification for not describing other species and just focusing on the wolf-thylacine comparison can be found at the end of this document ("Keeping the dialogue open" section), but I wanted to note here why I still slightly disagree with the authors in that regard. Specifically, there is a disagreement between the distance values plotted on Figure 2 and the distance values presented in Suppl. Table 1. If the latter are correct, the pattern described for the grey wolf and dingo (closest to thylacine in posterior cranium but far away in rostral region) applies to all *Canis* species except *C. simensis*, and to *Cuon* and *Lycaon* as well, not just the grey wolf and dingo. This shifts the results away from the uniqueness of the wolf and dingo relative to other canids and makes it a trend within the Subtribe Canina (now *Lupulella* and *C. simensis* are the exceptions to cranial shape/development within Canina). With this in mind, the authors could expand their hypothesis that wolf/dingo and thylacine both diverged from their ancestors to evolve a similar cranial shape, to this divergence occurring early on in the evolution of Canina, right after the *Canis/Cuon/Lycaon* branch diverged from *Lupulella*. *C. simensis* might represent a shift back to the ancestral canid condition, an early stage of divergence towards a new set of cranial features (see comment on L256-263), or their Ethiopian wolf specimens might be incorrectly assigned to that species*. If the authors agree with this interpretation, the overall discussion of their results could be easily shifted from the wolf comparison to a wolf-like canid comparison (i.e. Subtribe Canina).

* In my experience, misclassified specimens are common in museum collections, particularly when they were collected in the early 1900s or when they belong to species in distant continents.

Beyond that, I wanted to congratulate the authors again on a fantastic job improving this section. It reads much better, all my doubts regarding a specific narrative being pushed against other potential explanations have been quenched with clarifications and new analyses and improved figures, and there is no room for misinterpreting the authors as stating that the thylacine was piscivorous (sorry about that btw!). Congratulations!

Eloy Gálvez-López (anonymity waived)

L31: Change "carnivorous" by "hypercarnivorous". I think this will solidify any potential doubts regarding thylacine diet.

L36: Fix typo: "observatione.g."

L41: Either "similarities of the thylacine with canids", or "similarities between the thylacine and canids"

L72: Fix typo: "sparassodontse.g."

L386: That comma feels weird. I would remove it, but this is just a suggestion, not a request.

L397: You might want to rewrite this line as: “ We $\log_{10}$ transformed centroid sizes and regressed them against $\log_{10}$-transformed species’ average body masses […]”. I believe it’s shorter and easier to read.

L451-456: This paragraph no longer has a purpose, as “grey” has been added to all other relevant mentions of wolf (except L293), clarifying that the authors are referring to this species only. I would strongly recommend the authors to delete it.

L456: If the authors don’t remove those lines, the “prey30” typo needs to be fixed.

L124-L141 & Figure 2 / Suppl. Table 1: See comment above. This paragraph should be rewritten to acknowledge the discrepancy (and potential new evolutionary hypothesis), and the figure modified to present the correct values.

Extended Results: “Excluding *A. mongoliensis* from the analysis produced a nearly non-significant association (R^2 = 0.33, p= 0.000) between cranial shape and log(cranial centroid size)”. I am quite confused with this statement. Why is this nearly non-significant? If this is based on p=0.0000, which is common when outputs are limited to 4 decimal places and the value is lower than 0.00005, the results are actually the opposite (i.e., excluding *Andrewsarchus* increased the significance of the allometric relationship). With significance set at p=0.05, a nearly non-significant result would be p=0.0499 or higher.

L159-160: This no longer seems to be true, based on my earlier comment regarding the disagreement between distance values in Figure 2 and Suppl. Table 1. I would recommend extending this argument to Canina instead.

L172: I don’t understand what you mean here. It reads as if something is missing after “absolute”.

L219: I assume defining small prey as < 10 kg is a ballpark threshold, as there’s no associated reference. If that’s the case, I would recommend changing this to < 7.5 kg, representing 45% of the thylacine’s latest body weight estimations (ca.16.7 kg). Small and large prey are usually defined relative to predator size and Carbone et al. (1999) proposed that 45% threshold based on the energetics of hunting and prey processing.

Carbone C, Mace GM, Roberts SC, Macdonald DW (1999) Energetic constraints on the diet of terrestrial carnivores. Nature 402, 286–288.

Figure 5: I just wanted to state how gorgeous this figure is now. Zero doubts about what the terminal rosette is. Fantastic job!

L247: Fix “see aso” typo.

L256-263: If the authors adopt the wolf-like canid comparison focusing on Canina instead of just grey wolfs and dingoes, this could be a great opportunity to explore why *C. simensis* does not follow the trends observed in other Canina. Could the Ethiopian wolf be an early state of divergence towards acquiring a morphology similar to the thylacine/*Andrewsarchus*? They seem to be developing a terminal rosette, could the development of a taller snout and relatively large head follow?

L274-287: I still don’t think the IOF analysis and discussion connects with the rest of the study, but the authors make compelling arguments for keeping it in their rebuttal.

L292-298: This paragraph is the perfect example of how vastly the authors have improved the manuscript in this revision. The paragraph itself has barely changed but, while in the previous version I thought it exemplified all my issues with the manuscript and I requested that it be changed to a conditional tense, now I believe is the perfect summary for the preceding section. Again, great job.

L326: “thylacine-canid comparison” instead of “thylacine canid-comparison”

L335-336: Delete spaces before reference numbers

--- Keeping the dialogue open ---

Besides my comments on the manuscript proper, I wanted to address some of the topics discussed by the authors in their rebuttal.

Why we do not discuss other species more?

While I agree with the authors in that, when space is limited, not all topics can be discussed, I still think it would have benefitted the manuscript to spend some time addressing the species closest to the thylacine in the morphospace. In fact, I think it would have add support to the uniqueness of the thylacine phenotype as it shows how many different ecologies cluster in that region of the morphospace (this could even be a prime example of one-to-many morphofunctional relationship).

As for most of those species already having been discussed in Rovinsky et al (2021), maybe a more direct sign-posting should be included (e.g. “see Rovinsky et al (2021) for an in-depth discussion of X and Y species’ morphology compared with the thylacine’s”). While I praise the authors for the broad-picture scope of this study, combining findings from different disciplines, I feel that the fact that this manuscript and Rovinsky et al (2021) are companion pieces and should be read together, is not stressed enough. I read Rovinsky et al (2021) again right before tackling this review and it added great context to this revised manuscript.

Why is our emphasis on the wolf-thylacine comparison?

The authors claim that they keep the focus on the wolf because of (1) high public profile (do they mean popularity?) and (2) existing literature plus popular science. I read the rebuttal before the manuscript and my first thought was that this read like a cop-out at best, or an attempt to be “media-worthy” at worst. Given my previous concerns regarding a certain narrative being pushed, it’s fair to say that I started this review with quite an antagonistic mindset. Luckily, although the authors brush over the changes made to the manuscript to reduce the focus on the wolf in their rebuttal, those are significant and have truly changed my opinion on the manuscript. However, at a time when scientific research is often misconstrued by the public, lack of scientific rigor is often used as an excuse to cut funding (e.g. Argentinian government on CONICET), and the press is only interested in fluff science with clickbait-y results, I believe that we should refrain from letting high public profiles and popular science interfere with how we approach research. Please note that this is not a criticism to the authors, but a reflection on how scientific publications seem to be changing agendas from sound research to popular appeal and this is starting to seep into how we design or justify our research.

Back to the original question though, why keep the focus on the wolf, please refer to my comments on the discrepancy between Figure 2 and Suppl. Table 1. I believe in most cases the manuscript should shift to a comparison with wolf-like canids (i.e., the Subtribe Canina) instead of hammering on the uniqueness of grey wolfs and dingoes. In my opinion, this would increase the relevance of the findings presented here, and inspire new research avenues (for instance, looking at the cranial development of other Canina).
